# Supplementary material for: Stereoselective one-pot synthesis of polypropionates
Source: Nat Commun. 2017 Sep 25;8:679. doi: 10.1038/s41467-017-00787-y (PMC5612996; doi:10.1038/s41467-017-00787-y)
Supplement: Supplementary file 1 — Supplementary Information [file 41467_2017_787_MOESM1_ESM.pdf]

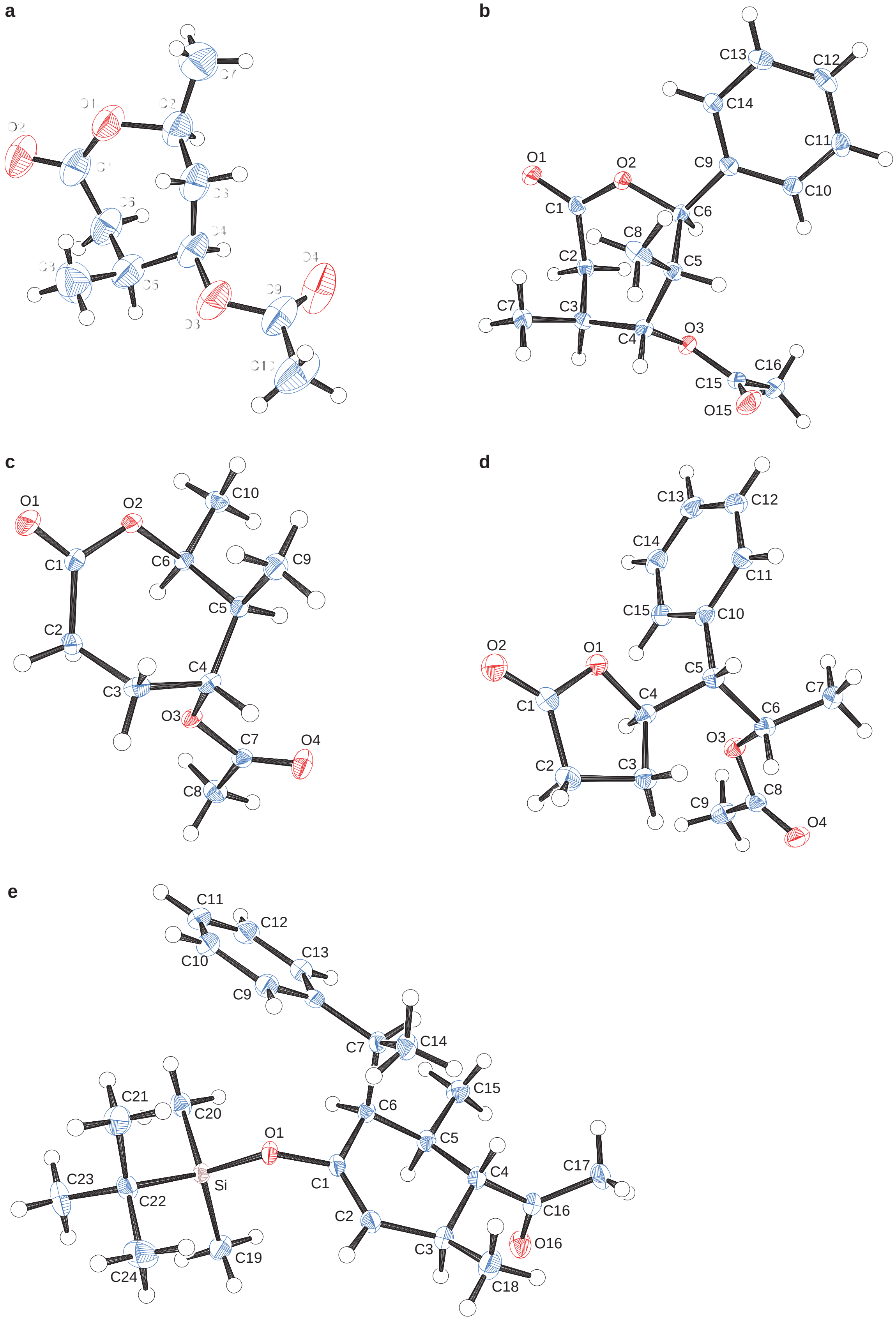

Supplementary Figure 1. Additional X-ray crystal structures. **a**, Compound **16**; **b**, compound **23**; **c**, compound **29**; **d**, compound **34**; **e**, compound **40**.

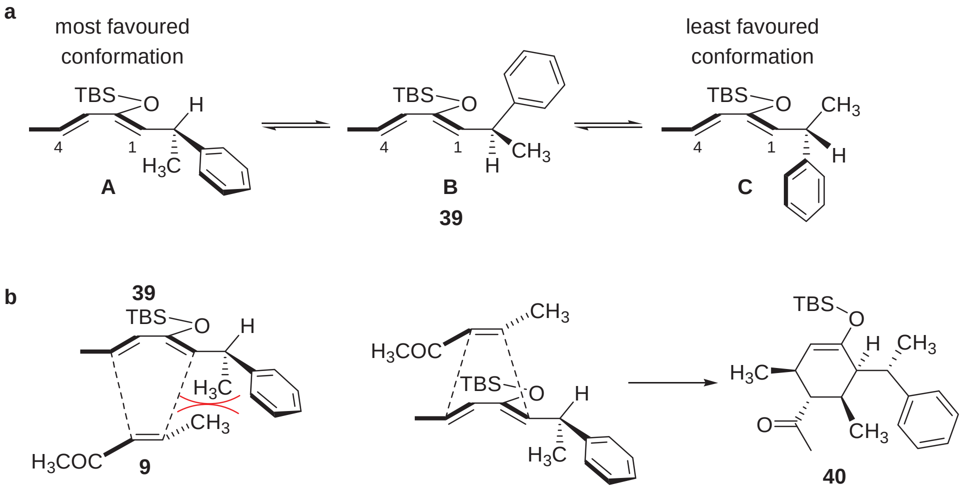

Supplementary Figure 2. Proposed rationale for the facial selectivity in the *exo*-Diels–Alder reaction of diene 39 and dienophile 9. a, The possible conformations of **39** based on the orientation of the substituents of the chiral carbon attached to C1 of the diene, with the most favoured conformation (**A**) having the least steric interaction with the silyloxy group. **b**, The approach of the dienophile **9** in one face of the diene **39** in **A** conformation is blocked by the presence of methyl group whereas the approach on the opposite face is less hindered, leading to the favourable formation of the cycloadduct **40**.

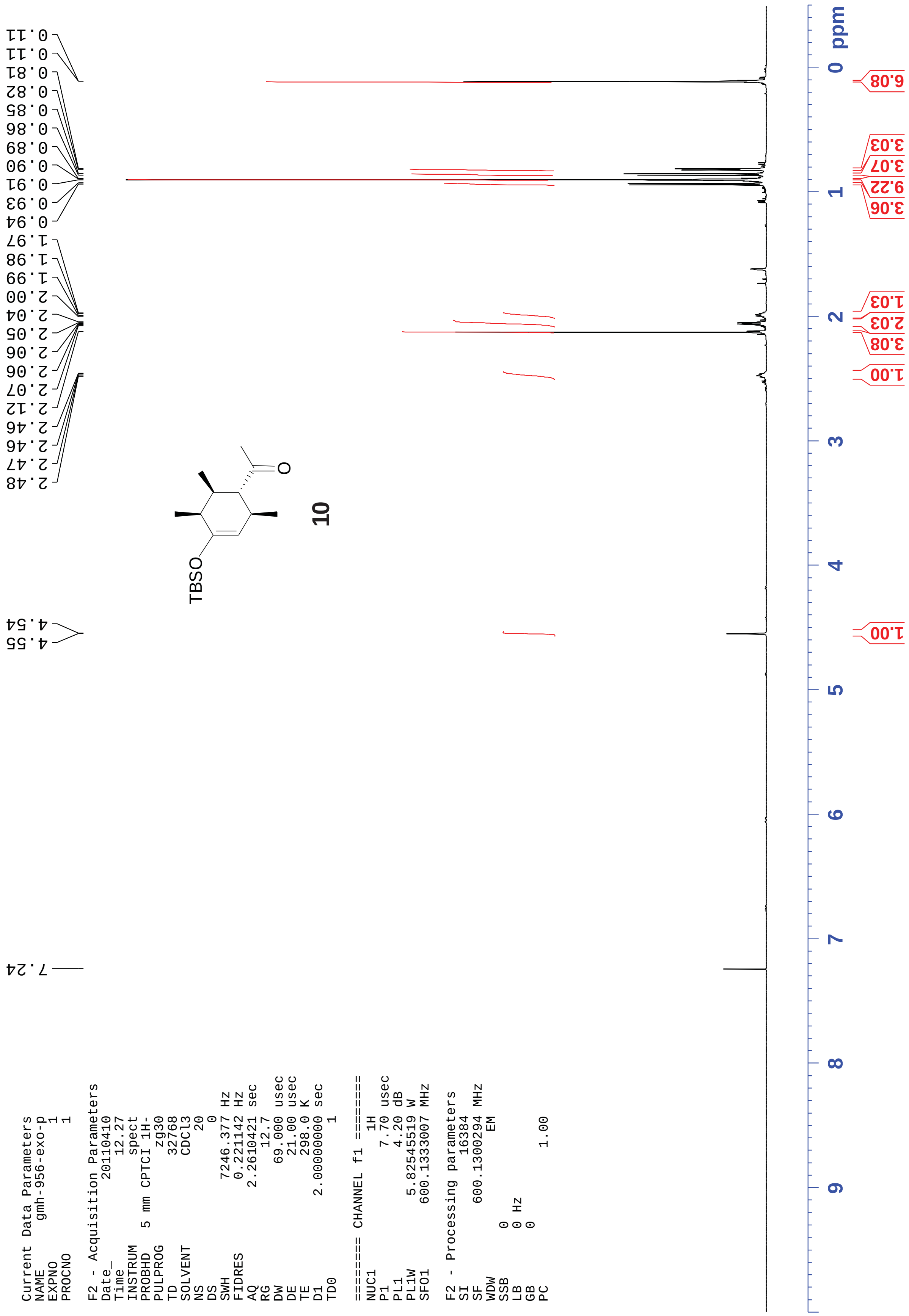

Supplementary Figure 3. ^{1}**H NMR spectrum of compound 10.**

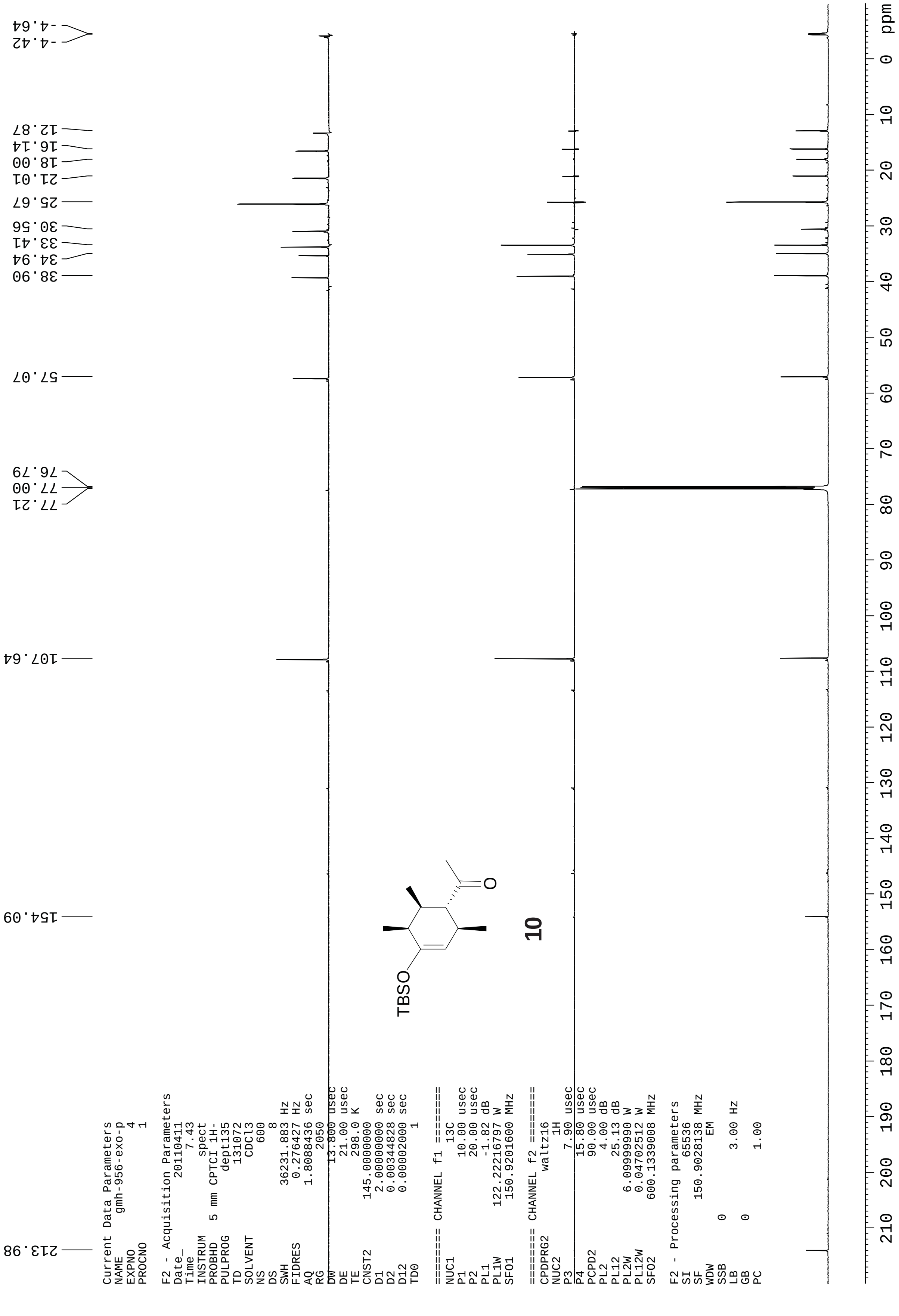

Supplementary Figure 4. ^{13}C and DEPT NMR spectra of compound 10.

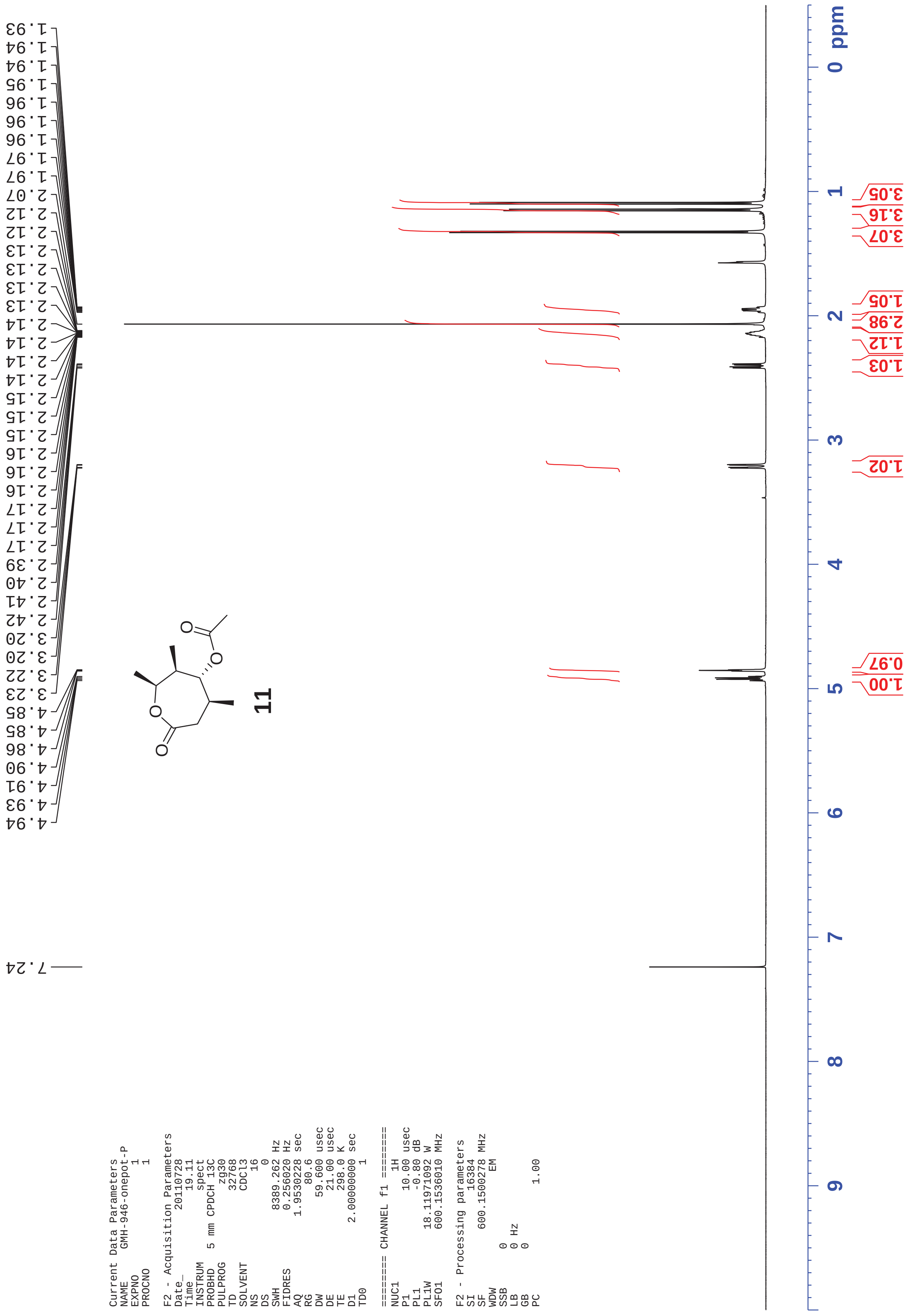

Supplementary Figure 5. ^{1}H NMR spectrum of compound 11.

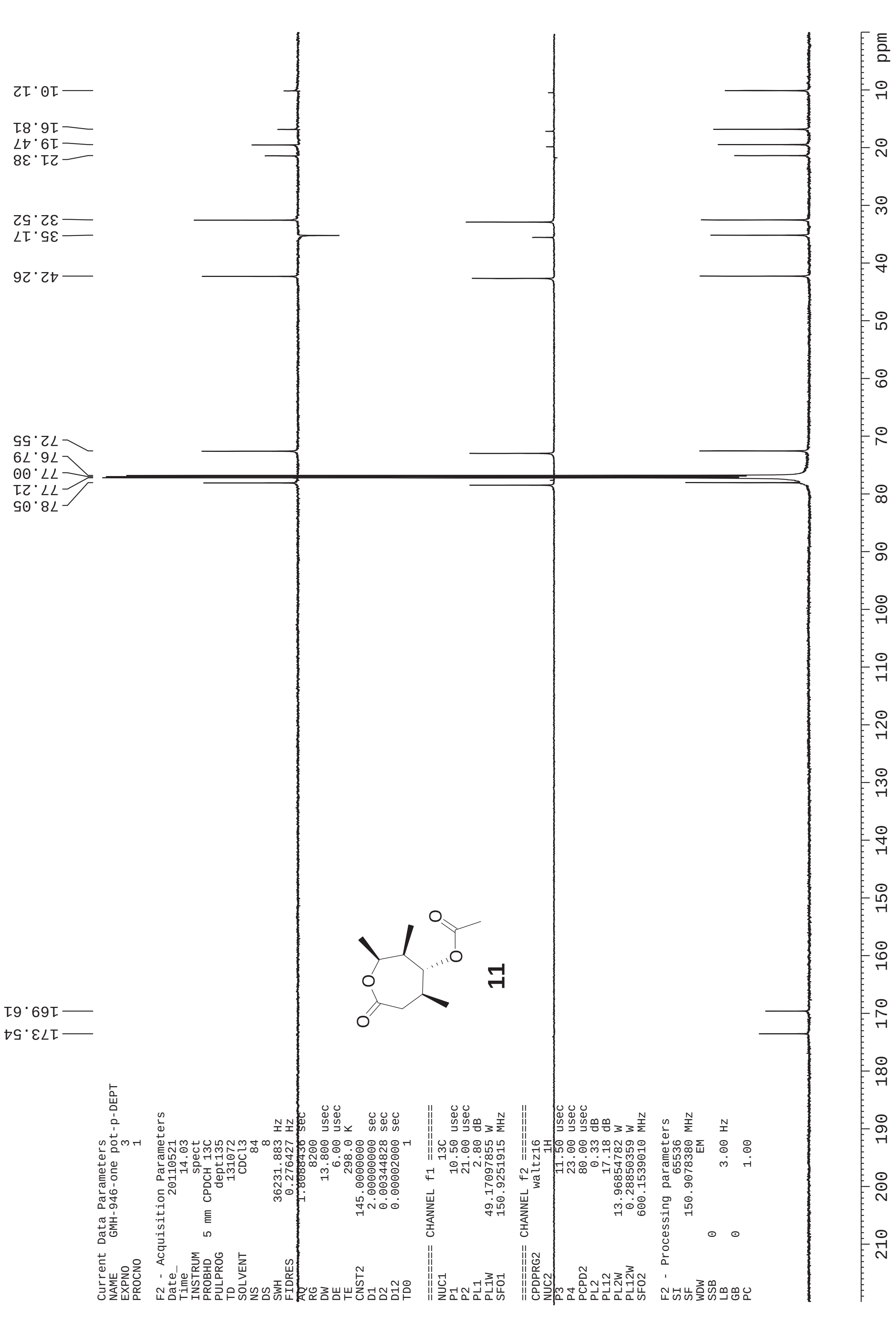

Supplementary Figure 6. ^{13}C and DEPT NMR spectra of compound 11.

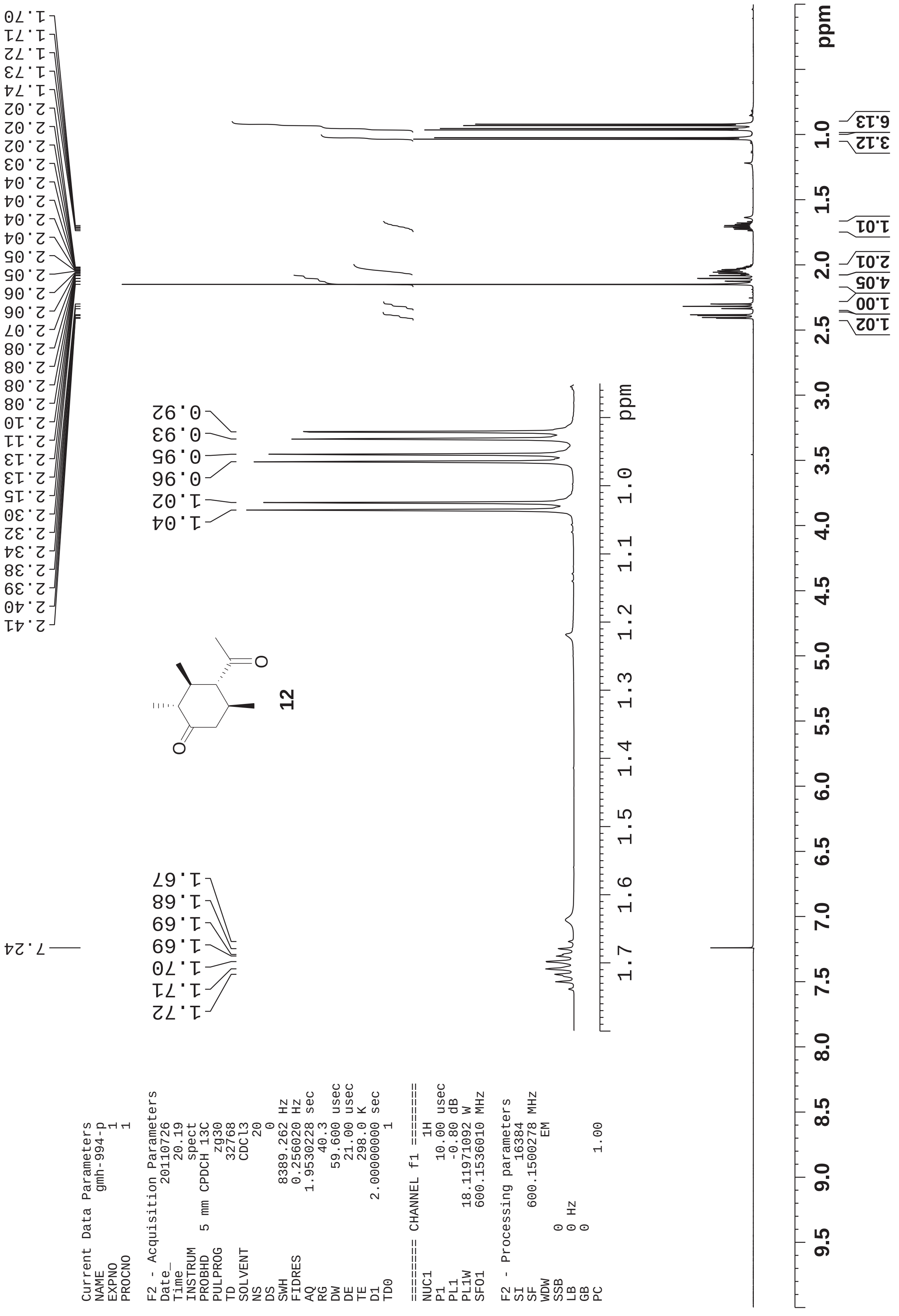

Supplementary Figure 7. ^{1}H NMR spectrum of compound 12.

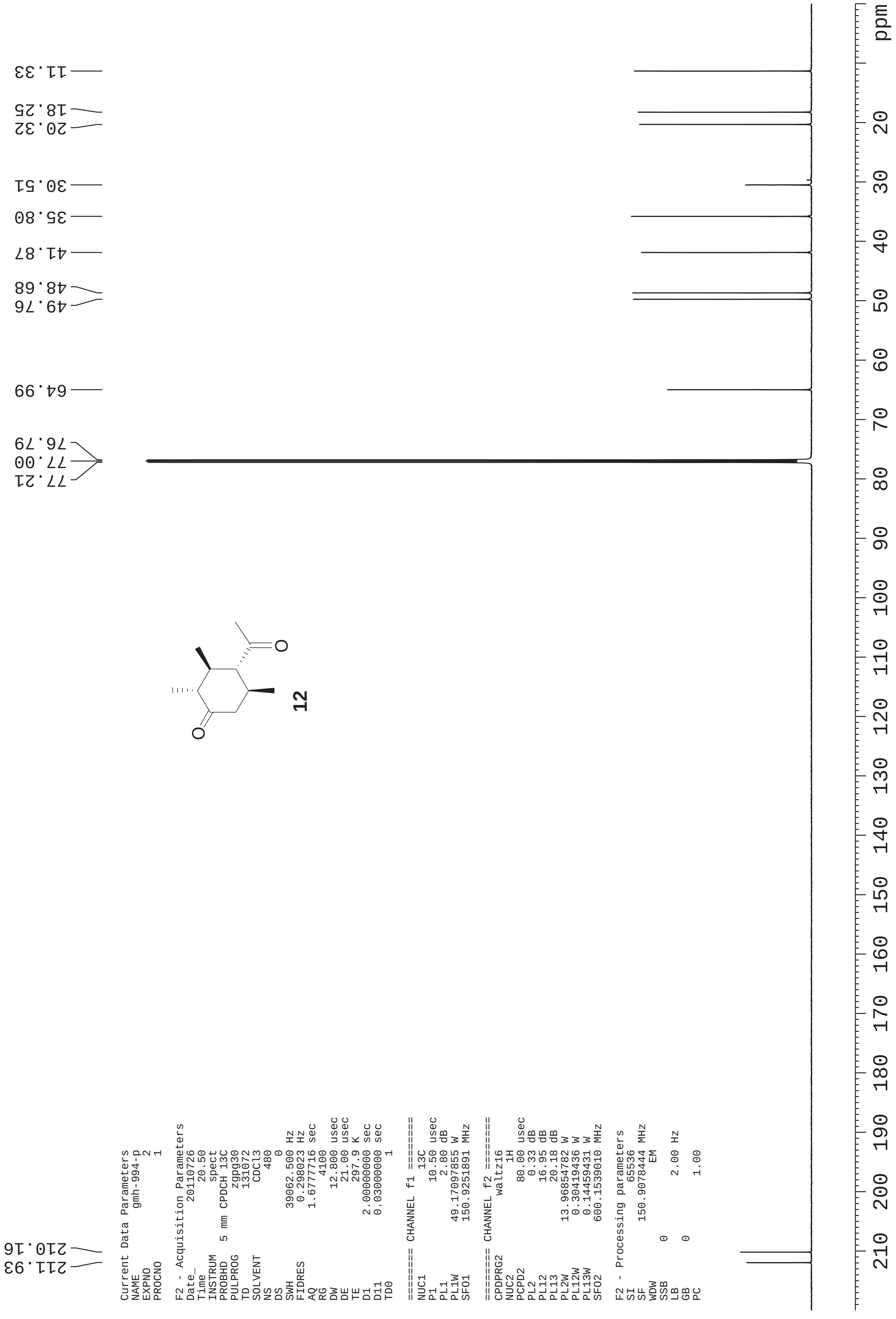

Supplementary Figure 8. ^{13}C NMR spectrum of compound 12.

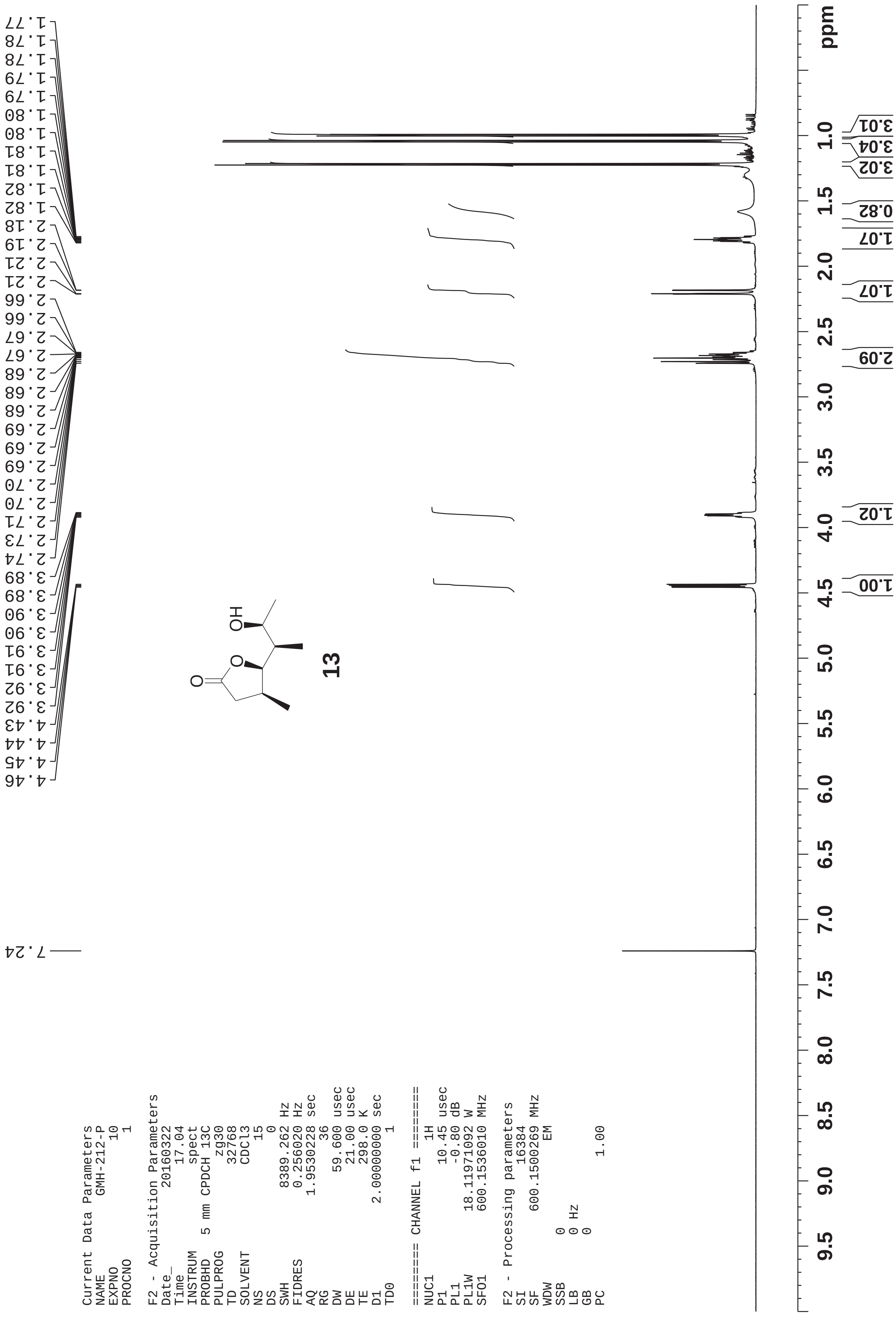

Supplementary Figure 9. ^{1}H NMR spectrum of compound 13.

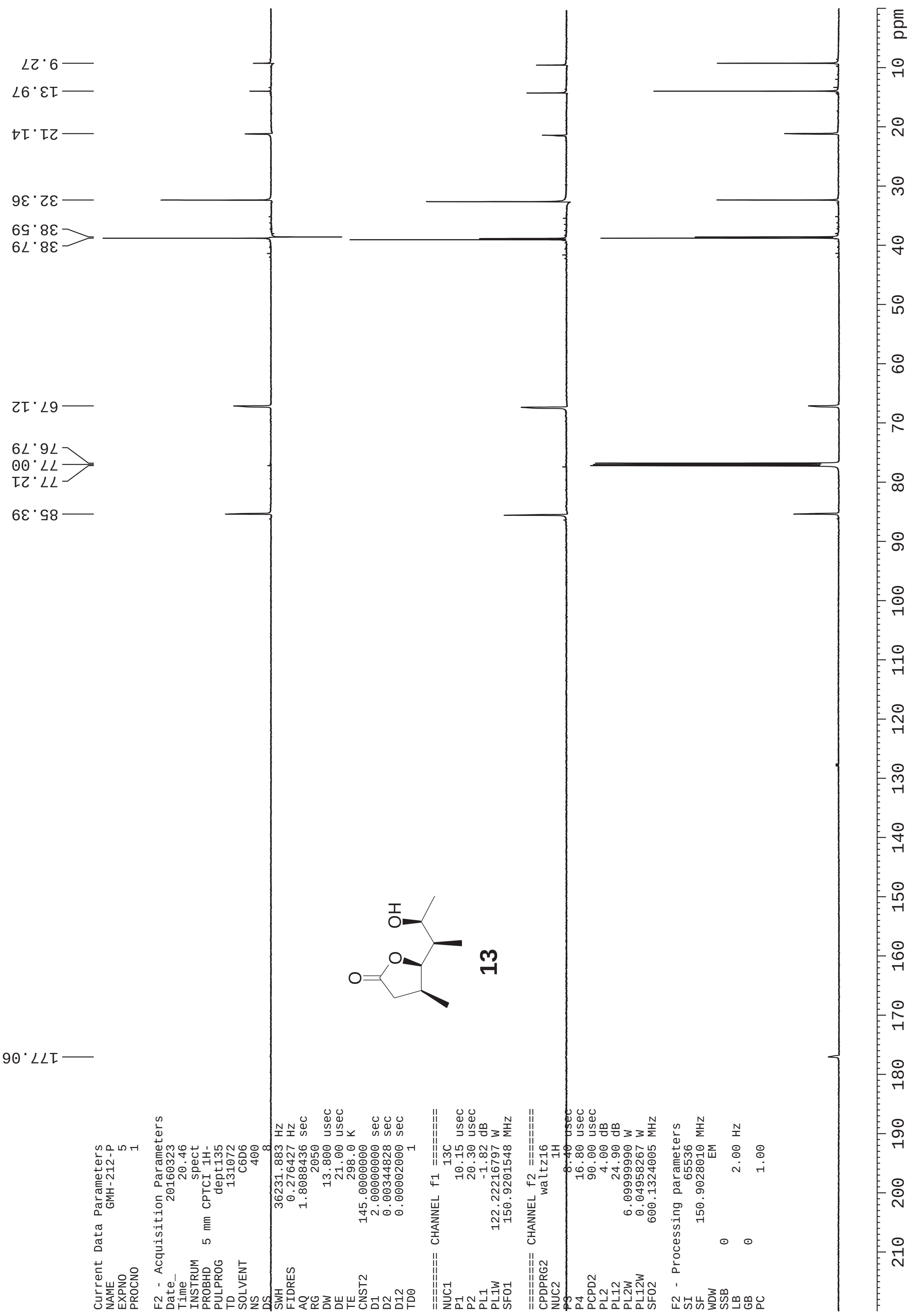

Supplementary Figure 10. ^{13}C and DEPT NMR spectra of compound 13.

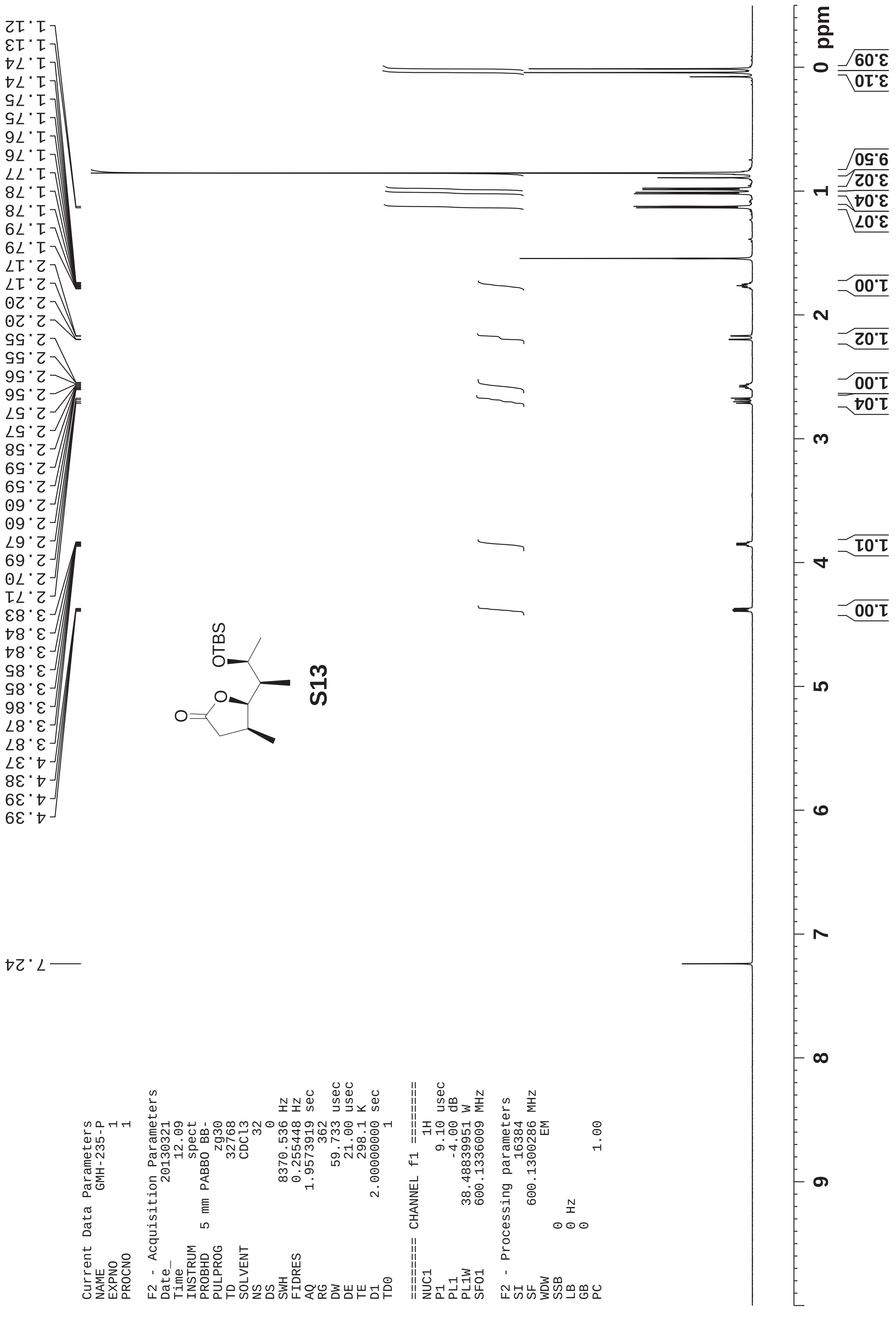

Supplementary Figure 11. **^{1}H NMR spectrum of compound S13.**

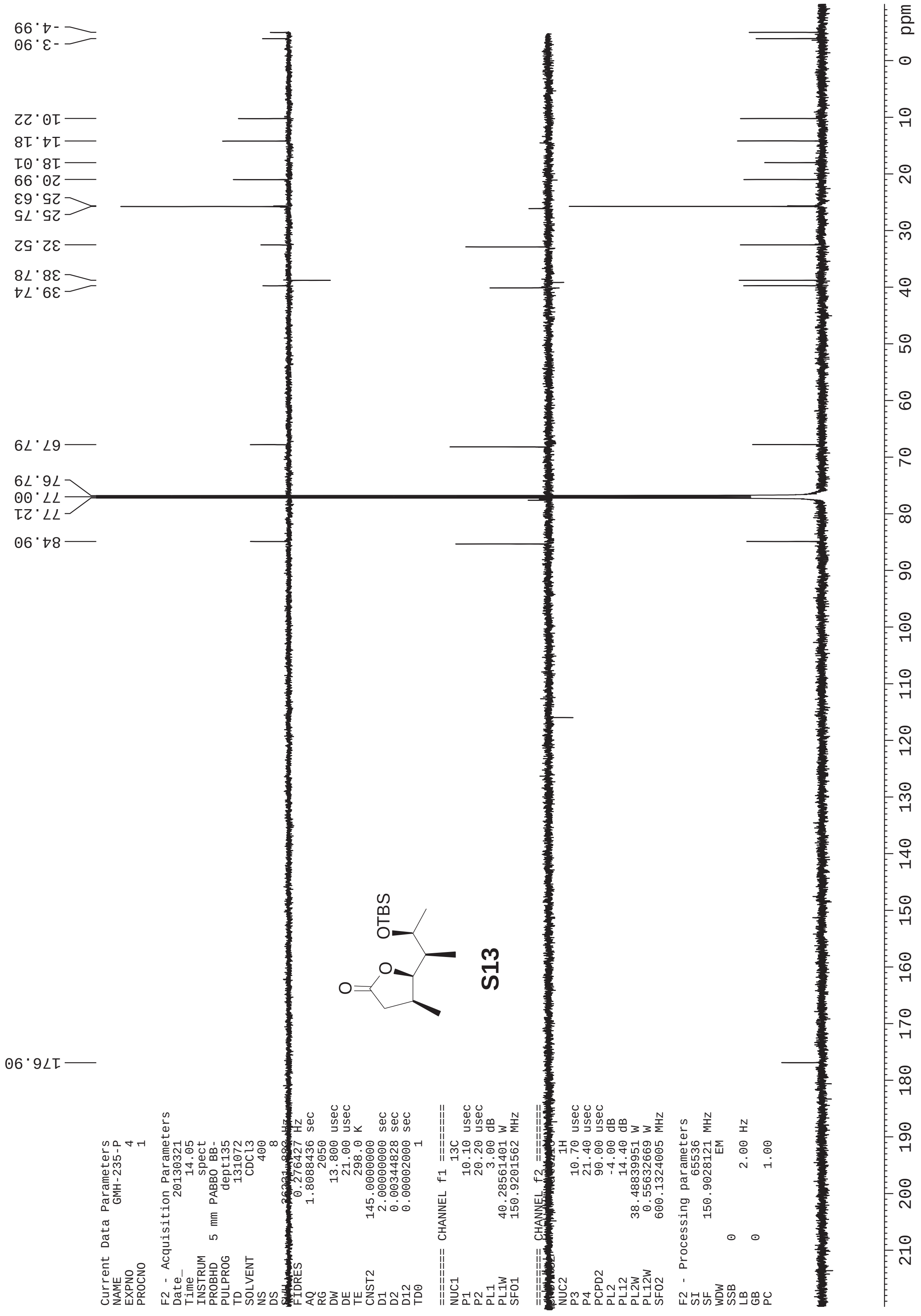

Supplementary Figure 12. ^{13}C and DEPT NMR spectra of compound S13.

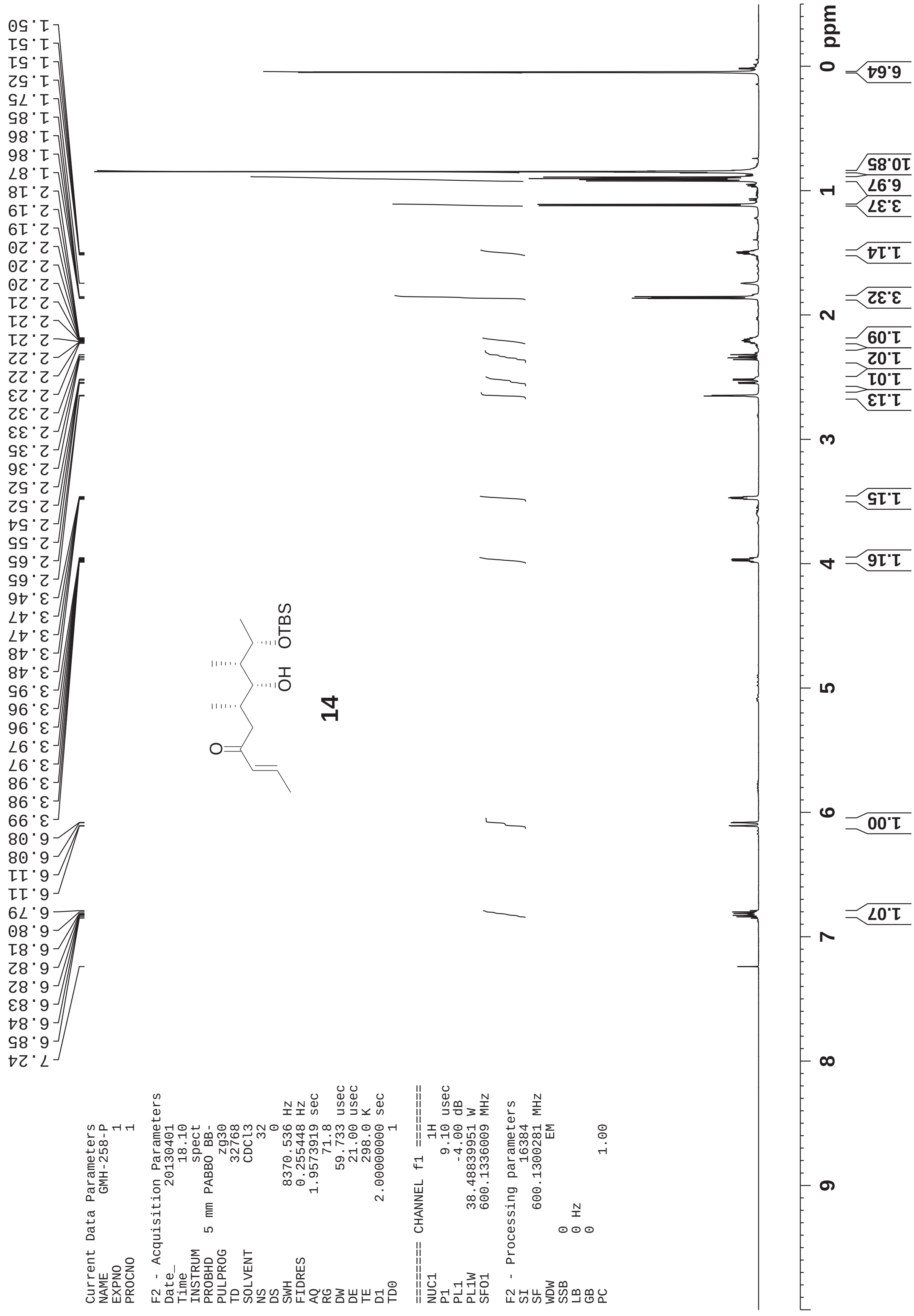

Supplementary Figure 13. **^{1}H NMR spectrum of compound 14.**

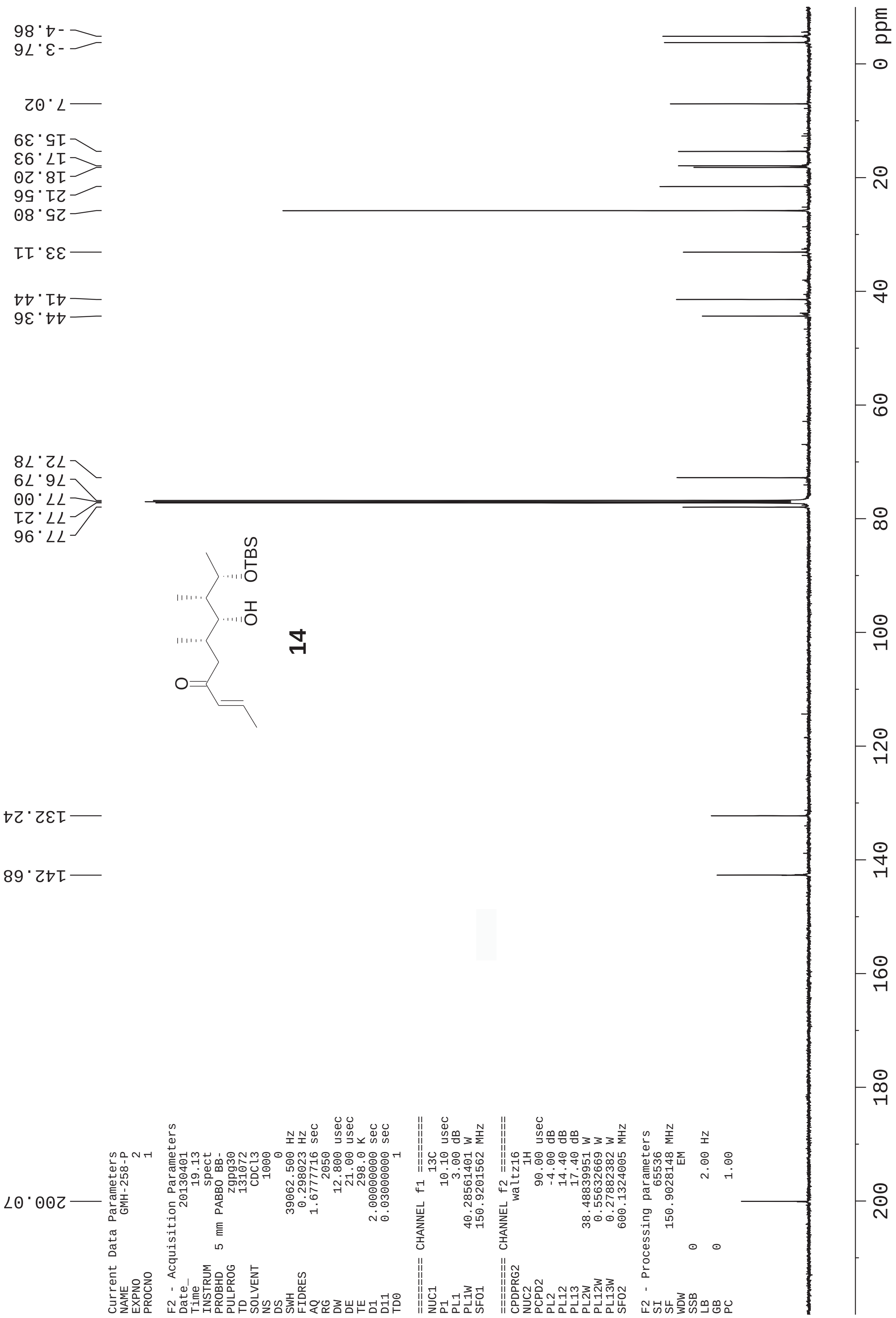

Supplementary Figure 14. ^{13}C NMR spectra of compound 14.

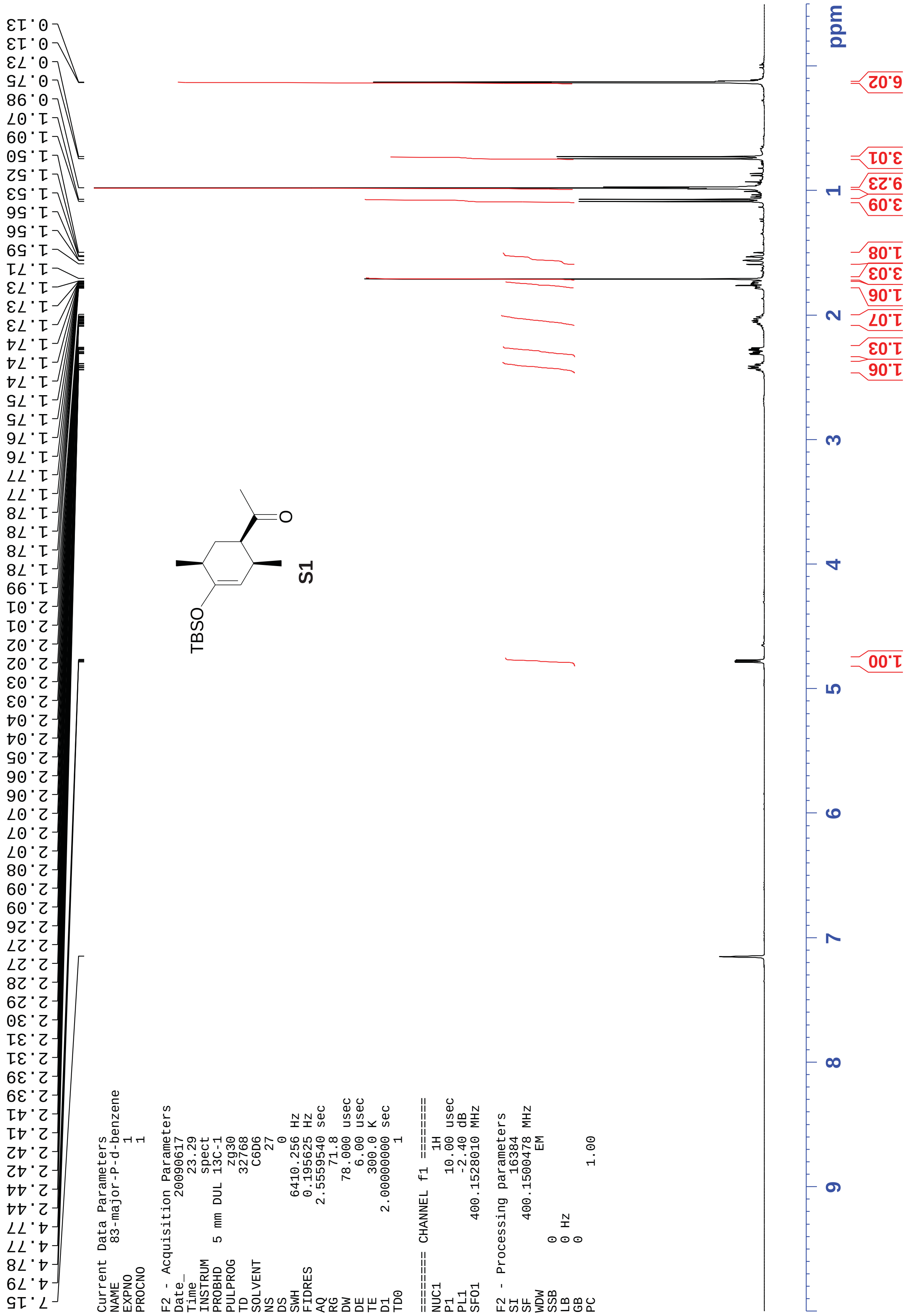

Supplementary Figure 15. ^{1}H NMR spectrum of compound S1.

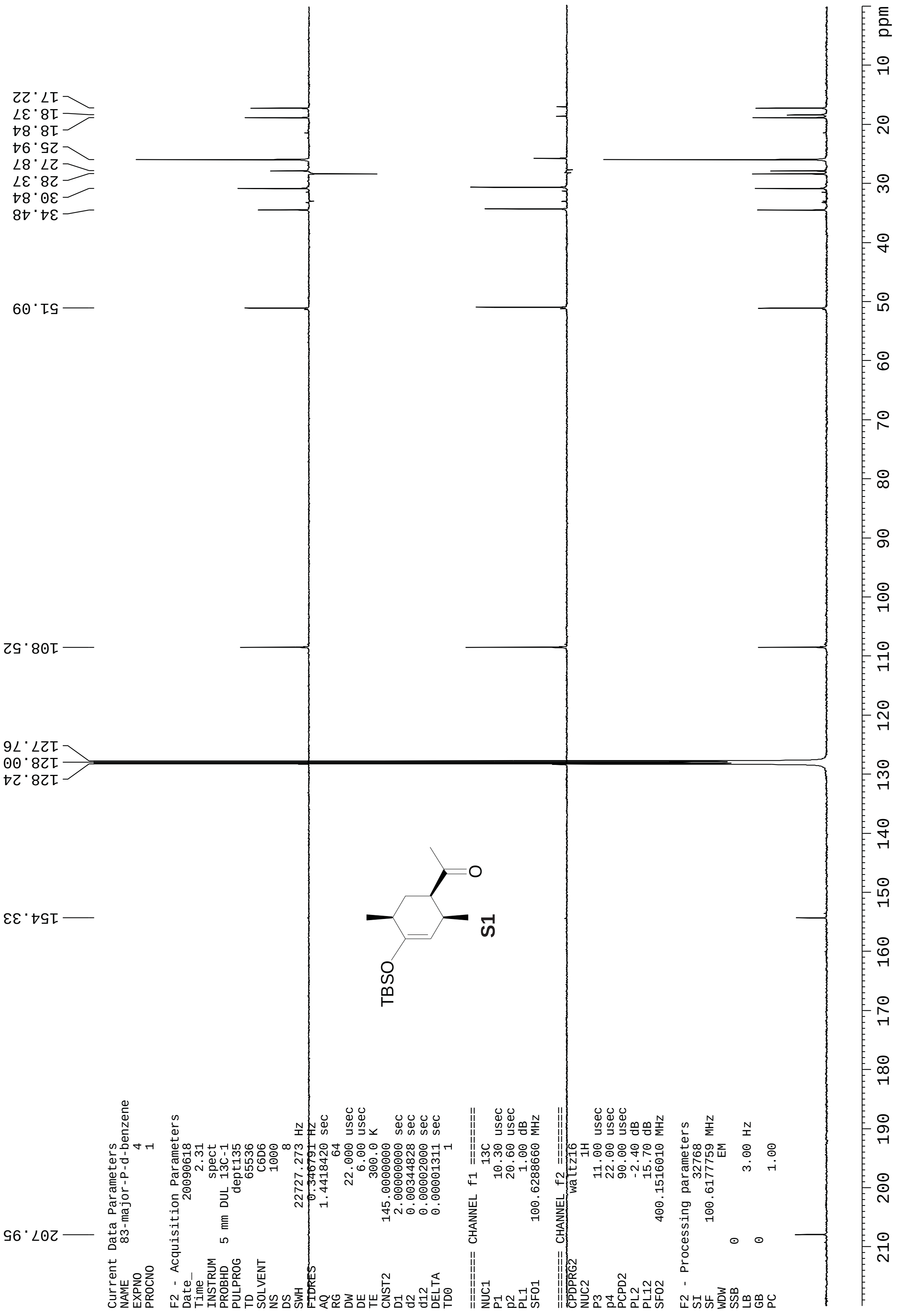

Supplementary Figure 16. ^{13}C and DEPT NMR spectra of compound S1.

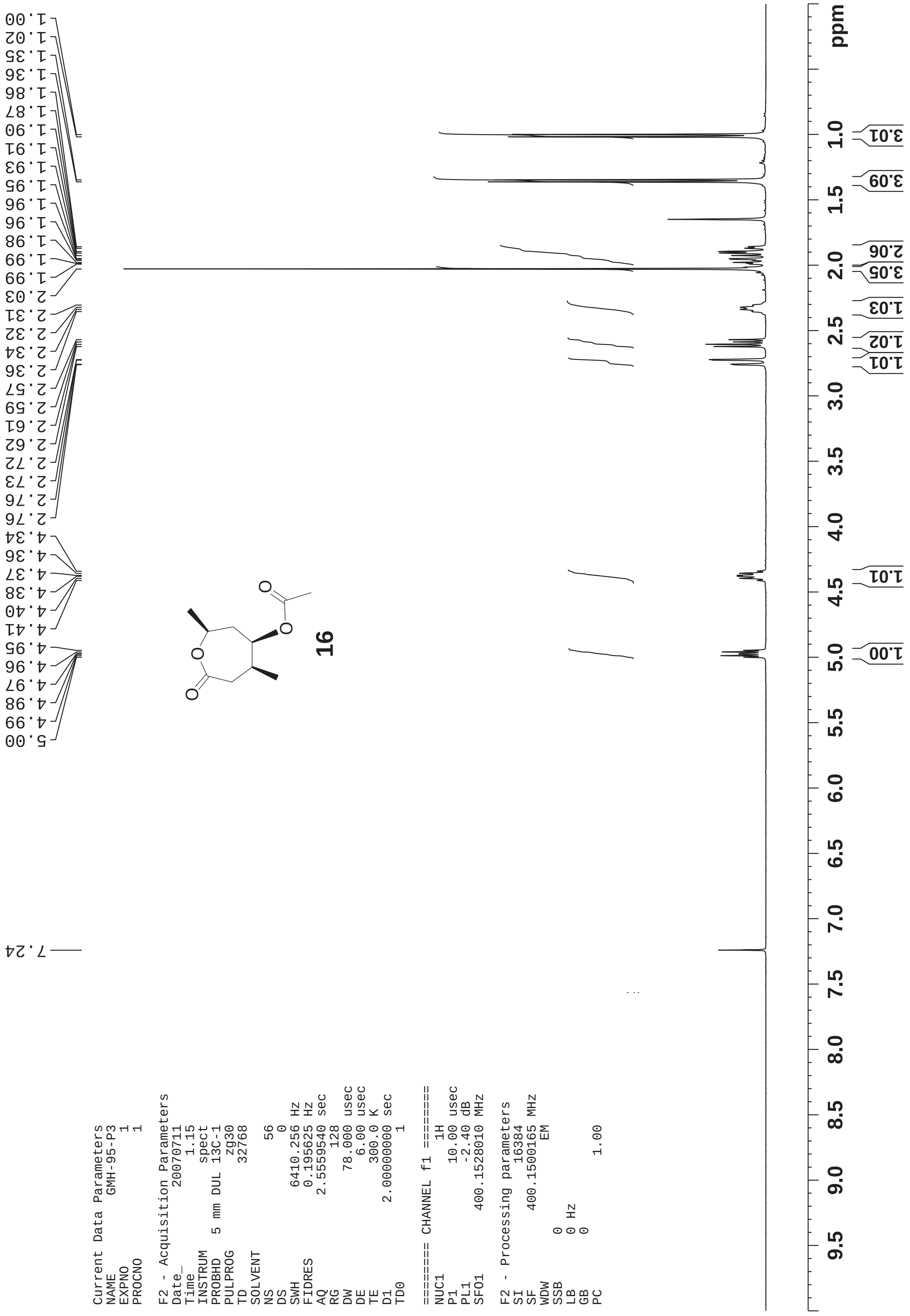

Supplementary Figure 17. ^{1}H NMR spectrum of compound 16.

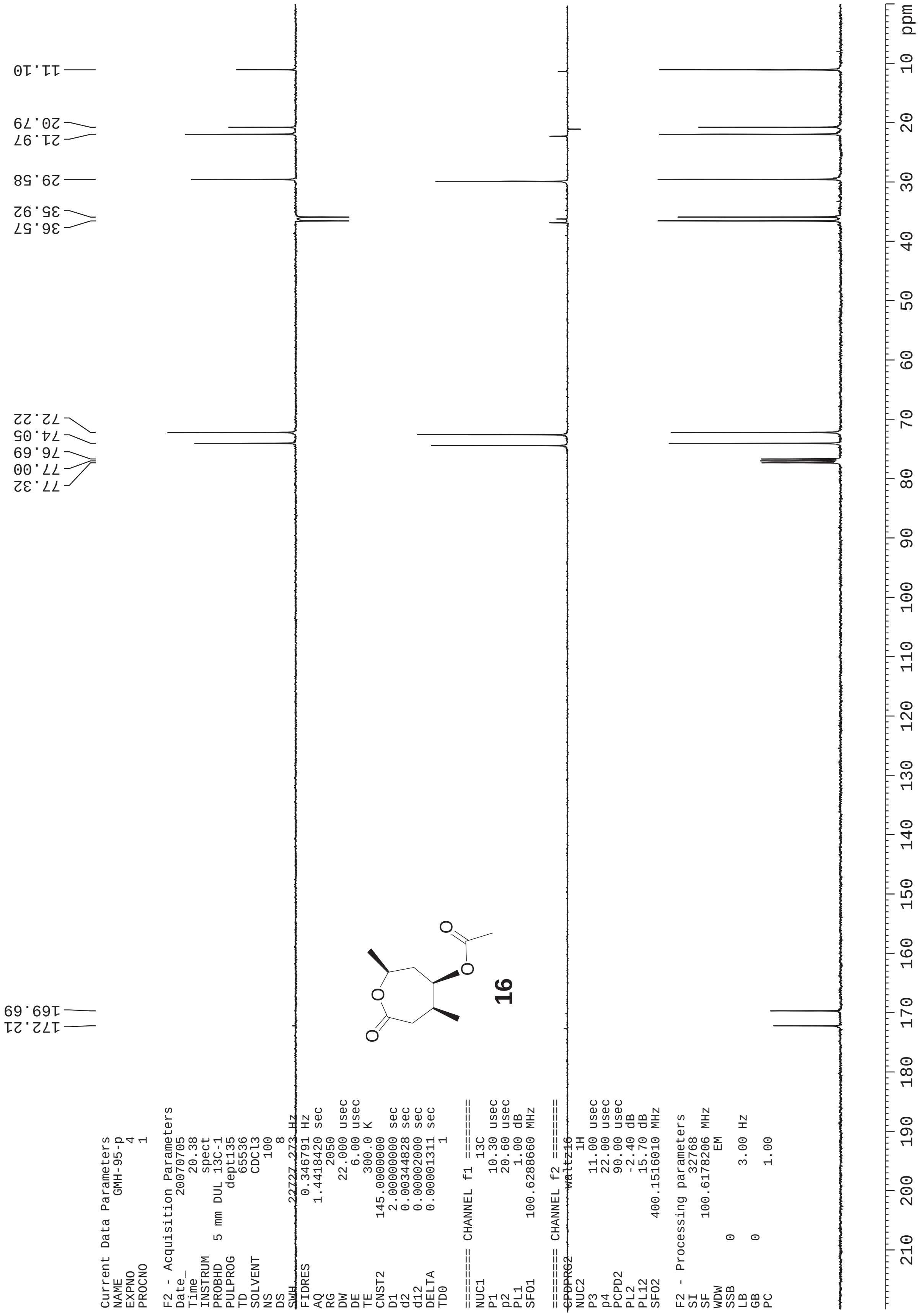

Supplementary Figure 18. ^{13}C and DEPT NMR spectra of compound 16.

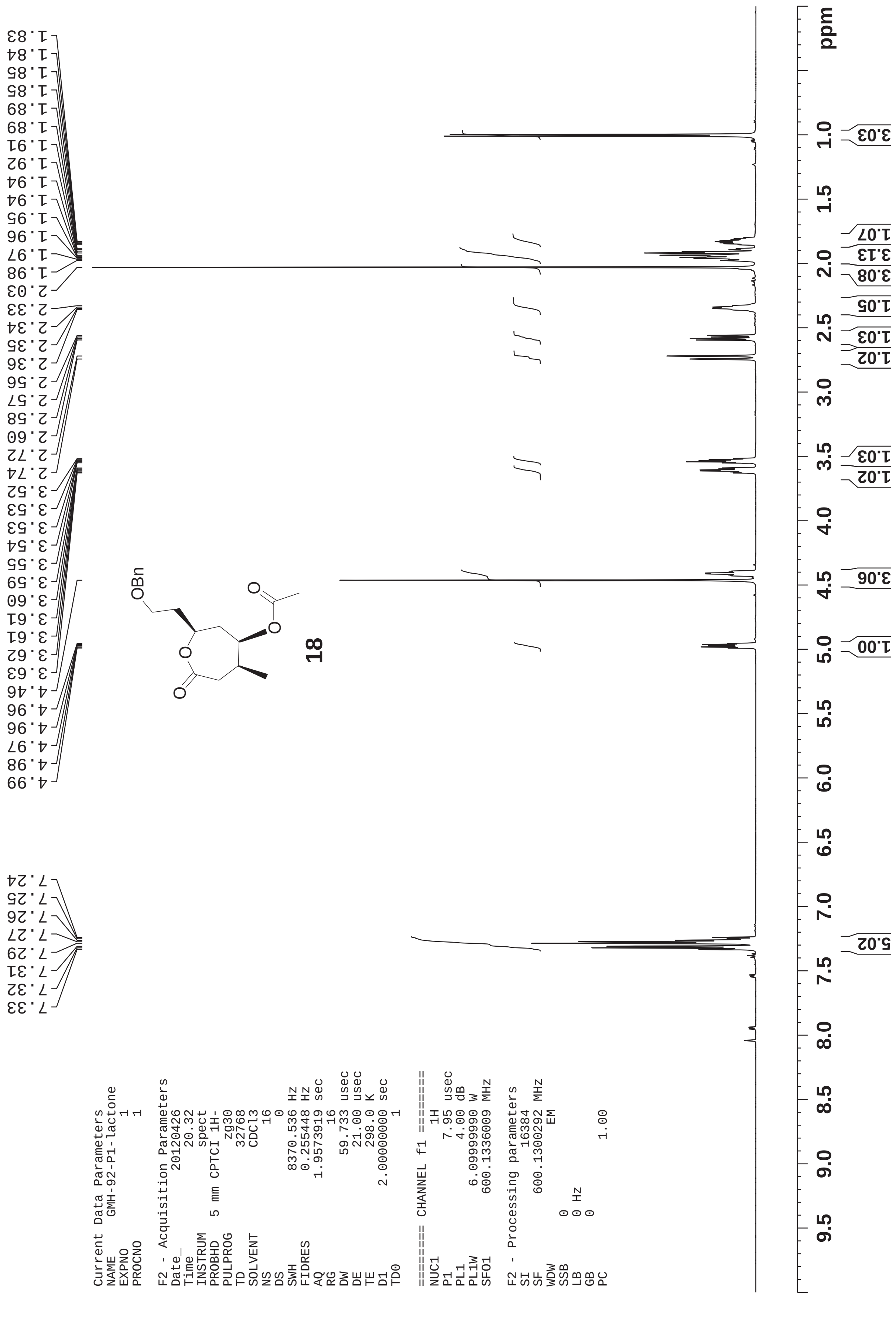

Supplementary Figure 19. ^{1}H NMR spectrum of compound 18.

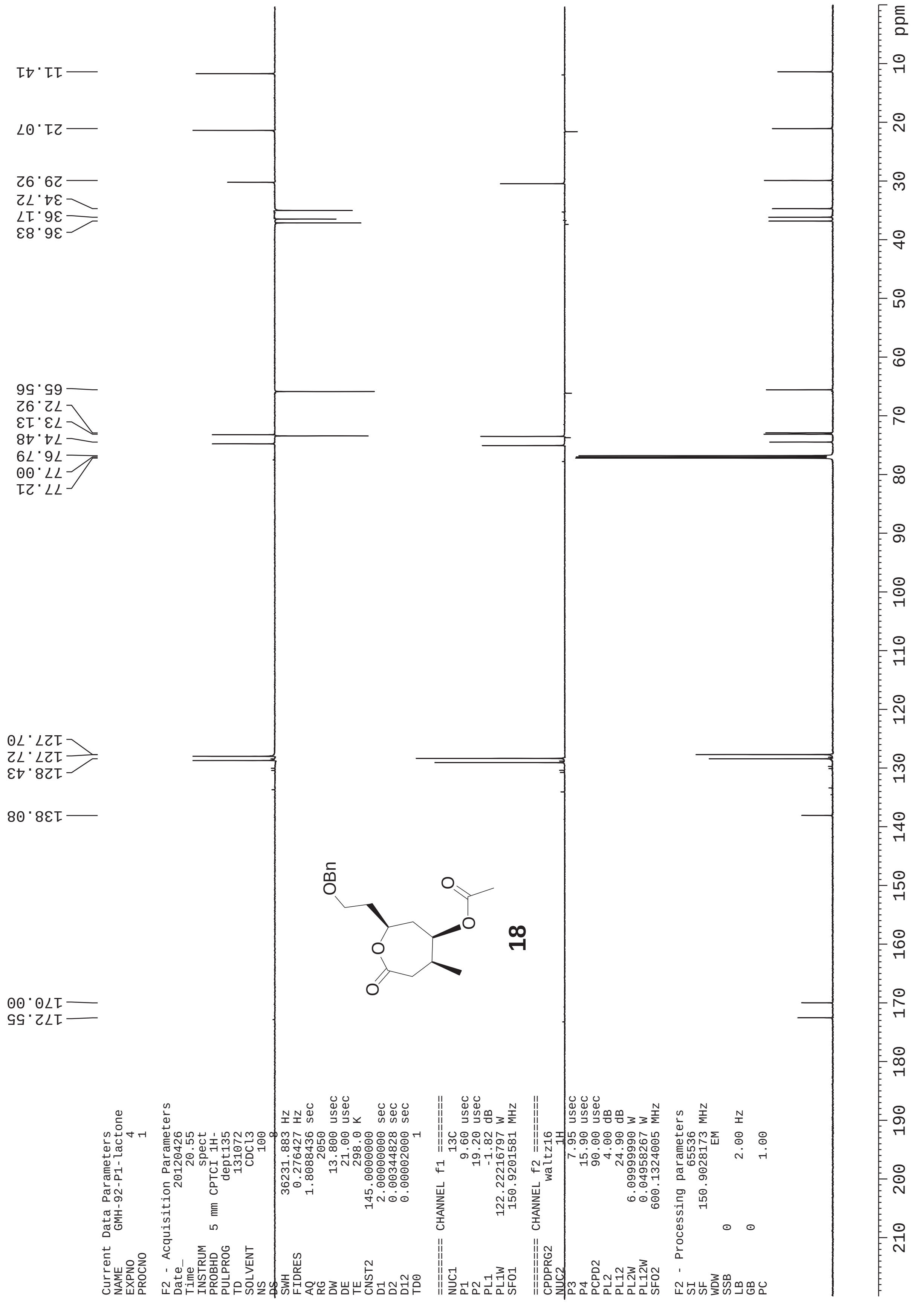

Supplementary Figure 20. ^{13}C and DEPT NMR spectra of compound 18.

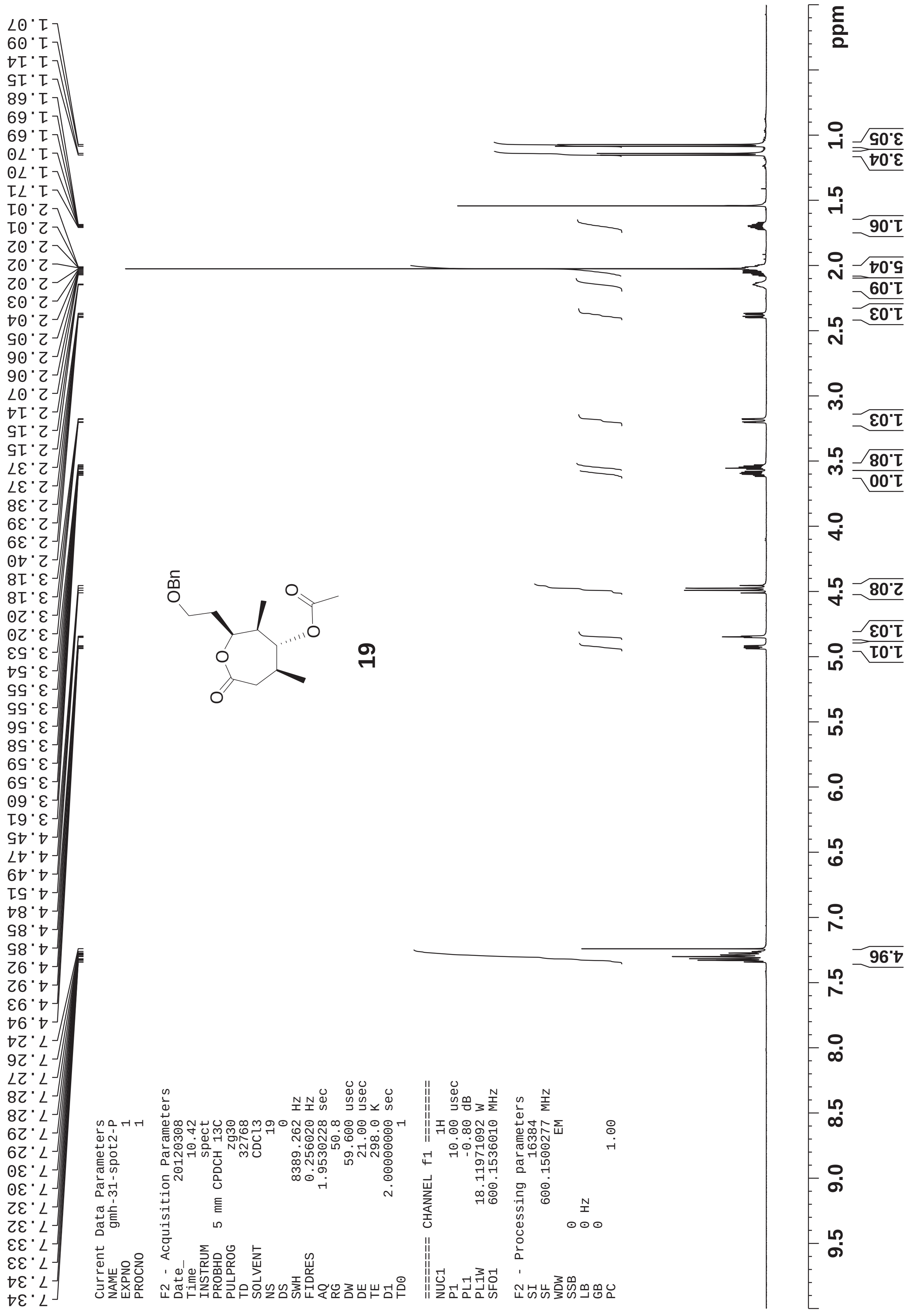

Supplementary Figure 21. ^{1}H NMR spectrum of compound 19.

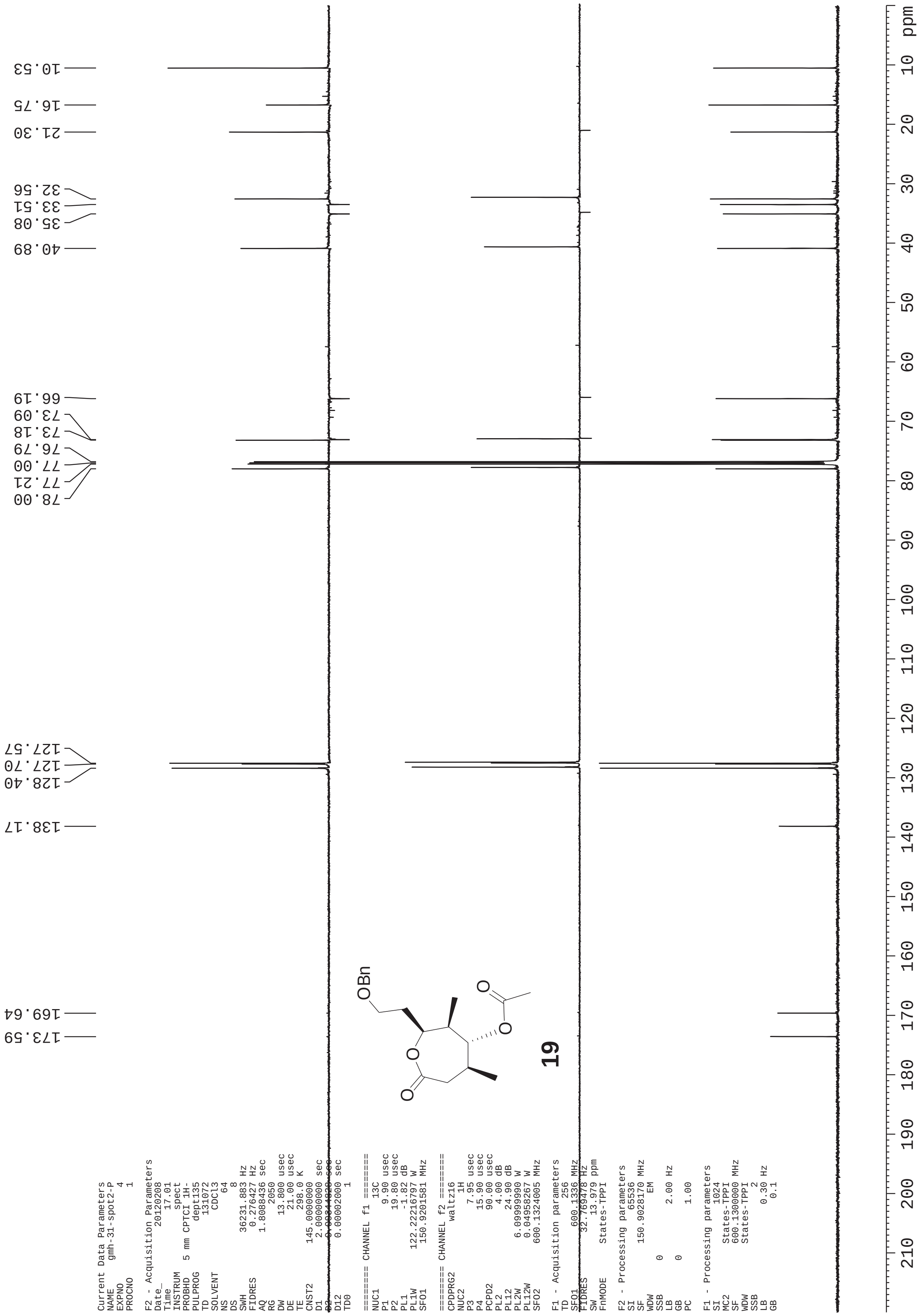

Supplementary Figure 22. ^{13}C and DEPT NMR spectra of compound 19.

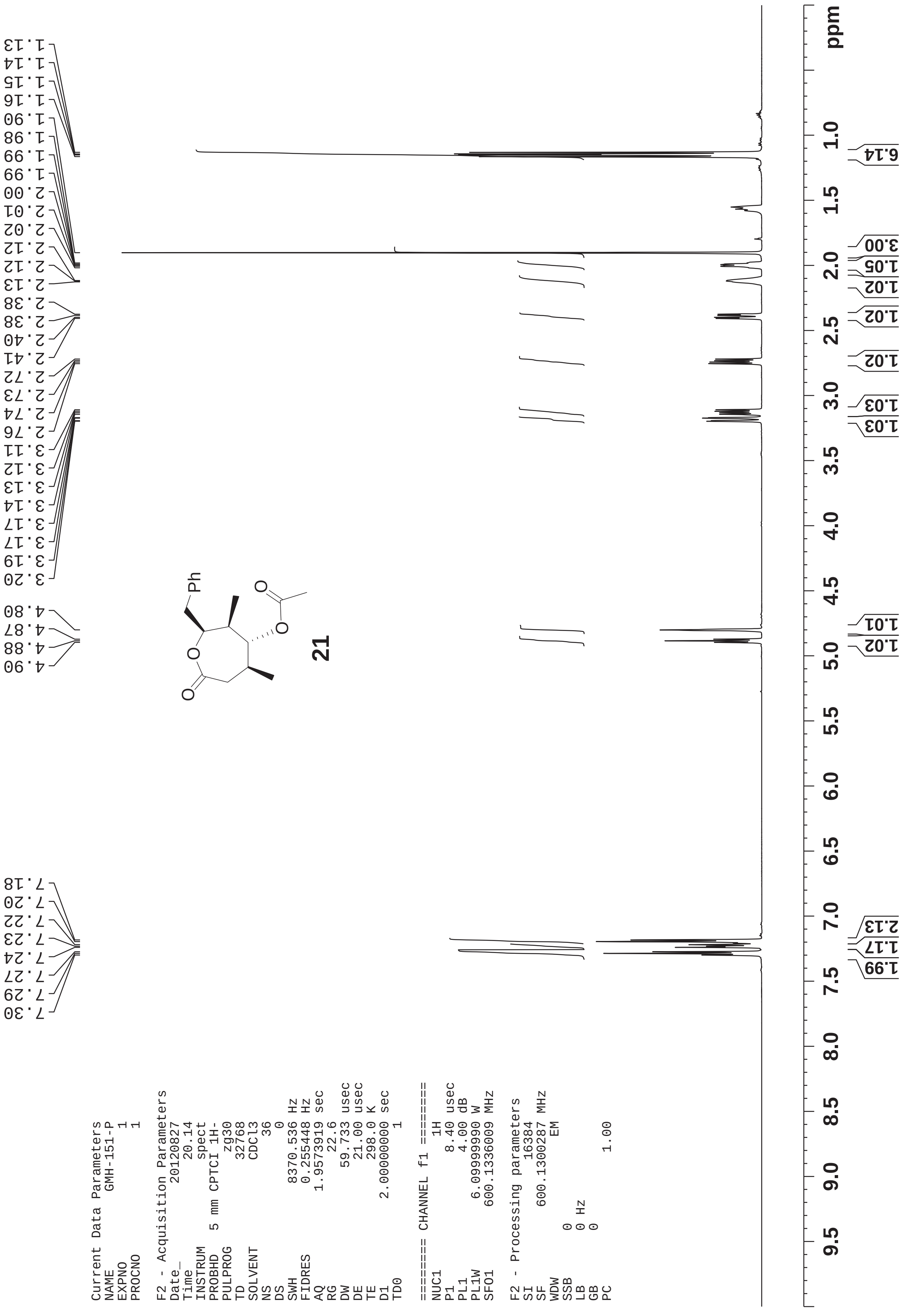

Supplementary Figure 23. ^{1}H NMR spectrum of compound 21.

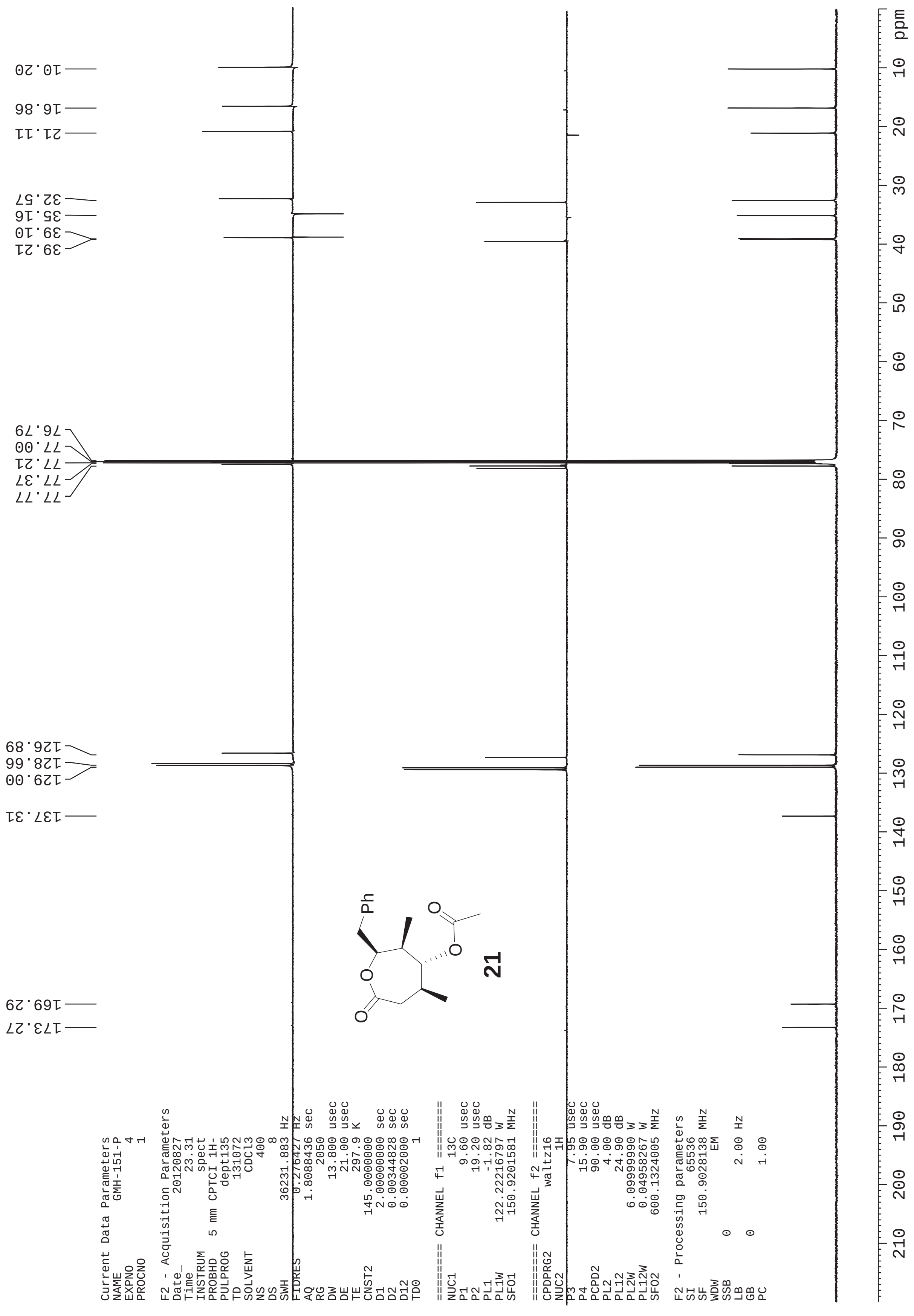

Supplementary Figure 24. ^{13}C and DEPT NMR spectra of compound 21.

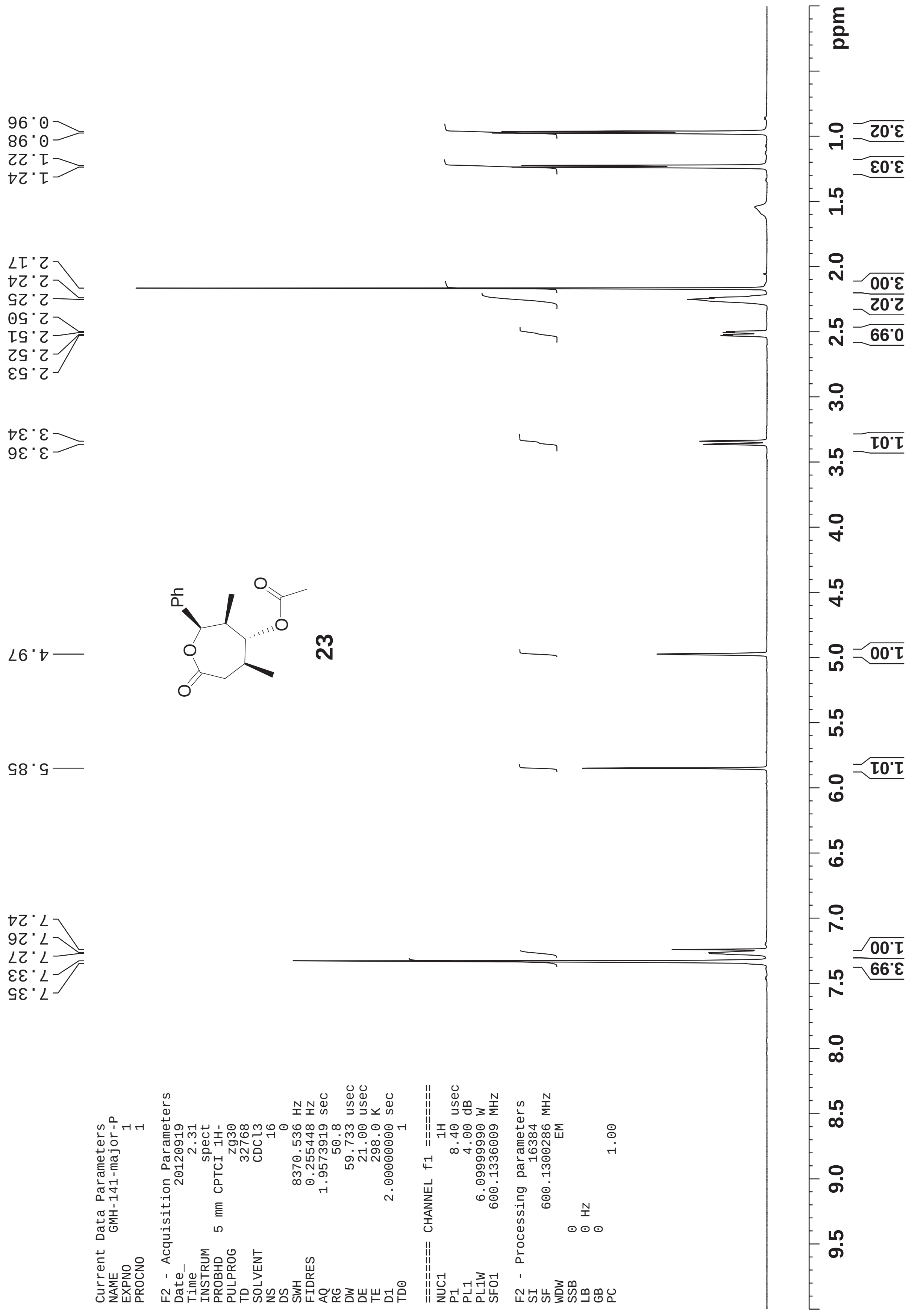

Supplementary Figure 25. ^{1}H NMR spectrum of compound 23.

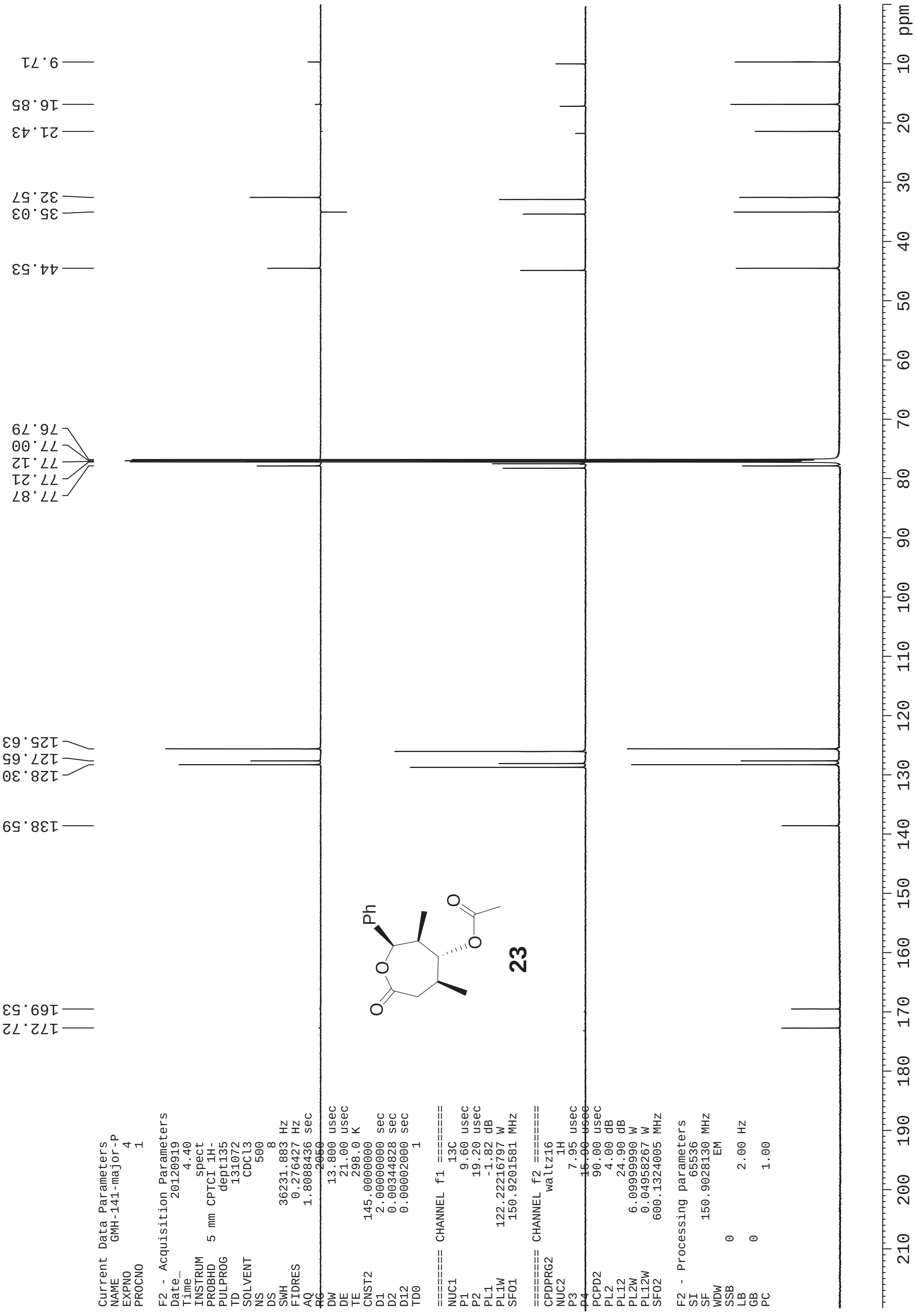

Supplementary Figure 26. ^{13}C and DEPT NMR spectra of compound 23.

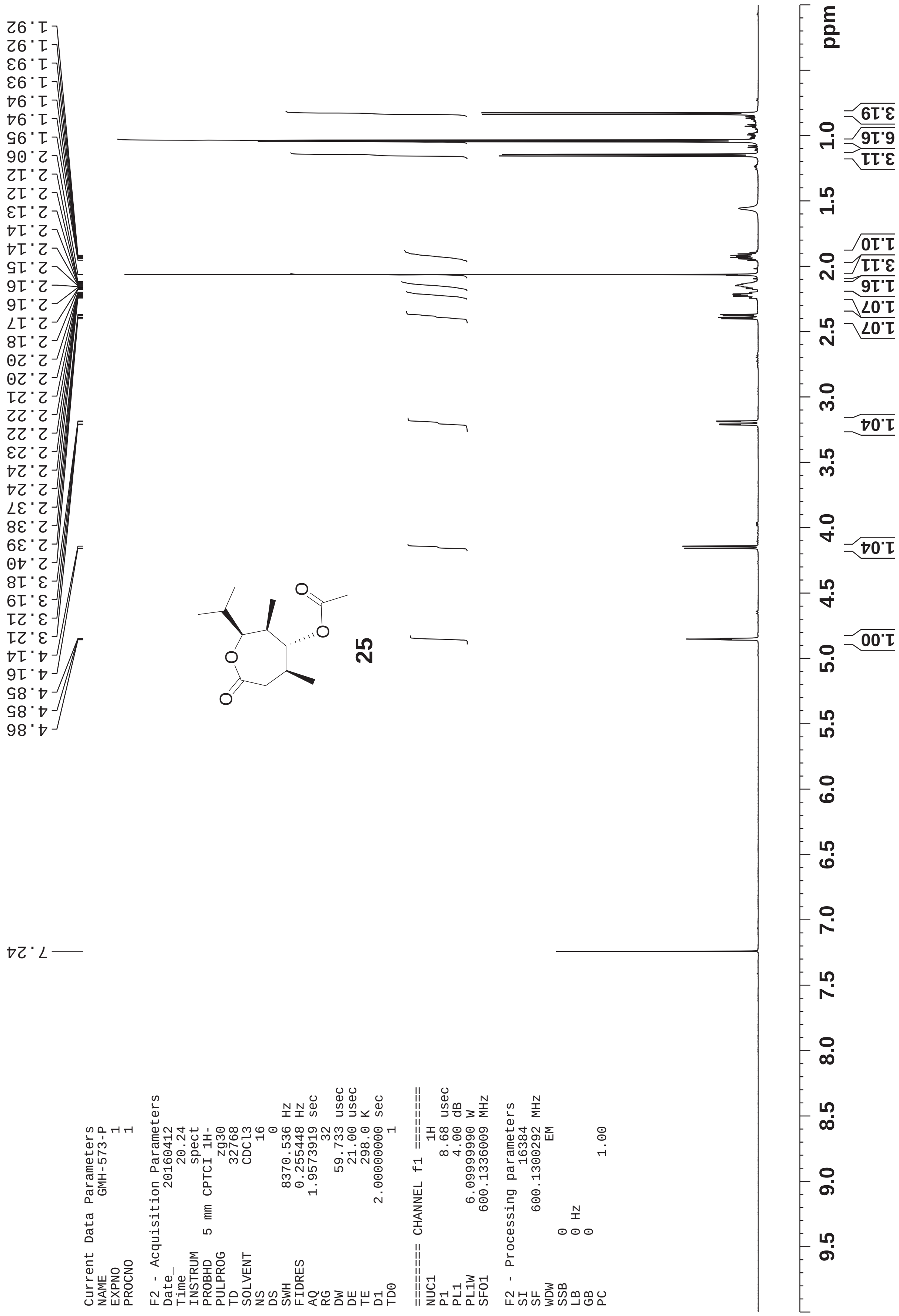

Supplementary Figure 27. ^{1}H NMR spectrum of compound 25.

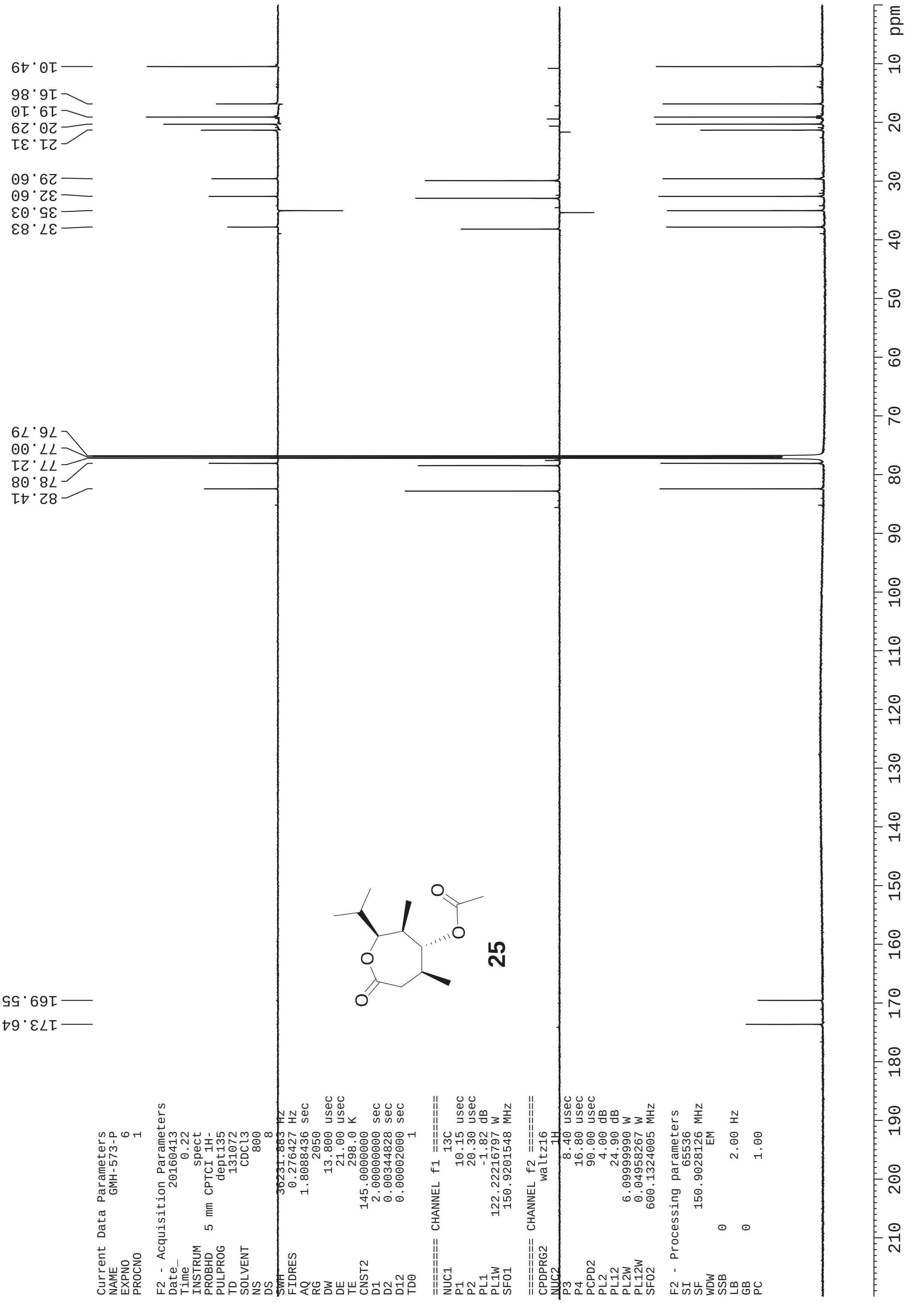

Supplementary Figure 28. ^{13}C and DEPT NMR spectra of compound 25.

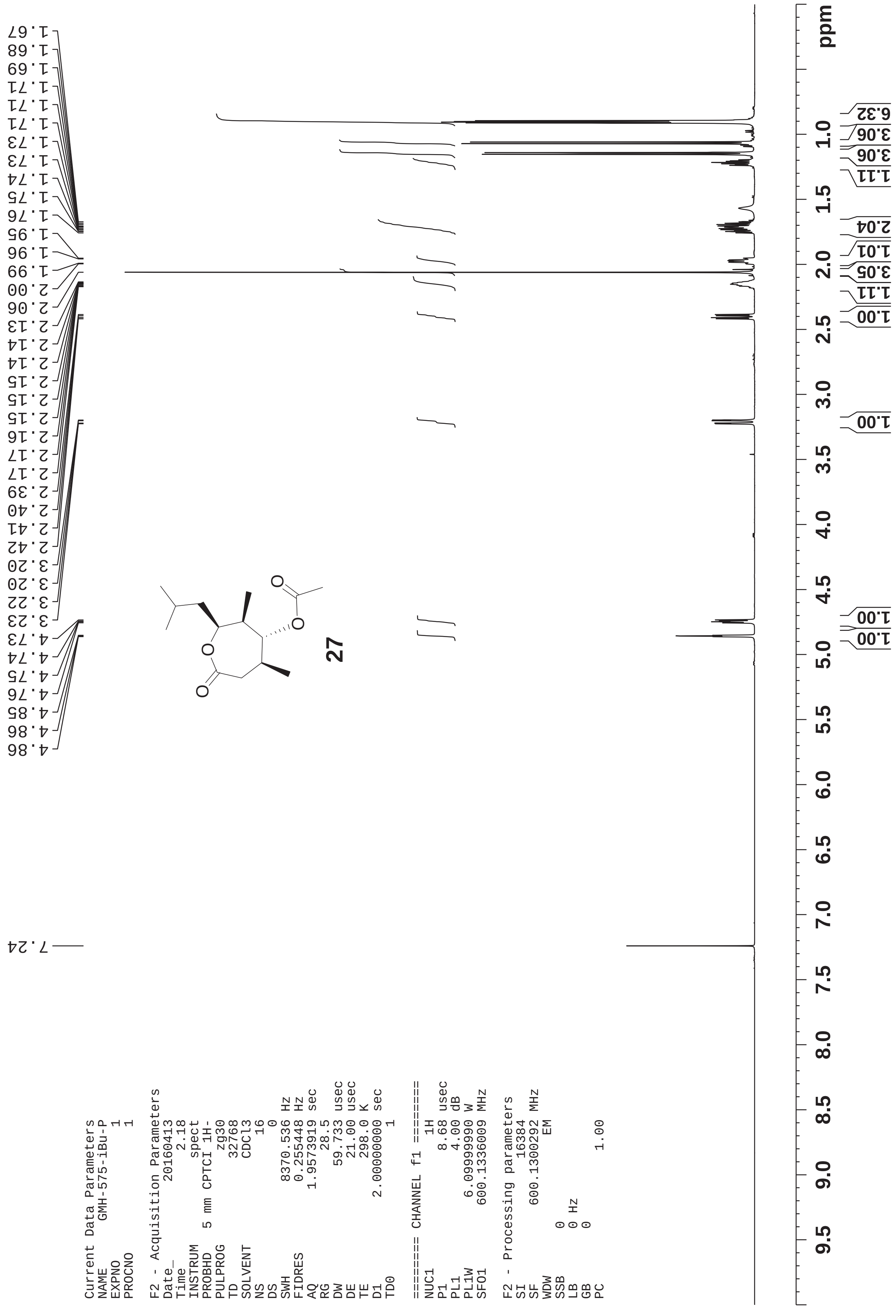

Supplementary Figure 29. ^{1}H NMR spectrum of compound 27.

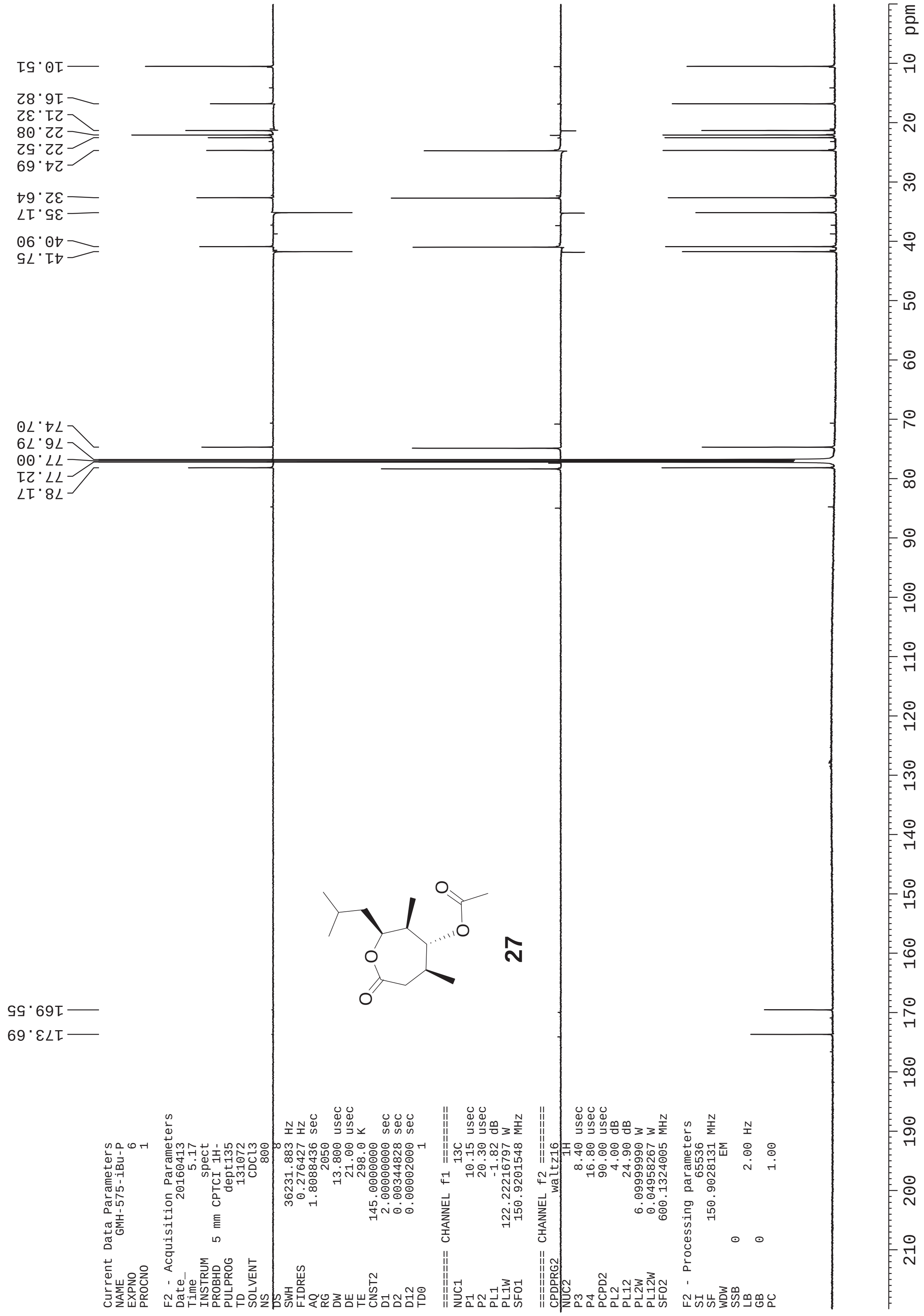

Supplementary Figure 30. ^{13}C and DEPT NMR spectra of compound 27.

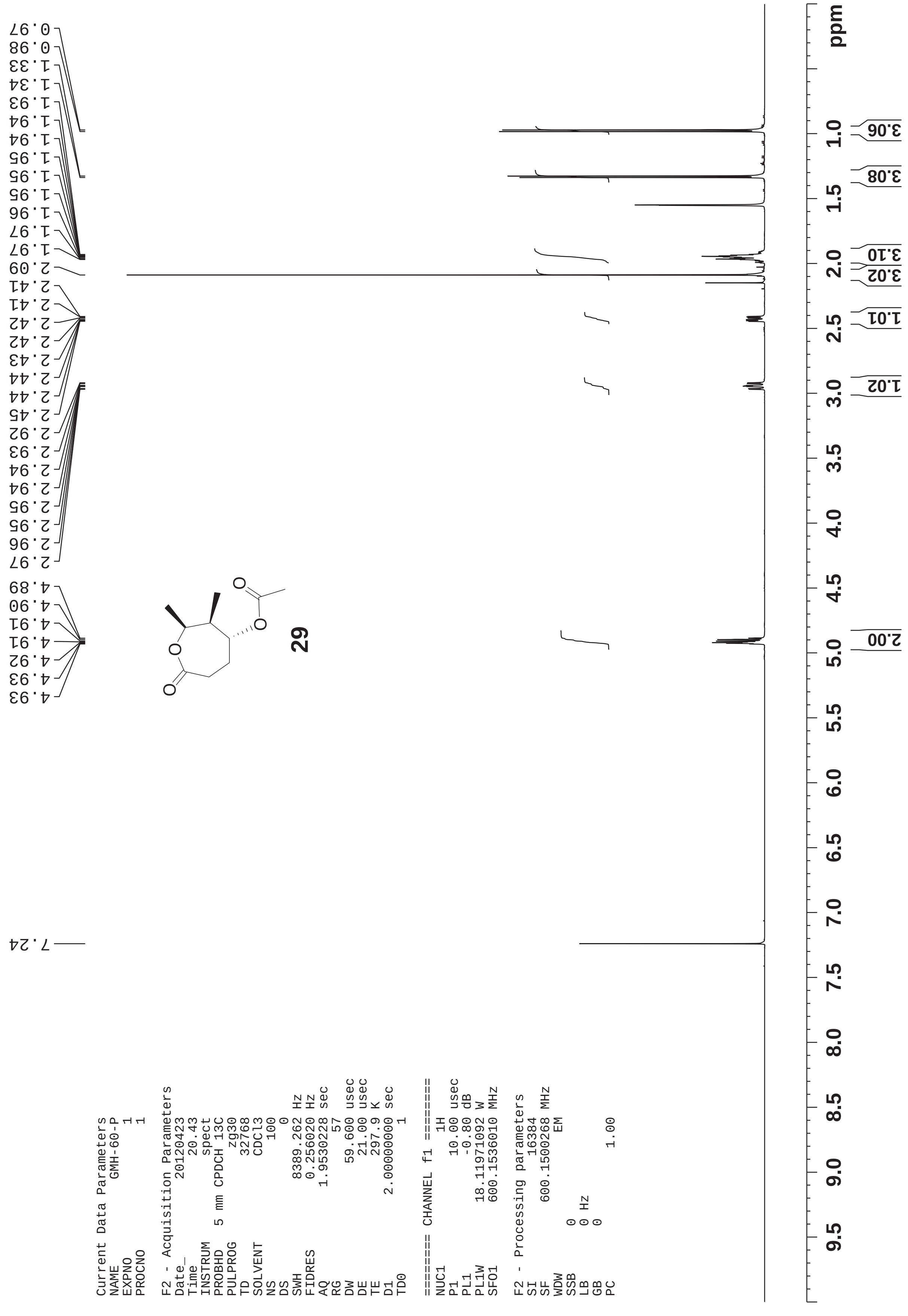

Supplementary Figure 31. ^{1}H NMR spectrum of compound 29.

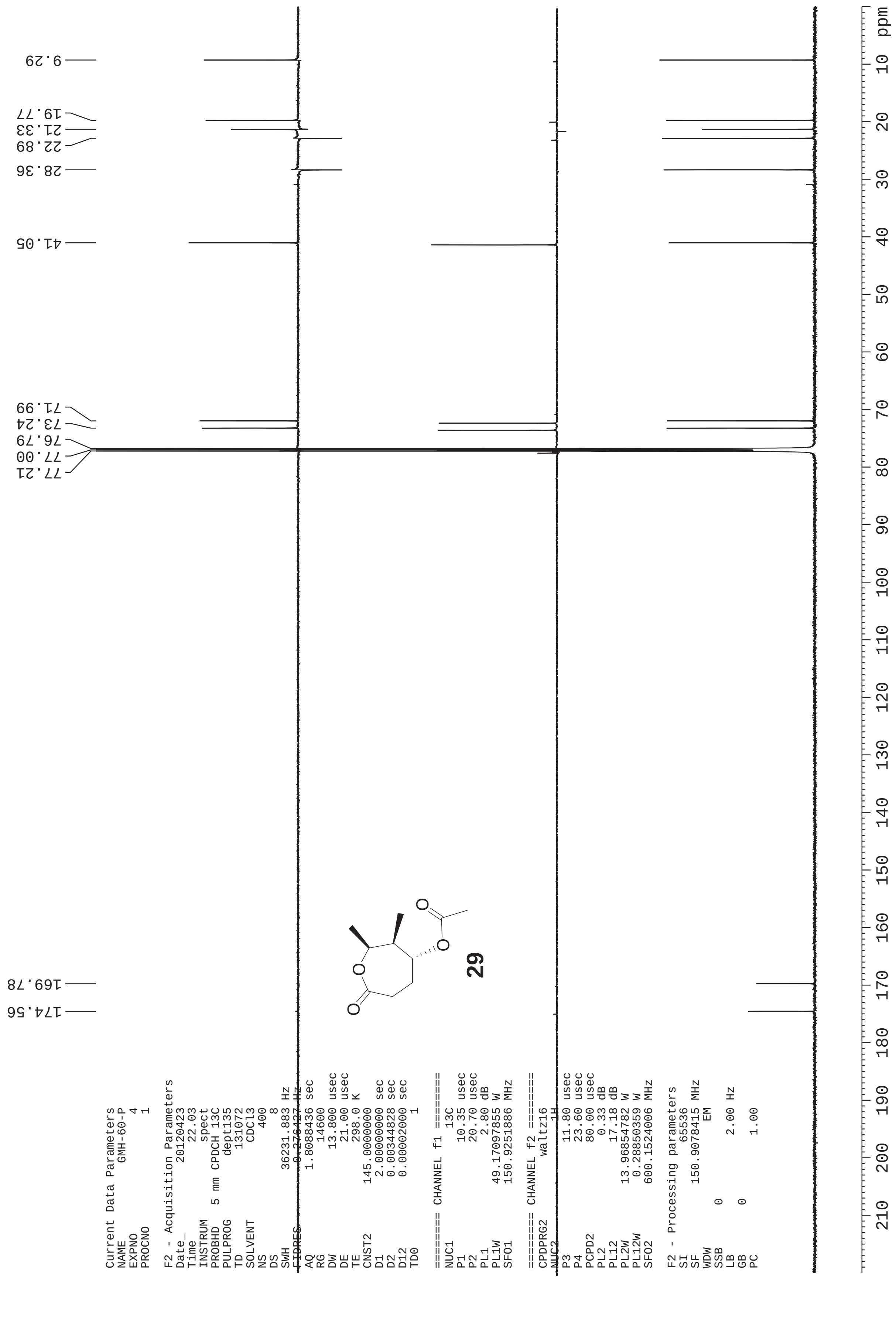

Supplementary Figure 32. ^{13}C and DEPT NMR spectra of compound 29.

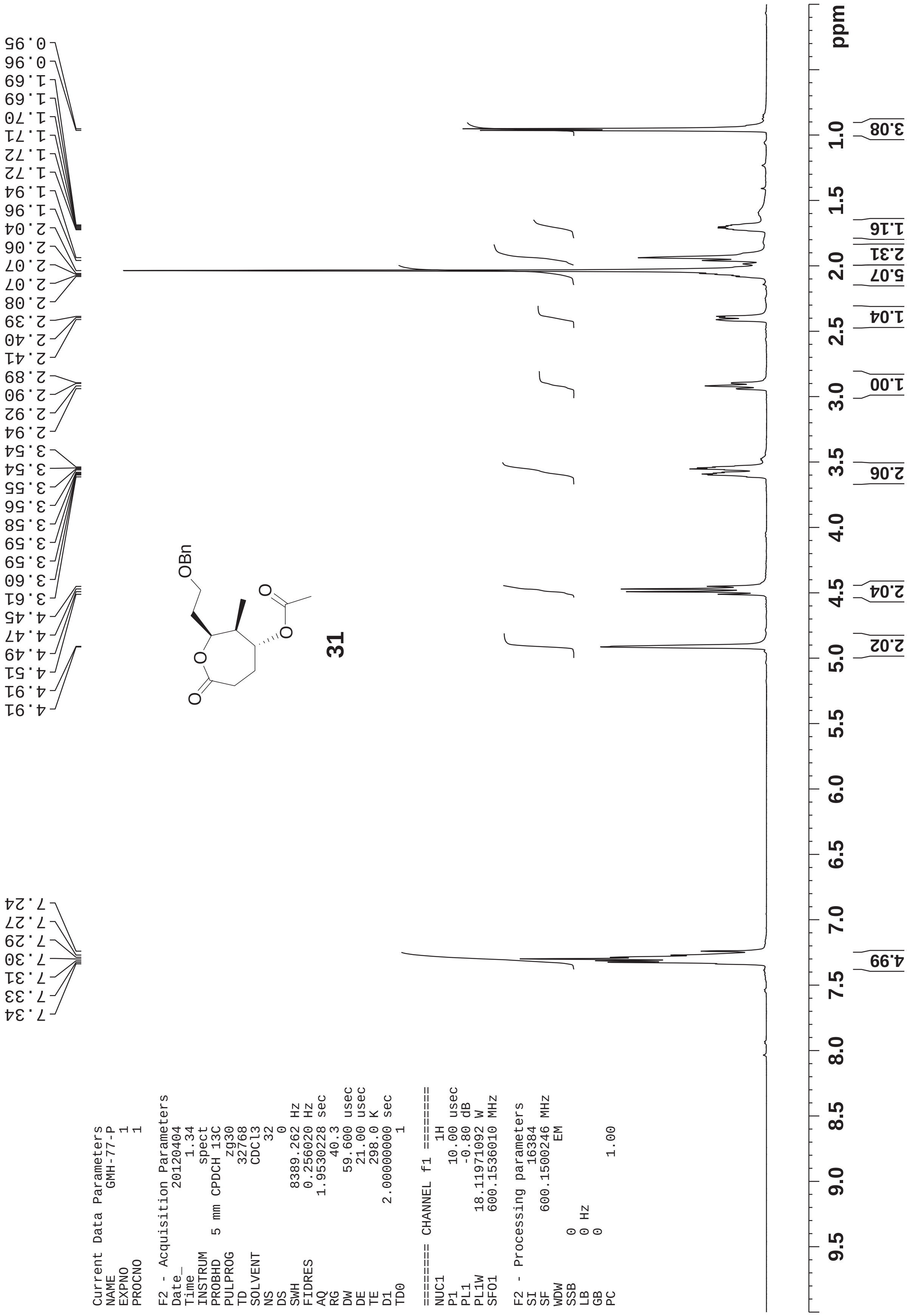

Supplementary Figure 33. ^{1}H NMR spectrum of compound 31.

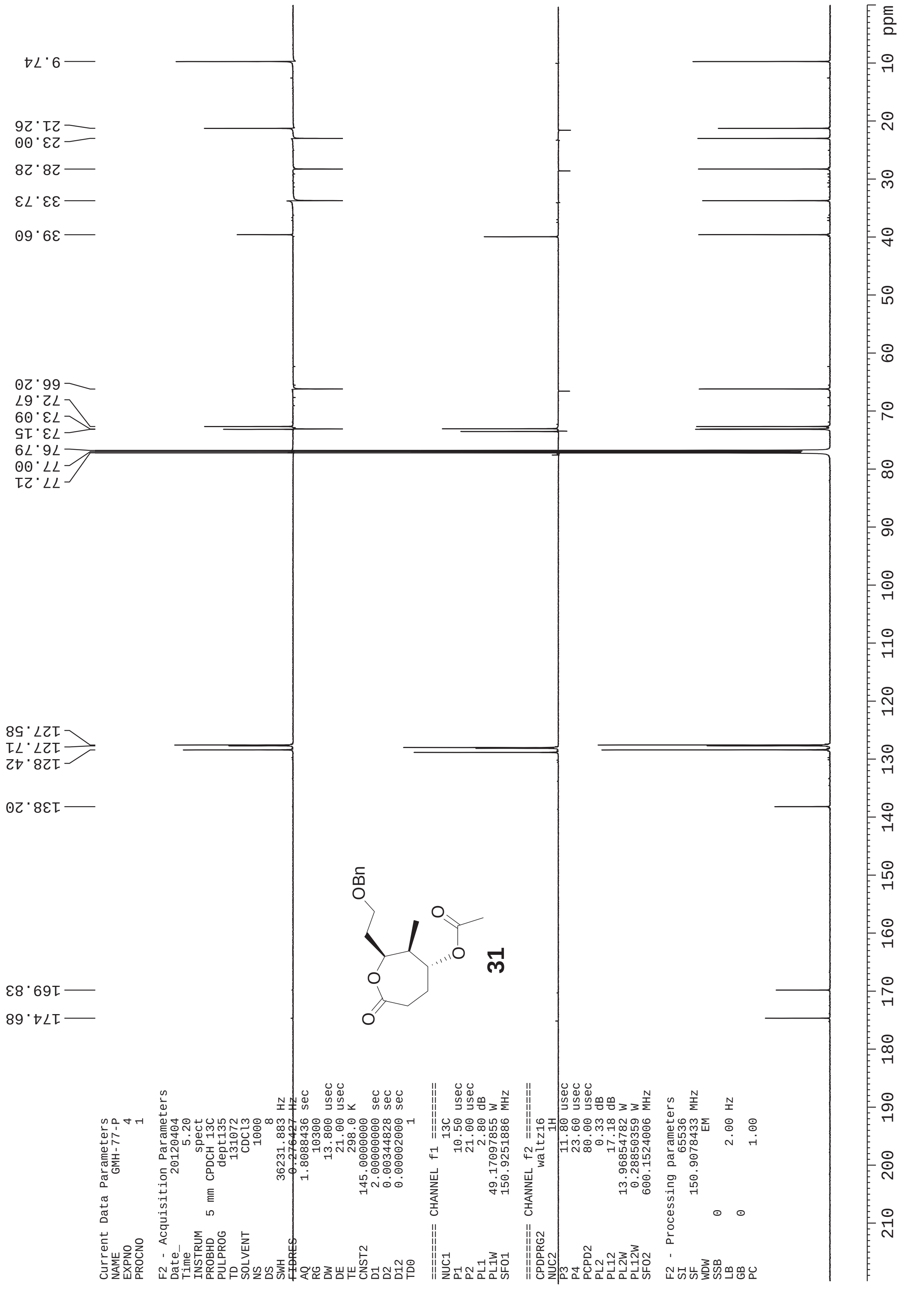

Supplementary Figure 34. ^{13}C and DEPT NMR spectra of compound 31.

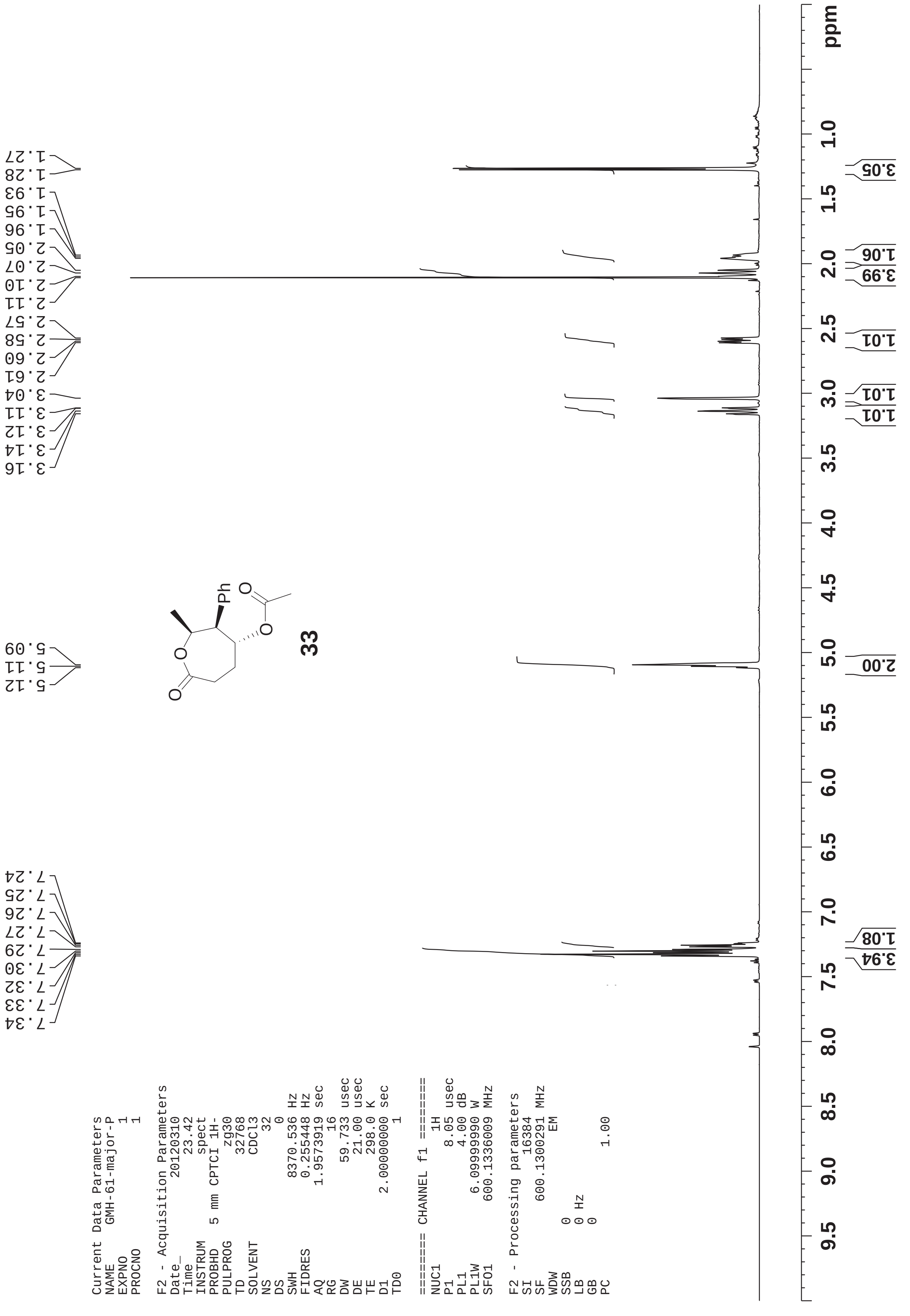

Supplementary Figure 35. ^{1}H NMR spectrum of compound 33.

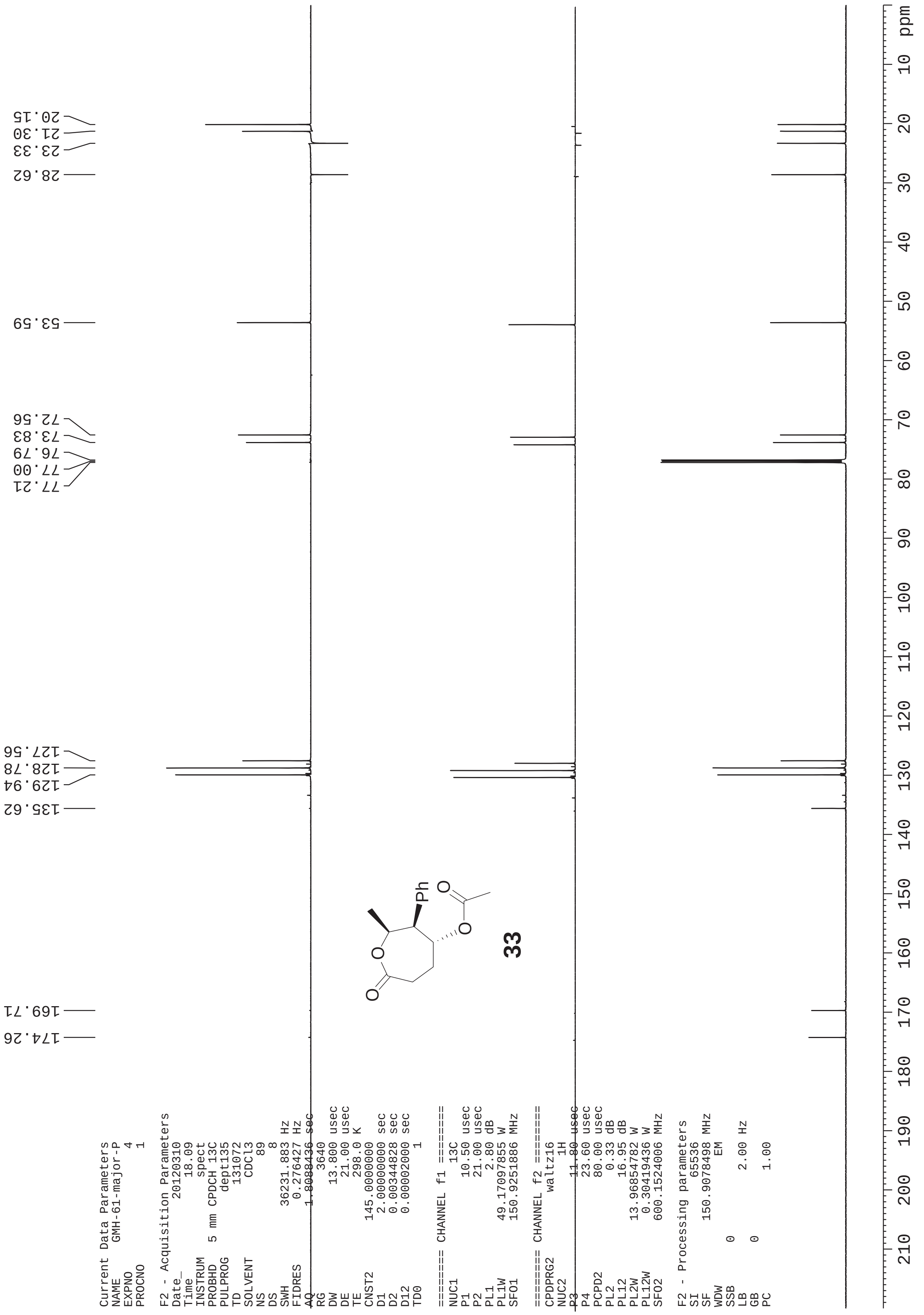

Supplementary Figure 36. ^{13}C and DEPT NMR spectra of compound 33.

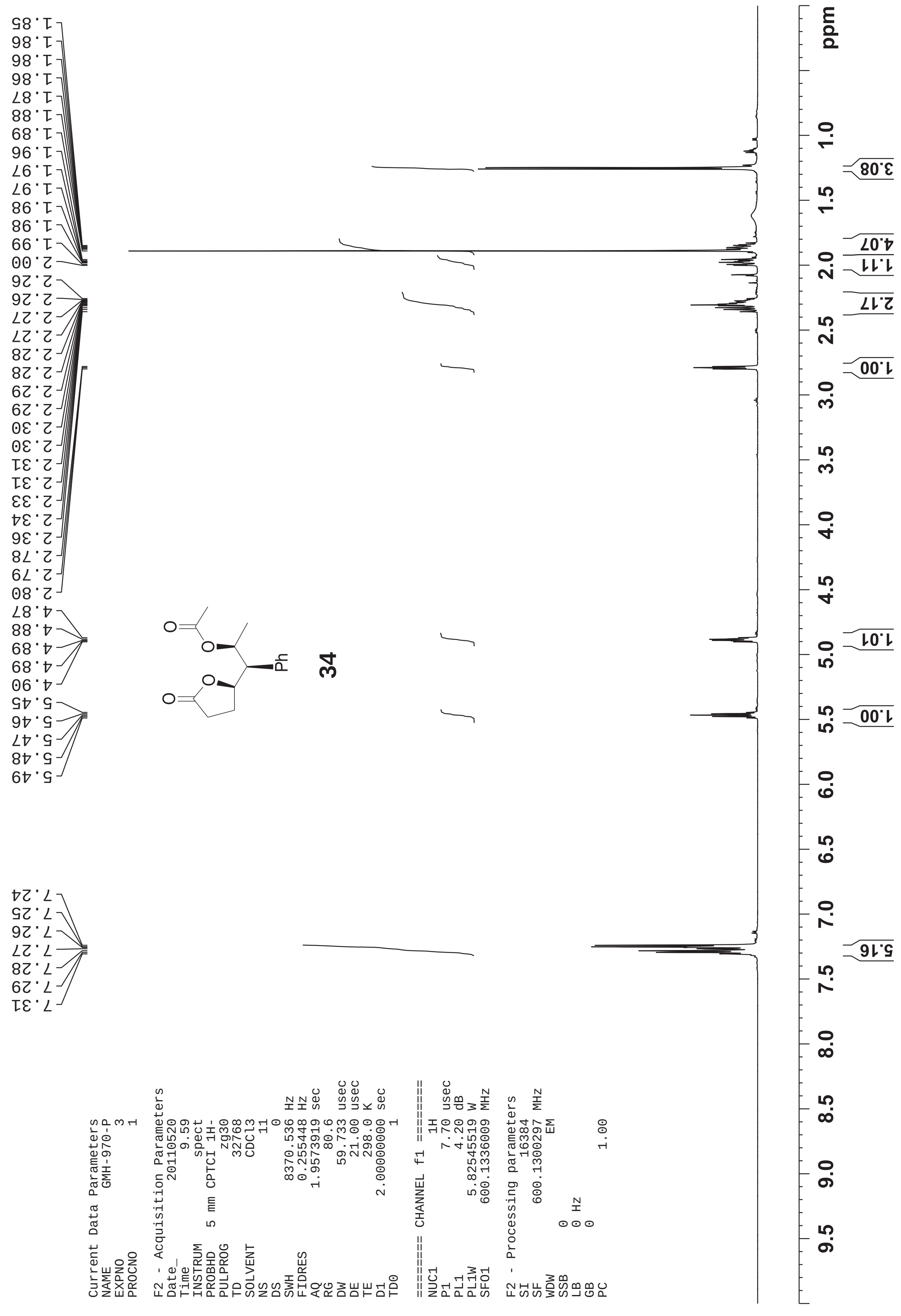

Supplementary Figure 37. ^{1}H NMR spectrum of compound 34.

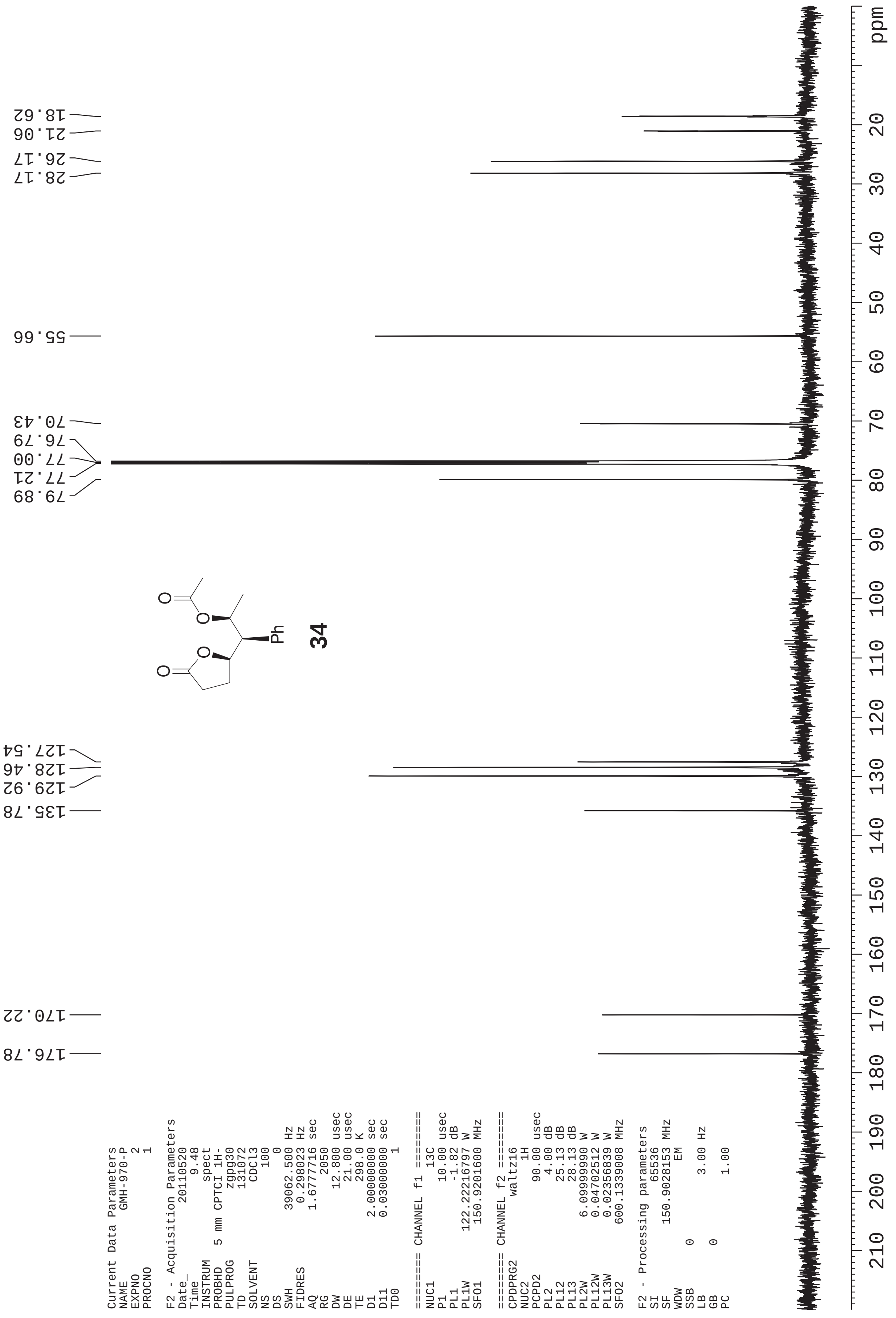

Supplementary Figure 38. ^{13}C NMR spectra of compound 34.

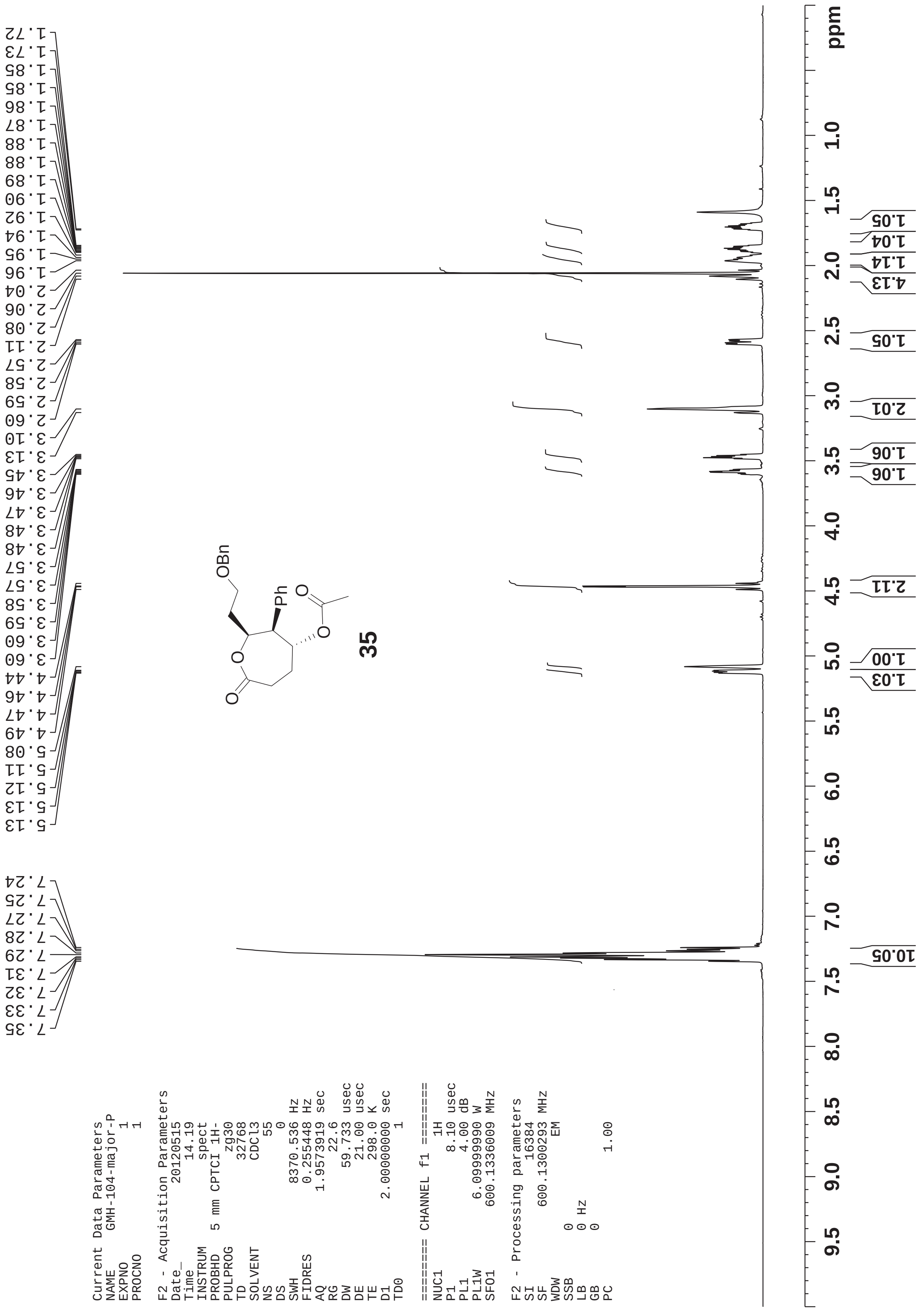

Supplementary Figure 39. ^{1}H NMR spectrum of compound 35.

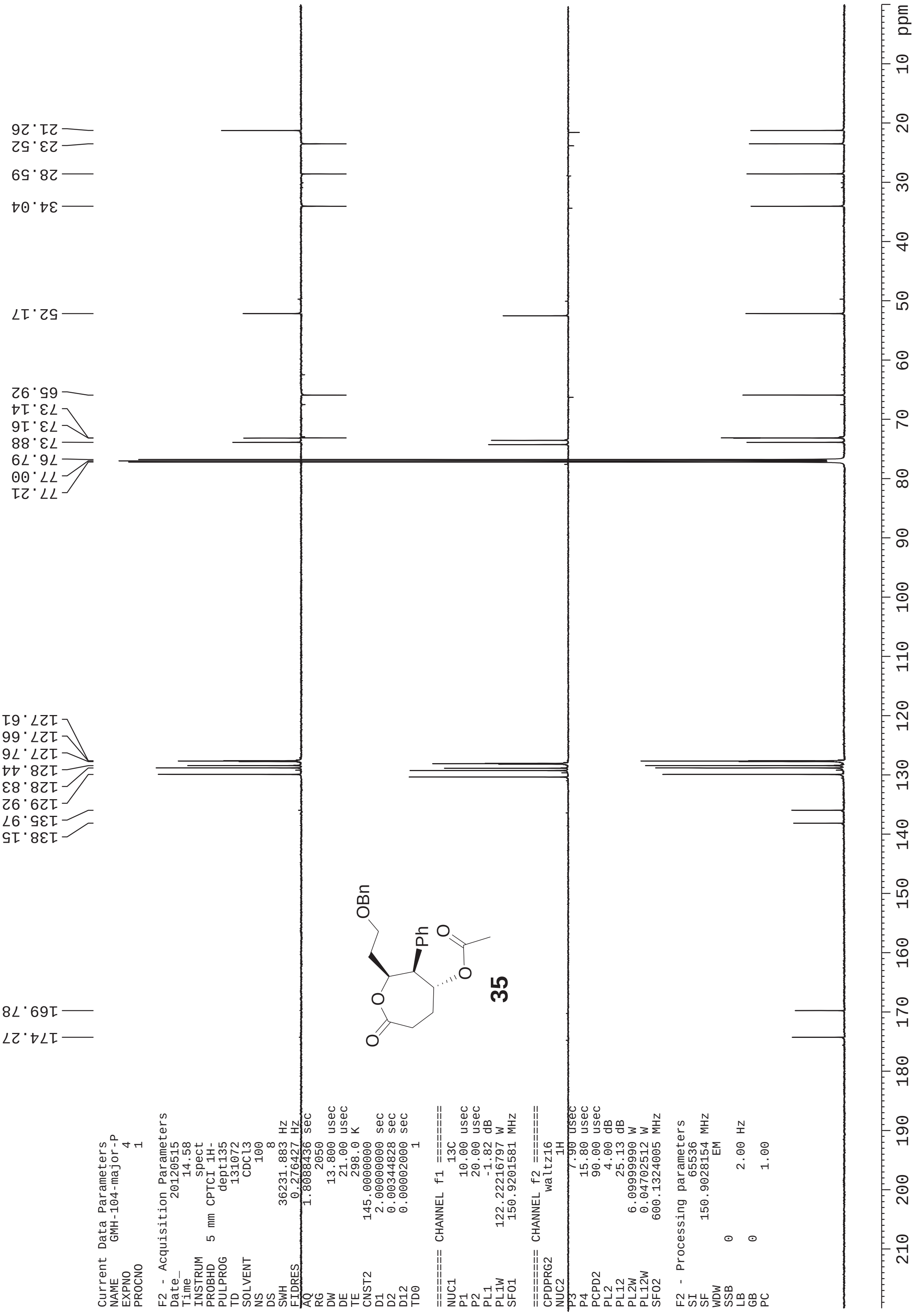

Supplementary Figure 40. ^{13}C and DEPT NMR spectra of compound 35.

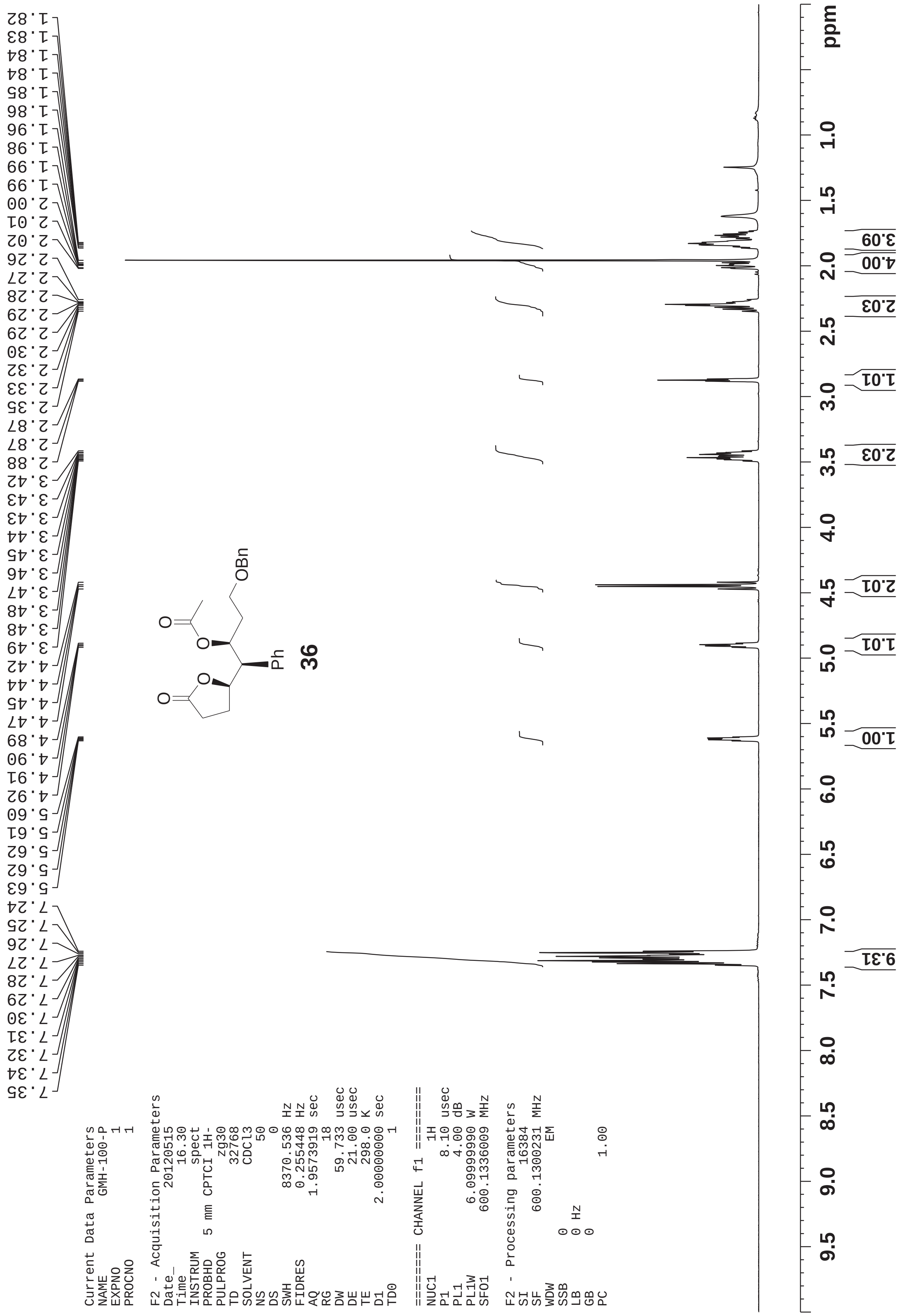

Supplementary Figure 41. ^{1}H NMR spectrum of compound 36.

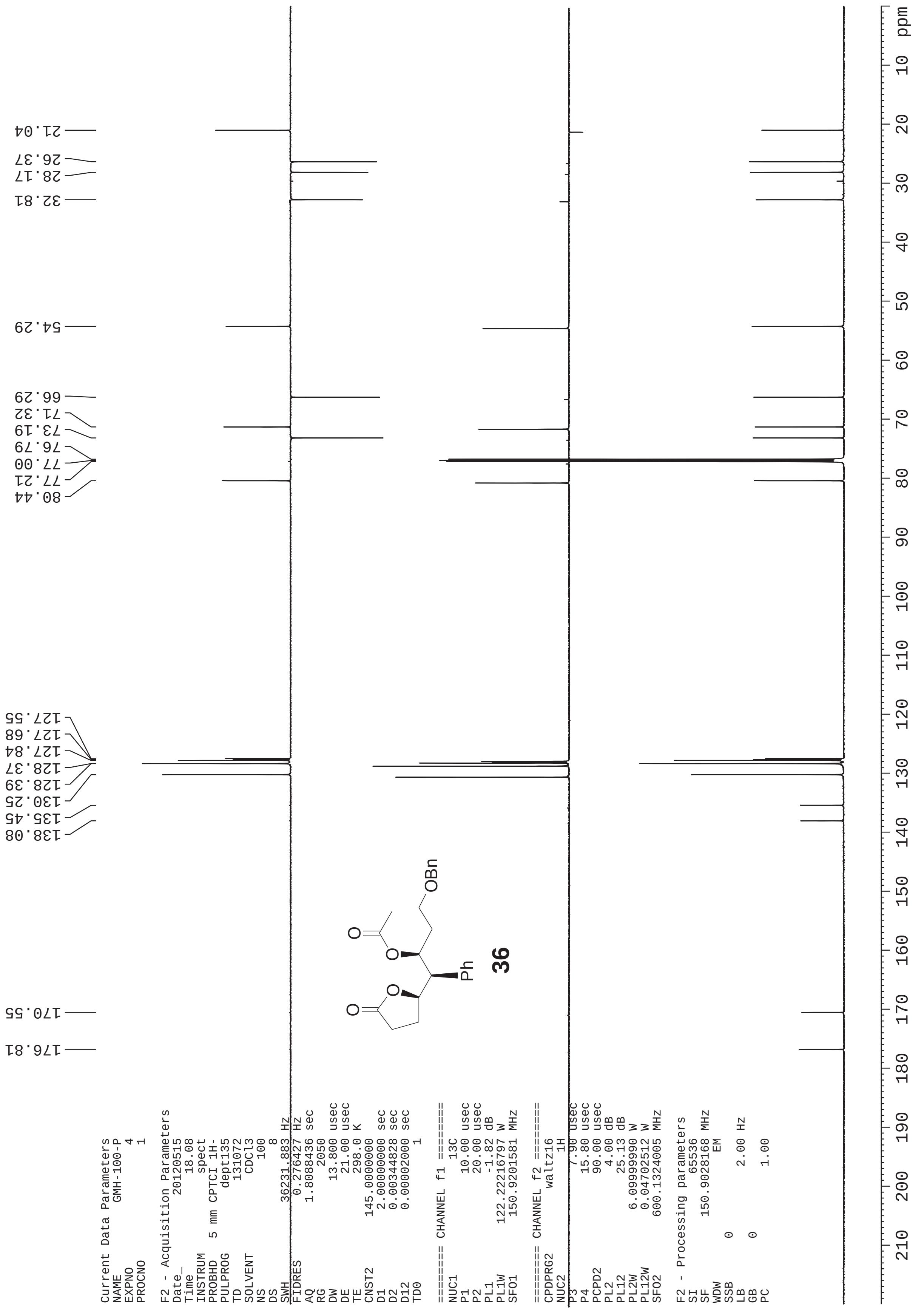

Supplementary Figure 42. ^{13}C and DEPT NMR spectra of compound 36.

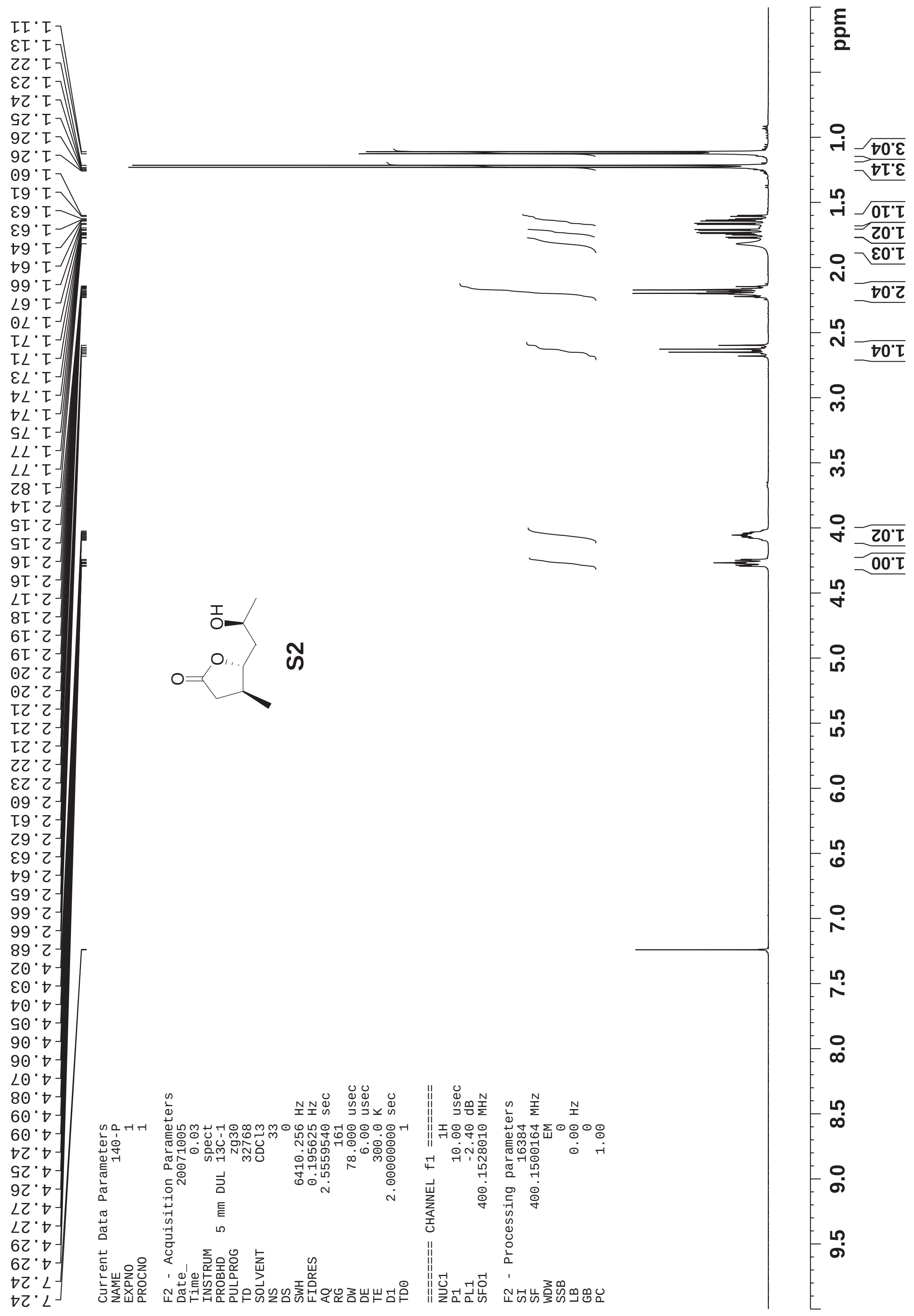

Supplementary Figure 43. ^{1}H NMR spectrum of compound S2.

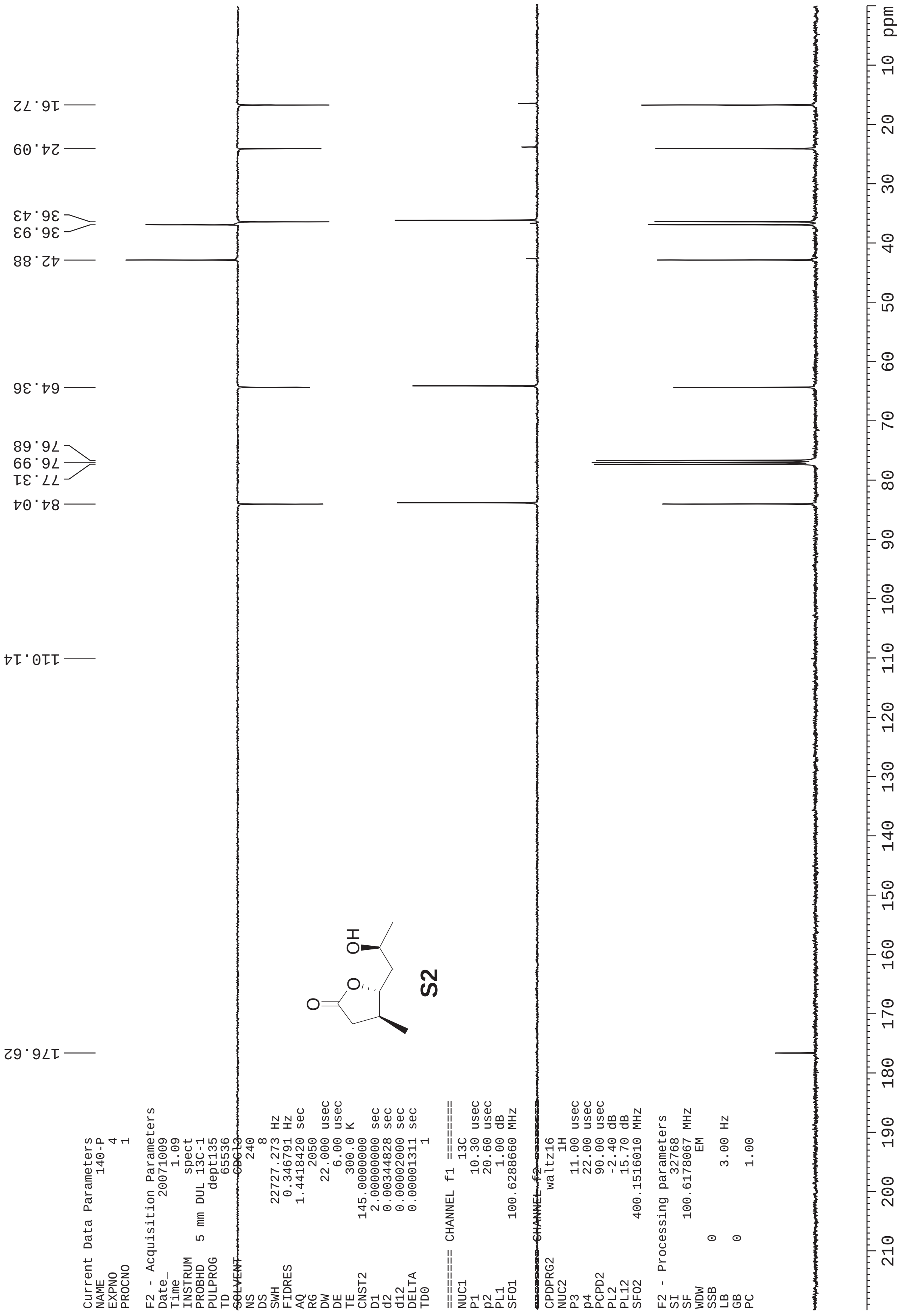

Supplementary Figure 44. ^{13}C and DEPT NMR spectra of compound S2.

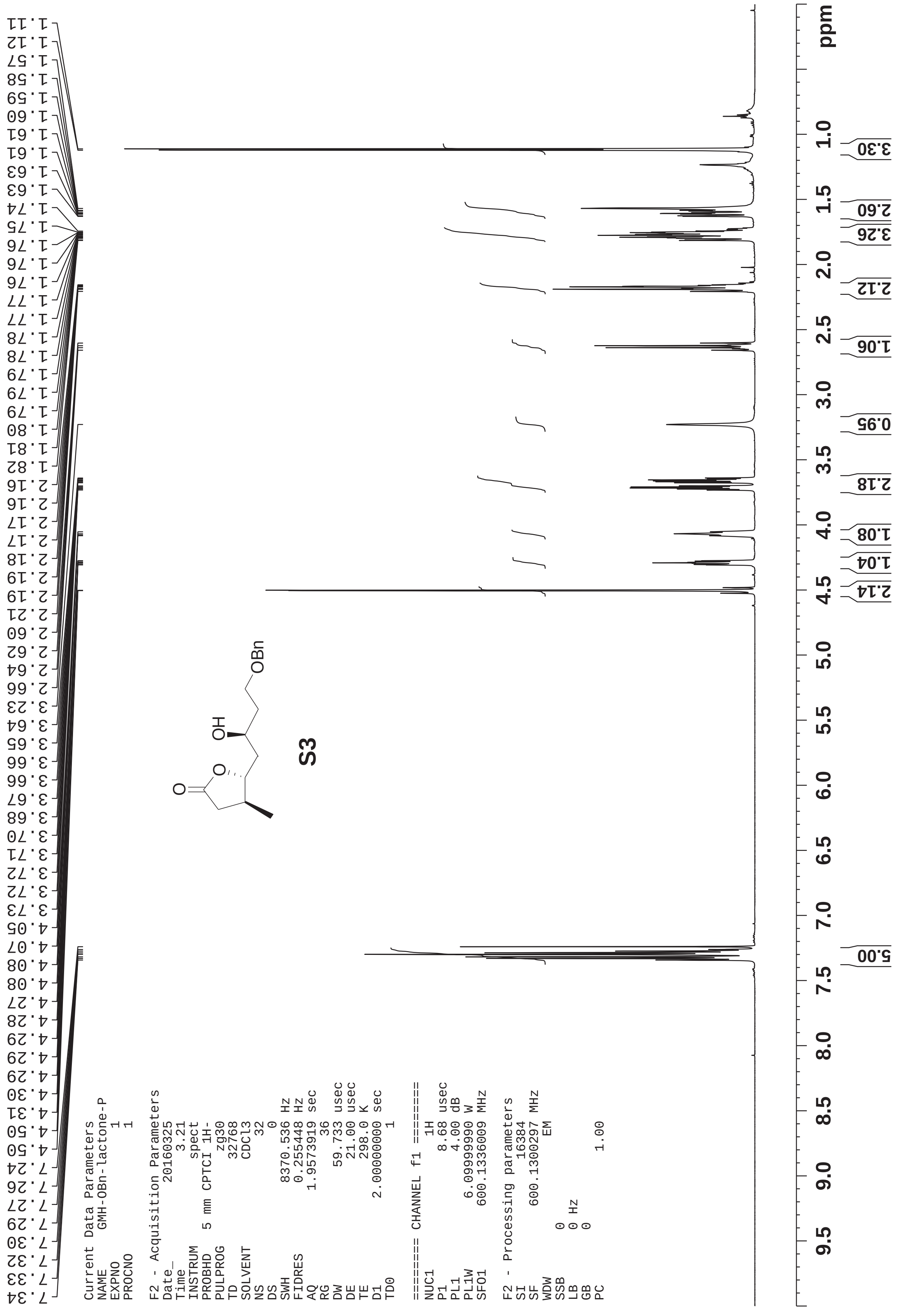

Supplementary Figure 45. ^{1}H NMR spectrum of compound S3.

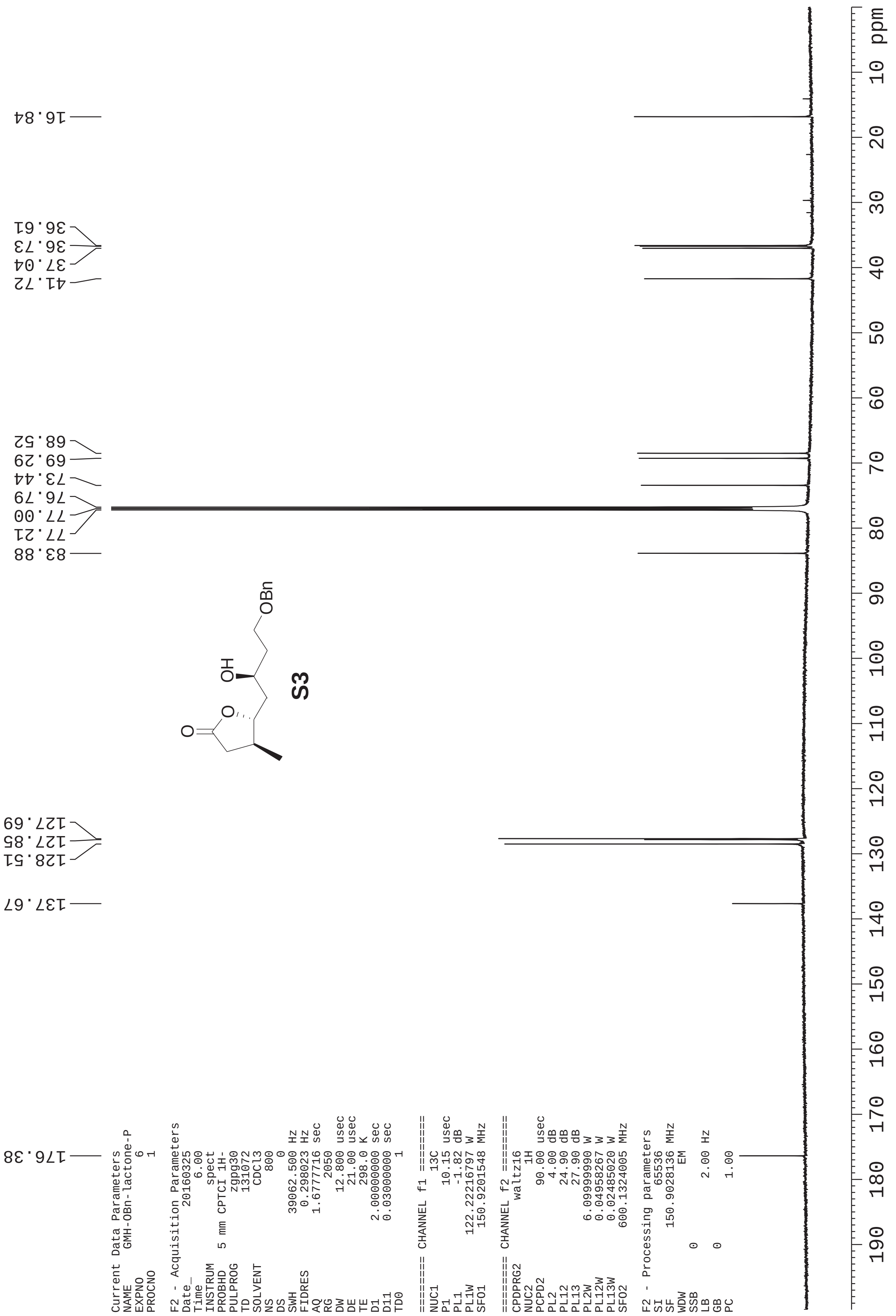

Supplementary Figure 46. ^{13}C NMR spectra of compound S3.

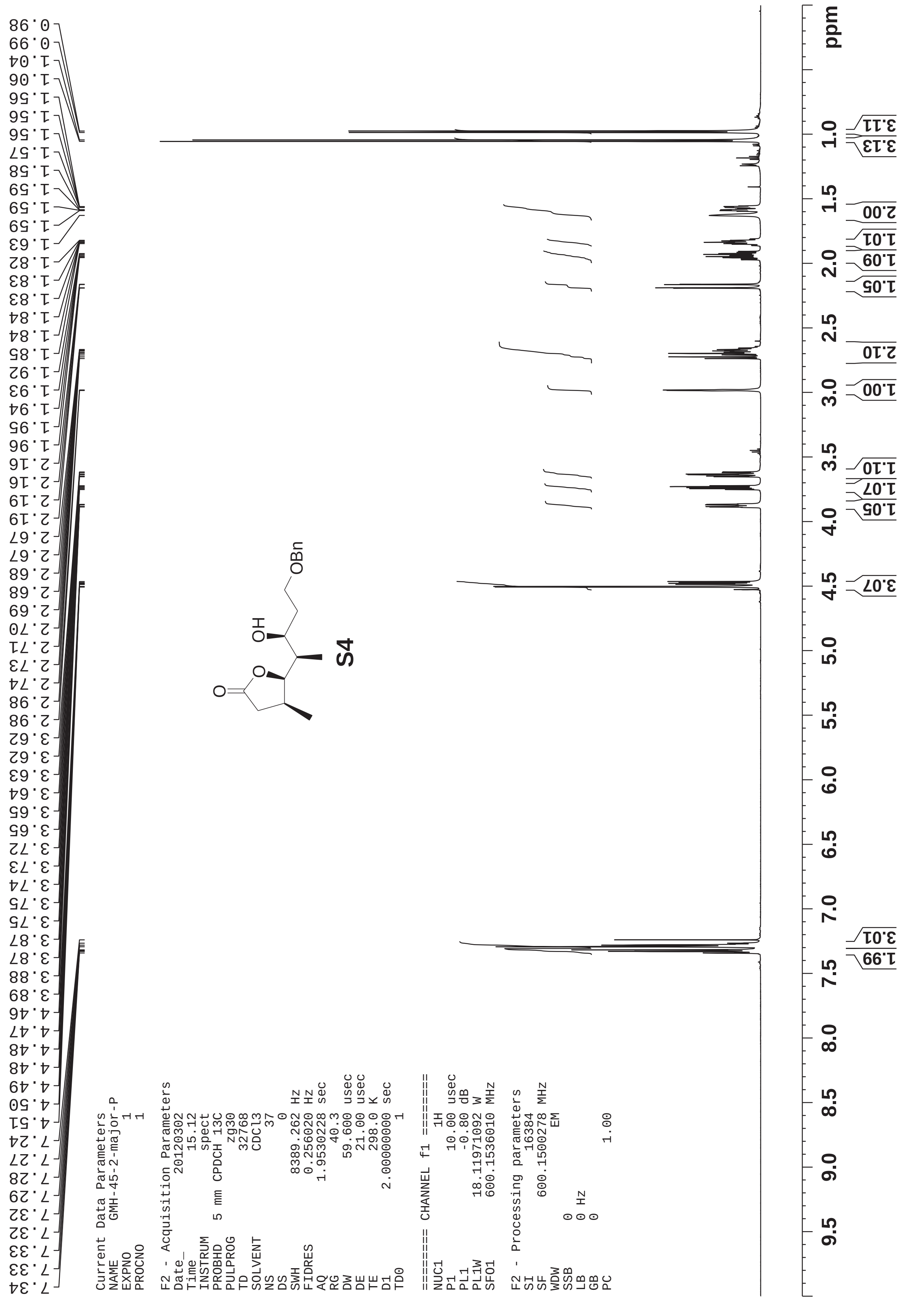

Supplementary Figure 47. ^{1}H NMR spectrum of compound S4.

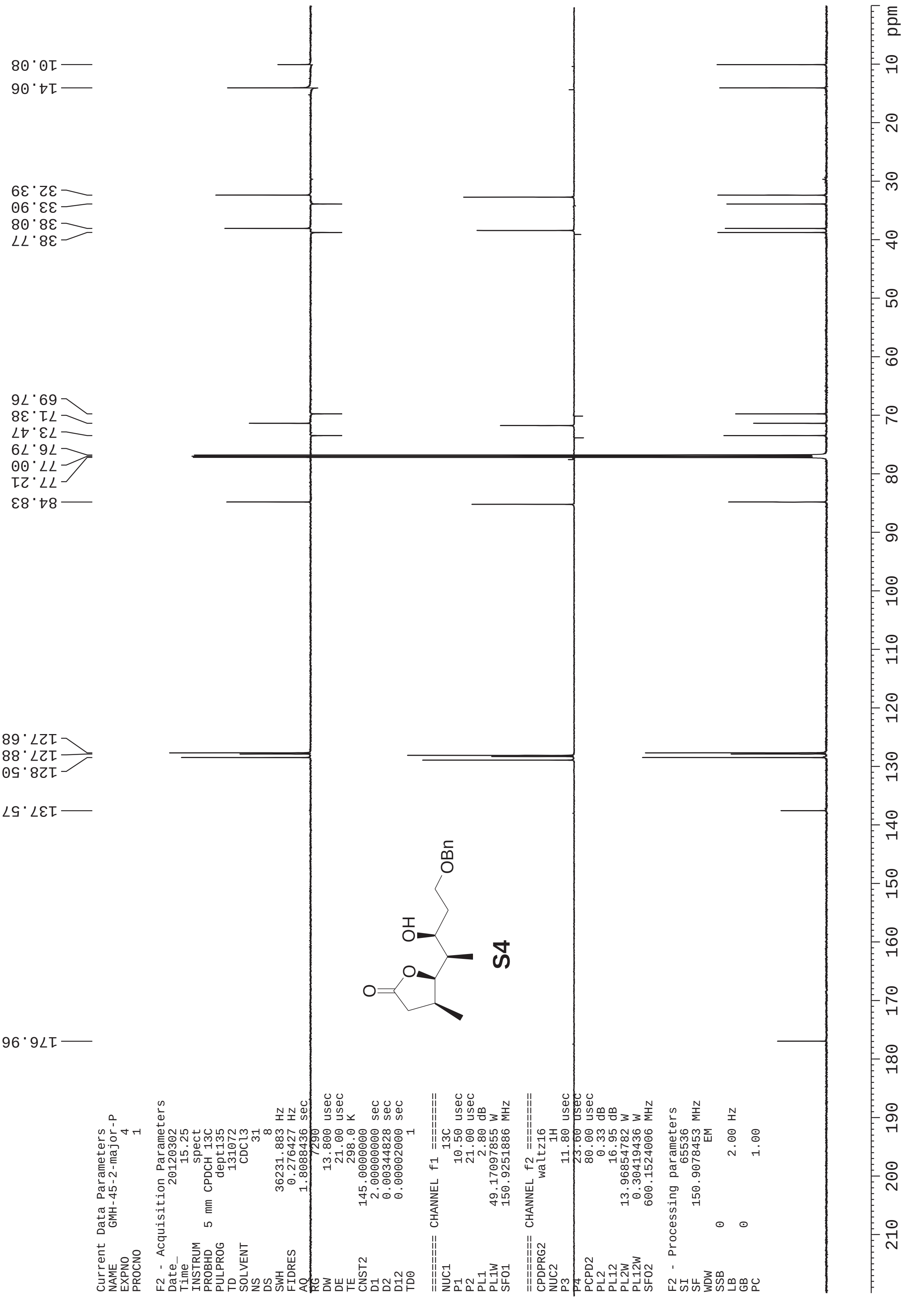

Supplementary Figure 48. ^{13}C and DEPT NMR spectra of compound S4.

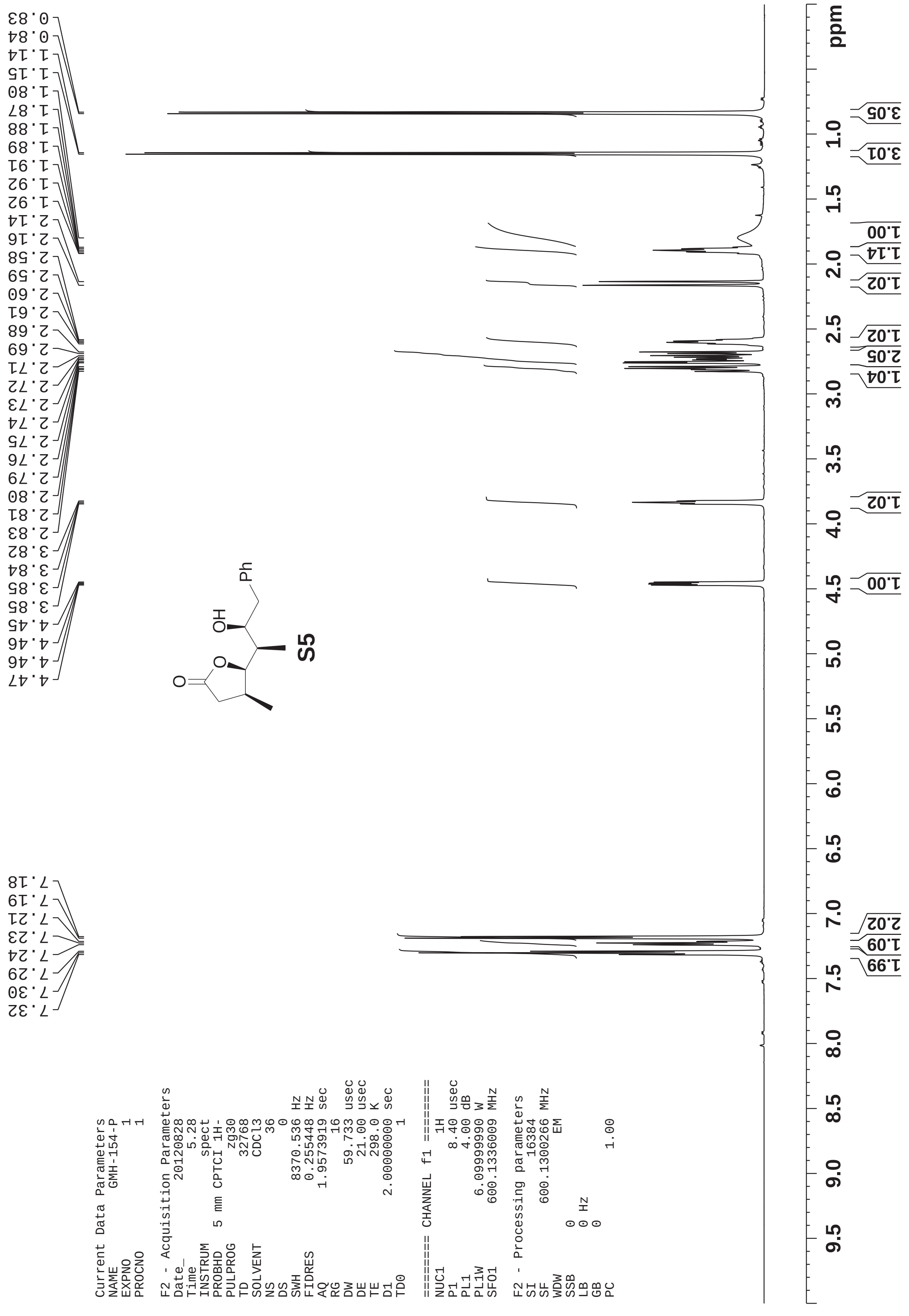

Supplementary Figure 49. ^{1}H NMR spectrum of compound S5.

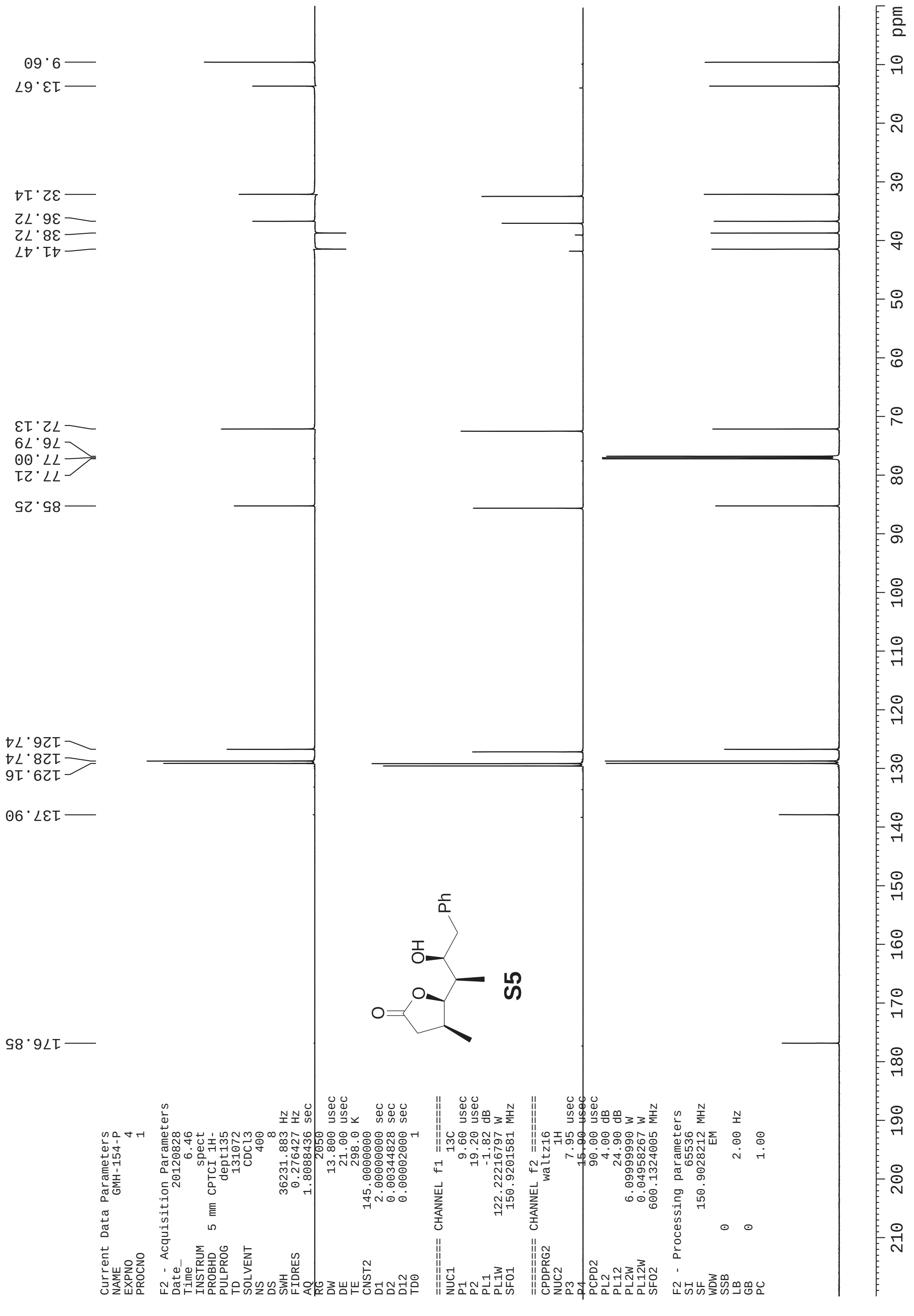

Supplementary Figure 50. ^{13}C and DEPT NMR spectra of compound S5.

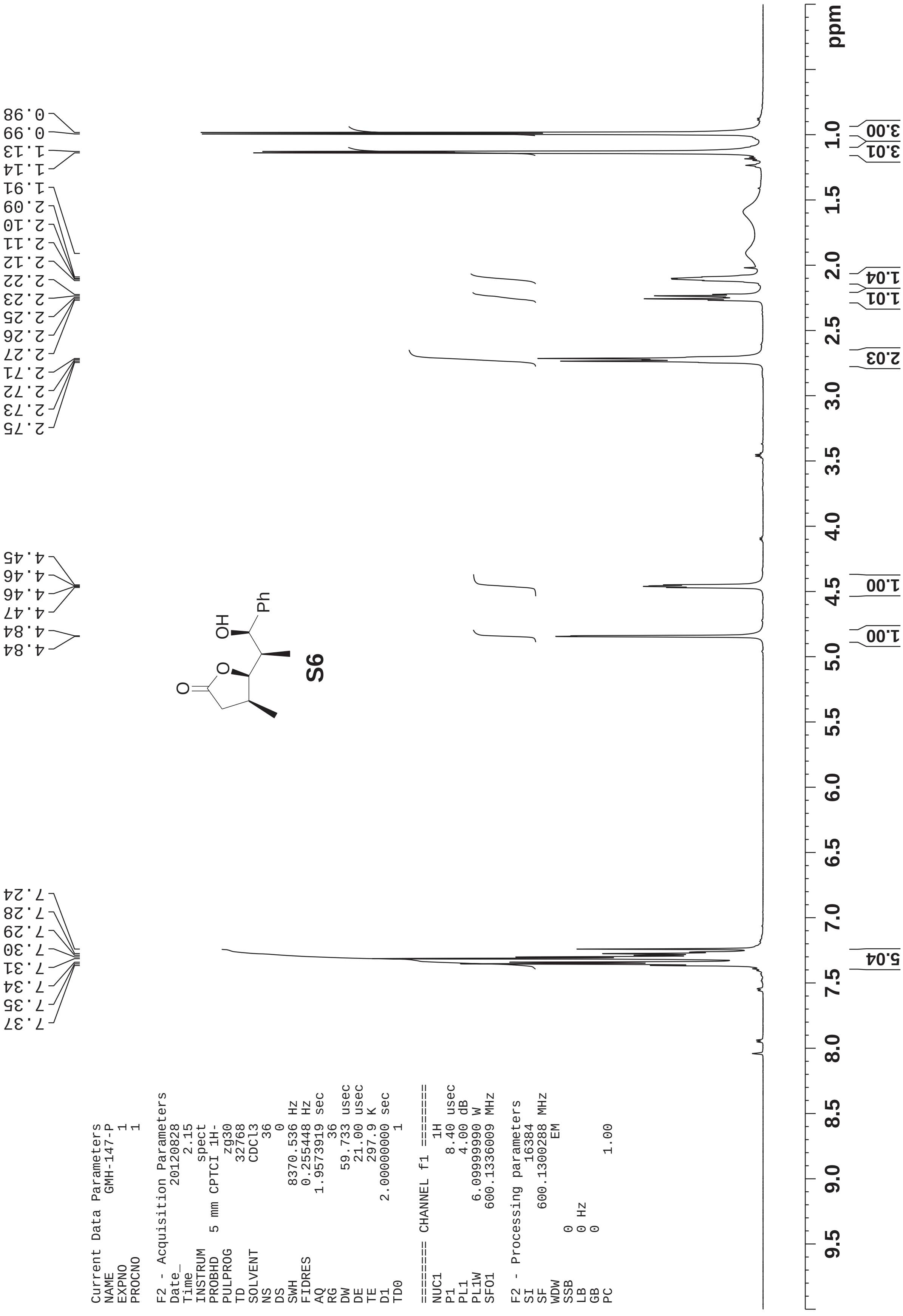

Supplementary Figure 51. ^{1}H NMR spectrum of compound S6.

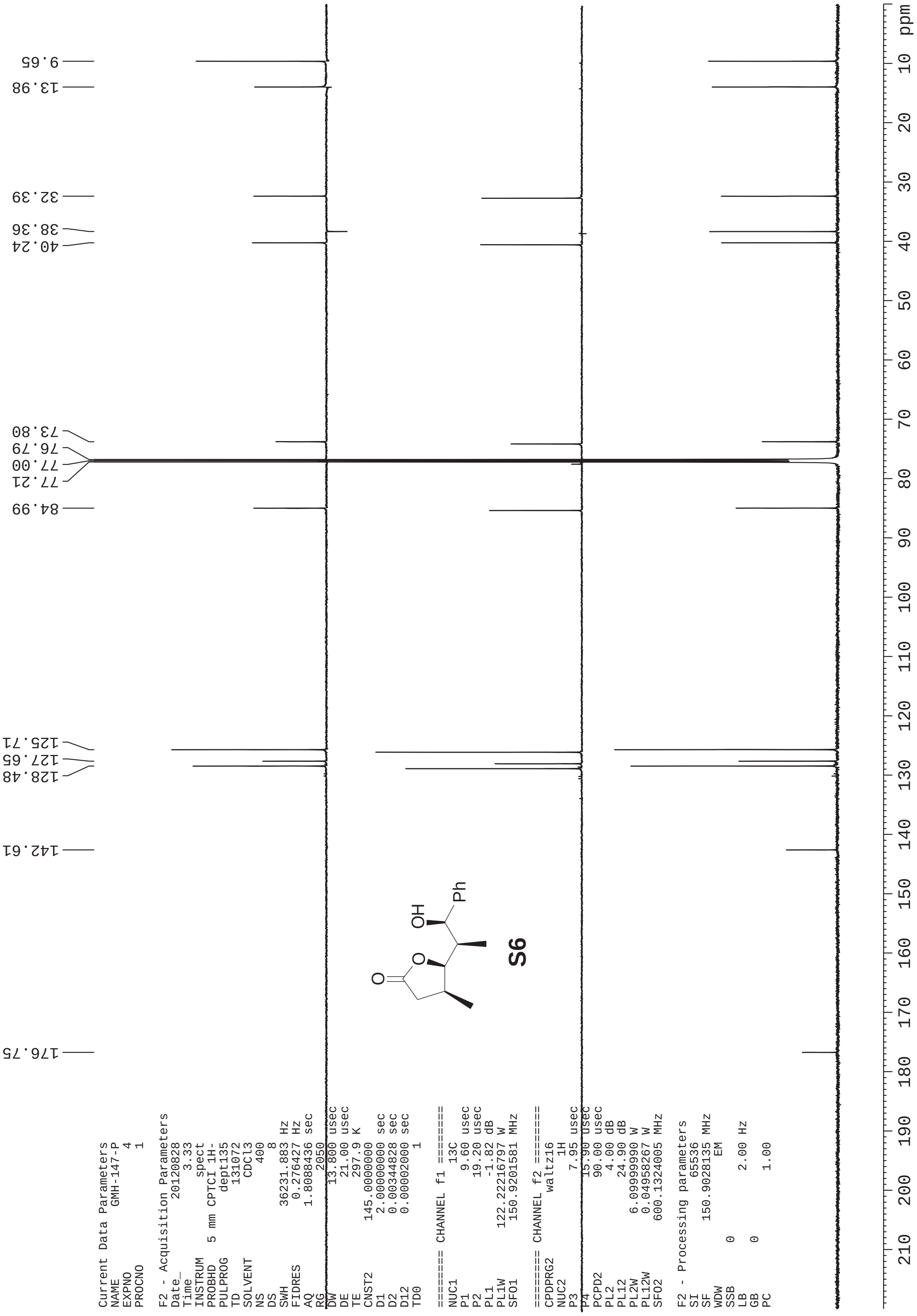

Supplementary Figure 52. ^{13}C and DEPT NMR spectra of compound S6.

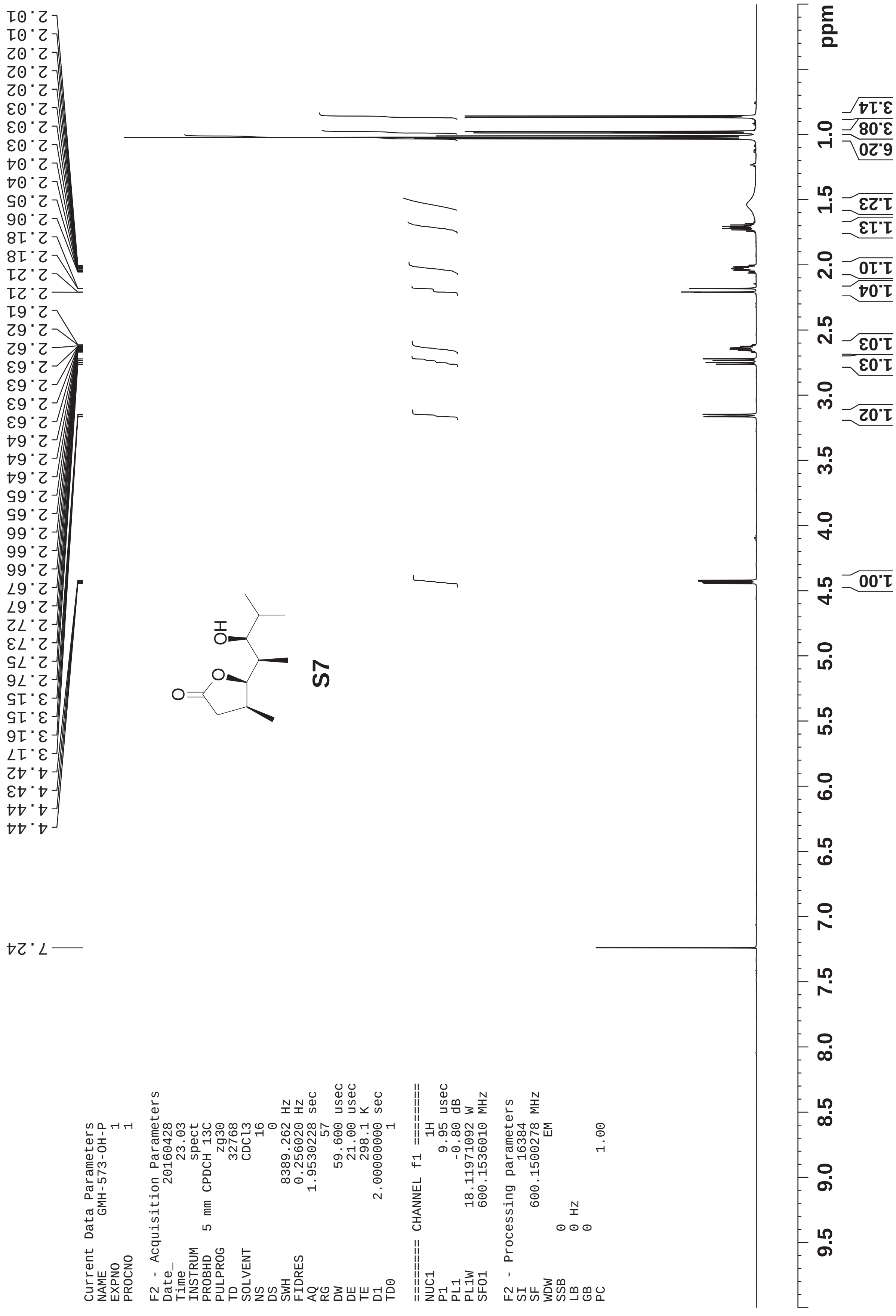

Supplementary Figure 53. ^{1}H NMR spectrum of compound S7.

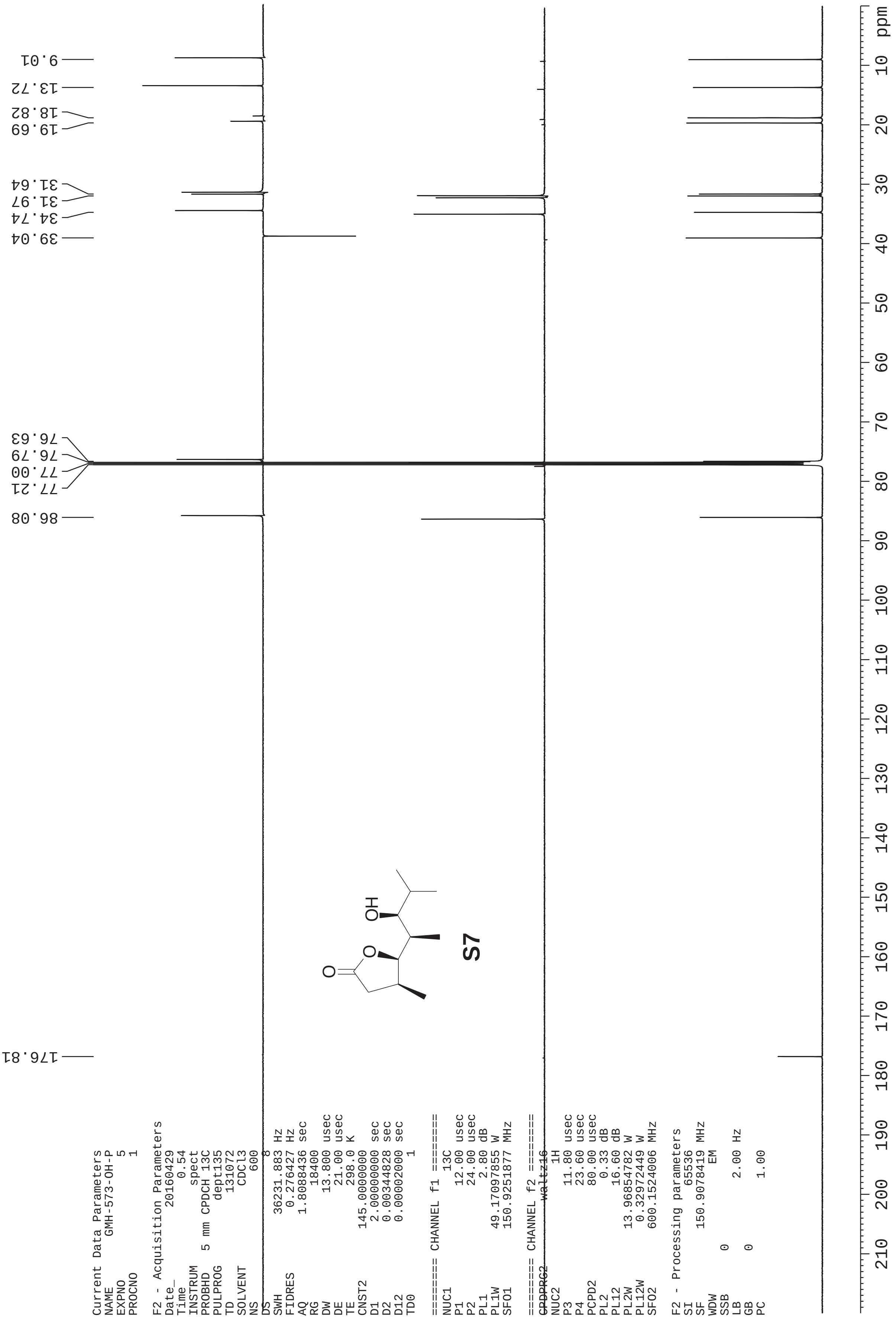

Supplementary Figure 54. ^{13}C and DEPT NMR spectra of compound S7.

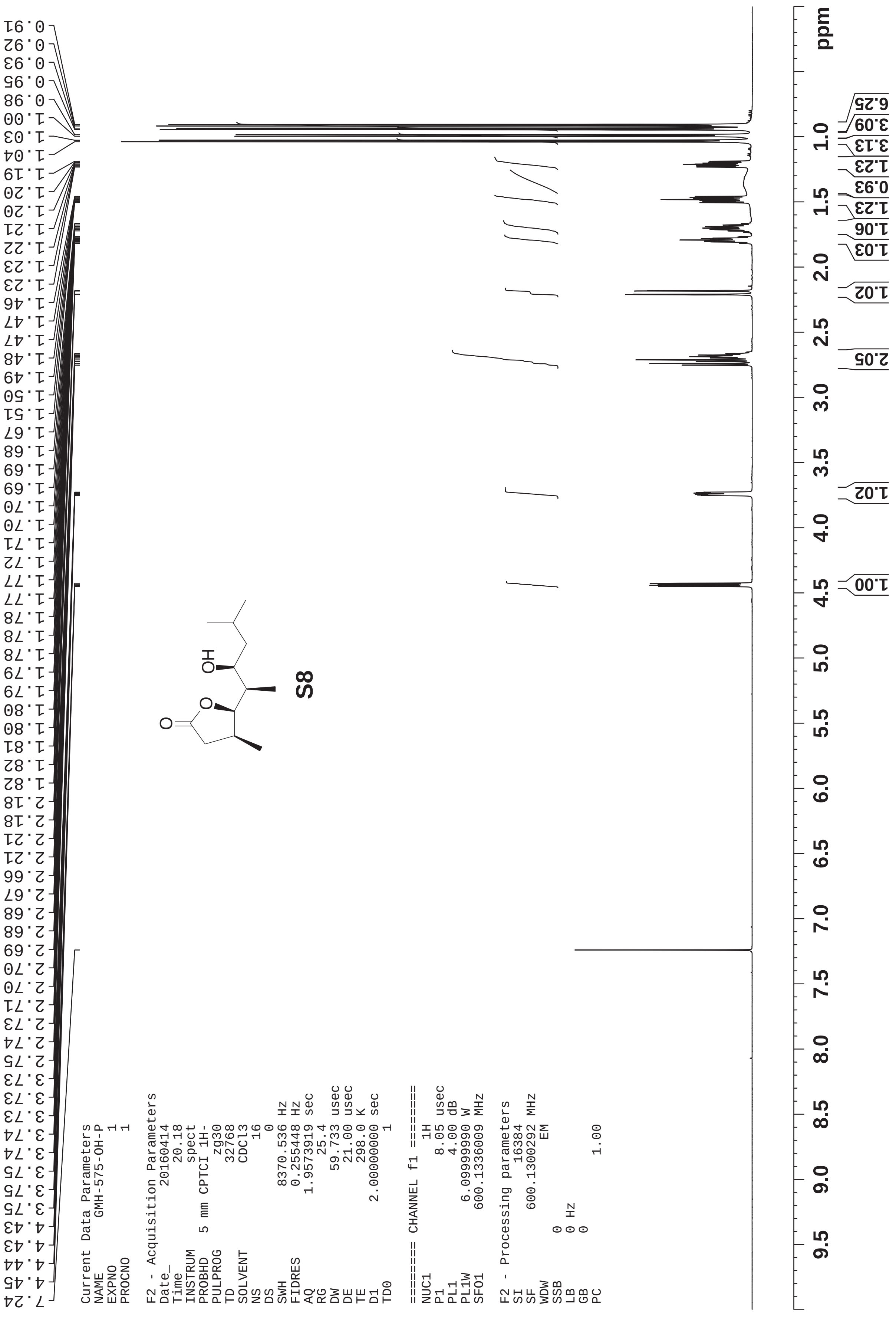

Supplementary Figure 55. ¹H NMR spectrum of compound S8.

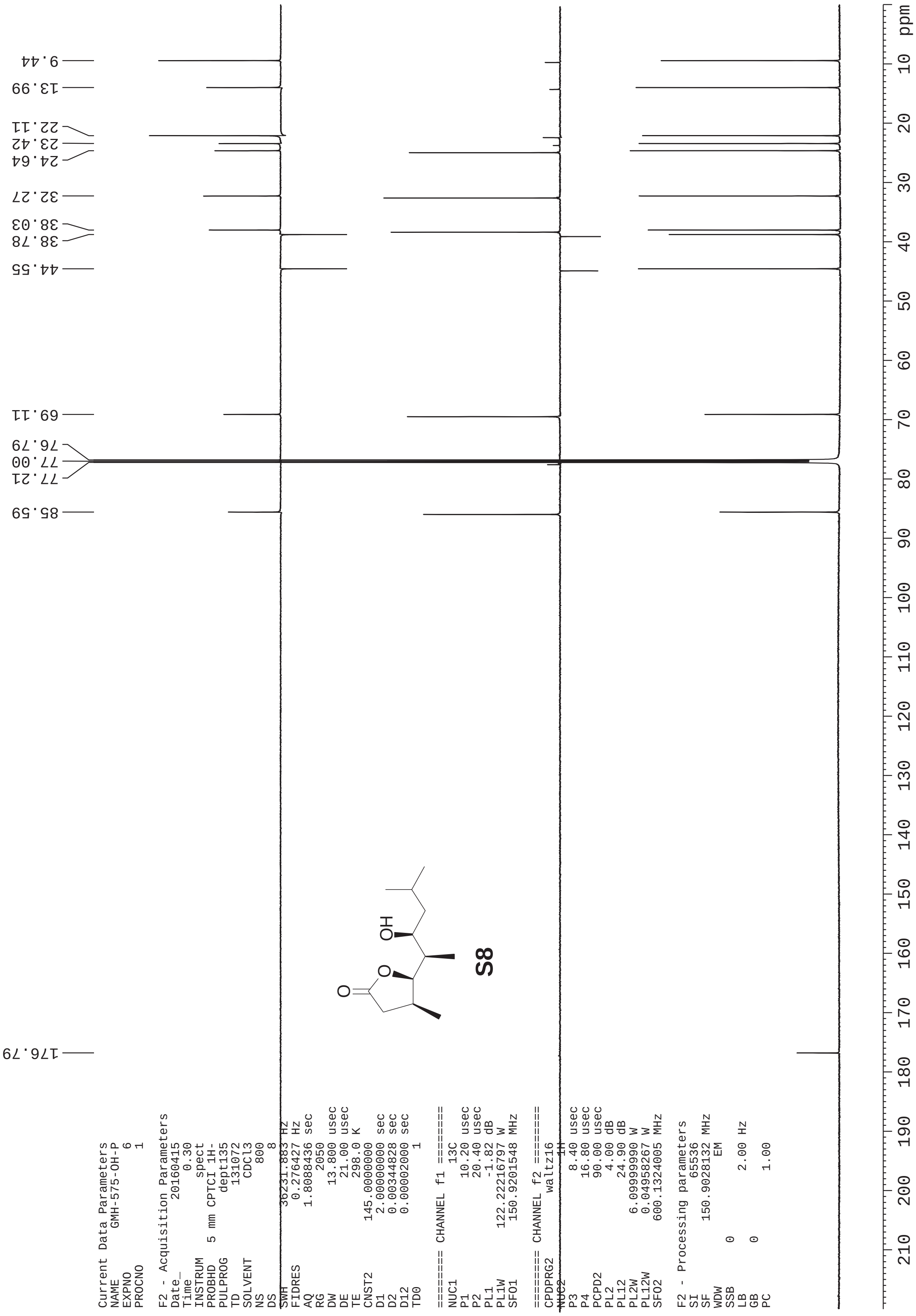

Supplementary Figure 56. ^{13}C and DEPT NMR spectra of compound S8.

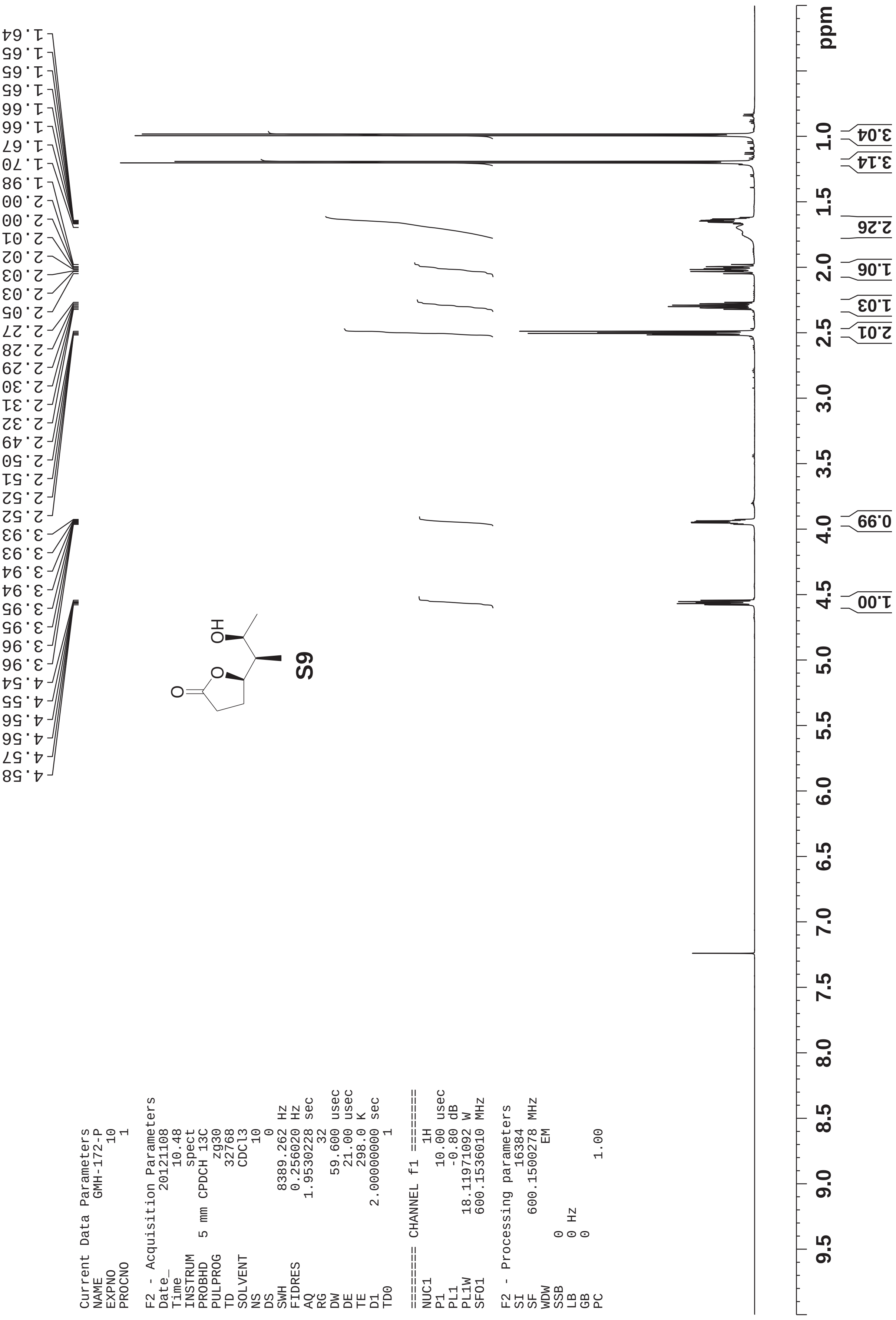

Supplementary Figure 57. ^{1}H NMR spectrum of compound S9.

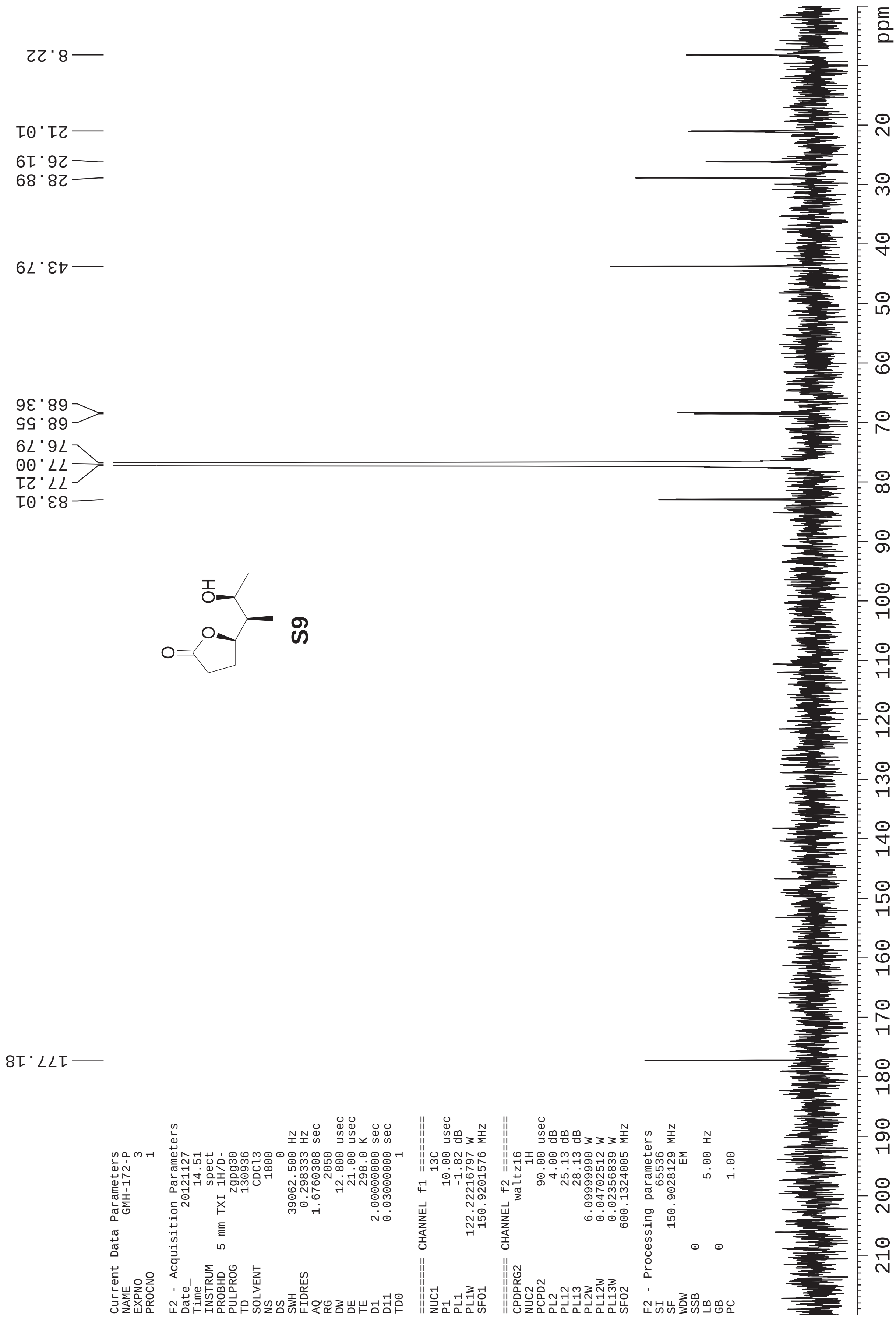

Supplementary Figure 58. ^{13}C NMR spectra of compound S9.

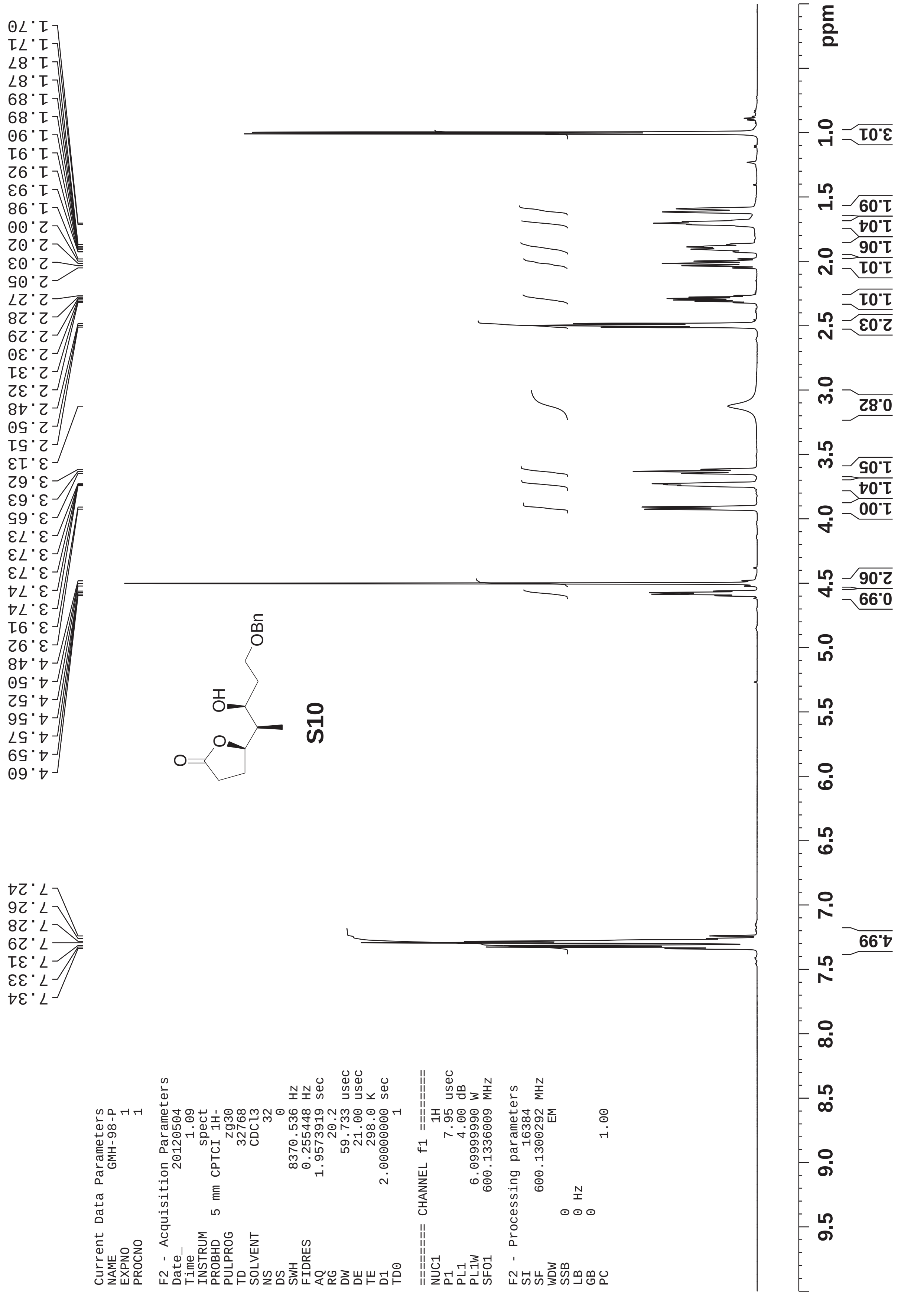

Supplementary Figure 59. ^{1}H NMR spectrum of compound S10.

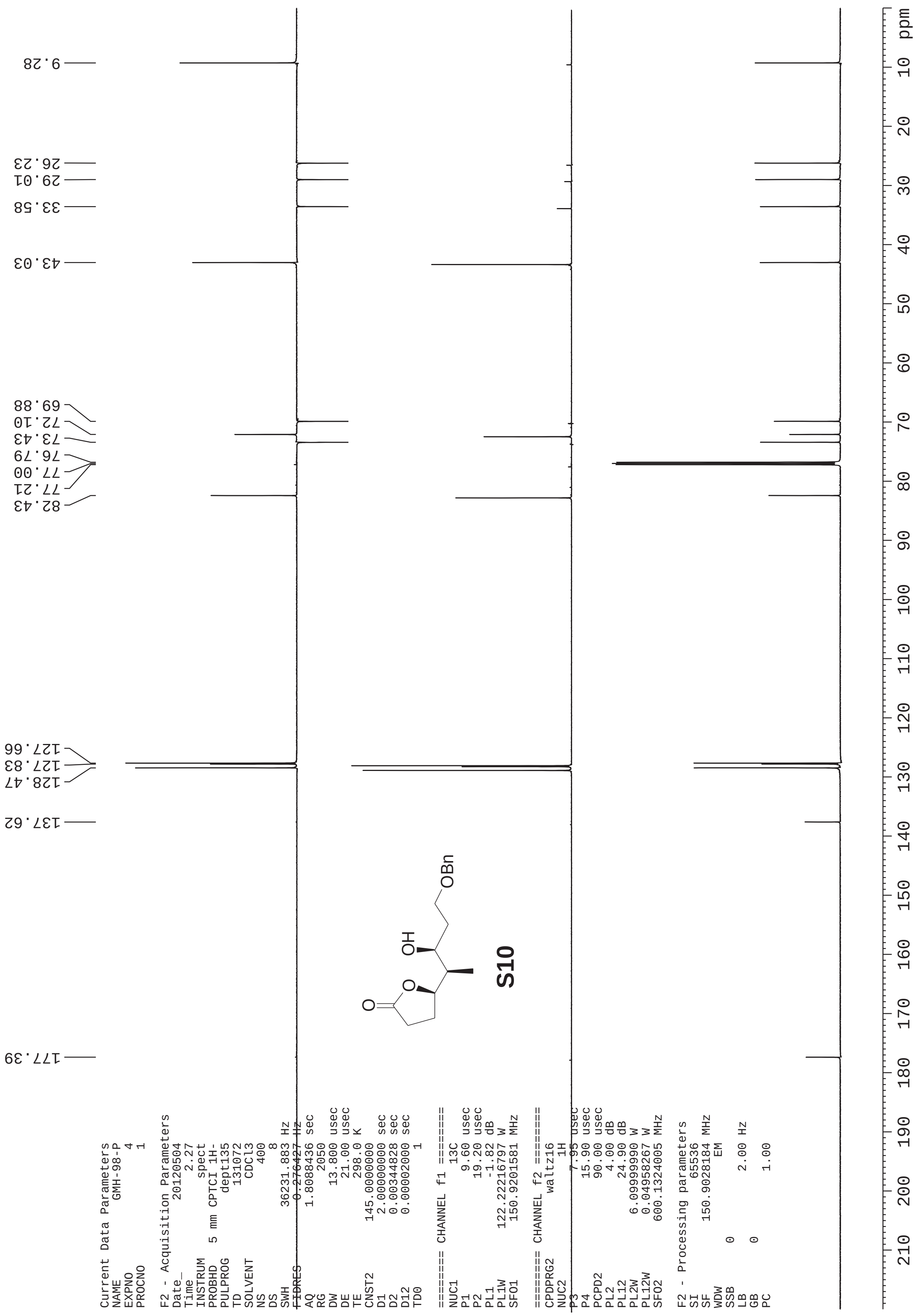

Supplementary Figure 60. ^{13}C and DEPT NMR spectra of compound S10.

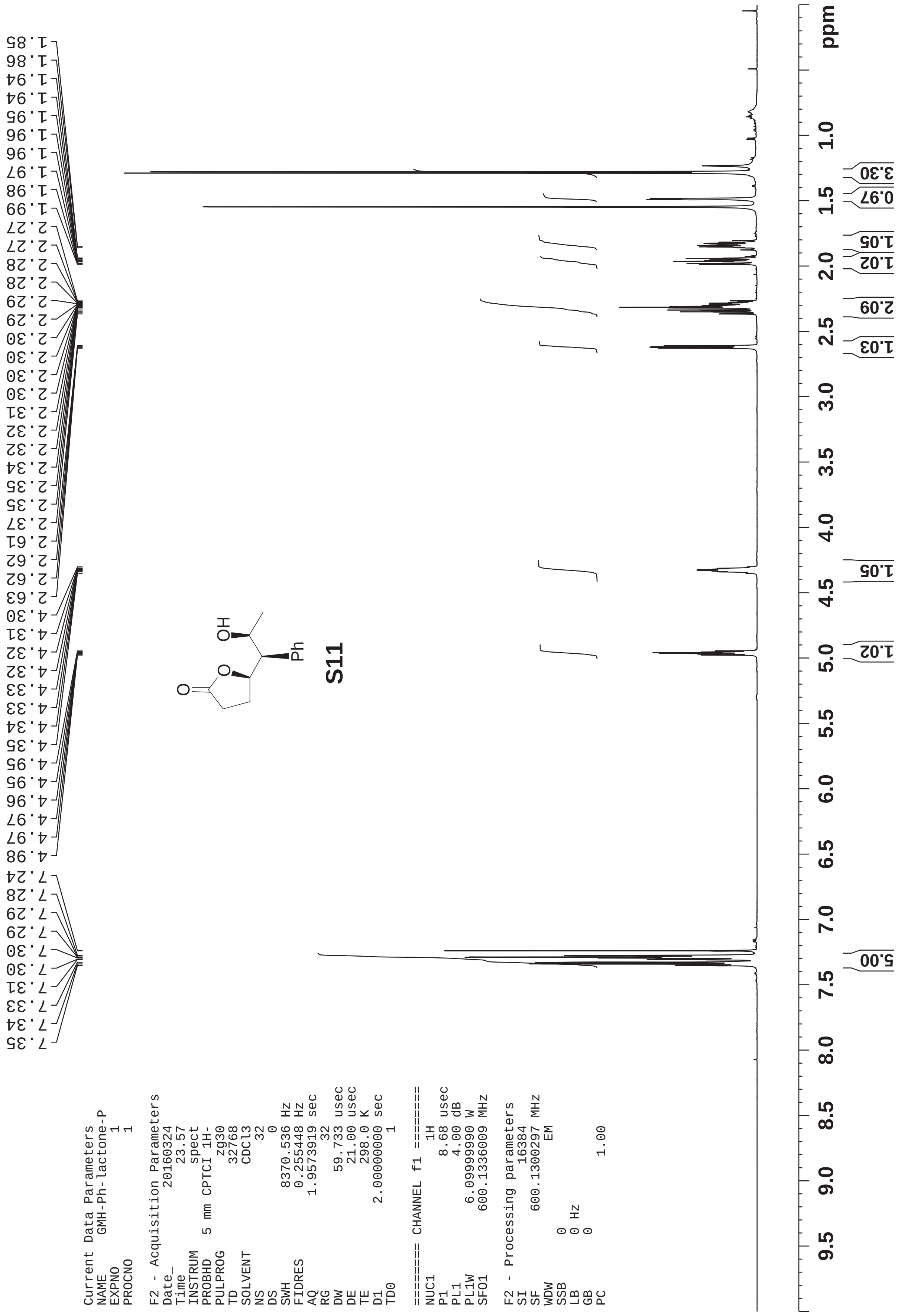

Supplementary Figure 61. ^{1}H NMR spectrum of compound S11.

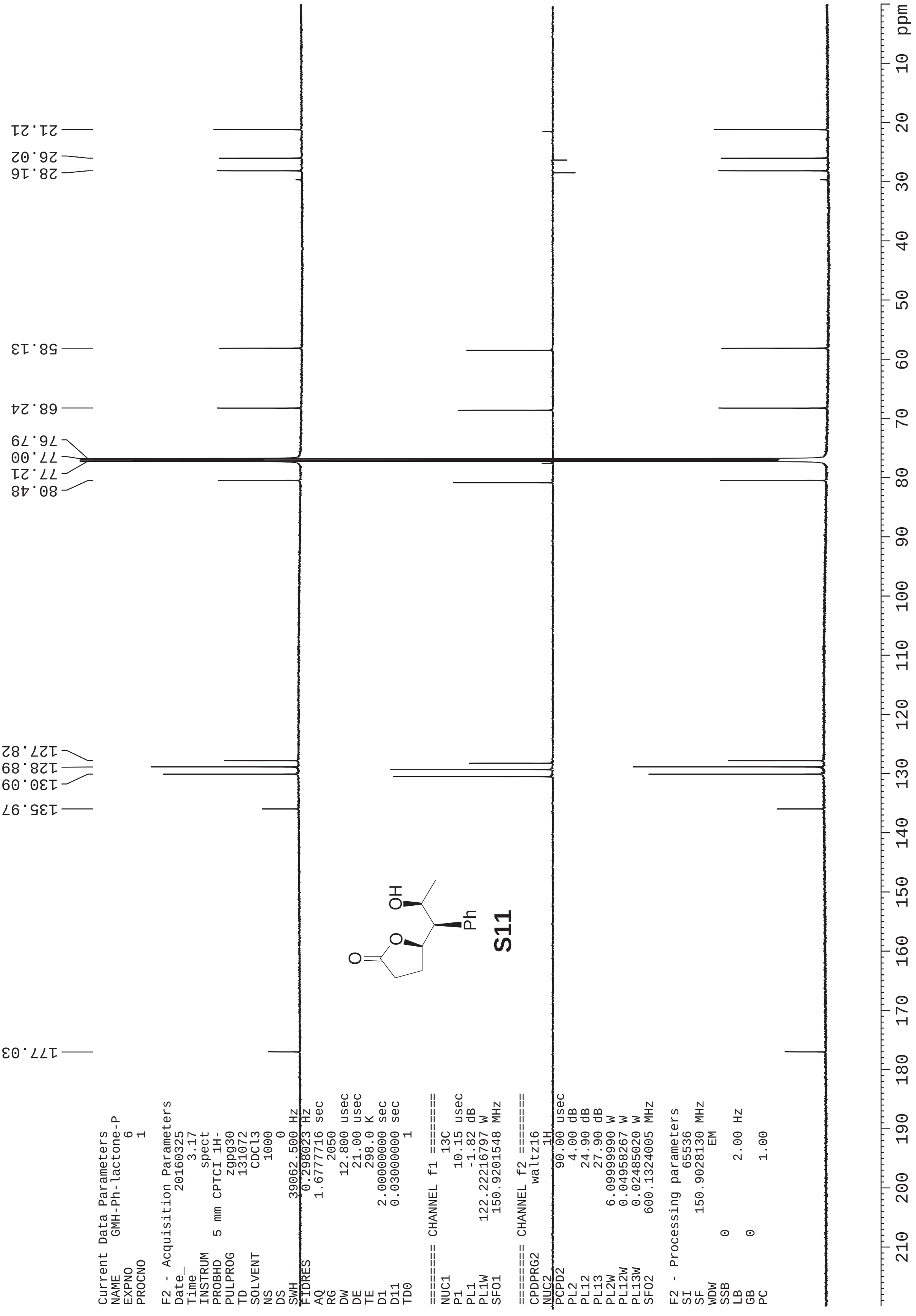

Supplementary Figure 62. ^{13}C and DEPT NMR spectra of compound S11.

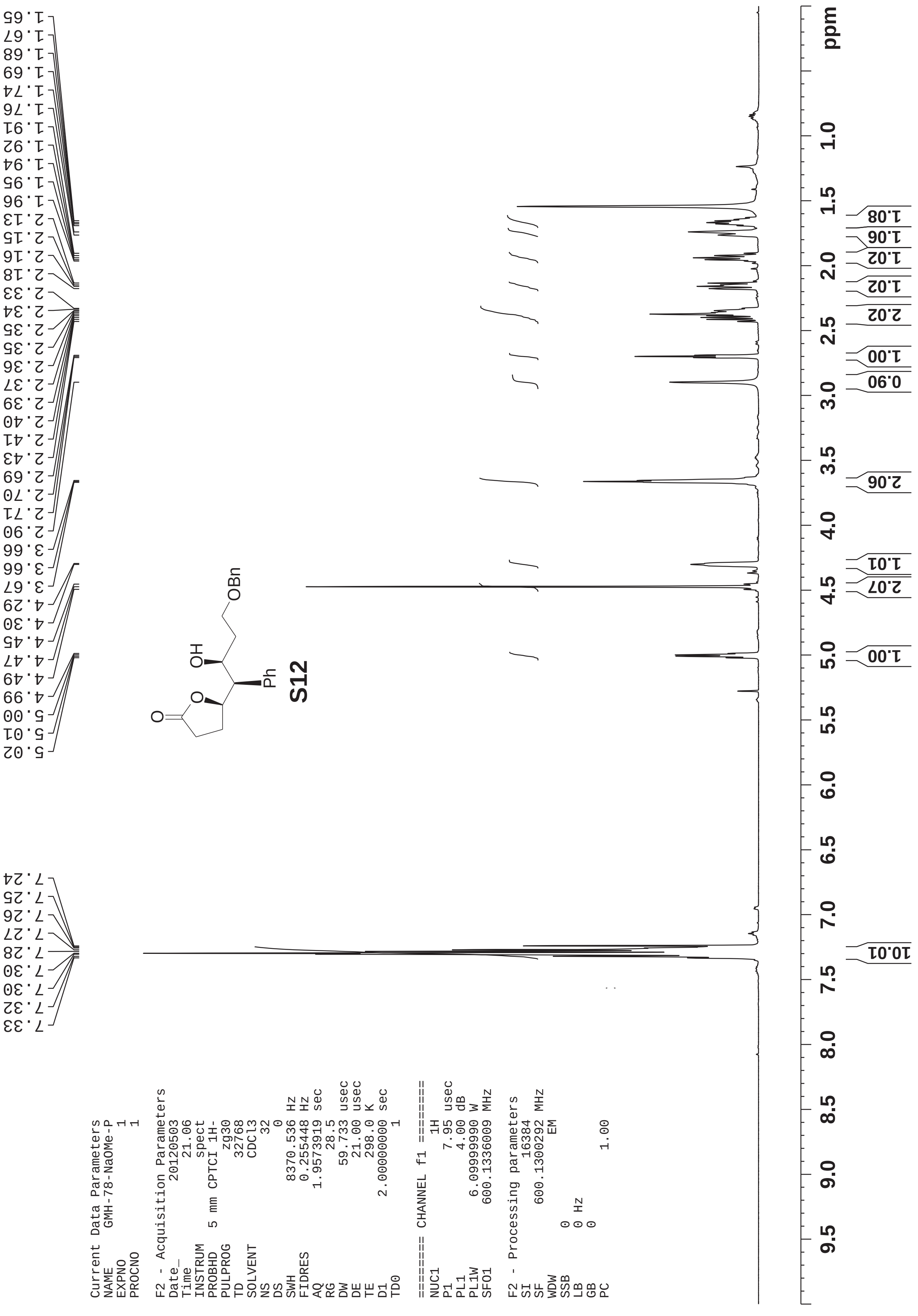

Supplementary Figure 63. ^{1}H NMR spectrum of compound S12.

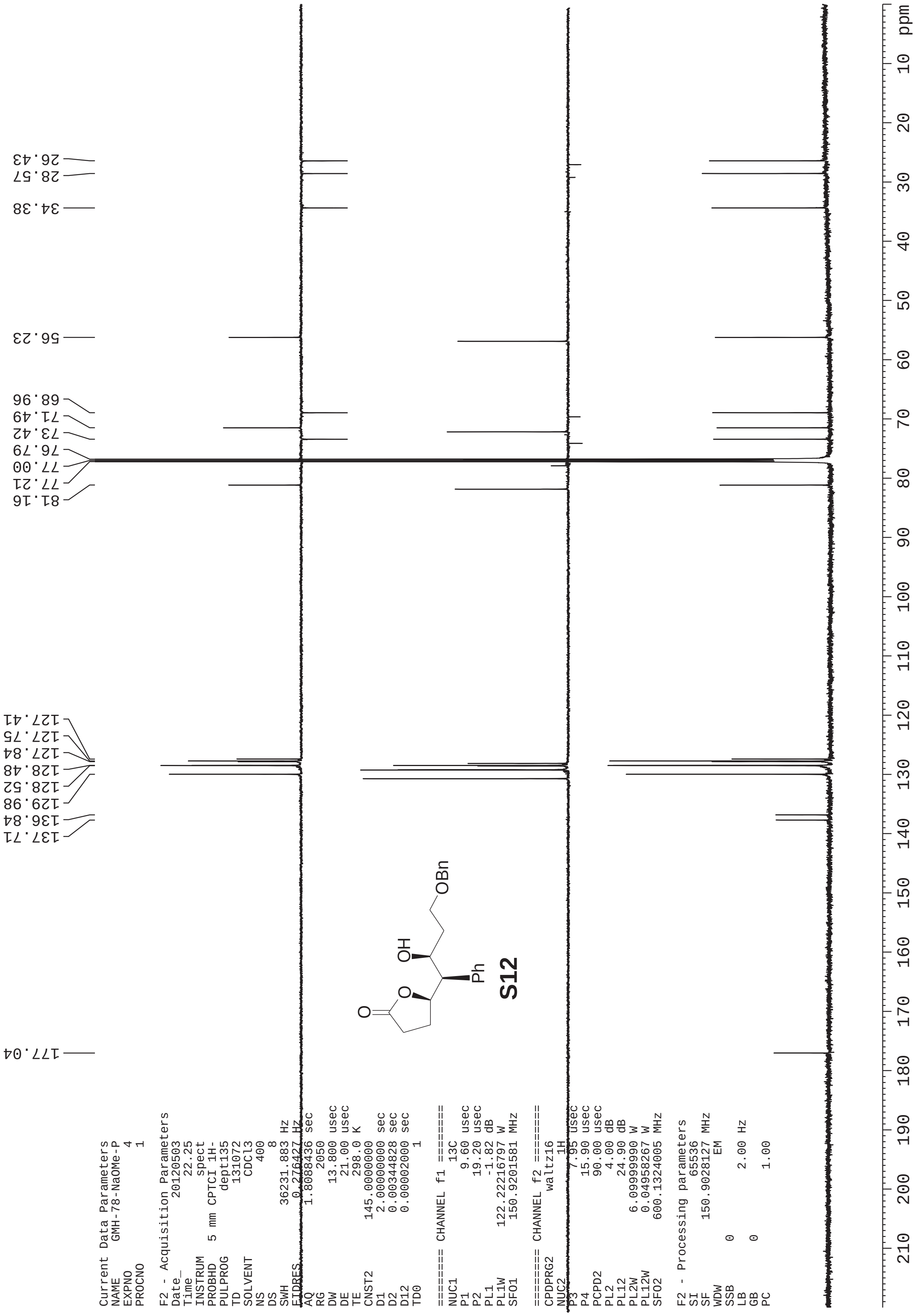

Supplementary Figure 64. ^{13}C and DEPT NMR spectra of compound S12.

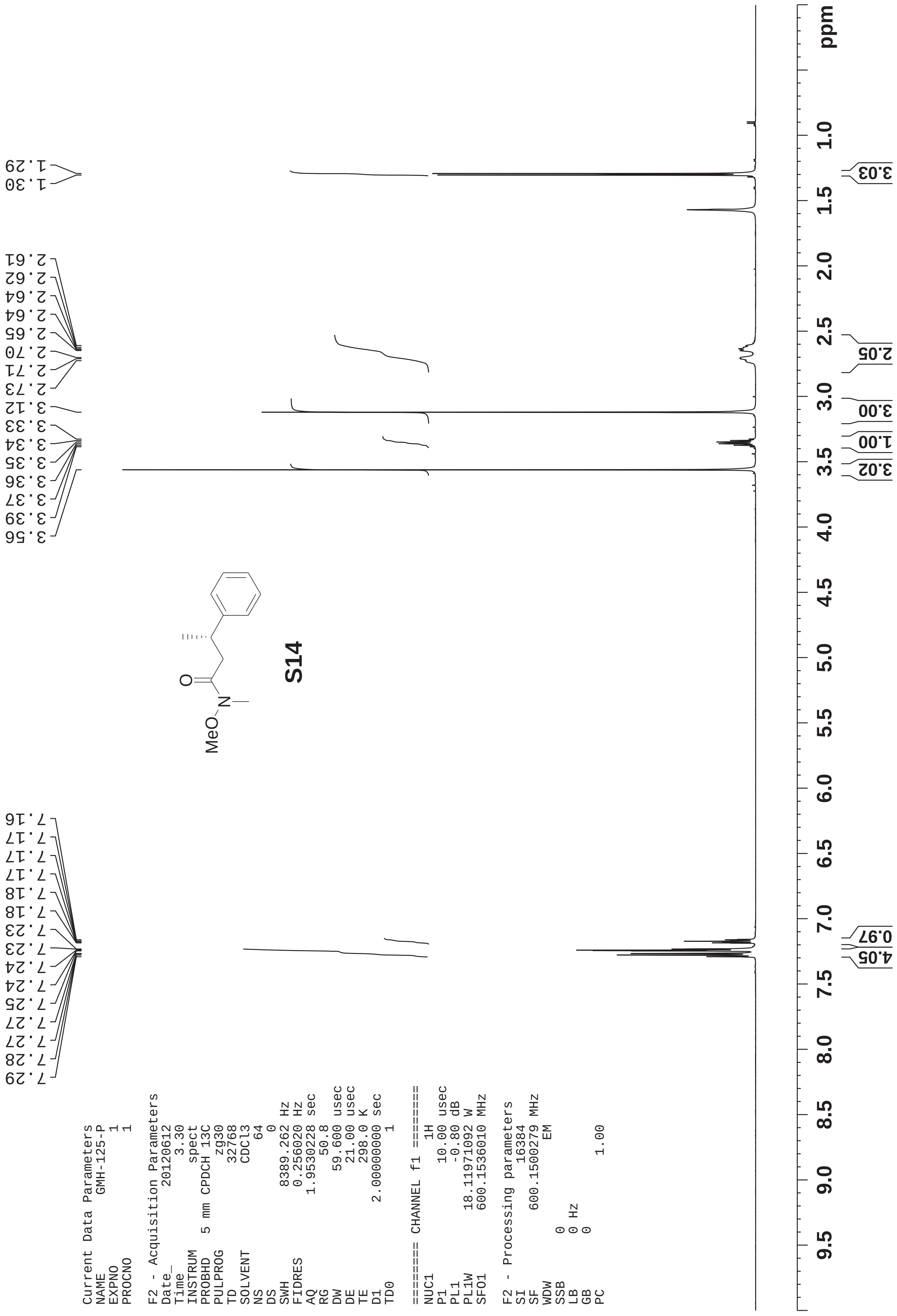

Supplementary Figure 65. ^{1}H NMR spectrum of compound S14.

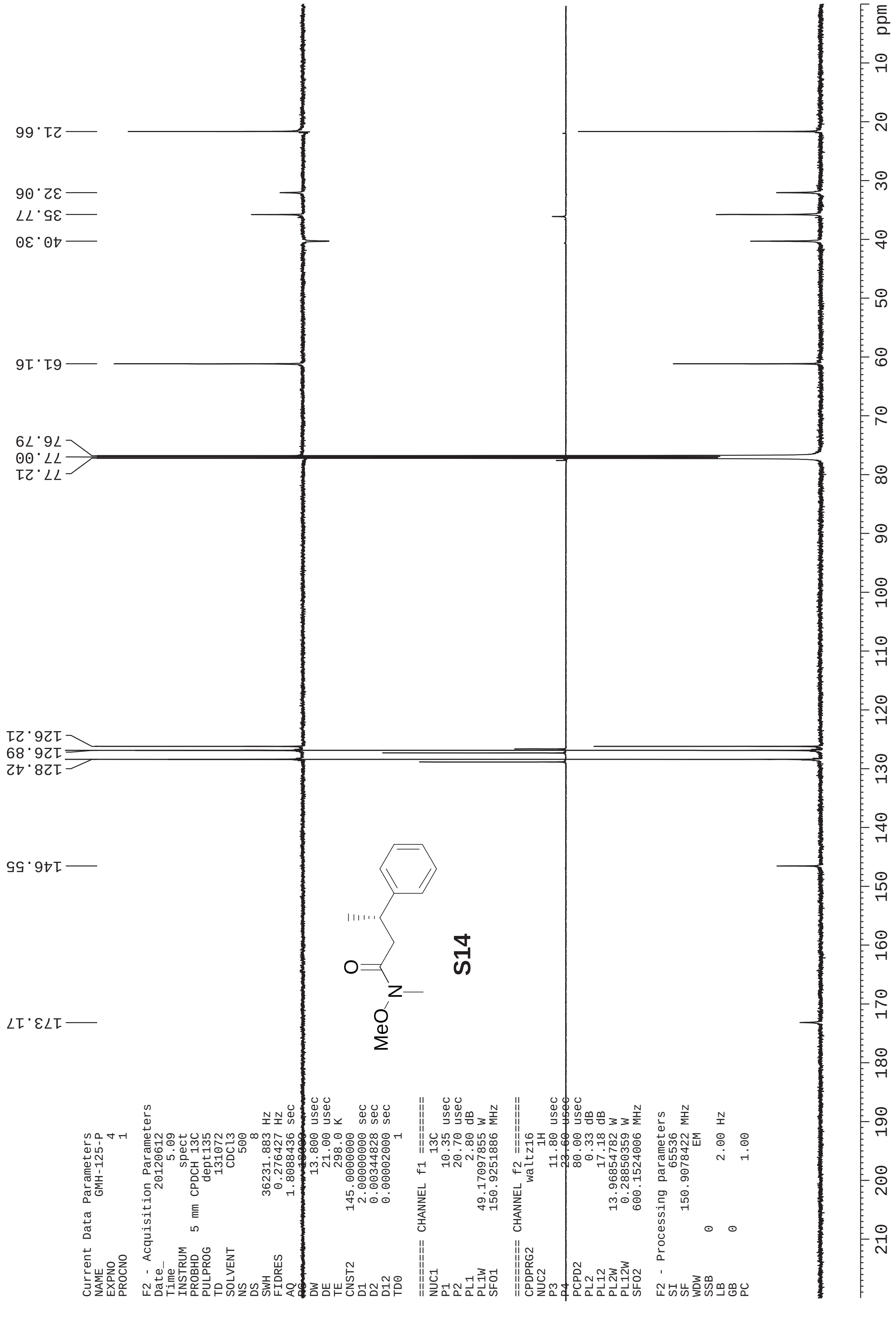

Supplementary Figure 66. ^{13}C and DEPT NMR spectra of compound S14.

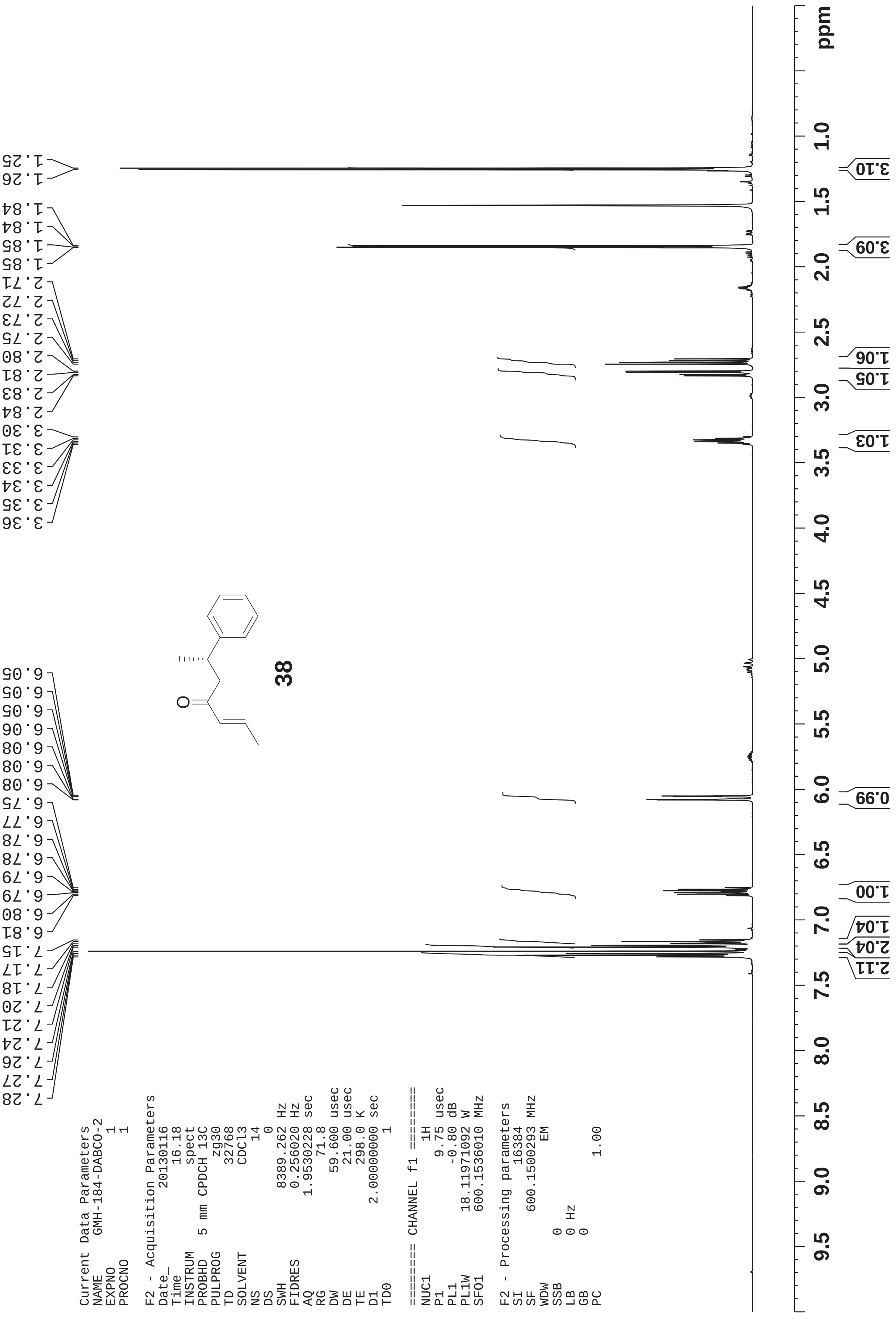

Supplementary Figure 67. ^{1}H NMR spectrum of compound 38.

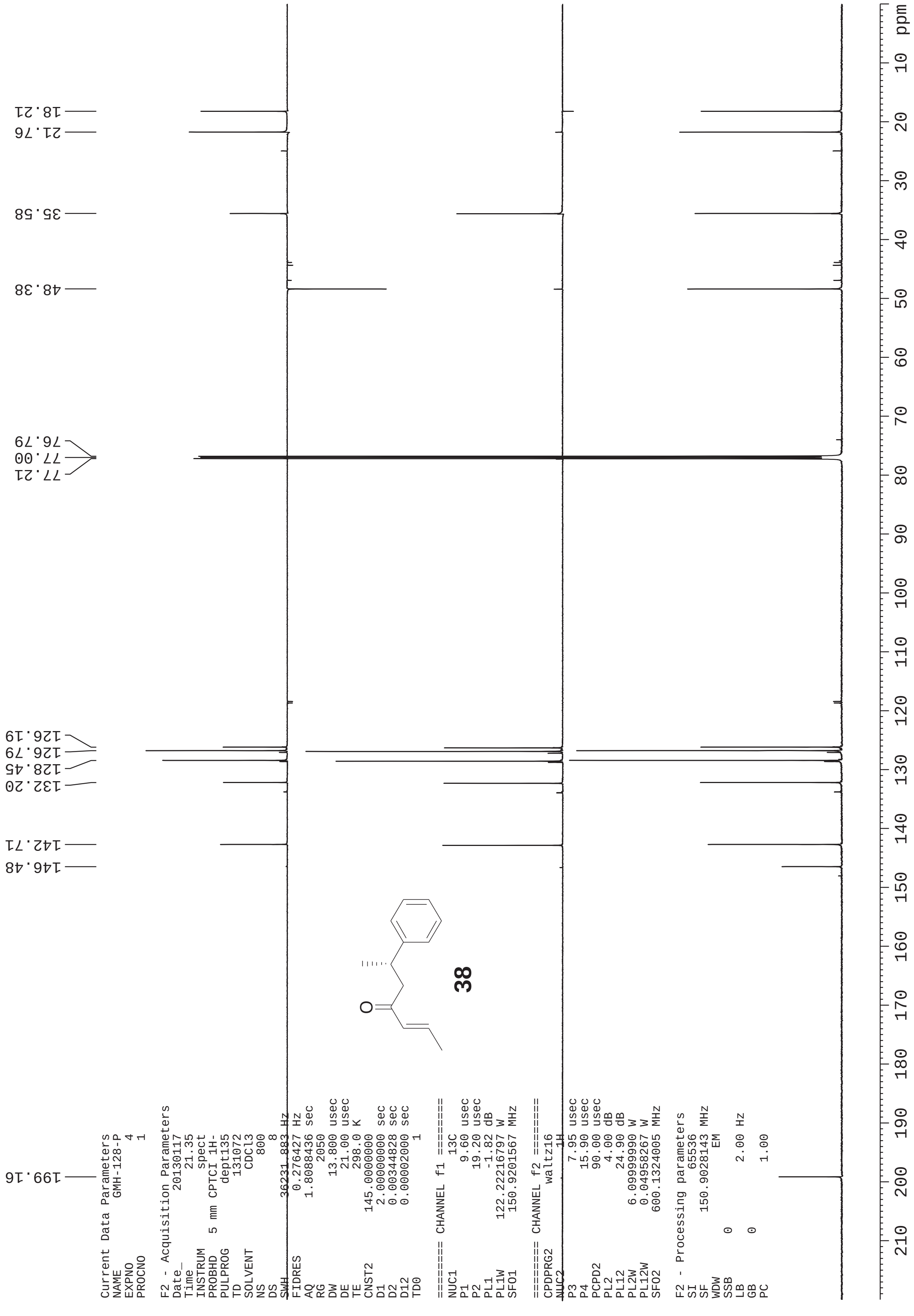

Supplementary Figure 68. ^{13}C and DEPT NMR spectra of compound 38.

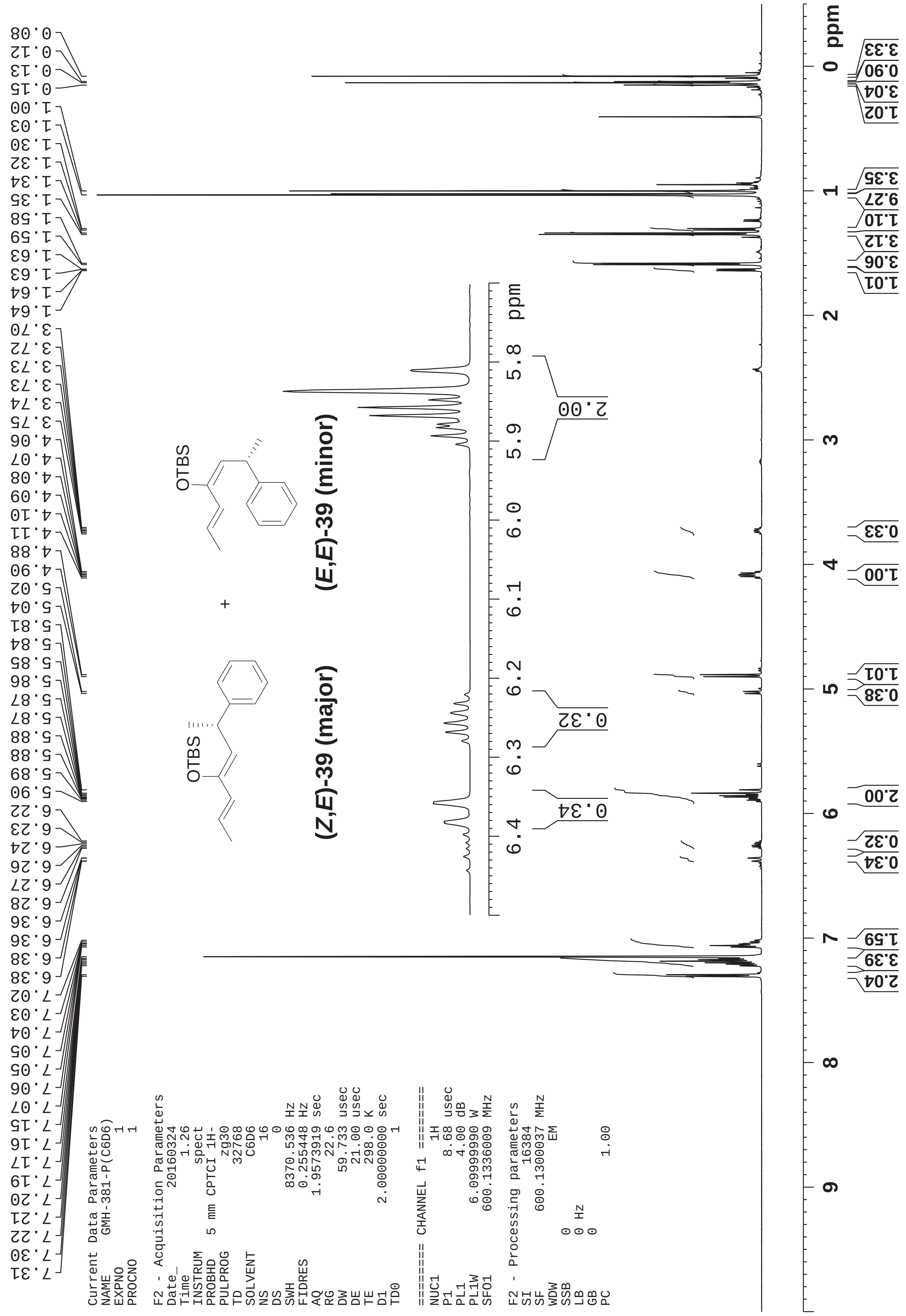

Supplementary Figure 69. ¹H NMR spectrum of compound 39.

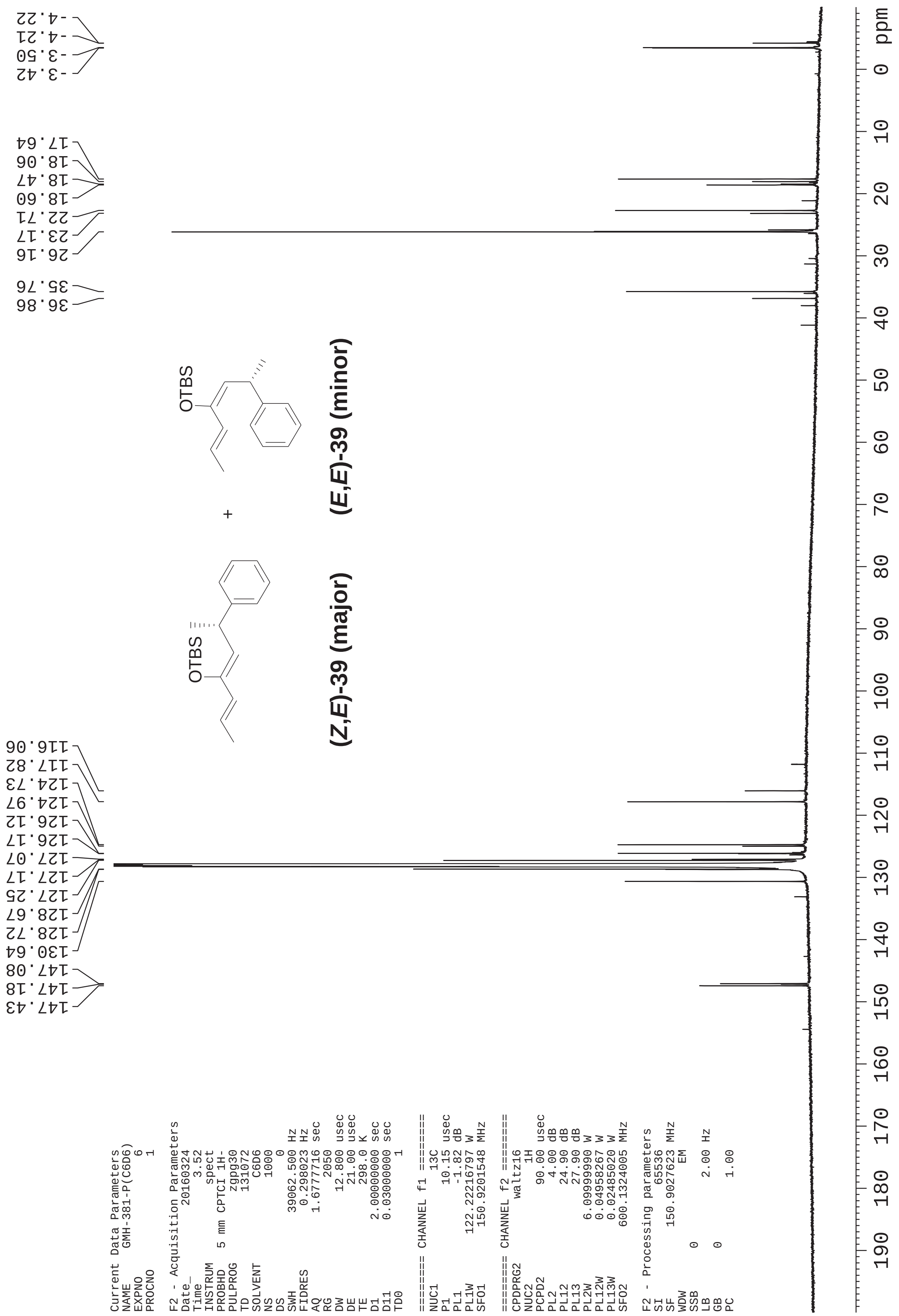

Supplementary Figure 70. ^{13}C NMR spectra of compound 39.

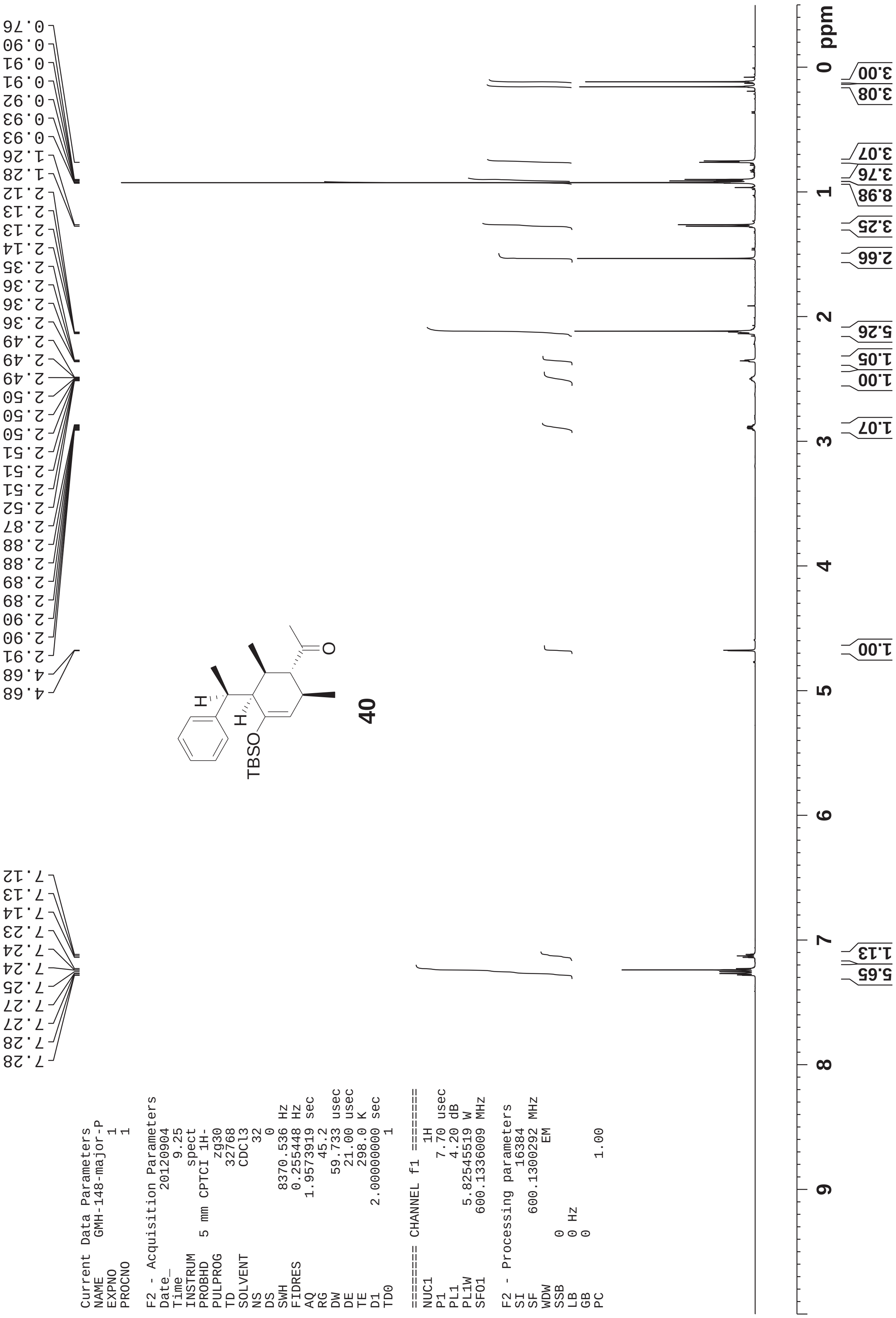

Supplementary Figure 71. ^{1}H NMR spectrum of compound 40.

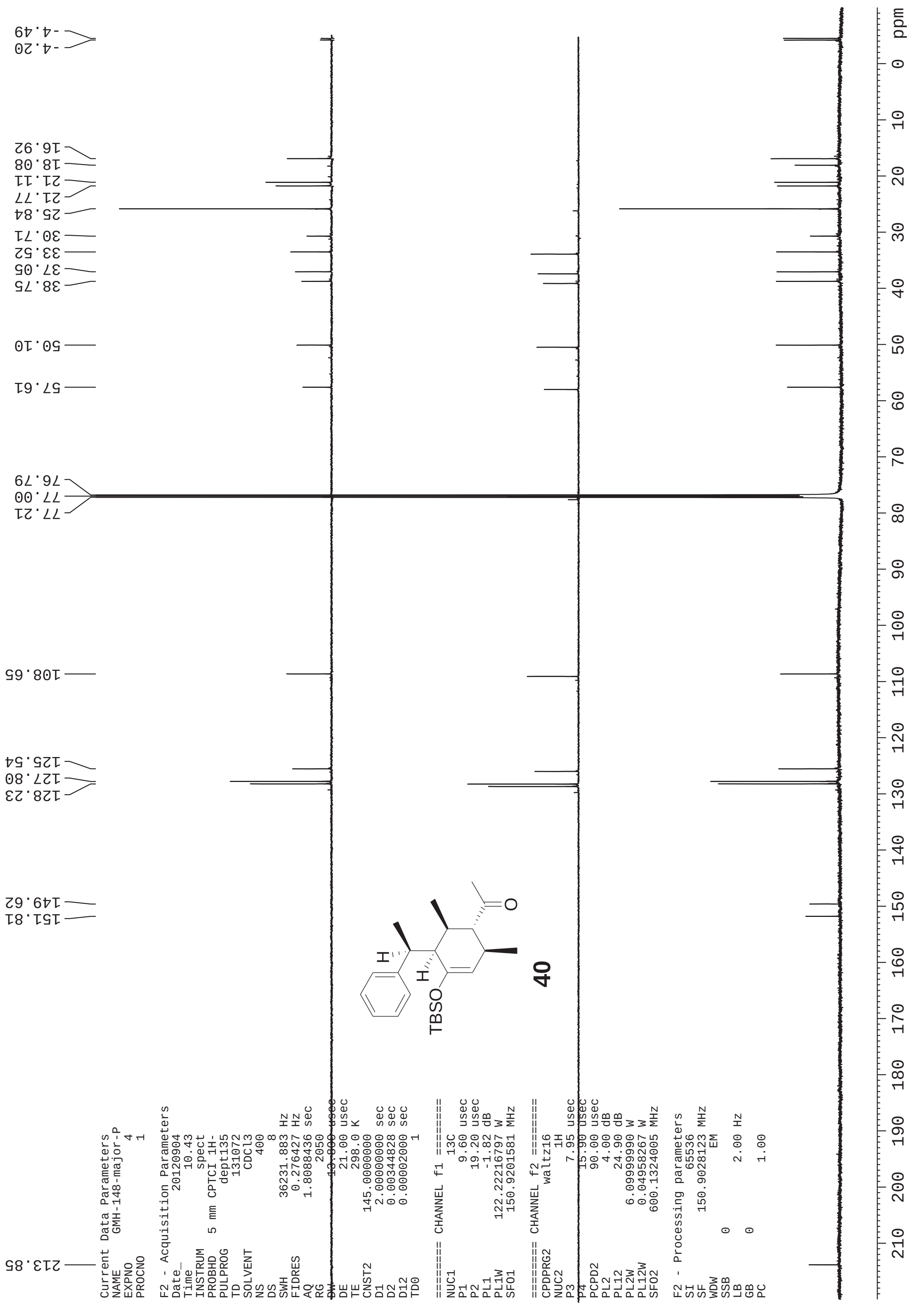

Supplementary Figure 72. ^{13}C and DEPT NMR spectra of compound 40.

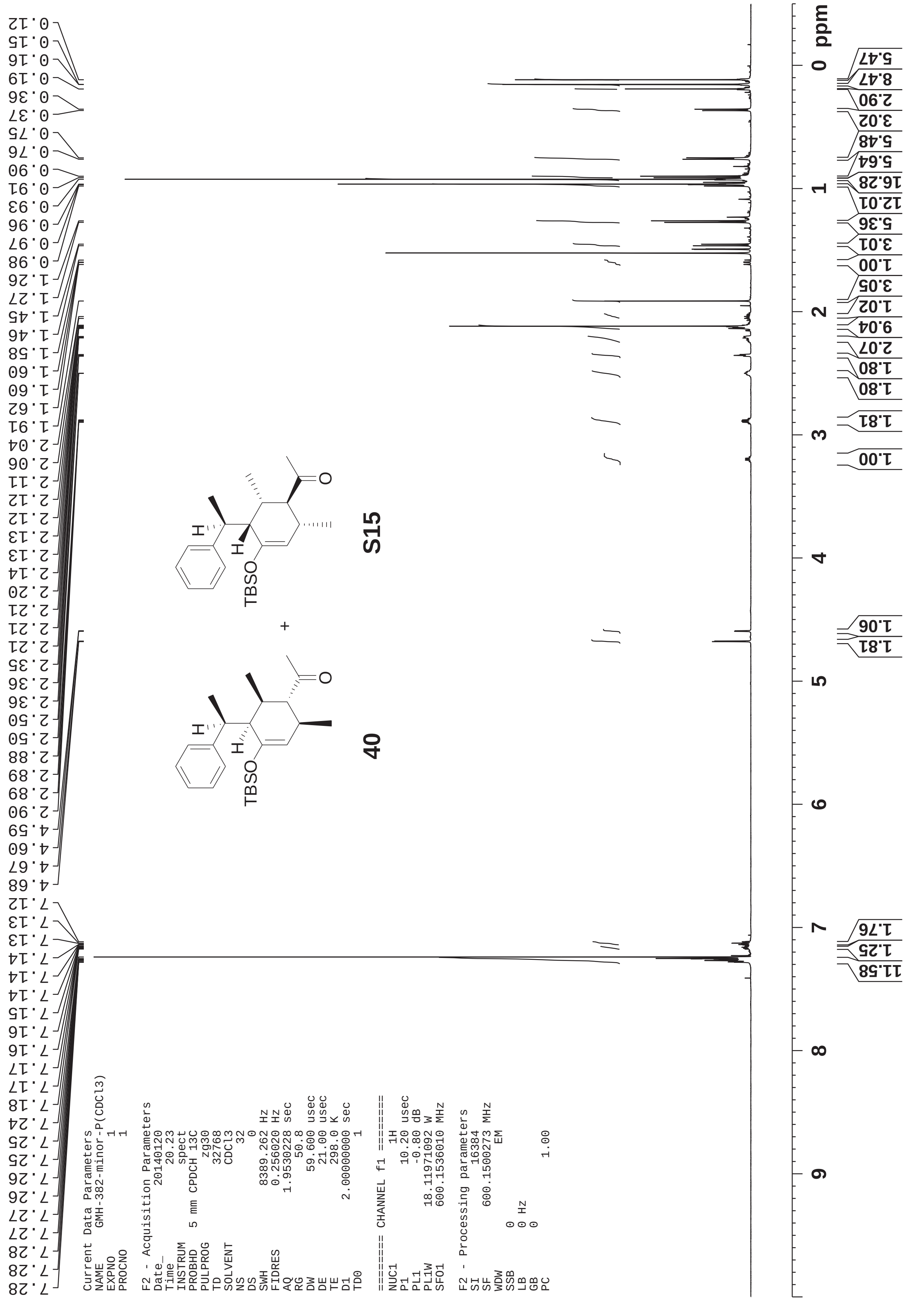

Supplementary Figure 73. ^{1}H NMR spectrum of mixed *exo*-adducts 40 and S15.

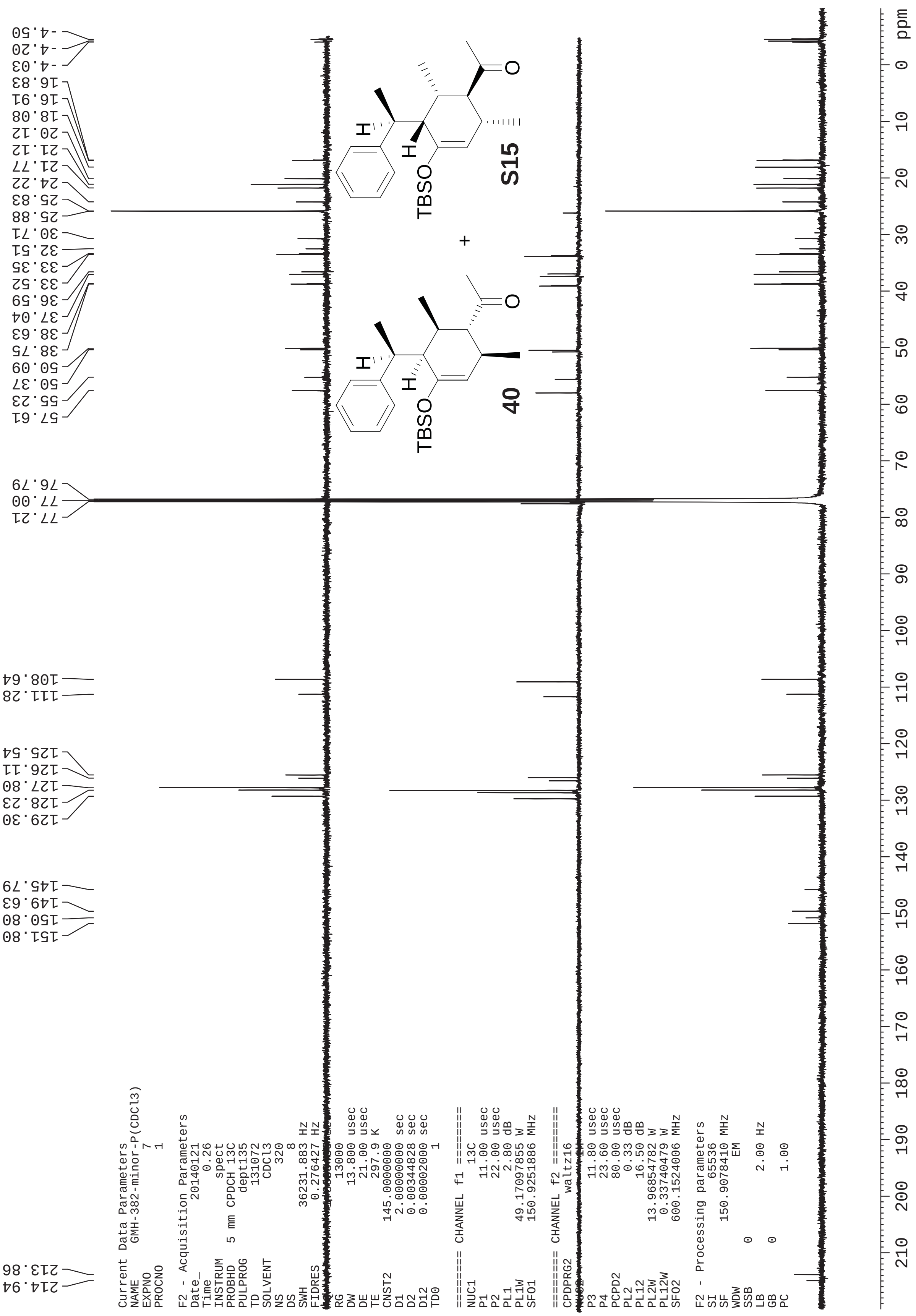

Supplementary Figure 74. ^{13}C and DEPT NMR spectra of mixed compounds 40 and S15.

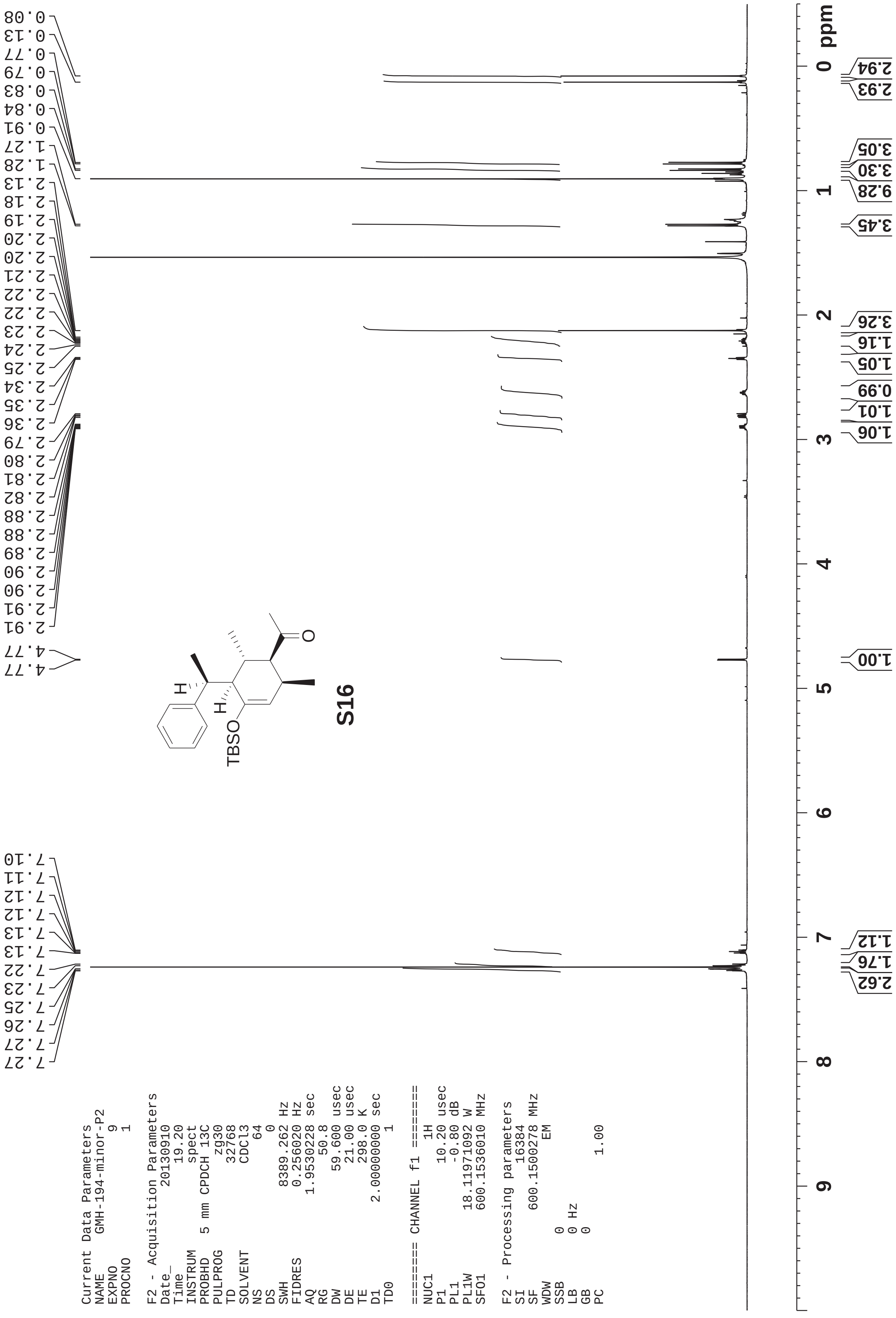

Supplementary Figure 75. ^{1}H NMR spectrum of compound S16.

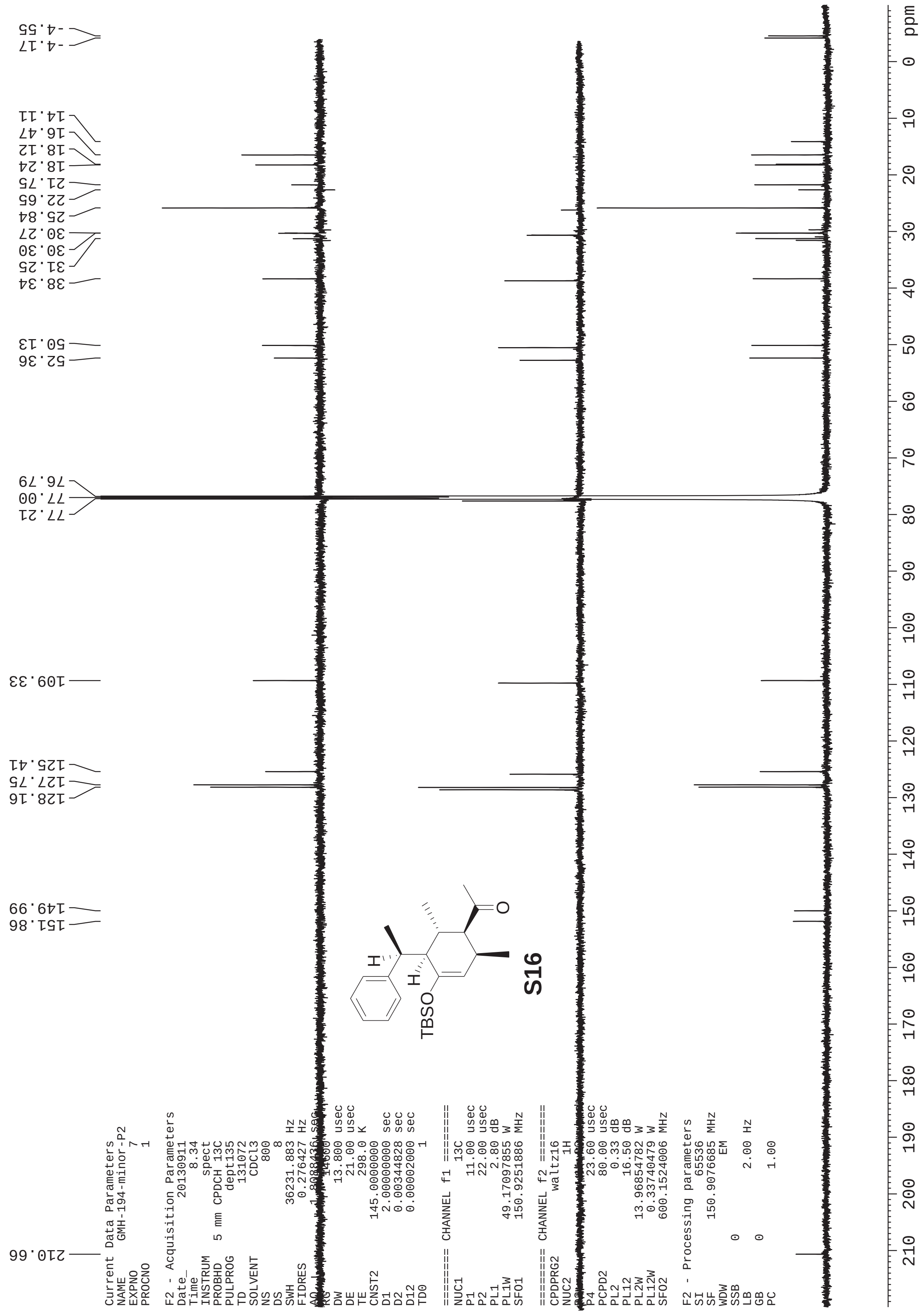

Supplementary Figure 76. ^{13}C and DEPT NMR spectra of compound S16.

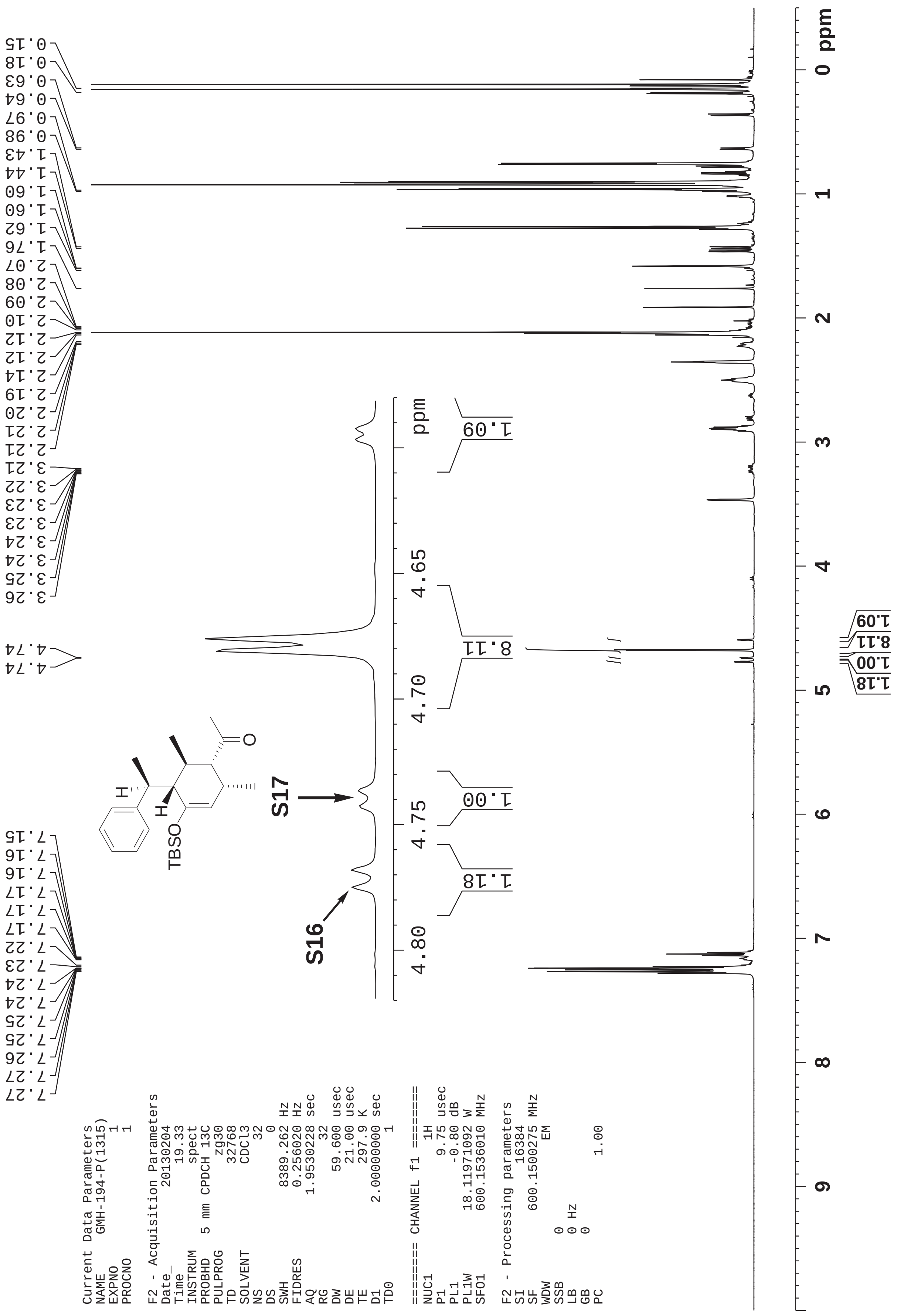

Supplementary Figure 77. ^{1}H NMR spectrum of compound S17 mixed with S16.

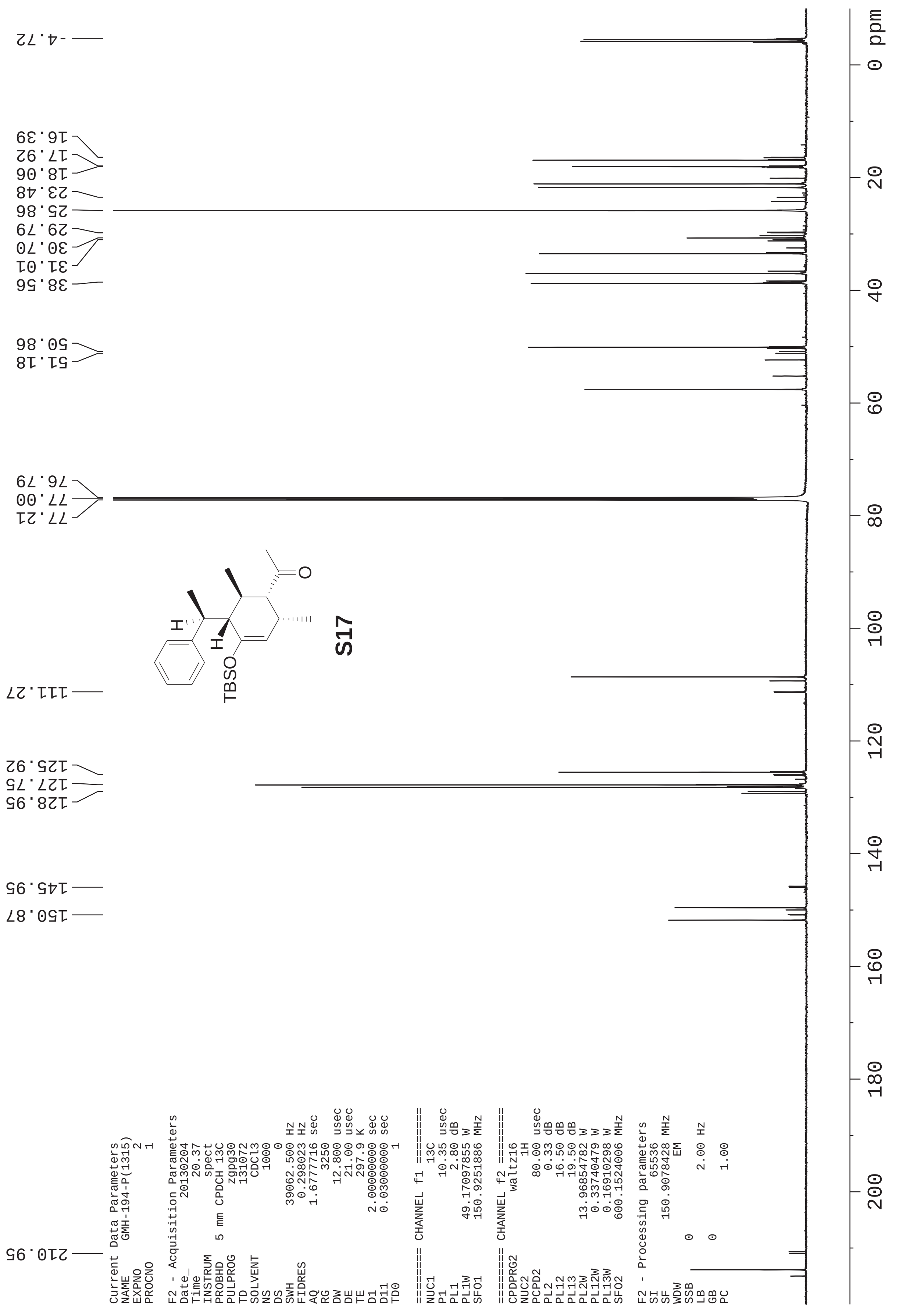

Supplementary Figure 78. ^{13}C NMR spectra of compound S17 mixed with S16.

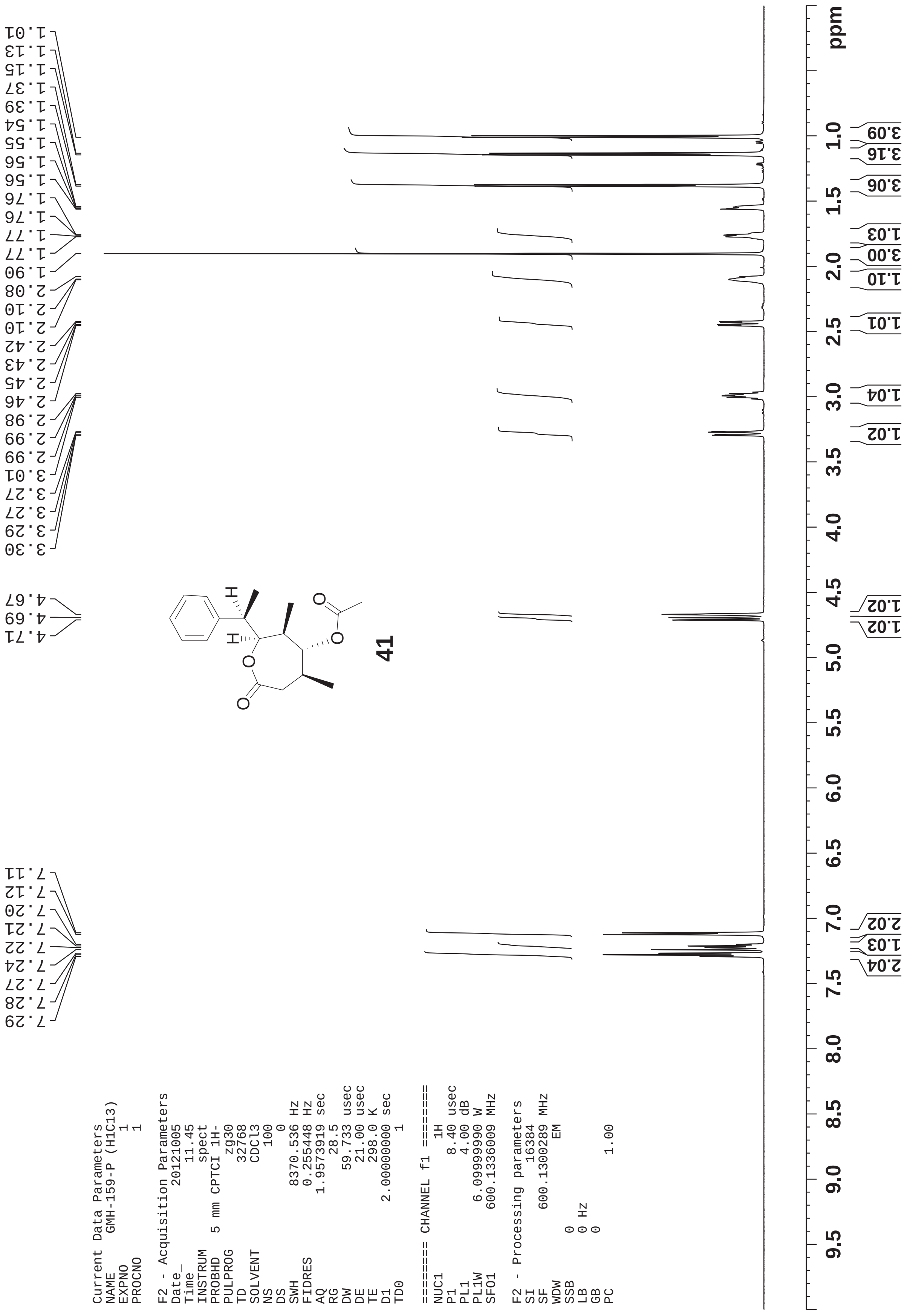

Supplementary Figure 79. ^{1}H NMR spectrum of compound 41.

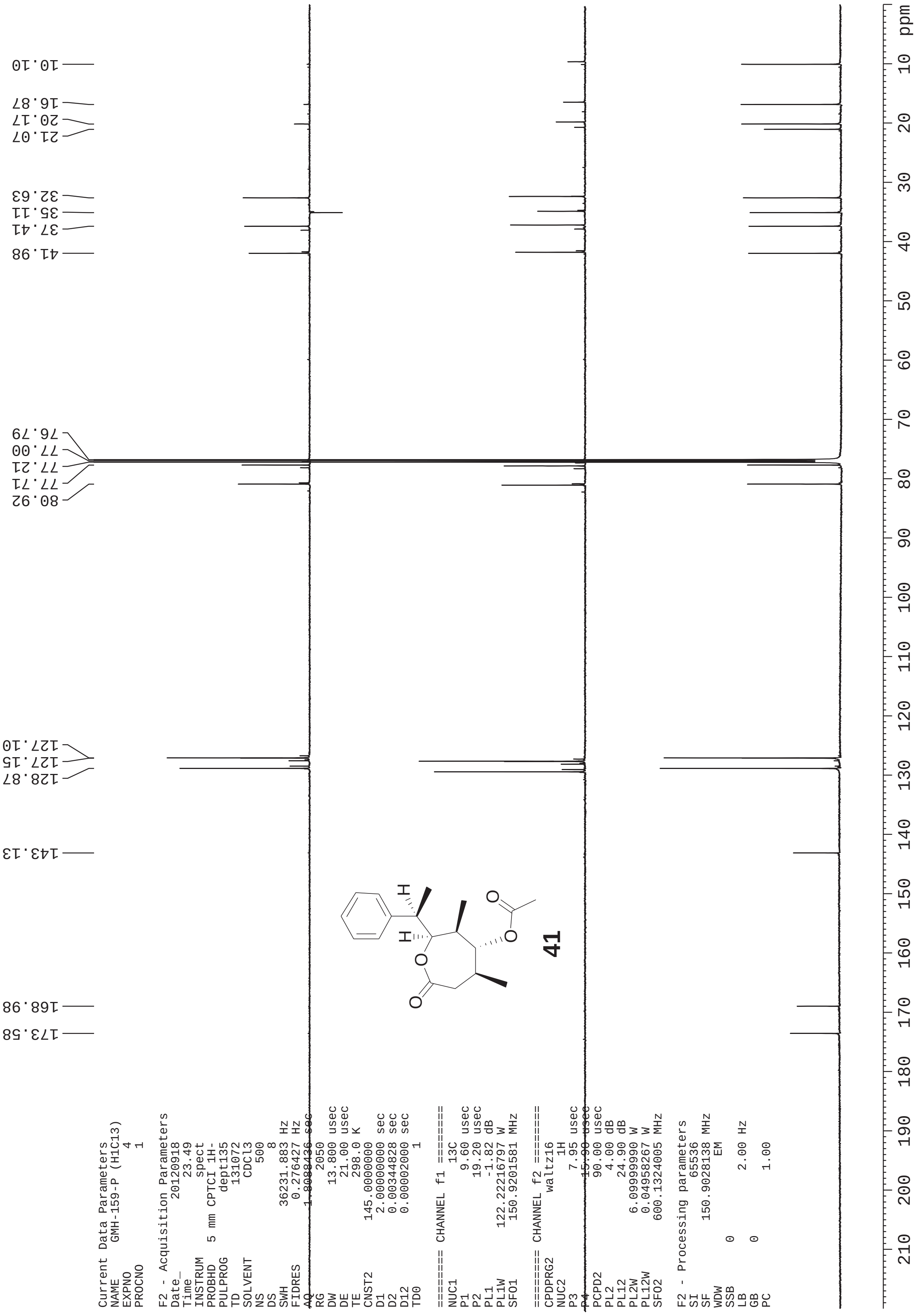

Supplementary Figure 80. ^{13}C and DEPT NMR spectra of compound 41.

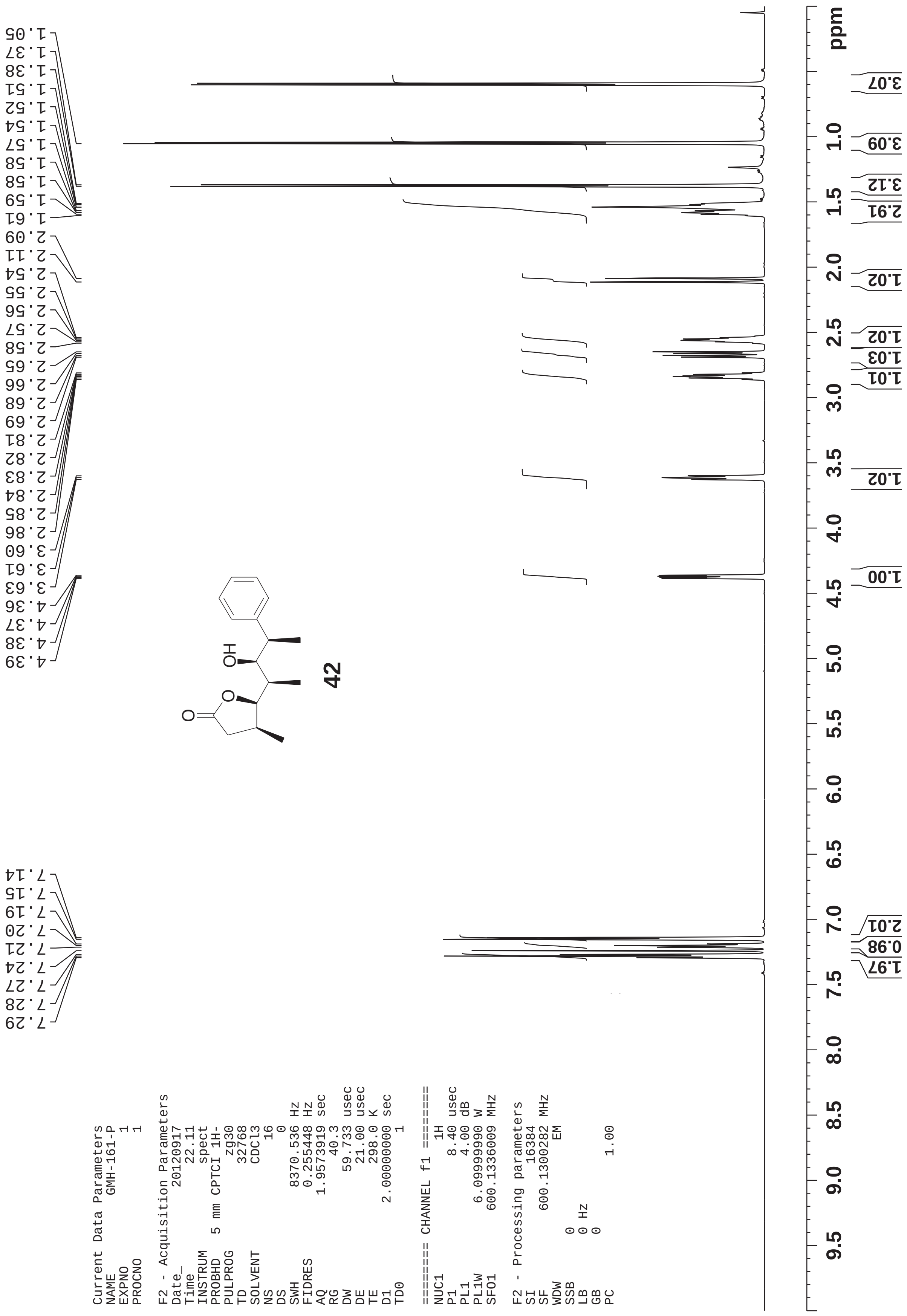

Supplementary Figure 81. ^{1}H NMR spectrum of compound 42.

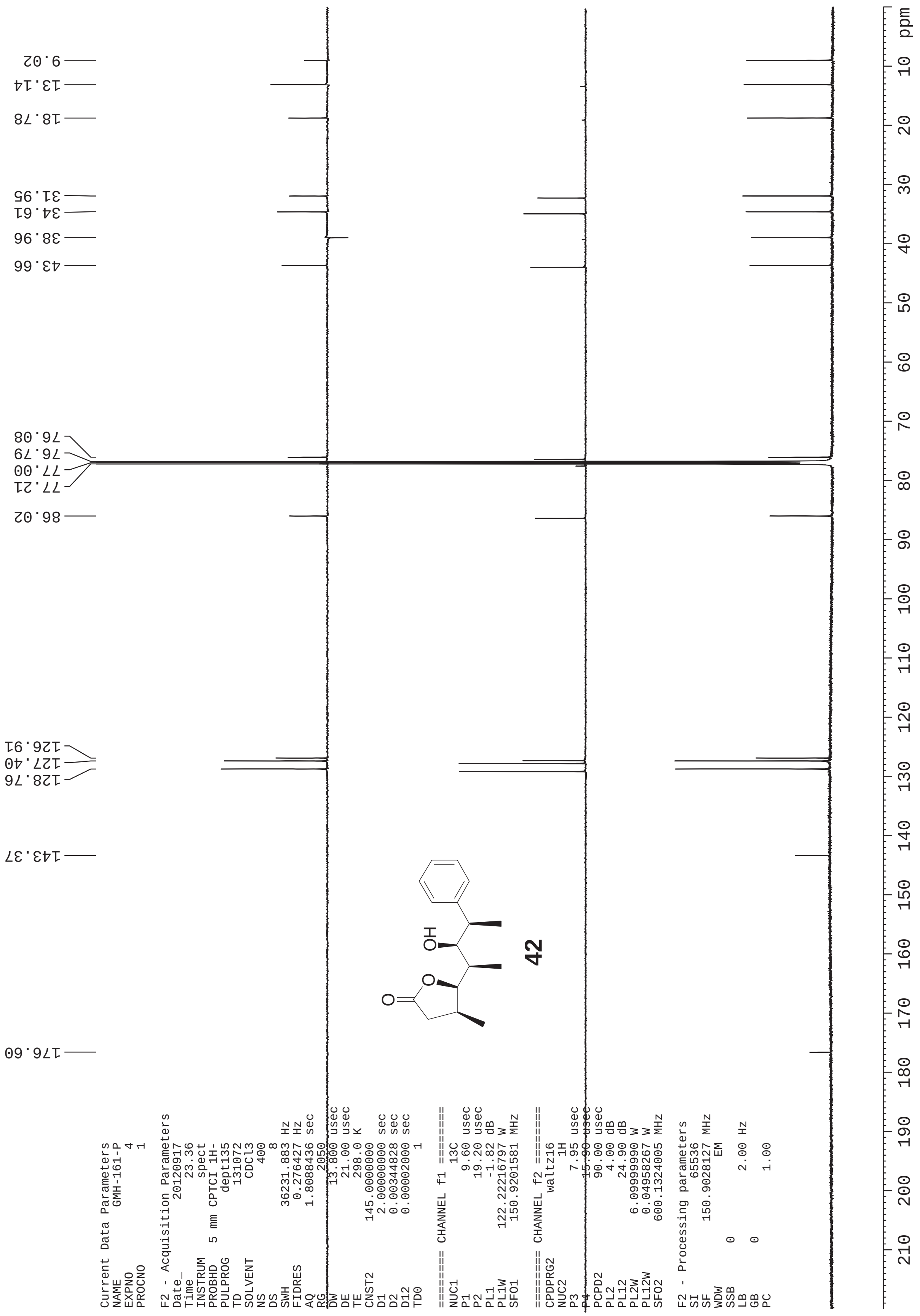

Supplementary Figure 82. ^{13}C and DEPT NMR spectra of compound 42.

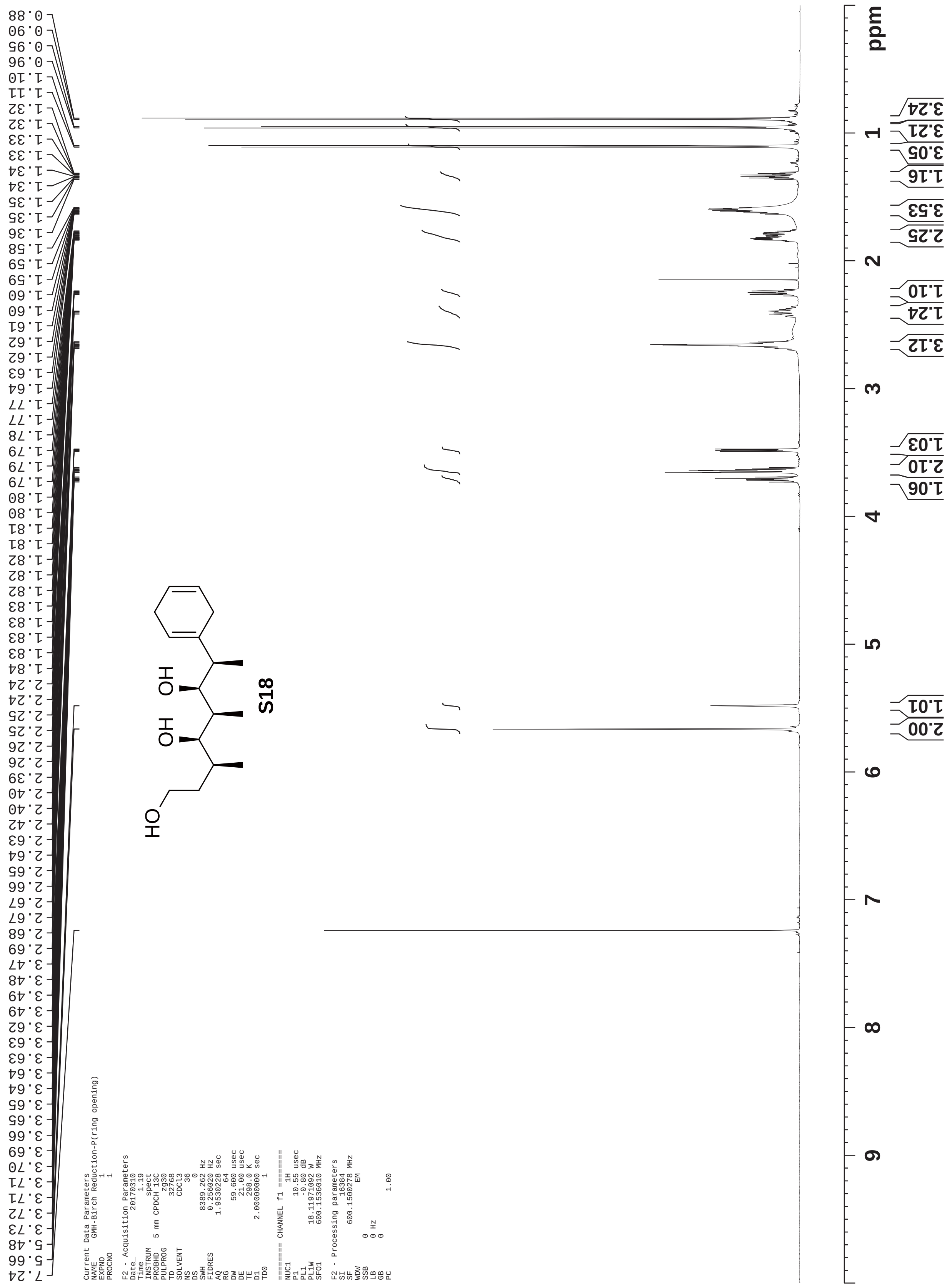

Supplementary Figure 83. ^{1}H NMR spectrum of compound S18.

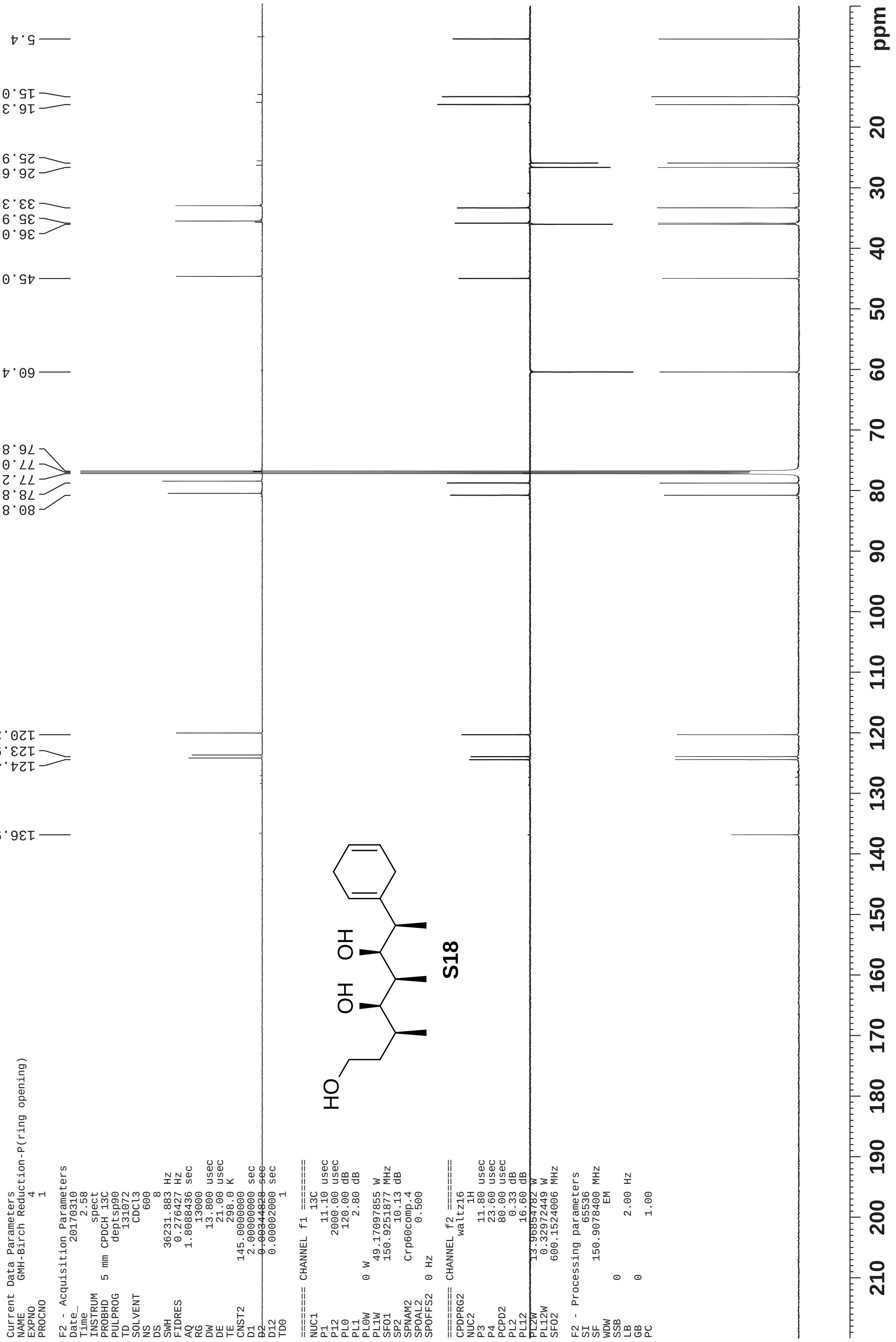

Supplementary Figure 84. ^{13}C and DEPT NMR spectra of compound S18.

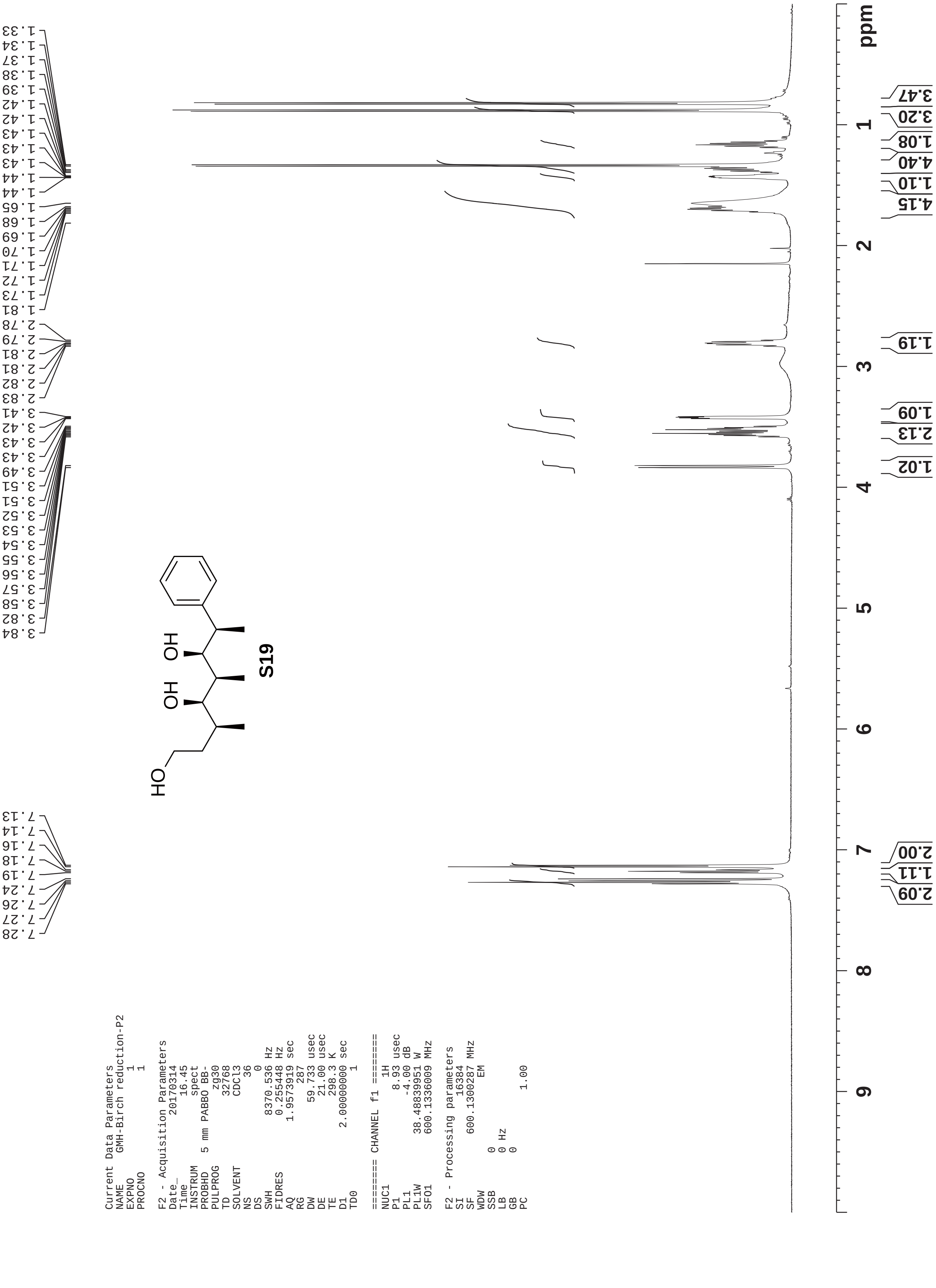

Supplementary Figure 85. ^{1}H NMR spectrum of compound S19.

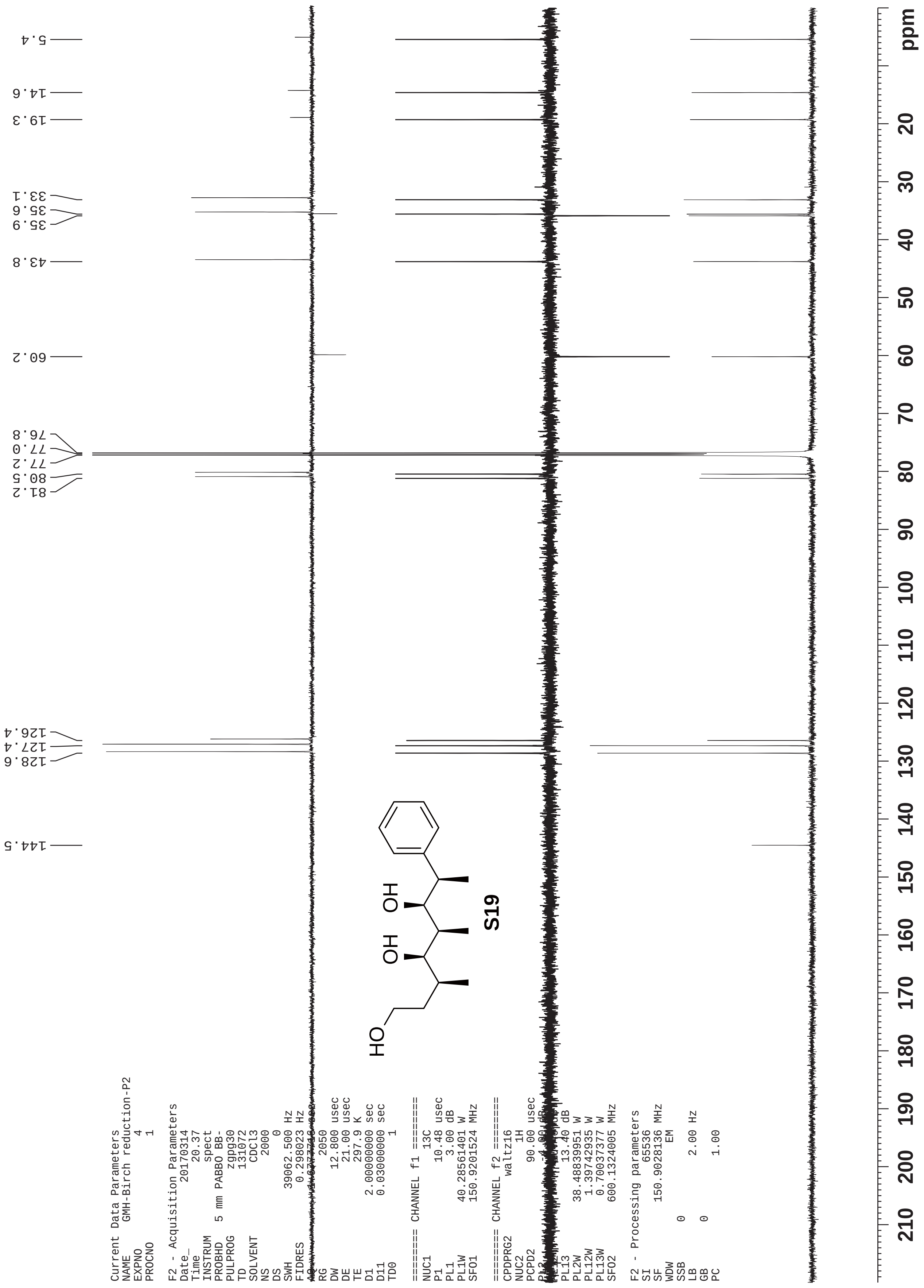

Supplementary Figure 86. ^{13}C and DEPT NMR spectra of compound S19.

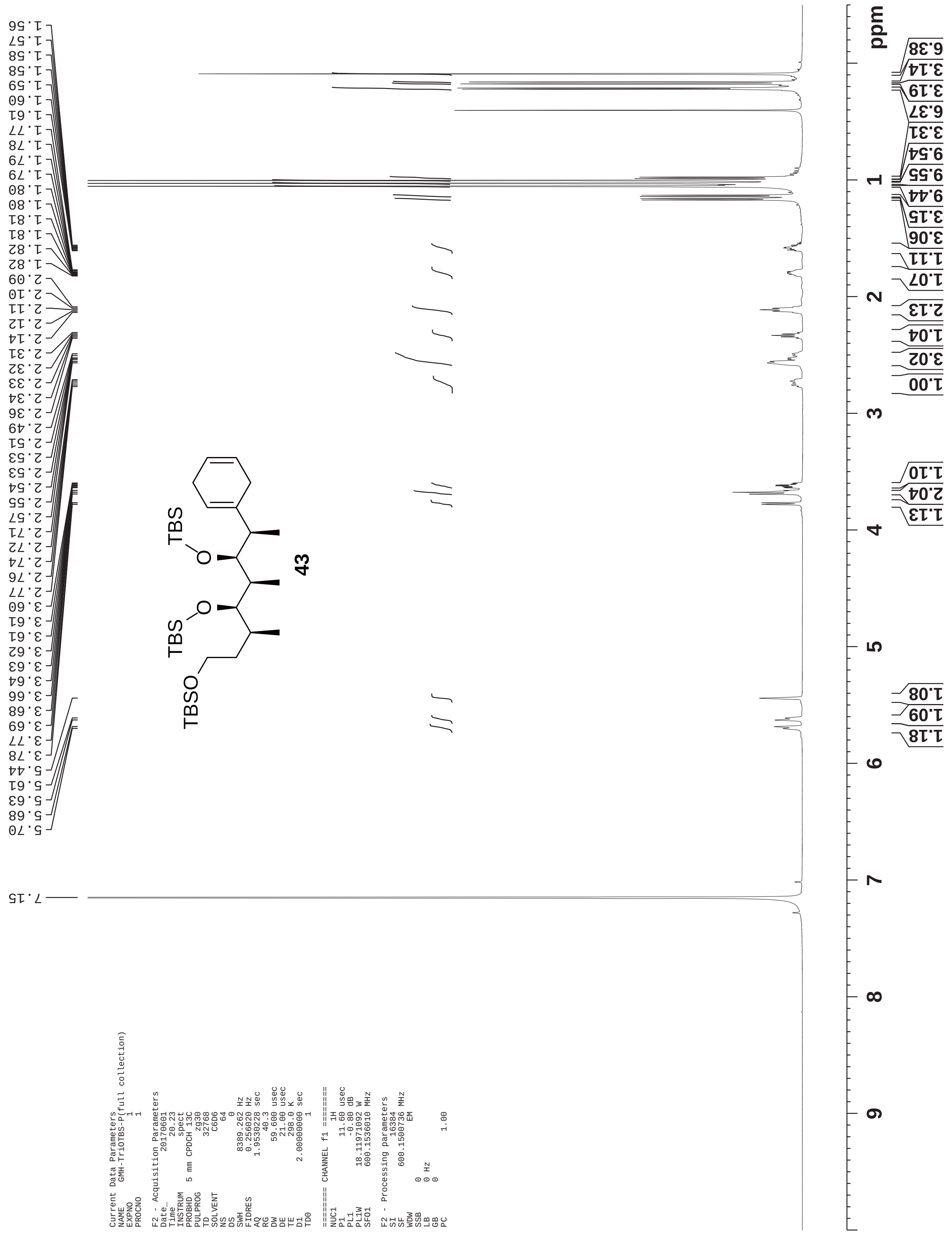

Supplementary Figure 87. ^{1}H NMR spectrum of compound 43.

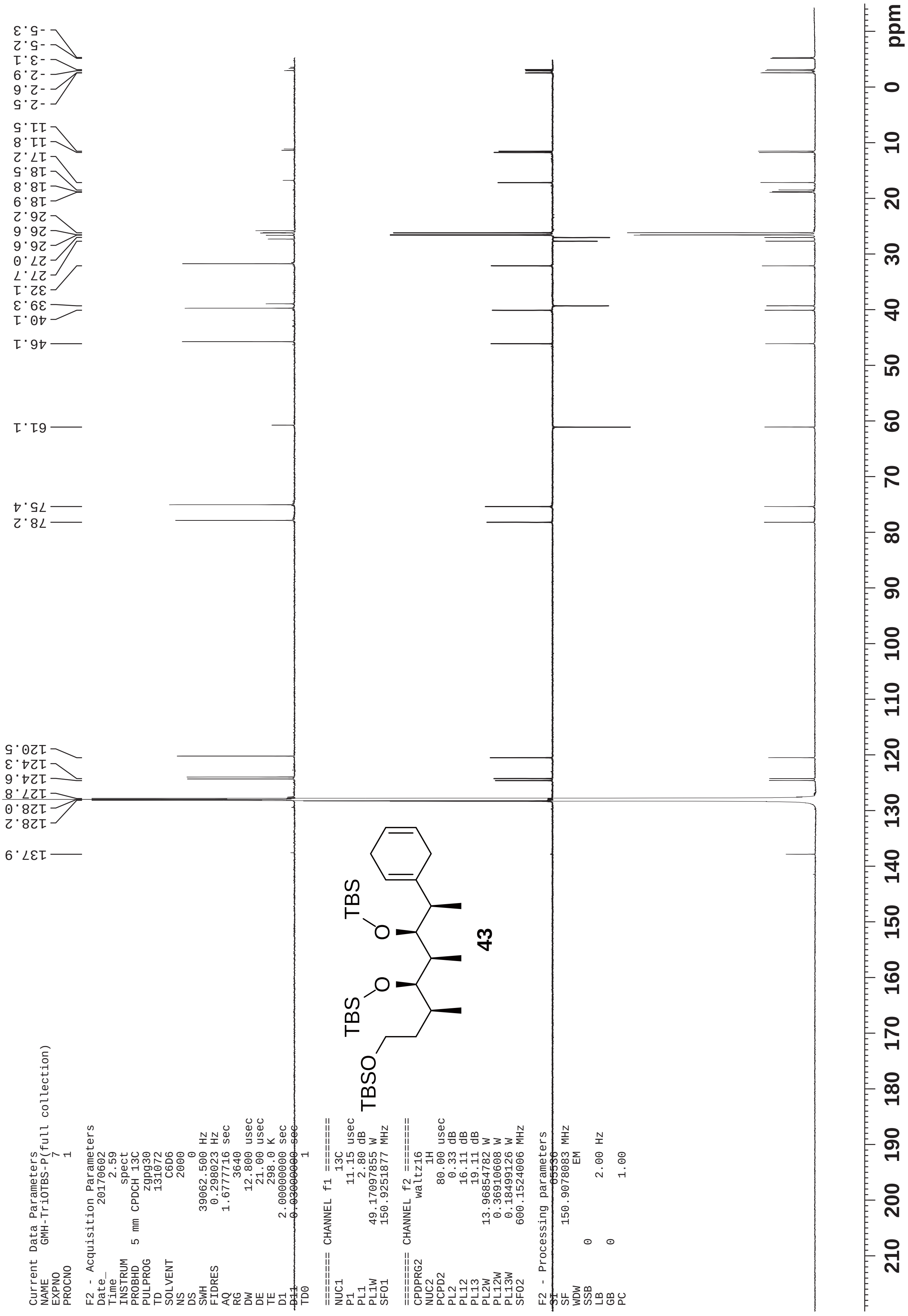

Supplementary Figure 88. ^{13}C and DEPT NMR spectra of compound 43.

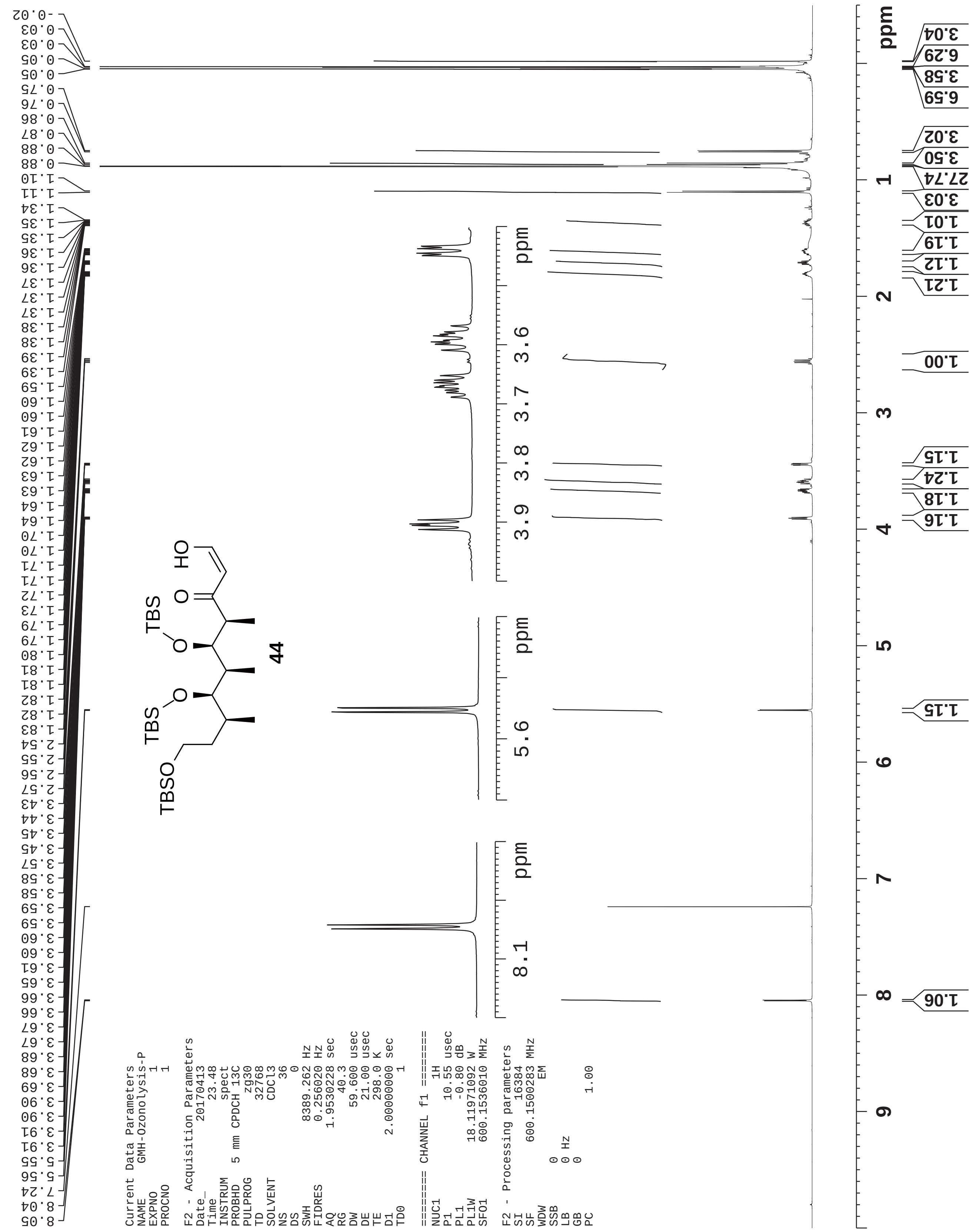

Supplementary Figure 89. ^{1}H NMR spectrum of compound 44.

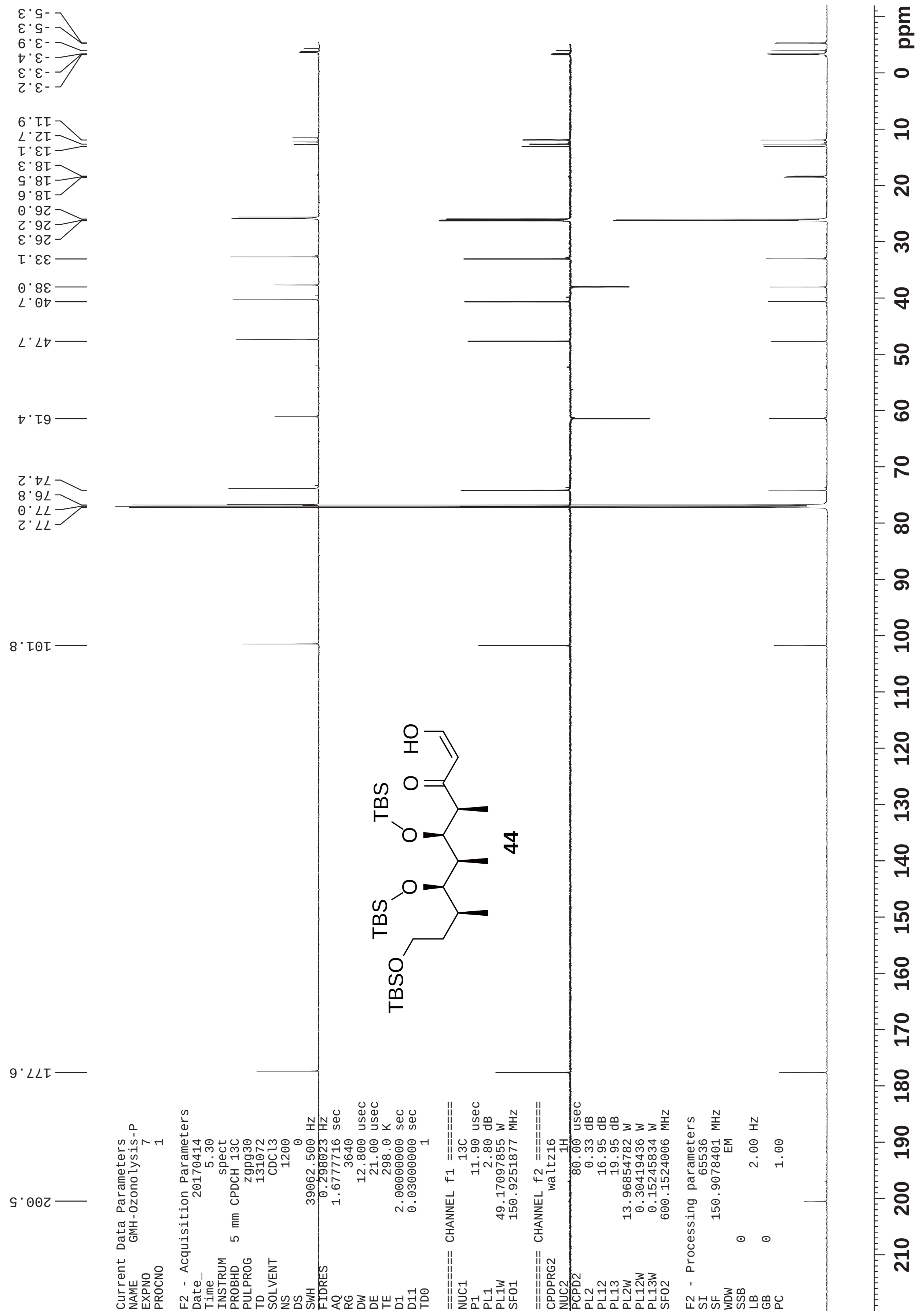

Supplementary Figure 90. ^{13}C and DEPT NMR spectra of compound 44.

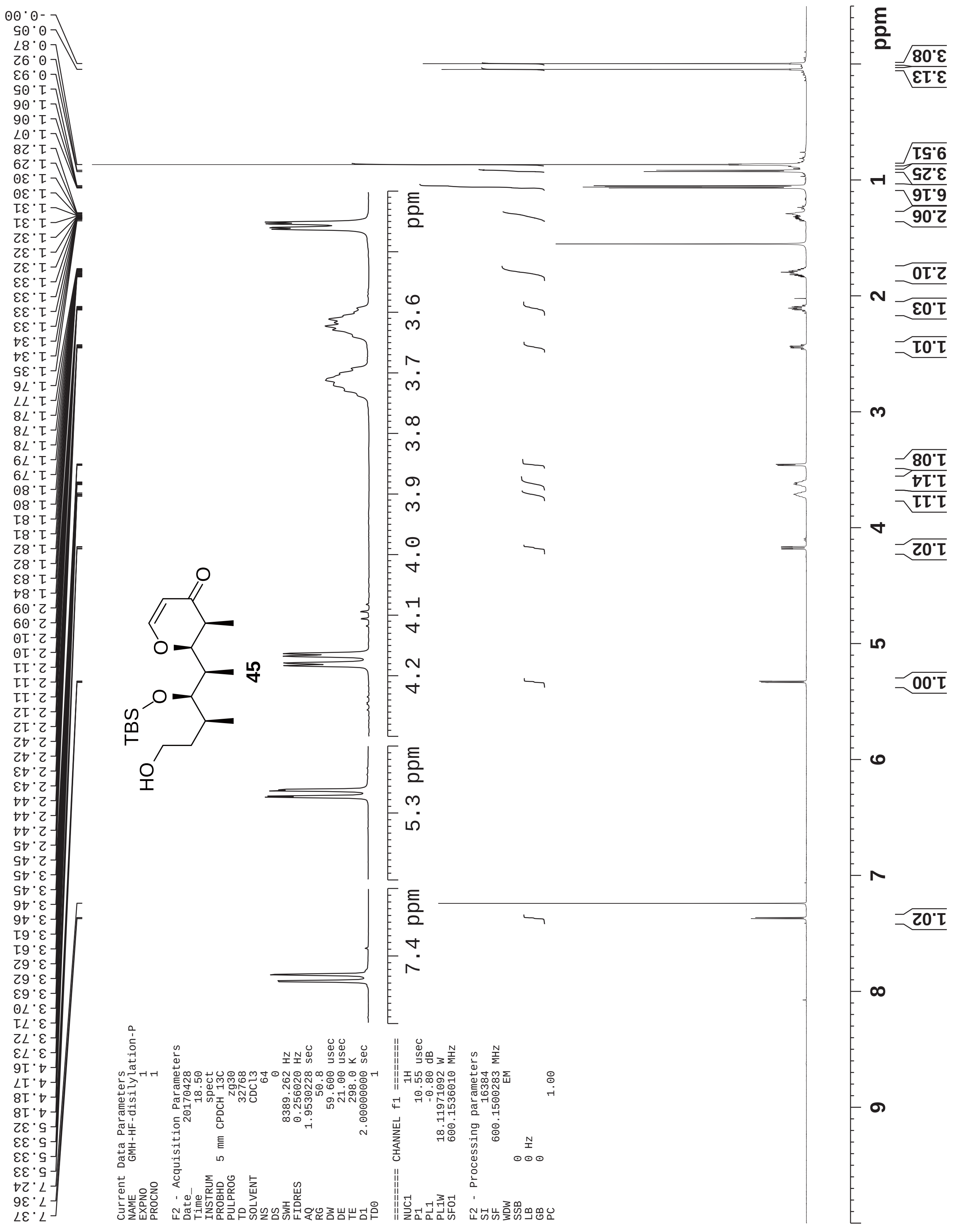

Supplementary Figure 91. ¹H NMR spectrum of compound 45.

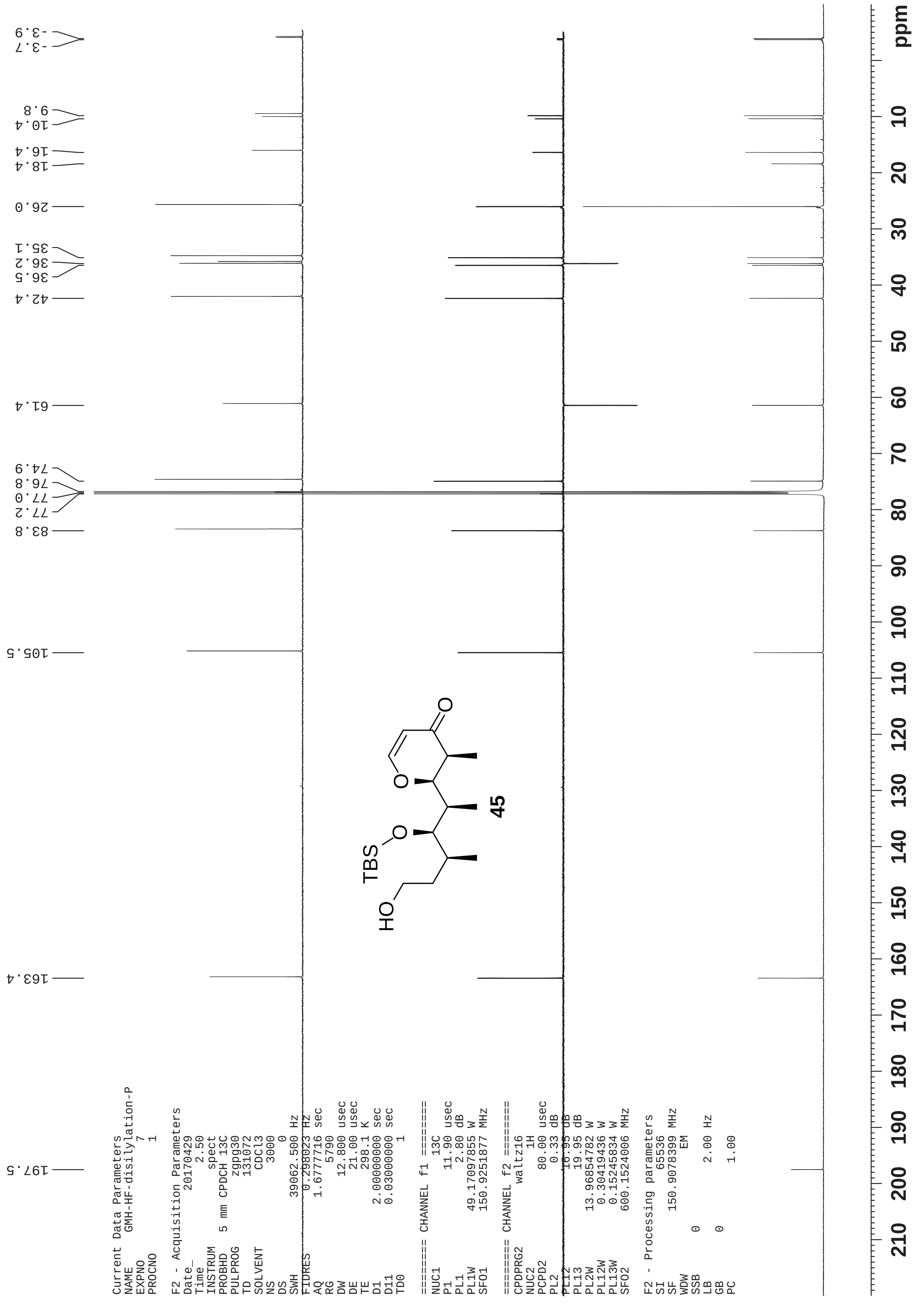

Supplementary Figure 92. ^{13}C and DEPT NMR spectra of compound 45.

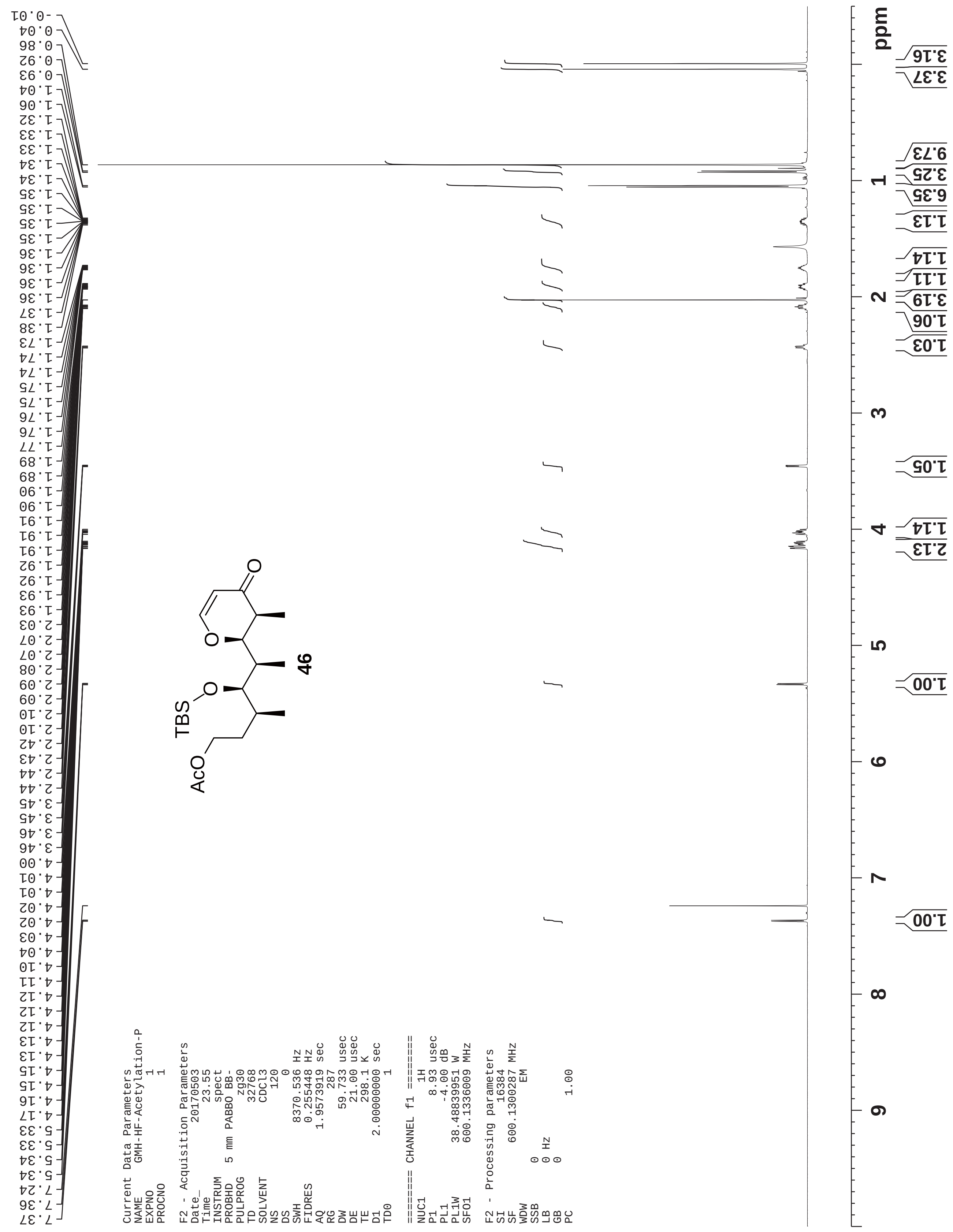

Supplementary Figure 93. ^{1}H NMR spectrum of compound 46.

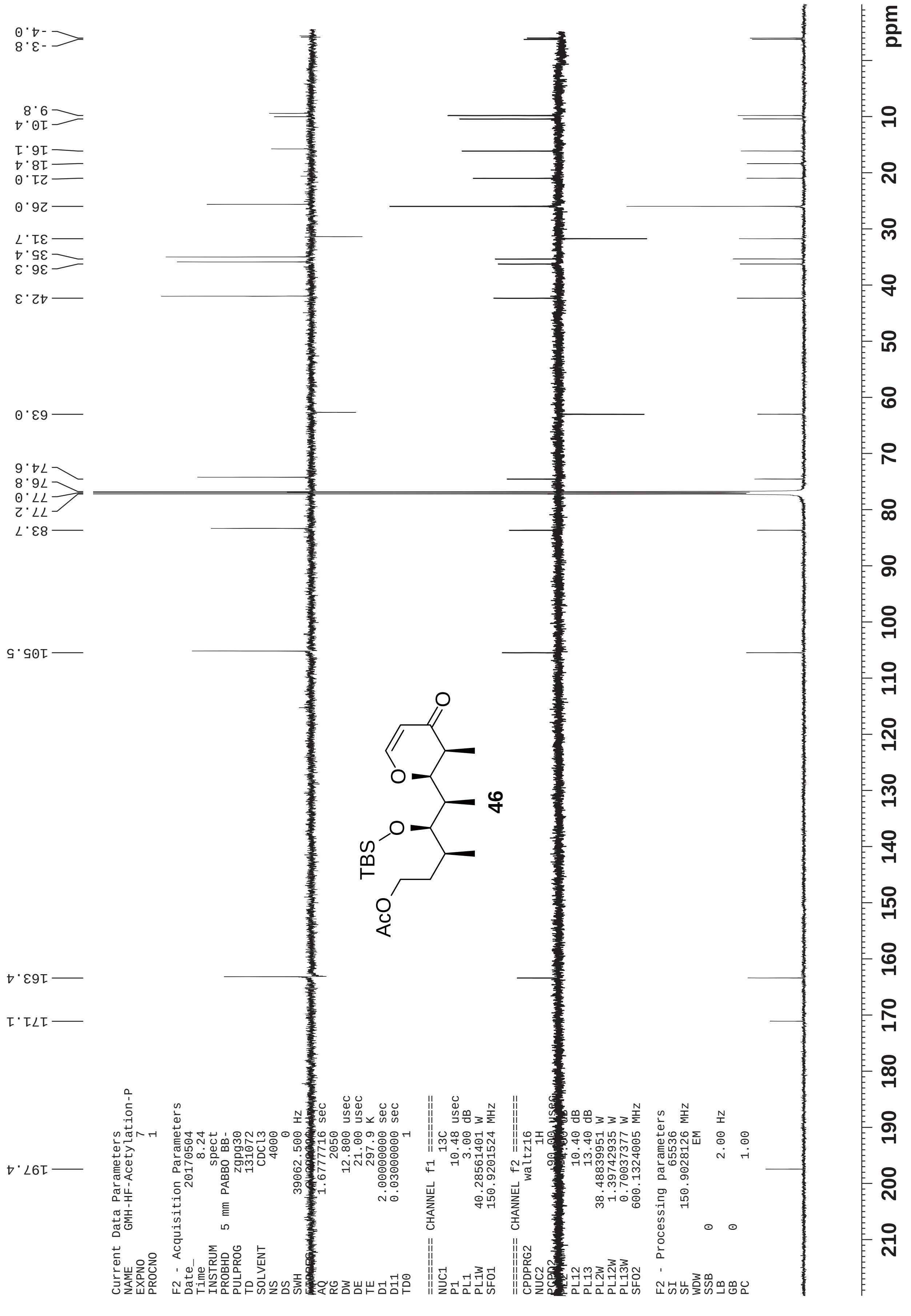

Supplementary Figure 94. ^{13}C and DEPT NMR spectra of compound 46.

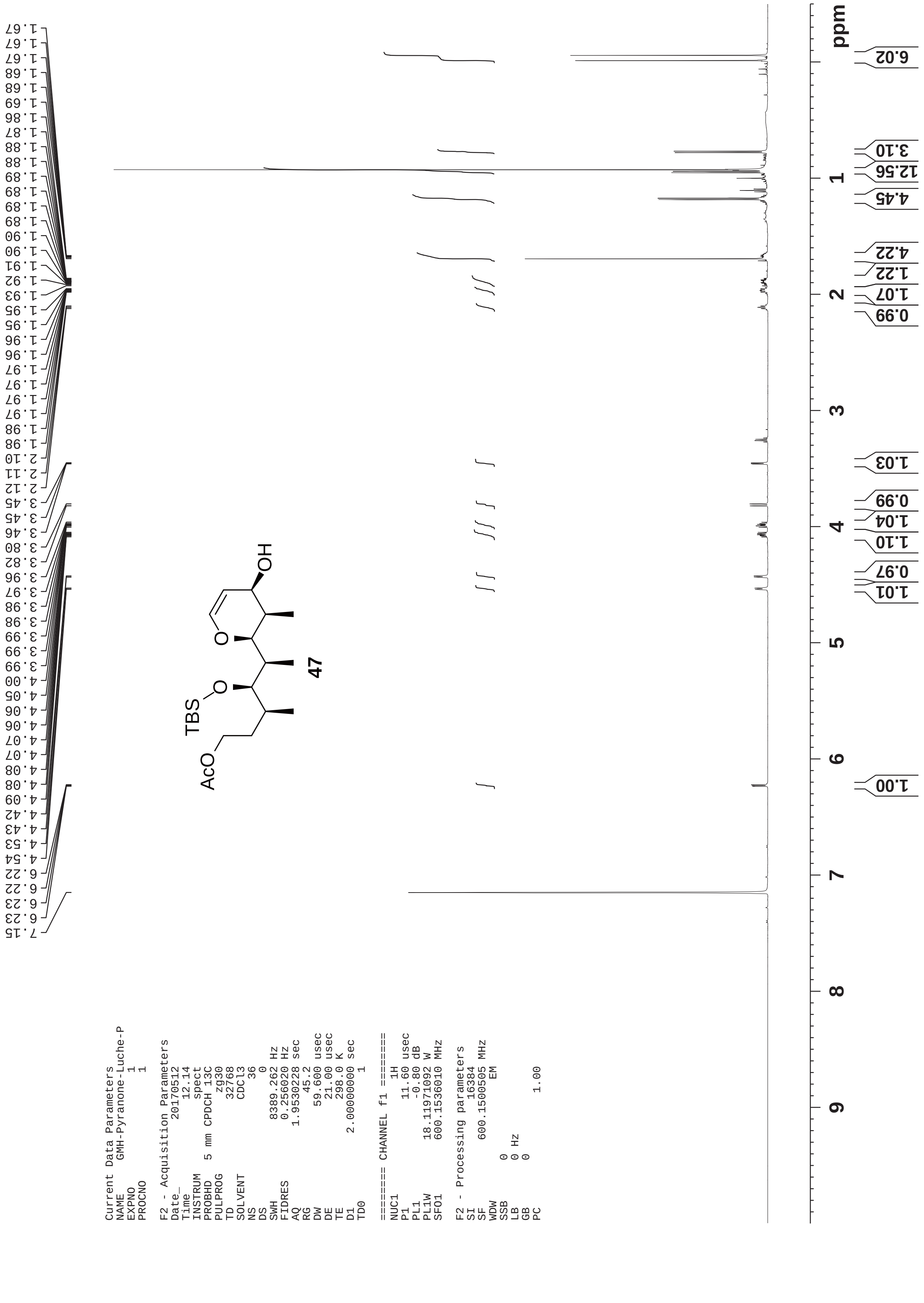

Supplementary Figure 95. ^{1}H NMR spectrum of compound 47.

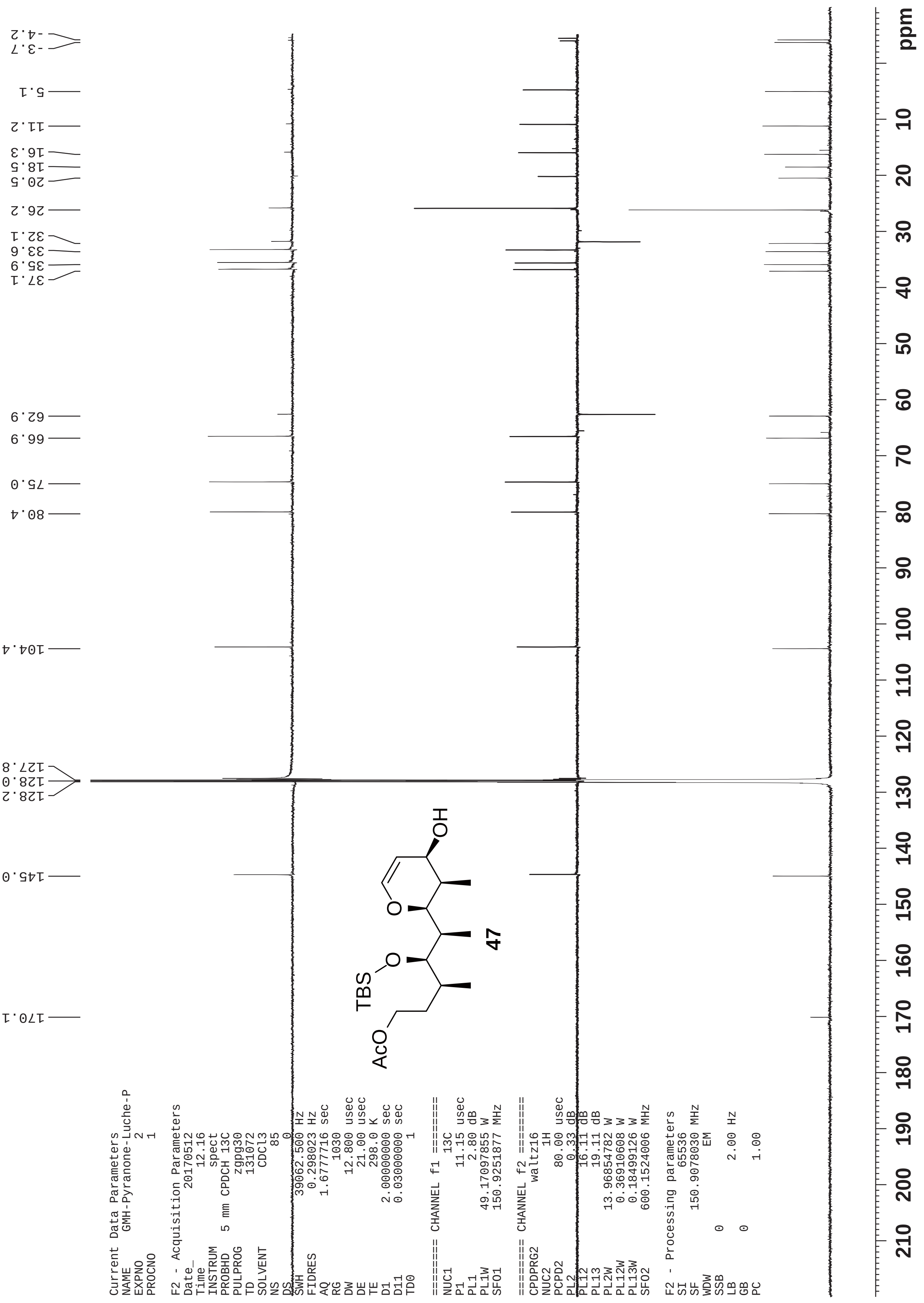

Supplementary Figure 96. ^{13}C and DEPT NMR spectra of compound 47.

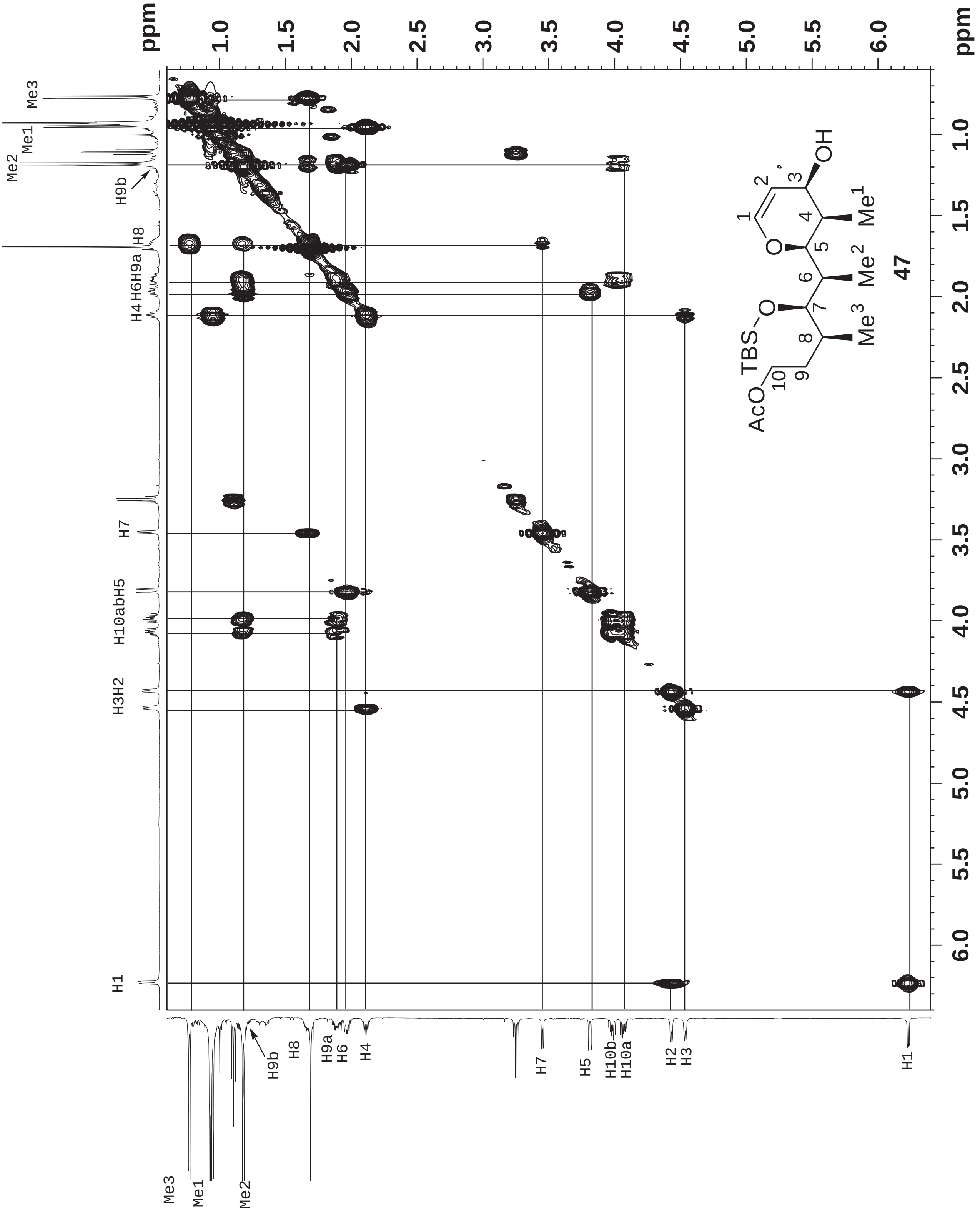

Supplementary Figure 97. COSY spectrum of compound 47.

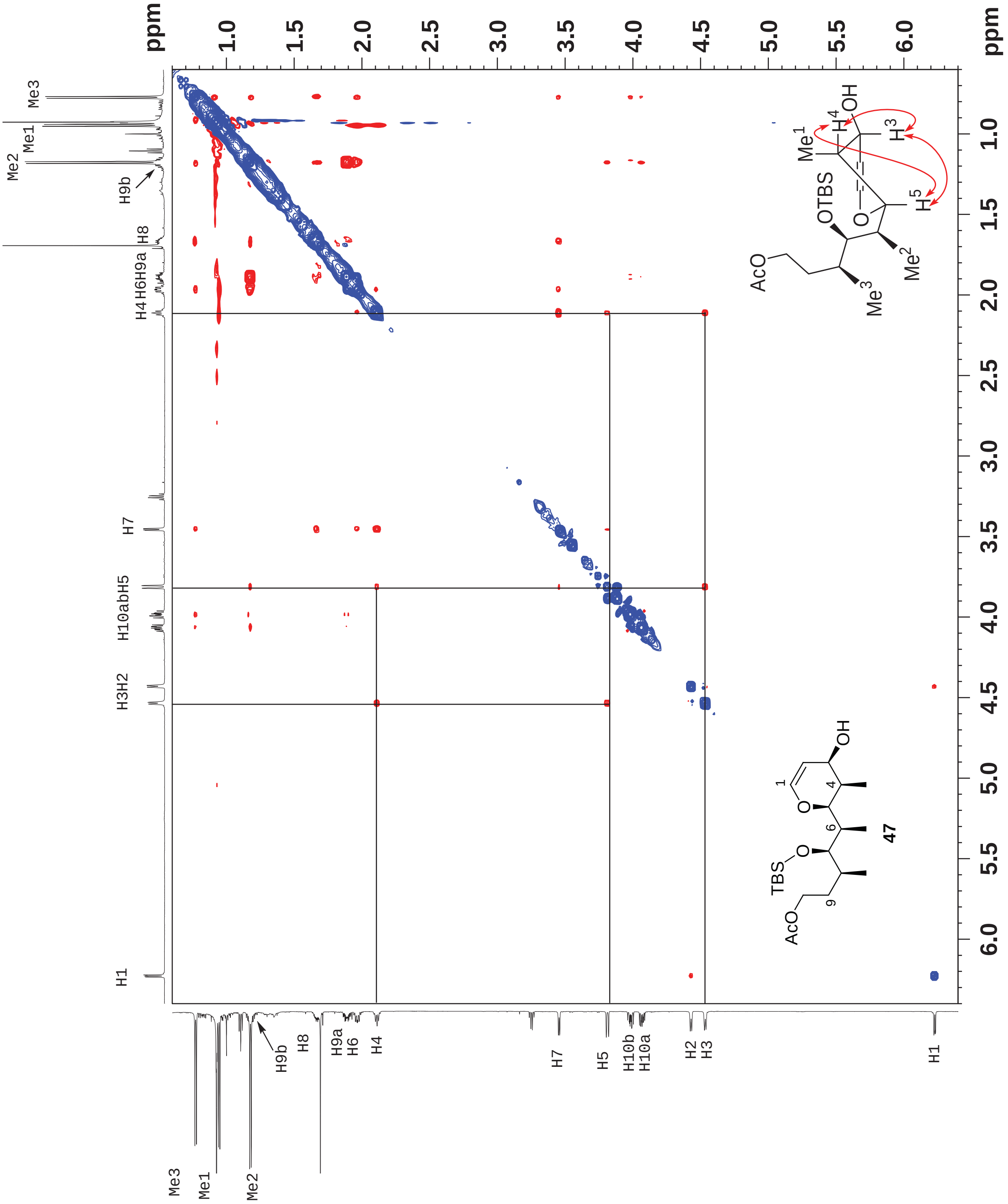

Supplementary Figure 98. NOESY spectrum of compound 47.

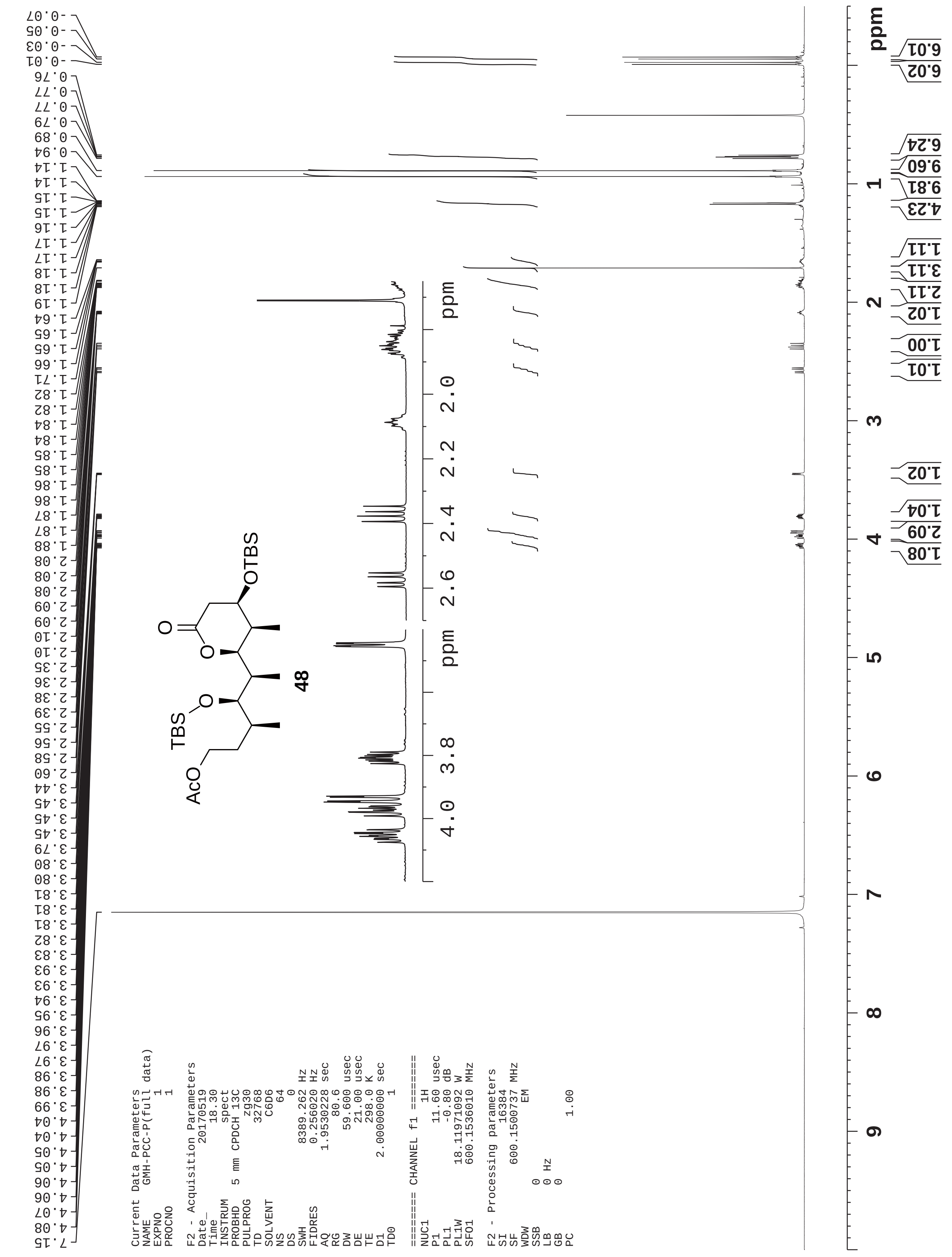

Supplementary Figure 99. ^{1}H NMR spectrum of compound 48.

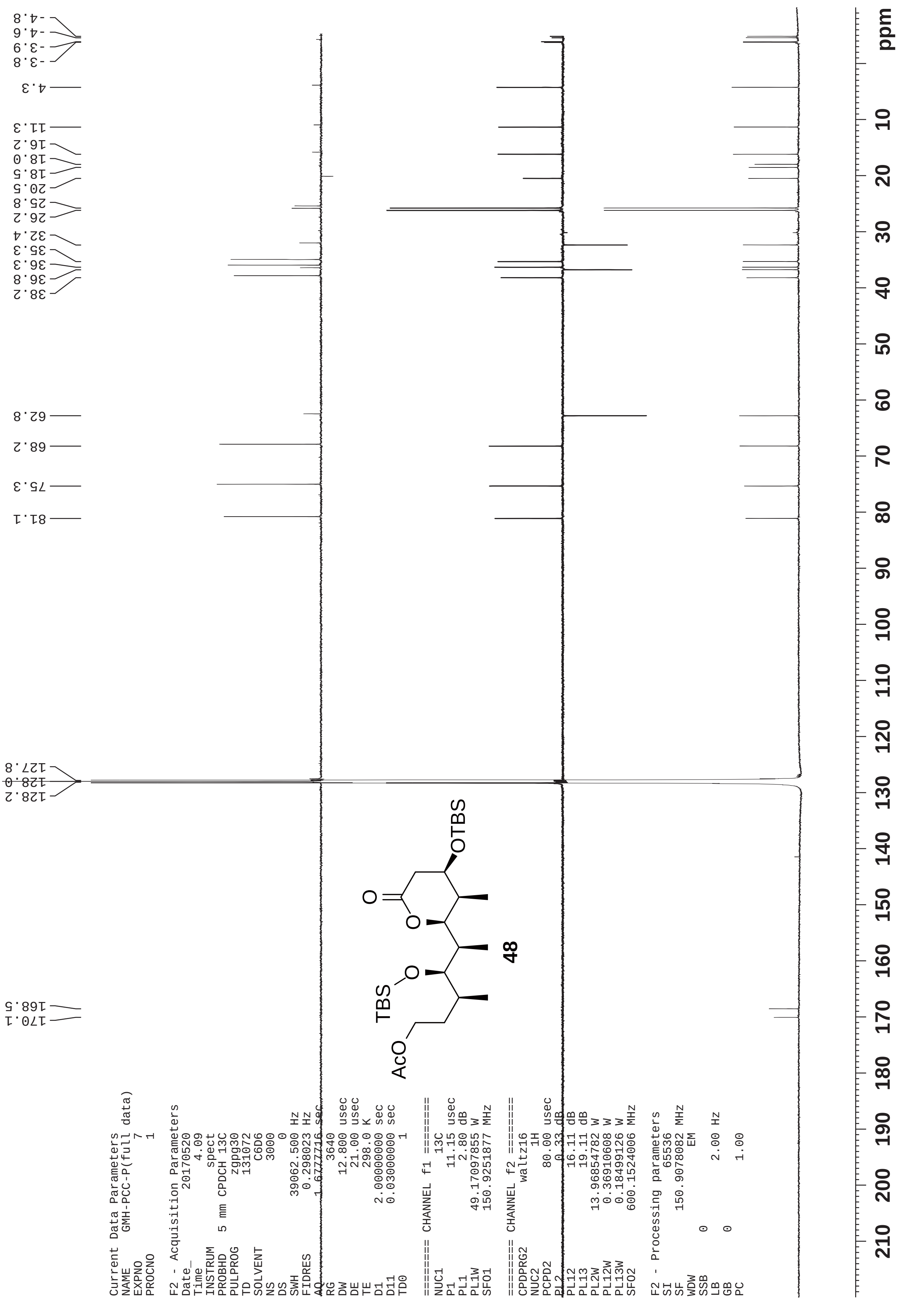

Supplementary Figure 100. ^{13}C and DEPT NMR spectra of compound 48.

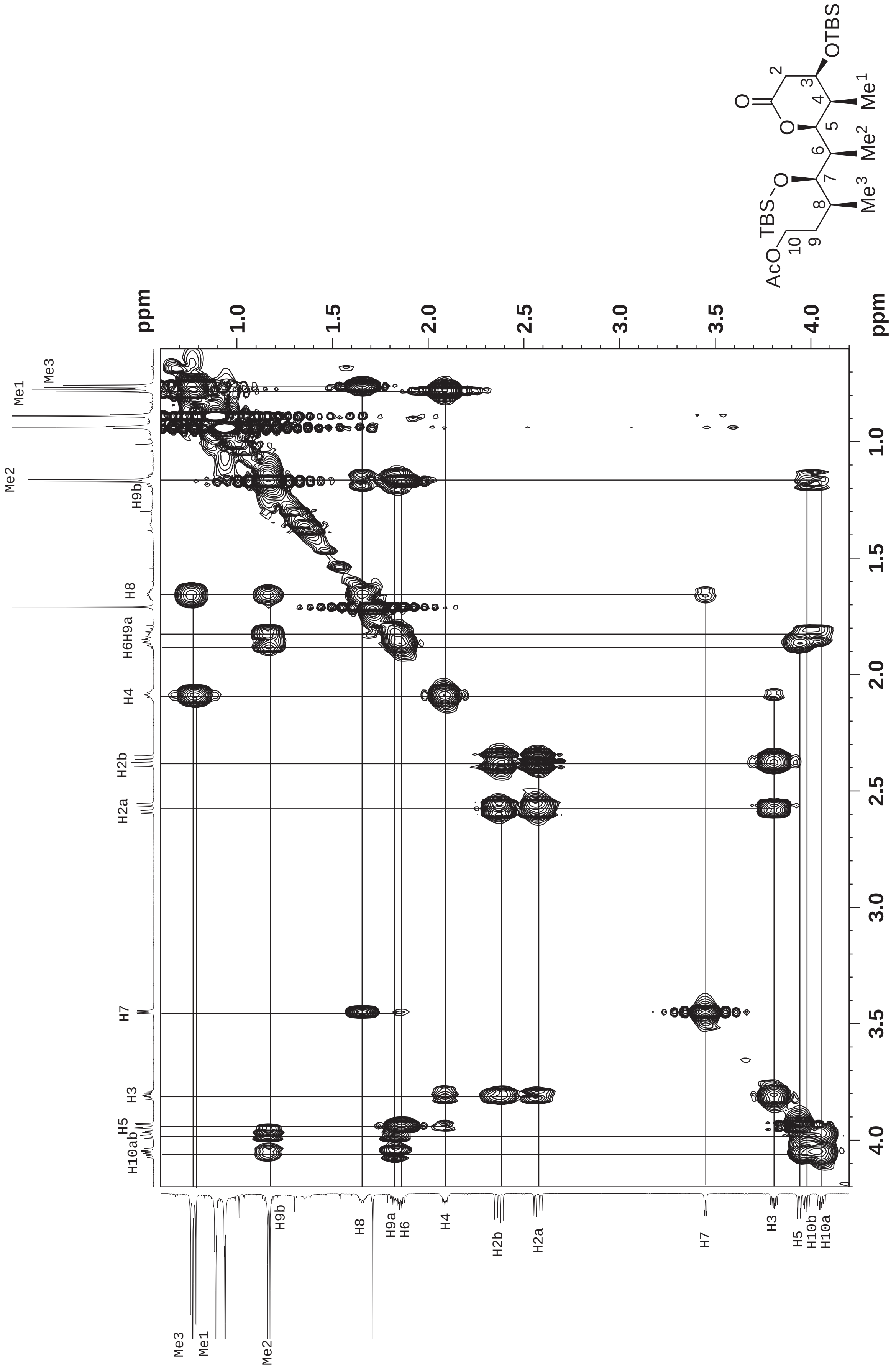

Supplementary Figure 101. COSY spectrum of compound 48.

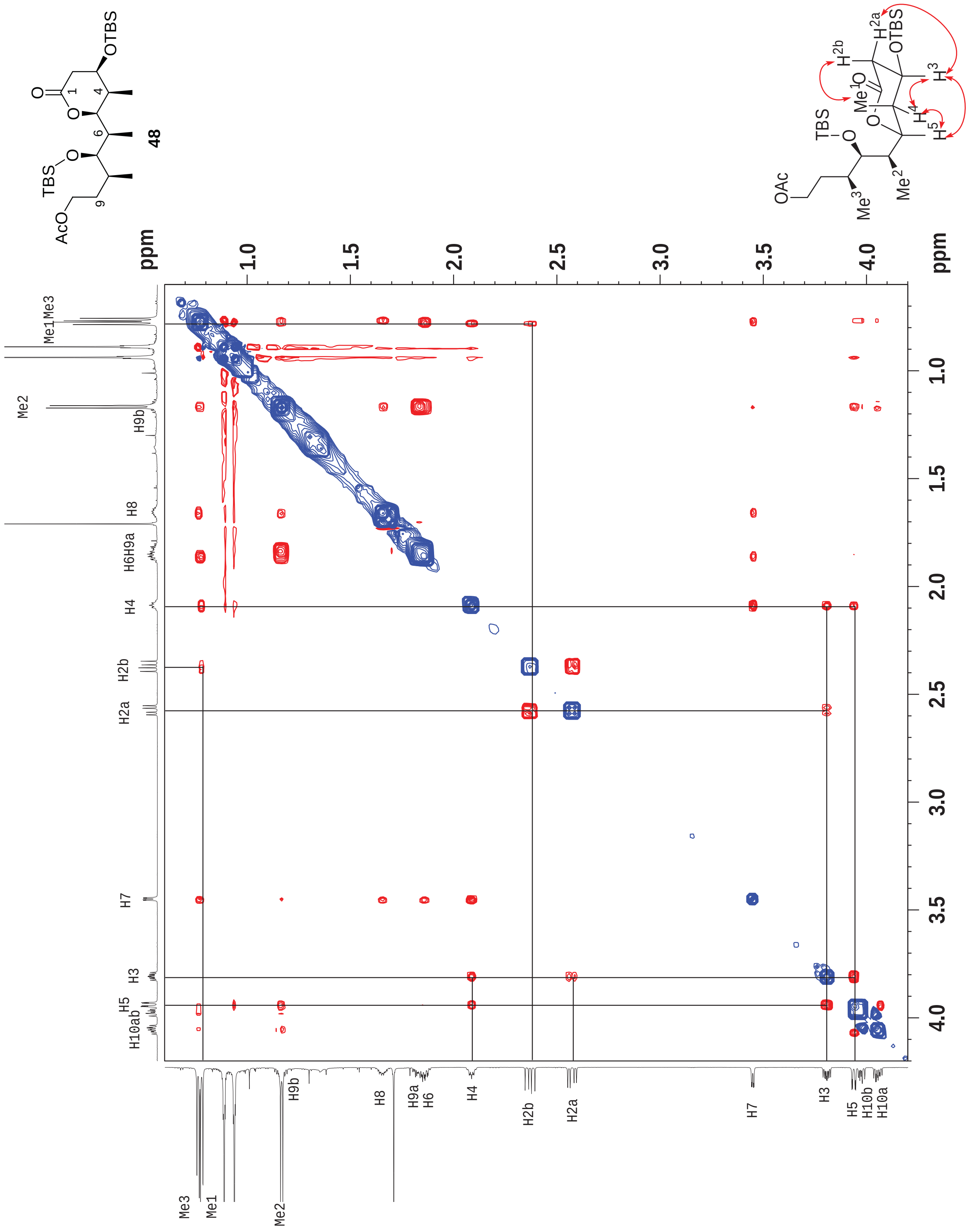

Supplementary Figure 102. NOESY spectrum of compound 48.

Supplementary Table 1. Evaluation of Lewis acids for Diels–Alder cycloaddition

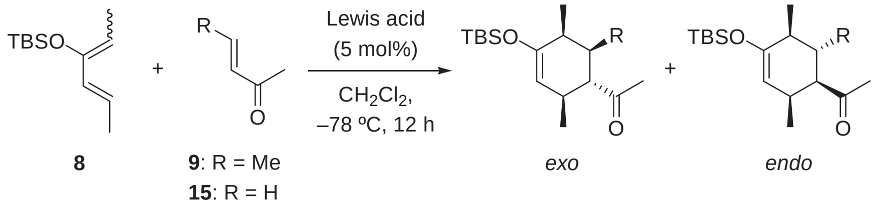

Entry	Enone	Lewis acid	Product	Yield (%)[a]	*exo*/*endo*[b]
1	**9**	$Cu(OTf)_2$	**10**	85	95/5
2	**9**	TMSOTf	**10**	63	95/5
3	**9**	Me_2AlCl	**10**	42	94/6
4	**9**	$SnCl_4$	**10**	52	>95/5
5	**9**	$Sc(OTf)_3$	**10**	79	>95/5
6	**9**	$BF_3 \cdot Et_2O$	**10**	90	95/5
7	**15**	$Cu(OTf)_2$	**S1**	92	<5/95
8	**15**	TMSOTf	**S1**	60	<5/95
9	**15**	Me_2AlCl	**S1**	34	13/87
10	**15**	$SnCl_4$	**S1**	32	14/86
11	**15**	$Sc(OTf)_3$	**S1**	37	46/54
12	**15**	$BF_3 \cdot Et_2O$	**S1**	52	14/86

[a]Based on product isolated after chromatographic separation. [b]Determined by ^{1}H NMR analysis of crude product mixtures.

Supplementary Table 2. Crystal data and structure refinement for compound 11 (CCDC 1511846)

Identification code	i13226	
Empirical formula	$C_{11}H_{18}O_4$	
Formula weight	214.25	
Temperature	100.0(1) K	
Wavelength	0.71073 Å	
Crystal system	Triclinic	
Space group	P -1	
Unit cell dimensions	a = 7.008 Å	α = 106.04°
	b = 7.156 Å	β = 97.62°
	c = 11.975 Å	γ = 99.67°
Volume	558.7 $Å^3$	
Z	2	
Density (calculated)	1.274 Mg/m^3	
Absorption coefficient	0.096 mm^{-1}	
F(000)	232	
Crystal size	0.3 × 0.24 × 0.2 mm^3	
Theta range for data collection	3.00 to 29.47°	
Index ranges	–9<=h<=9, –9<=k<=9, –14<=l<=16	
Reflections collected	5294	
Independent reflections	2643 [R(int) = 0.0179]	
Completeness to theta = 25.00°	99.90%	
Absorption correction	Semi-empirical from equivalents	
Max. and min. transmission	1 and 0.9784	
Refinement method	Full-matrix least-squares on F^2	
Data / restraints / parameters	2643 / 0 / 209	
Goodness-of-fit on F^2	1.042	
Final R indices [I>2sigma(I)]	R1 = 0.0362, wR2 = 0.0920	
R indices (all data)	R1 = 0.0421, wR2 = 0.0961	
Extinction coefficient	0.009(5)	
Largest diff. peak and hole	0.356 and –0.210 e.$Å^{-3}$	

Supplementary Table 3. Crystal data and structure refinement for compound 16 (CCDC 1511847)

Identification code	07jl73_0m	
Empirical formula	$C_{10}H_{16}O_4$	
Formula weight	200.23	
Temperature	296(2) K	
Wavelength	0.71073 Å	
Crystal system	Triclinic	
Space group	P-1	
Unit cell dimensions	a = 7.6701(2) Å	α = 111.724(2)°
	b = 8.0763(2) Å	β = 96.807(2)°
	c = 9.5222(3) Å	γ = 98.512(2)°
Volume	532.20(3) $Å^3$	
Z	2	
Density (calculated)	1.249 Mg/m^3	
Absorption coefficient	0.096 mm^{-1}	
F(000)	216	
Crystal size	0.30 × 0.22 × 0.05 mm^3	
Theta range for data collection	2.34 to 28.98°	
Index ranges	–10<=h<=10, –10<=k<=10, –12<=l<=12	
Reflections collected	12605	
Independent reflections	2798 [R(int) = 0.0920]	
Completeness to theta = 28.98°	99.50%	
Absorption correction	Empirical	
Max. and min. transmission	0.94961 and 0.79654	
Refinement method	Full-matrix least-squares on F^2	
Data / restraints / parameters	2798 / 0 / 130	
Goodness-of-fit on F^2	0.945	
Final R indices [I>2sigma(I)]	R1 = 0.0470, wR2 = 0.1328	
R indices (all data)	R1 = 0.0712, wR2 = 0.1538	
Largest diff. peak and hole	0.241 and –0.226 e.$Å^{-3}$	

Supplementary Table 4. Crystal data and structure refinement for compound 23 (CCDC 1511848)

Identification code	i13839	
Empirical formula	$C_{16}H_{20}O_4$	
Formula weight	276.32	
Temperature	100.0(1) K	
Wavelength	0.71073 Å	
Crystal system	Monoclinic	
Space group	P2(1)/n	
Unit cell dimensions	a = 8.00500(10) Å	α = 90°.
	b = 11.0252(2) Å	β = 97.0200(10)°
	c = 16.3524(3) Å	γ = 90°
Volume	1432.39(4) $Å^3$	
Z	4	
Density (calculated)	1.281 Mg/m^3	
Absorption coefficient	0.091 mm^{-1}	
F(000)	592	
Crystal size	0.32 x 0.28 x 0.18 mm3	
Theta range for data collection	2.23 to 27.10°.	
Index ranges	-10<=h<=10, -14<=k<=12, -20<=l<=20	
Reflections collected	11892	
Independent reflections	3157 [R(int) = 0.0229]	
Completeness to theta = 25.00°	99.90%	
Absorption correction	Semi-empirical from equivalents	
Max. and min. transmission	0.9838 and 0.9714	
Refinement method	Full-matrix least-squares on F^2	
Data / restraints / parameters	3157 / 0 / 185	
Goodness-of-fit on F^2	1.054	
Final R indices [I>2sigma(I)]	R1 = 0.0353, wR2 = 0.0816	
R indices (all data)	R1 = 0.0440, wR2 = 0.0865	
Extinction coefficient	0.0073(15)	
Largest diff. peak and hole	0.365 and –0.189 e.$Å^{-3}$	

Supplementary Table 5. Crystal data and structure refinement for compound 29 (CCDC 1511849)

Identification code	i15013	
Empirical formula	$C_{10}H_{16}O_4$	
Formula weight	200.23	
Temperature	100.0(2) K	
Wavelength	0.71073 Å	
Crystal system	Monoclinic	
Space group	P 21/c	
Unit cell dimensions	a = 6.1819(2) Å	α = 90°
	b = 19.6642(6) Å	β = 104.247(2)°
	c = 8.7180(3) Å	γ = 90°
Volume	1027.18(6) $Å^3$	
Z	4	
Density (calculated)	1.295 Mg/m^3	
Absorption coefficient	0.099 mm^{-1}	
F(000)	432	
Crystal size	0.320 × 0.280 × 0.140 mm^3	
Theta range for data collection	2.071 to 26.372°	
Index ranges	–7<=h<=7, –24<=k<=24, –10<=l<=10	
Reflections collected	25806	
Independent reflections	2100 [R(int) = 0.0282]	
Completeness to theta = 25.000°	99.90%	
Absorption correction	Semi-empirical from equivalents	
Max. and min. transmission	0.9705 and 0.9365	
Refinement method	Full-matrix least-squares on F^2	
Data / restraints / parameters	2100 / 33 / 131	
Goodness-of-fit on F^2	1.052	
Final R indices [I>2sigma(I)]	R1 = 0.0321, wR2 = 0.0769	
R indices (all data)	R1 = 0.0343, wR2 = 0.0785	
Extinction coefficient	0.004(2)	
Largest diff. peak and hole	0.372 and –0.202 e.$Å^{-3}$	

Supplementary Table 6. Crystal data and structure refinement for compound 34 (CCDC 1511880)

Identification code	i13165	
Empirical formula	$C_{15}H_{18}O_4$	
Formula weight	262.29	
Temperature	100.0(1) K	
Wavelength	0.71073 Å	
Crystal system	Orthorhombic	
Space group	P2(1)2(1)2(1)	
Unit cell dimensions	a = 8.574 Å	α = 90°
	b = 8.800 Å	β = 90°
	c = 17.704 Å	γ = 90°
Volume	1335.8 $Å^3$	
Z	4	
Density (calculated)	1.304 Mg/m^3	
Absorption coefficient	0.094 mm^{-1}	
F(000)	560	
Crystal size	0.28 × 0.24 × 0.16 mm^3	
Theta range for data collection	2.30 to 26.36°	
Index ranges	–10<=h<=10, –10<=k<=10, –22<=l<=19	
Reflections collected	36941	
Independent reflections	2722 [R(int) = 0.0515]	
Completeness to theta = 25.00°	100.00%	
Absorption correction	Semi-empirical from equivalents	
Max. and min. transmission	0.9703 and 0.91	
Refinement method	Full-matrix least-squares on F^2	
Data / restraints / parameters	2722 / 0 / 245	
Goodness-of-fit on F^2	1.066	
Final R indices [I>2sigma(I)]	R1 = 0.0257, wR2 = 0.0632	
R indices (all data)	R1 = 0.0273, wR2 = 0.0641	
Absolute structure parameter	–0.1(7)	
Extinction coefficient	0.0044(15)	
Largest diff. peak and hole	0.190 and –0.141 e.$Å^{-3}$	

Supplementary Table 7. Deacetylation and translactonization of acetylated ε- and γ-lactone

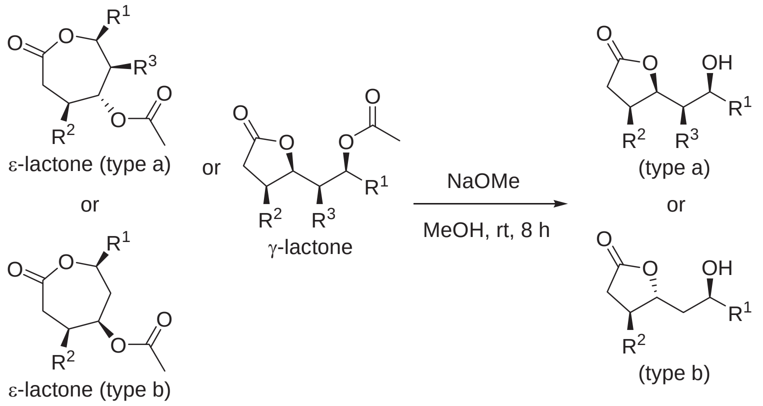

Entry	Starting lactone	Product	Yield (%)[a]
1	**16**	**S2** (type b: $R^1 = R^2 = Me$)	96
2	**18**	**S3** (type b: $R^1 = (CH_2)_2OBn$; $R^2 = Me$)	94
3	**19**	**S4** (type a: $R^1 = (CH_2)_2OBn$; $R^2 = R^3 = Me$)	94
4	**21**	**S5** (type a: $R^1 = CH_2Ph$; $R^2 = R^3 = Me$)	96
5	**23**	**S6** (type a: $R^1 = Ph$; $R^2 = R^3 = Me$)	95
6	**25**	**S7** (type a: $R^1 = CH_2\textit{i}Pr$; $R^2 = R^3 = Me$)	95
7	**27**	**S8** (type a: $R^1 = CH_2\textit{i}Bu$; $R^2 = R^3 = Me$)	94
8	**29**	**S9** (type a: $R^1 = R^3 = Me$; $R^2 = H$)	97
9	**31**	**S10** (type a: $R^1 = (CH_2)_2OBn$; $R^2 = H$; $R^3 = Me$)	93
10	**33**	**S11** (type a: $R^1 = Me$; $R^2 = H$; $R^3 = Ph$)	95
11	**34**	**S11**	97
12	**35**	**S12** (type a: $R^1 = (CH_2)_2OBn$; $R^2 = H$; $R^3 = Ph$)	95
13	**36**	**S12**	95

[a]Based on product isolated after chromatographic separation.

Supplementary Table 8. Crystal data and structure refinement for compound 40 (CCDC 1511850)

Identification code	i13946	
Empirical formula	$C_{24}H_{38}O_2Si$	
Formula weight	386.63	
Temperature	100.0(1) K	
Wavelength	0.71073 Å	
Crystal system	Triclinic	
Space group	P-1	
Unit cell dimensions	a = 7.4396(3) Å	α = 67.380(3)°
	b = 11.7668(5) Å	β = 84.506(3)°
	c = 14.4083(6) Å	γ = 83.796(3)°
Volume	1155.44(8) $Å^3$	
Z	2	
Density (calculated)	1.111 Mg/m^3	
Absorption coefficient	0.117 mm^{-1}	
F(000)	424	
Crystal size	0.24 x 0.14 x 0.10 mm^3	
Theta range for data collection	1.53 to 27.10°	
Index ranges	–7<=h<=9, –15<=k<=15, –18<=l<=18	
Reflections collected	22999	
Independent reflections	5096 [R(int) = 0.0655]	
Completeness to theta = 25.00°	100.00%	
Absorption correction	Semi-empirical from equivalents	
Max. and min. transmission	0.9884 and 0.9725	
Refinement method	Full-matrix least-squares on F^2	
Data / restraints / parameters	5096 / 0 / 253	
Goodness-of-fit on F^2	1.016	
Final R indices [I>2sigma(I)]	R1 = 0.0422, wR2 = 0.0928	
R indices (all data)	R1 = 0.0637, wR2 = 0.1044	
Largest diff. peak and hole	0.371 and –0.240 e.$Å^{-3}$	

Supplementary Table 9. Crystal data and structure refinement for compound 41 (CCDC 1556026)

Identification code	i16636	
Empirical formula	$C_{18}H_{24}O_4$	
Formula weight	304.37	
Temperature	100.0(2) K	
Wavelength	0.71073 Å	
Crystal system	Monoclinic	
Space group	P 21	
Unit cell dimensions	a = 8.8720(3) Å	a= 90°.
	b = 11.0082(3) Å	b= 108.429(2)°.
	c = 9.1544(3) Å	g = 90°.
Volume	848.21(5) $Å^3$	
Z	2	
Density (calculated)	1.192 Mg/m^3	
Absorption coefficient	0.083 mm^{-1}	
F(000)	328	
Crystal size	0.360 × 0.280 × 0.240 mm^3	
Theta range for data collection	2.345 to 27.100°	
Index ranges	–11<=h<=11, –14<=k<=14, –11<=l<=11	
Reflections collected	29015	
Independent reflections	3748 [R(int) = 0.0464]	
Completeness to theta = 25.000°	100.00%	
Absorption correction	Semi-empirical from equivalents	
Max. and min. transmission	0.9705 and 0.9001	
Refinement method	Full-matrix least-squares on F^2	
Data / restraints / parameters	3748 / 1 / 204	
Goodness-of-fit on F^2	1.05	
Final R indices [I>2sigma(I)]	R1 = 0.0305, wR2 = 0.0683	
R indices (all data)	R1 = 0.0350, wR2 = 0.0710	
Absolute structure parameter	0.2(3)	
Extinction coefficient	0.018(3)	
Largest diff. peak and hole	0.183 and –0.146 e.$Å^{-3}$	

Supplementary Table 10. Crystal data and structure refinement for compound 42 (CCDC 1556034)

Identification code	i16662	
Empirical formula	$C_{16}H_{22}O_3$	
Formula weight	262.33	
Temperature	100.0(2) K	
Wavelength	0.71073 Å	
Crystal system	Orthorhombic	
Space group	P 21 21 21	
Unit cell dimensions	a = 7.0558(2) Å	α = 90°
	b = 11.7298(3) Å	β = 90°
	c = 17.7125(5) Å	γ = 90°
Volume	1465.94(7) Å^3	
Z	4	
Density (calculated)	1.189 Mg/m^3	
Absorption coefficient	0.081 mm^{-1}	
F(000)	568	
Crystal size	0.440 × 0.360 × 0.340 mm^3	
Theta range for data collection	2.082 to 27.086°.	
Index ranges	–9<=h<=9, –15<=k<=15, –22<=l<=22	
Reflections collected	59569	
Independent reflections	3229 [R(int) = 0.0421]	
Completeness to theta = 25.000°	100.00%	
Absorption correction	Semi-empirical from equivalents	
Max. and min. transmission	0.9705 and 0.897	
Refinement method	Full-matrix least-squares on F^2	
Data / restraints / parameters	3229 / 0 / 178	
Goodness-of-fit on F^2	1.047	
Final R indices [I>2sigma(I)]	R1 = 0.0298, wR2 = 0.0729	
R indices (all data)	R1 = 0.0331, wR2 = 0.0755	
Absolute structure parameter	0.0(2)	
Extinction coefficient	0.0067(18)	
Largest diff. peak and hole	0.190 and –0.137 e.Å^{-3}	

Supplementary Methods

General methods

CH_2Cl_2 were purified and dried from a safe purification system by passing through activated Al_2O_3 under argon pressure. Tetrahydrofuran (THF) was distilled from sodium benzophenone ketyl prior to use. All other reagents were obtained from commercial sources and used without further purification. Flash column chromatography was carried out on Silica Gel 60 (230–400 mesh, E. Merck). TLC was performed on glass plates precoated with Silica Gel 60 F254 (0.25 mm, E. Merck); visualization was performed under ultraviolet light, followed by dipping in 15% phosphomolybdic acid in ethanol and subsequent heating on a hot plate. Specific rotations were measured on a HORIBA SEPA-300 high sensitive polarimeter at 589 nm employing a 10 mm cell at ambient conditions and reported in $10^{-1}\cdot deg\cdot cm^2\cdot g^{-1}$; the sample concentrations are in $g\cdot dL^{-1}$. Melting points were determined using Büchi melting point B-540 apparatus. The IR spectra were recorded using liquid films on KBr plates or solutions in a Perkin-Elmer Spectrum 100 FT-IR spectrometer. ^1H and ^{13}C NMR spectra were recorded on Bruker AMX400 (400 MHz) and Bruker AVANCE-600 (600 MHz) instruments. Chemical shifts are in ppm referenced relative to residual proton and carbon signals of the solvent. Proton peak assignments were performed using two-dimensional NMR techniques (^1H-^1H COSY, HMQC and NOESY). The hydrogen multiplicities of carbon peaks were determined using DEPT-90 and DEPT-135 experiments, the spectra of which were herein provided together with the power-gated-decoupled ^{13}C NMR spectrum. Mass spectra were obtained with a FAB JMS-700 double focusing mass spectrometer, Waters LCT Premier XE equipped with a dual ionization ESCi® (ESI/APCi) source options and ESI Finnigan LCQ mass spectrometer. X-ray diffractions were obtained using Bruker X8APEX single-crystal X-ray diffractometer. The X-ray structures were solved and refined employing the Bruker SHELXTL software package. The twinning crystal data were refined by choosing the corresponding Flack parameter of desired enantiomer in a two-component inversion twin procedure.

Synthetic methods and characterization data

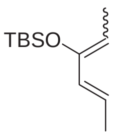

8

(2*Z*,4*E*)/(2*E*,4*E*)-3-(*tert*-butyldimethylsilyloxy)-2,4-hexadiene (8). Lithium hexamethyldisilazide (LiHMDS, 37 mL of a 1.0 M solution in THF, 37 mmol) was slowly added to a solution of 4-hexen-3-one (**7**, 92%, 3.80 mL, 30.6 mmol) in anhydrous THF (102 mL) at –78 °C under N_2 atmosphere. After 30 min of stirring, *tert*-butyldimethylsilyl trifluoromethanesulfonate (TBSOTf, 98%, 7.9 mL, 34 mmol) was added dropwise. The reaction was allowed to warm to room temperature and stirred for 6 h. The reaction was quenched with satd. $NaHCO_{3(aq)}$, followed by extraction with Et_2O. The combined organic layer was dried over anhydrous $MgSO_4$, filtered and concentrated under reduced pressure. Purification of the crude product by flash column chromatography (*n*-hexane/Et_3N = 95/5) provided the silyloxydiene **8** (5.78 g, 89%, (*Z,E*)/(*E,E*) = 86/14). The physical data of the (*Z,E*) and (*E,E*) isomers of **8** have been reported in the literature[1].

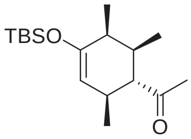

10

***rac*-1-[(1*R*,2*S*,5*S*,6*R*)/(1*S*,2*R*,5*R*,6*S*)-4-*tert*-butyldimethylsilyloxy-2,5,6-trimethyl-cyclohex-3-enyl]ethanone (10).** Freshly distilled enone **9** (165 µL, 1.70 mmol) was added to a stirring solution of $BF_3 \cdot Et_2O$ (8.7 µL, 71 µmol) in CH_2Cl_2 (2 mL) at –78 °C. After stirring at –78 °C for 10 min, diene **8** [(*Z,E*)/(*E,E*) = 86/14, 300 mg, 1.42 mmol (based on the (*Z,E*) isomer)] in CH_2Cl_2 (0.8 mL) was added dropwise and the resulting solution was stirred at the same temperature for 12 h. The reaction was quenched with Et_3N and satd. $NaHCO_{3(aq)}$. The crude organic portion was extracted with CH_2Cl_2, dried over anhydrous $MgSO_4$, filtered and concentrated under reduced pressure. The residue was purified by flash column chromatography (*n*-hexane/ethyl acetate = 20/1) to give the Diels-Alder cycloadduct **10** as well as the minor *endo*-adduct isomer (total: 0.320 g, 90%, *exo*/*endo* = 95/5). Other Lewis acids (5 mol%) were also used to facilitate the reaction as described in Supplementary Table 1. IR (thin film): ν 2958, 2930, 2859, 1710, 1667, 1463, 1358, 1255, 1056, 837, 779 cm^{-1}; HRMS (APCI): *m/z* calcd for $C_{17}H_{33}O_2Si$ ($[M + H]^+$): 297.2250, found: 297.2249. For the *exo*-adduct: ^1H NMR (600 MHz, $CDCl_3$): δ 4.55 (d, *J* = 2.1 Hz, 1H; H3), 2.48–2.47 (m, 1H; H2) , 2.12 (s, 3H; $COCH_3$), 2.07–2.04 (m, 2H; H1, H4), 2.00–1.97 (m, 1H; H5), 0.93 (d, *J* = 7.0 Hz, 3H; CH_3), 0.90 (s, 9H; Si-$C(CH_3)_3$), 0.86 (d, *J* = 6.8 Hz, 3H; CH_3), 0.78 (d, *J* = 6.3 Hz, 3H; CH_3) , 0.11 (s, 6H; Si-$(CH_3)_2$); ^{13}C NMR (150 MHz, $CDCl_3$): δ 213.9 (C), 154.1 (C), 107.6 (CH), 57.1 (CH), 38.9 (CH), 34.9 (CH), 33.4 (CH), 30.5 (CH_3), 25.6 ($CH_3 \times 3$), 21.0

(CH_3), 18.0 (C), 16.1 (CH_3), 12.9 (CH_3), –4.4 (CH_3), –4.6 (CH_3); For the *endo*-adduct: ^{1}H NMR (600 MHz, $CDCl_3$): δ 4.87 (dd, *J* = 6.2, 1.3 Hz, 1H; H3), 2.54–2.50 (m, 1H; H2) , 2.12 (s, 3H; $COCH_3$), 2.06–2.04 (m, 2H; H1, H4), 1.99–1.98 (m, 1H; H5), 1.07 (d, *J* = 7.0 Hz, 3H; CH_3), 0.90 (s, 9H; Si-C(CH_3)$_3$), 0.87 (d, *J* = 6.8 Hz, 3H; CH_3), 0.76 (d, *J* = 6.3 Hz, 3H; CH_3) , 0.11 (s, 6H; Si-(CH_3)$_2$); ^{13}C NMR (150 MHz, $CDCl_3$): δ 210.9 (C), 153.1 (C), 108.0 (CH), 57.5 (CH), 41.2 (CH), 40.5 (CH), 33.0 (CH), 30.3 (CH_3), 25.6 (CH_3 × 3), 22.7 (CH_3), 18.6 (CH_3), 18.0 (C), 17.0 (CH_3), –3.7 (CH_3), –3.9 (CH_3).

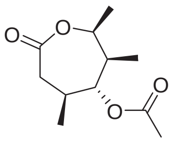

11

***rac*-[(2*S*,3*R*,4*R*,5*S*)/(2*R*,3*S*,4*S*,5*R*)-2,3,5-trimethyl-7-oxooxepan-4-yl acetate (11).** *Method A*: To a solution of the *exo*-cycloadduct **10** (299 mg, 1.01 mmol) in CH_2Cl_2 (10 mL) was added trifluoroacetic acid (TFA, 85 µL, 1.1 mmol) dropwise at 0 °C. After stirring for 20 min, *meta*-chloroperoxybenzoic acid (*m*CPBA, 77%, 1.36 g, 6.08 mmol) was added in one portion. The resultant mixture was allowed to warm to ambient temperature and was stirred for 24 h. After this time, the reaction mixture was slowly quenched with satd. $Na_2S_2O_{3(aq)}$ at 0°C, and the crude organic portion was extracted with CH_2Cl_2. The combined organic layer was washed with satd. $NaHCO_{3(aq)}$ and brine, dried over $MgSO_4$, filtered and concentrated under reduced pressure. Purification via flash column chromatography (*n*-hexane/ethyl acetate = 6:1) provided the lactone **11** (0.162, 75%) as a colourless solid. *Method B*: Freshly distilled enone **9** (0.33 mL, 3.40 mmol) was added by syringe to a stirring solution of $BF_3·Et_2O$ (18 µL, 0.15 mmol) in CH_2Cl_2 (2 mL) at –78 °C. After stirring at –78 °C for 10 min, diene **9** [(*Z,E*)/(*E,E*) = 86/14, 600 mg, 2.83 mmol (based in the (*Z*,*E*) isomer)] in CH_2Cl_2 (6.0 mL) was added dropwise, and the reaction was allowed to stir for 12 h at the same temperature. TFA (240 µL, 3.11 mmol) was next added in one portion, and the reaction was allowed to warm to 0 °C. After 20 min at 0 °C, the resulting mixture was poured into a solution of *m*CPBA (77%, 5.0 g, 22.6 mmol) in CH_2Cl_2 (22 mL) at 0 °C and stirred at ambient temperature for 24 h. The reaction mixture was slowly quenched with satd. $Na_2S_2O_{3(aq)}$ at 0 °C, and the crude organic portion was extracted with CH_2Cl_2. The combined organic layer was washed with satd. $NaHCO_{3(aq)}$ and brine, dried over $MgSO_4$, filtered and concentrated under reduced pressure. Purification by flash column chromatography (*n*-hexane/ethyl acetate = 6/1) gave the ε-lactone **11** (0.33 g, 63%) as a colourless solid. m.p. 88–90 °C

(recrystallized from *n*-hexane/$CHCl_3$); IR (thin film): ν 2980, 2942, 1780, 1733, 1455, 1374, 1244, 1184, 1022 cm^{-1}; ^1H NMR (600 MHz, $CDCl_3$): δ 4.91 (q, *J* = 6.5 Hz, 1H; ε-methine-H), 4.85 (t, *J* = 3.0 Hz, 1H; γ-methine-H), 3.24 (dd, *J* = 14.0, 3.0 Hz, 1H; α-methylene-H), 2.40 (dd, *J* = 13.8, 5.0 Hz, 1H; α-methylene-H), 2.19–2.16 (m, 1H; β-methine-H), 2.06 (s, 3H; CO_2CH_3), 1.99–1.95 (m, 1H; δ-methine-H), 1.32 (d, *J* = 6.5 Hz, 3H; CH_3), 1.14 (d, *J* = 7.7 Hz, 3H; CH_3), 1.09 (d, *J* = 7.6 Hz, 3H; CH_3); ^{13}C NMR (150 MHz, $CDCl_3$): δ 173.5 (C), 169.6 (C), 78.0 (CH), 72.5 (CH), 42.2 (CH), 35.2 (CH_2), 32.5 (CH), 21.3 (CH_3), 19.5 (CH_3), 16.8 (CH_3), 10.1 (CH_3); HRMS (FAB): *m/z* calcd for $C_{11}H_{19}O_4$ ($[M + H]^+$): 215.1283, found 215.1282. An X-ray analysis of the crystal confirmed the structure of **11** (Fig. 3b, CCDC 1511846).

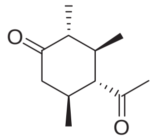

12

***rac*-(2*R*,3*R*,4*R*,5*S*)/(2*R*,3*R*,4*R*,5*S*)-4-acetyl-2,3,5-trimethylcyclohexanone (12).** To a solution of **10** (300 mg, 1.01 mmol) in THF (10 mL) at 0 °C was added tetrabutylammonium fluoride (1.0 M in THF, 1.2 mL, 1.2 mmol). The reaction mixture allowed to warm to ambient temperature and was stirred for 8 h. The reaction was quenched with satd.$NH_4Cl_{(aq)}$, extracted with ethyl acetate, dried over anhydrous $MgSO_4$, filtered and concentrated. The residue was purified by flash column chromatography (*n*-hexane/ethyl acetate = 4/1) to give **12** (142 mg, 77 %) as a colourless oil. IR (thin film): ν 2969, 1709, 1455, 1377, 1105, 760, 701 cm^{-1}; ^1H NMR (600 MHz, $CDCl_3$): δ 2.40 (dd, *J* = 12.9, 3.6 Hz, 1H), 2.32 (t, *J* = 10.8 Hz, 1H), 2.15 (s, 3H), 2.12–2.02 (m, 3H), 1.73–1.68 (m, 1H), 1.03 (d, *J* = 6.5 Hz, 3H), 0.96 (d, *J* = 6.5 Hz, 3H), 0.93 (d, *J* = 6.3 Hz, 3H); ^{13}C NMR (150 MHz, $CDCl_3$): δ 211.9 (C), 210.1 (C), 64.9 (CH), 49.7 (CH), 48.6 (CH_2), 41.8 (CH), 35.8 (CH_3), 30.5 (CH), 20.3 (CH_3), 18.2 (CH_3), 11.3 (CH_3); HRMS (ESI): *m/z* calcd for $C_{11}H_{18}O_2Na$ ($[M + Na]^+$): 205.1204, found: 205.1202.

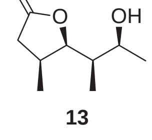

13

***rac*-(4*S*,5*R*)/(4*R*,5*S*)-dihydro-5-[(2*R*,3*S*)/(2*S*,3*R*)-3-hydroxybutan-2-yl]-4-methylfuran-2(3*H*)-one (13).** To a solution of ε-lactone **11** (100 mg, 0.467 mmol) in

methanol was added NaOMe (28 mg, 0.51 mmol) at 0 °C. The resulting mixture was allowed to warm up to room temperature. Upon consumption of the starting material as indicated by TLC analysis (about 8 h), the reaction was neutralized with DOWEX 50 WX2-200 IR resin. The mixture was filtered, and the filtrate was concentrated under reduced pressure. Purification of the residue by flash column chromatography (*n*-hexane/ethyl acetate = 1/2) gave the γ-lactone **13** (77 mg, 96%) as a colourless oil. m.p. 61–62 °C (recrystallized from *n*-hexane/$CHCl_3$); IR (thin film): ν 2975, 2925, 1768, 1736, 1460, 1386, 1221, 1185, 1158, 1018, 927, 935, 885 cm^{-1}; ^1H NMR (600 MHz, $CDCl_3$): δ 4.45 (dd, *J* = 8.8, 5.0 Hz, 1H; γ-methine-H), 3.90 (qd, *J* = 6.4, 2.1 Hz, 1H; ε-methine-H), 2.72 (dd, *J* = 16.6, 7.5 Hz, 1H; α-methylene-H), 2.70–2.66 (m, 1H; β-methine-H), 2.20 (dd, *J* = 16.6, 1.9 Hz, 1H; α-methylene-H), 1.82–1.77 (m, 1H; δ-methine-H), 1.58 (br, 1H; OH), 1.22 (d, *J* = 6.4 Hz, 3H; CH_3), 1.04 (d, *J* = 6.8 Hz, 3H; CH_3), 1.00 (d, *J* = 6.9 Hz, 3H; CH_3); ^{13}C NMR (150 MHz, $CDCl_3$): δ 177.1 (C), 85.4 (CH), 67.1 (CH), 38.8 (CH), 38.6 (CH_2), 32.4 (CH), 21.1 (CH_3), 14.0 (CH_3), 9.3 (CH_3); HRMS (APCI): *m/z* calcd for $C_9H_{16}O_3Na$ ($[M + Na]^+$): 195.0997, found: 195.0993.

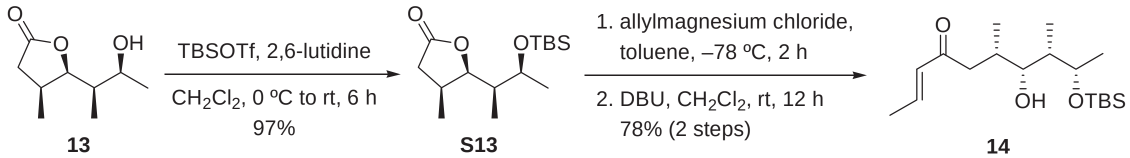

13 **S13** **14**

***rac*-5-[(2*R*,3*S*)/(2*S*,3*R*)-3-*tert*-butyldimethylsilyloxybutan-2-yl)-(4*S*,5*R*)/(4*R*,5*S*)-dihydro-4-methylfuran-2(3*H*)-one (S13).** To a solution of alcohol **13** (0.500 g, 2.91 mmol) in anhydrous CH_2Cl_2 (6.0 mL) was added 2,6-lutidine (0.500 mL, 4.36 mmol) under N_2 atmosphere. The stirred mixture was cooled to 0 °C and *tert*-butyldimethylsilyl triflate (TBSOTf, 98%, 0.75 mL, 3.20 mmol) was added dropwise. Once addition of TBSOTf was complete, the mixture was allowed to warm to room temperature and stirred further for 6 h. The reaction was quenched with satd. $NaHCO_{3(aq)}$. The crude organic portion was extracted with CH_2Cl_2, dried over anhydrous $MgSO_4$, filtered and concentrated under reduced pressure. The residue was purified by flash column chromatography (*n*-hexane/ethyl acetate = 4/1) to afford the silyl ether **S13** (0.81 g, 97%) as a colourless oil. IR (thin film): ν 2957, 2931, 1776, 1636, 1472, 1463, 1383, 1254, 1185, 1154, 1030, 931, 836 cm^{-1}; ^1H NMR (600 MHz, $CDCl_3$): δ 4.38 (dd, *J* = 8.9, 5.0 Hz, 1H; γ-methine-H), 3.85 (qd, *J* = 6.3, 2.3 Hz, 1H; ε-methine-H), 2.69 (dd, *J* = 16.9, 7.5 Hz, 1H; α-methylene-H), 2.60–2.55 (m, 1H, β-methine-H), 2.18 (dd, *J* = 16.9, 1.8 Hz, 1H; α-methylene-H), 1.79–1.74 (m, 1H, δ-methine-H), 1.13 (d, *J* = 6.3 Hz, 3H; CH_3), 1.02 (d, *J* = 6.7 Hz, 3H; CH_3), 0.98 (d, *J* = 7.1 Hz, 3H; CH_3), 0.85 (s, 9H;

Si-C(CH_3)$_3$), 0.04 (s, 3H; Si-CH_3), 0.01 (s, 3H; Si-CH_3); ^{13}C NMR (150 MHz, $CDCl_3$): δ 176.9 (C), 84.9 (CH), 67.8 (CH), 39.7 (CH), 38.8 (CH_2), 32.5 (CH), 25.7 (CH_3 × 3), 20.9 (CH_3), 18.0 (C), 14.2 (CH_3), 10.2 (CH_3), –3.9 (CH_3), –4.0 (CH_3); HRMS (ESI): *m/z* calcd for $C_{15}H_{30}O_3NaSi$ ([M + H]$^+$): 309.1862, found: 309.1860.

***rac*-(*E*,6*S*,7*R*,8*S*,9*S*)/(*E*,6*R*,7*S*,8*R*,9*R*)-9-*tert*-butyldimethylsilyloxy-7-hydroxy-6,8-dimethyldec-2-en-4-one (14).** The solution of compound **S13** (600 mg, 2.10 mmol) in dry toluene (10.5 mL) was cooled to –78 °C. With vigorous stirring, allylmagnesium chloride (2.0 M solution in THF, 1.1 mL, 2.2 mmol) was added dropwise using a microliter syringe for about 5 min. The reaction was stirred under argon for 2 h at –78 °C. The reaction mixture was quenched with satd. $NH_4Cl_{(aq)}$, followed by extraction with ethyl acetate. The combined organic layer was washed with brine, dried over $MgSO_4$, filtered and concentrated under reduced pressure. The residue was then diluted with CH_2Cl_2 (10.5 mL), and to this mixture was added 1,8-diazabicyclo[5.4.0]undec-7-ene (DBU, 0.160 mL, 1.15 mmol). After stirring for 8 h at room temperature, the reaction mixture was quenched with satd. $NH_4Cl_{(aq)}$ (20 mL). The whole mixture was transferred to a separatory funnel, and the organic layer was separated with further extraction with CH_2Cl_2. The combined organic extract was washed with brine, dried over $MgSO_4$, filtered and concentrated under reduced pressure. The crude material was purified by column chromatography (*n*-hexane/ethyl acetate = 7/1) to give compound **14** (537 mg, 78%) as a colourless oil. IR (thin film): ν 2958, 2930, 2857, 1697, 1669, 1630, 1471, 1462, 1376, 1254, 1160, 1083, 1031, 962, 775 cm^{-1}; ^{1}H NMR (600 MHz, $CDCl_3$): δ 6.82 (dq, *J* = 15.6 6.9 Hz, 1H; β-vinylic H), 6.10 (dd, *J* = 15.6, 1.5 Hz, 1H; α-vinylic H), 3.97 (qd, *J* = 6.3, 2.9 Hz, 1H; ε-methine-H), 3.47 (ddd, *J* = 6.3, 3.1, 2.2 Hz, 1H; γ-methine-H), 2.65 (d, *J* = 3.1 Hz, 1H; γ-OH), 2.53 (dd, *J* = 15.4, 4.7 Hz, 1H; α-methylene-H), 2.34 (dd, *J* = 15.4, 8.6 Hz, 1H; α-methylene-H), 2.21 (dddd, *J* = 13.3, 8.6, 4.7, 2.2 Hz, 1H; β-methine-H), 1.86 (dd, *J* = 6.9, 1.5 Hz, 3H; vinylic CH_3), 1.52–1.50 (m, 1H; δ-methine-H), 1.12 (d, *J* = 6.3 Hz, 3H; CH_3), 0.92 (d, *J* = 6.7 Hz, 3H; CH_3), 0.90 (d, *J* = 6.9 Hz, 3H; CH_3), 0.85 (s, 9H; Si-C(CH_3)$_3$), 0.05 (s, 3H, Si-CH_3), 0.04 (s, 3H, Si-CH_3); ^{13}C NMR (150 MHz, $CDCl_3$): δ 200.0 (C), 142.7 (CH), 132.2 (CH), 77.9 (CH), 72.8 (CH), 44.3 (CH_2), 41.4 (CH), 33.1 (CH), 25.8 (CH_3 × 3), 21.6 (CH_3), 18.2 (C), 17.9 (CH_3), 15.4 (CH_3), 7.0 (CH_3), –3.8 (CH_3), –4.9 (CH_3); HRMS (ESI): *m/z* calcd for $C_{18}H_{36}O_3NaSi$ ([M + Na]$^+$): 351.2331, found: 351.2336.

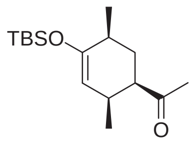

S1

***rac*-1-[(1*R*,2*S*,5*S*)/(1*S*,2*R*,5*R*)-4-*tert*-butyldimethylsilyloxy-2,5-dimethylcyclohex-3-enyl)ethanone (S1).** Freshly distilled methylvinyl ketone (**15**, 170 μL, 2.12 mmol) was added by syringe to a stirring mixture of Lewis acid (see Supplementary Table 1, 71 μmol) in CH_2Cl_2 (2 mL) at –78 °C under argon atmosphere. After stirring at –78 °C for 10 min, diene **8** [(*Z,E*)/(*E,E*) = 86/14, 300 mg, 1.42 mmol (based on the (*Z,E*) isomer)] in CH_2Cl_2 (0.8 mL) was added dropwise, and the resulting mixture was stirred at this temperature for 12 h. The reaction was quenched with Et_3N and satd. $NaHCO_{3(aq)}$. The crude organic portion was extracted with CH_2Cl_2, dried over anhydrous $MgSO_4$, filtered and concentrated under reduced pressure. The residue was purified by flash column chromatography (*n*-hexane/ethyl acetate = 20/1) to give the *endo*-cycloadduct **S1** as well as its minor *exo*-adduct isomer (see Supplementary Table 1) as colourless oils. For the *endo*-adduct **S1**: IR (thin film): ν 3407, 2958, 2931, 2859, 1716, 1658, 1463, 1370, 1257, 1173, 841, 778 cm^{-1}; ^{1}H NMR (400 MHz, C_6D_6): δ 4.78 (dd, *J* = 5.7, 1.8 Hz, 1H; H3), 2.42 (m, 1H, H2) , 2.29 (ddd, *J* = 12.8, 5.3, 2.6 Hz, 1H; H1), 2.04 (m, 1H; H4), 1.76 (dddd, *J* = 13.2, 5.9, 2.6, 1.1 Hz, 1H; H6), 1.71 (s, 3H; $COCH_3$), 1.54 (ddd, *J* = 13.2, 12.8, 11.2 Hz, 1H; H5), 1.08 (d, *J* = 6.9 Hz, 3H; CH_3), 0.98 (s, 9H; Si-C$(CH_3)_3$), 0.74 (d, *J* = 6.9 Hz, 3H; CH_3), 0.15 (s, 3H; Si-CH_3), 0.14 (s, 3H; Si-CH_3); ^{13}C NMR (100 MHz, C_6D_6): δ 207.9 (C), 154.3 (C), 108.5 (CH), 51.1 (CH), 34.4 (CH), 30.8 (CH), 28.3 (CH_2), 27.8 (CH_3), 25.9 (CH_3 × 3), 18.8 (CH_3), 18.3 (C), 17.2 (CH_3), –4.3 (CH_3), –4.4 (CH_3); HRMS (APCI): *m/z* calcd for $C_{16}H_{31}O_2Si$ ($[M + H]^+$): 283.2093, found: 283.2098. The relative stereochemistry was determined by NOESY experiment and coupling constants analysis. For the *exo*-adduct: IR (thin film): ν 3406, 2957, 2931, 2860, 1716, 1657, 1465, 1371, 1257, 1173, 843, 776 cm^{-1}; ^{1}H NMR (600 MHz, C_6D_6): δ 4.65 (d, *J* = 2.4 Hz, 1H; H3), 2.69 (dqd, *J* = 8.8, 6.8, 2.4 Hz, 1H; H2), 2.11–2.07 (m, 1H; H4), 2.09 (ddd, *J* = 11.7, 8.8, 2.9 Hz, 1H; H1), 1.76 (s, 3H; $COCH_3$), 1.67 (ddd, *J* = 13.1, 11.7, 5.9 Hz, 1H; H6), 1.33 (ddd, *J* = 13.1, 2.9, 2.4 Hz, 1H; H5), 1.05 (d, *J* = 7.1 Hz, 3H; CH_3), 0.98 (s, 9H; Si-C$(CH_3)_3$), 0.87 (d, *J* = 6.9 Hz, 3H; CH_3), 0.13 (s, 3H; Si-CH_3), 0.11 (s, 3H; Si-CH_3); ^{13}C NMR (150 MHz, C_6D_6): δ 209.2 (C), 153.5 (C), 108.4 (CH), 51.3 (CH), 33.2 (CH), 32.9 (CH_2), 31.4 (CH), 28.3 (CH_3), 25.9 (CH_3 × 3), 21.4 (CH_3), 18.8 (CH_3), 18.2 (C), –4.3 (CH_3), –4.5 (CH_3); ^{1}H NMR (600 MHz, $CDCl_3$): δ 4.56 (d, *J* = 2.2 Hz, 1H; H3), 2.55 (dqd, *J* = 9.1, 7.0, 2.2 Hz, 1H; H2), 2.25 (ddd, *J* = 11.9, 9.1, 2.7 Hz, 1H; H1), 2.17–2.6 (m, 1H; H4), 2.14 (s, 3H; $COCH_3$), 1.79 (ddd,

J = 12.9, 11.9, 5.9 Hz, 1H; H6), 1.59 (ddd, *J* = 12.9, 2.7, 2.3 Hz, 1H; H5), 1.10 (d, *J* = 7.1 Hz, 3H; CH_3), 0.89 (s, 9H; Si-$C(CH_3)_3$), 0.88 (d, *J* = 7.0 Hz, 3H; CH_3), 0.11 (s, 6H; Si-$(CH_3)_2$); ^{13}C NMR (150 MHz, $CDCl_3$): δ 212.2 (C), 153.3 (C), 108.3 (CH), 51.3 (CH), 32.9 (CH_2), 32.8 (CH), 31.3 (CH), 28.8 (CH_3), 25.6 (CH_3 × 3), 21.1 (CH_3), 18.7 (CH_3), 18.0 (C), –4.4 (CH_3), –4.6 (CH_3); HRMS (ESI): *m/z* calcd for $C_{16}H_{31}O_2Si$ ($[M + H]^+$): 283.2093, found: 283.2079. The relative stereochemistry was determined by NOESY experiment and coupling constants analysis.

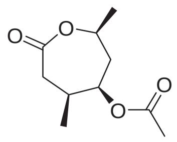

16

***rac*-(2*S*,4*R*,5*S*)/(2*R*,4*S*,5*R*)-2,5-dimethyl-7-oxooxepan-4-yl acetate (16).** Using a procedure similar to *Method B* in the preparation of compound **11**, copper(II) triflate [$Cu(OTf)_2$, 52 mg, 1.4 mmol], freshly distilled methylvinyl ketone (**15**, 0.380 mL, 4.25 mmol) and diene **8** [(*Z,E*)/(*E,E*) = 86/14, 600 mg, 2.83 mmol (based on the (*Z,E*) isomer)] in CH_2Cl_2 (6.0 mL) were allowed to react at –78 °C for 12 h. Then, TFA (0.24 mL, 3.1 mmol) was added in one portion. After 20 min at 0 °C, the whole mixture was poured into a solution of *m*CPBA (5.00 g, 22.6 mmol) in CH_2Cl_2 (22 mL) and stirred at ambient temperature for 24 h. The reaction was quenched, and then, purified by flash column chromatography to give the ε-lactone **16** (0.257 g, 53%) as a colourless solid. m.p. 110–112 °C (recrystallized from *n*-hexane/$CHCl_3$); IR (thin film): ν 3494, 2978, 2940, 1732, 1371, 1244, 1111, 1030 cm^{-1}; ^{1}H NMR (400 MHz, $CDCl_3$): δ 4.97 (td, *J* = 11.5, 4.8 Hz, 1H; γ-methine-H), 4.38 (qd, *J* = 12.9, 6.4 Hz, 1H; ε-methine-H), 2.74 (dd, *J* = 14.2, 1.5 Hz, 1H; α-methylene-H), 2.60 (dd, *J* = 14.2, 6.9 Hz, 1H; α-methylene-H), 2.33 (br, 1H; β-methine-H), 2.03 (s, 3H; CO_2CH_3), 1.99–1.86 (m, 2H; δ-methylene-H), 1.35 (d, *J* = 6.4 Hz, 3H; CH_3), 1.01 (d, *J* = 7.1 Hz, 3H; CH_3); ^{13}C NMR (100 MHz, $CDCl_3$): δ 172.2 (C), 169.7 (C), 74.0 (CH_2), 72.2 (CH_2), 36.5 (CH), 35.9 (CH_2), 29.6 (CH), 21.9 (CH_3), 20.8 (CH_3), 11.1 (CH_3); HRMS (FAB): *m/z* calcd for $C_{10}H_{17}O_4$ ($[M+ H]^+$): 201.1127, found: 201.1125. An X-ray analysis of the crystal confirmed the structure of **16** (Supplementary Fig. 1a, CCDC 1511847).

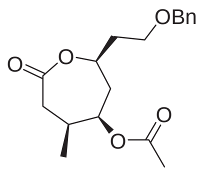

18

***rac*-(2*S*,4*R*,5*S*)/(2*R*,4*S*,5*R*)-2-[2-(benzyloxy)ethyl]-5-methyl-7-oxooxepan-4-yl acetate (18).** Using a procedure similar to *Method B* in the preparation of compound **11**, $Cu(OTf)_2$ (56 mg, 1.5 mmol), freshly distilled methylvinyl ketone (**15**, 0.400 mL, 4.52 mmol) and diene **17** [(*Z*,*E*)/(*E*,*E*) = 80/20, 1.00 g, 3.01 mmol (based on the (*Z*,*E*) isomer)] in CH_2Cl_2 (6.0 mL) were allowed to react at –78 °C for 12 h. Then, TFA (0.25 mL, 3.2 mmol) was added in one portion. After 20 min at 0 °C, the whole mixture was poured into a solution of *m*CPBA (5.40 g, 24.1 mmol) in CH_2Cl_2 (22.0 mL) and stirred at ambient temperature for 24 h. The reaction was quenched, and then, purified by flash column chromatography to give the ε-lactone **18** (0.331 g, 43%) as a colourless oil. IR (thin film): ν 2966, 1734, 1642, 1370, 1242, 1104, 1027, 740, 699 cm^{-1}; ^{1}H NMR (600 MHz, $CDCl_3$): δ 7.33–7.25 (m, 5H; Ph-H), 4.97 (td, *J* = 11.5, 4.6 Hz, 1H; γ-methine-H), 4.46 (s, 2H; benzylic H), 4.41 (td, *J* = 8.9, 3.6 Hz, 1H; ε-methine-H), 3.61 (td, *J* = 9.2, 4.3 Hz, 1H; linked chain-methylene-H), 3.53 (dt, *J* = 9.7, 5.0 Hz, 1H; 1H; linked chain-methylene-H), 2.73 (d, *J* = 14.2 Hz, 1H; α-methylene-H), 2.58 (dd, *J* = 14.2, 6.9 Hz, 1H; α-methylene-H), 2.36–2.33 (m, 1H; β-methylene-H), 2.03 (s, 3H; CO_2CH_3), 1.97–1.88 (m, 3H; δ-methine-H and linked chain-methylene-H), 1.83 (ddt, *J* = 13.7, 8.9, 4.6 Hz, 1H; δ-methine-H), 1.00 (d, *J* = 7.1 Hz, 3H; CH_3); ^{13}C NMR (150 MHz, $CDCl_3$): δ 172.6 (C), 170.7 (C), 138.1 (C), 128.4 (CH × 2), 127.72 (CH), 127.70 (CH × 2), 74.5 (CH), 73.1 (CH_2), 72.9 (CH), 65.6 (CH_2), 36.8 (CH_2), 36.2 (CH_2), 34.7 (CH_2), 29.9 (CH), 21.1 (CH_3), 11.4 (CH_3); HRMS (ESI): *m/z* calcd for $C_{18}H_{24}O_5Na$ ($[M + Na]^+$): 343.1512, found 343.1530.

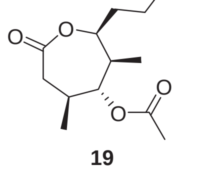

19

***rac*-(2*S*,3*R*,4*R*,5*S*)/(2*R*,3*S*,4*S*,5*R*)-2-[2-(benzyloxy)ethyl]-3,5-dimethyl-7-oxooxepan-4-yl acetate (19).** Using a procedure similar to *Method B* in the preparation of compound **11**, $BF_3{\cdot}Et_2O$ (19 μL, 0.15 mmol), freshly distilled ketone **9** (0.36 mL, 3.6 mmol) and diene **17** [(*Z*,*E*)/(*E*,*E*) = 80/20, 1.0 g, 3.01 mmol (based on the (*Z*,*E*) isomer] in CH_2Cl_2 (6.0 mL) were

allowed to react at −78 °C for 12 h. Then, TFA (0.25 mL, 3.2 mmol) was added in one portion. After 20 min at 0 °C, the whole mixture was poured into a solution of *m*CPBA (5.4 g, 24.1 mmol) in CH_2Cl_2 (22.0 mL) and stirred at ambient temperature for 24 h. The reaction was quenched, and then, purified by flash column chromatography ε-lactone **19** (0.515 g, 64%) as a colourless oil. m.p. 67–68 °C (the crystals were grown in refrigerator); IR (thin film): ν 2973, 1733, 1636, 1369, 1268, 1239, 1092, 1025, 739, 698 cm^{-1}; ^{1}H NMR (600 MHz, $CDCl_3$): δ 7.34–7.26 (m, 5H; Ph-H), 4.93 (dd, *J* = 9.5, 3.9 Hz, 1H; ε-methine-H), 4.85 (dd, *J* = 4.1, 3.0 Hz, 1H; γ-methine-H), 4.50 (<u>A</u>Bq, *J* = 11.9 Hz, 1H; benzylic H), 4.46 (A<u>B</u>q, *J* = 11.9 Hz, 1H; benzylic H), 3.60 (td, *J* = 9.2, 4.5 Hz, 1H; linked chain-methylene-H), 3.56–3.53 (m, 1H; linked chain-methylene-H), 3.19 (dd, *J* = 14.0, 3.0 Hz, 1H; α-methylene-H), 2.38 (dd, *J* = 14.0, 5.6 Hz, 1H; α-methylene-H), 2.15 (br, 1H; β-methylene-H), 2.07–2.04 (m, 1H; linked chain-methylene-H), 2.02 (s, 3H; CO_2CH_3), 2.03–2.00 (m, 1H; δ-methine-H), 1.70 (dddd, *J* = 14.3, 9.2, 5.3, 4.1 Hz, 1H; linked chain-methylene-H), 1.15 (d, *J* = 7.7 Hz, 3H; Me); 1.08 (d, *J* = 7.7 Hz, 3H; Me); ^{13}C NMR (150 MHz, $CDCl_3$): δ 173.6 (C), 169.6 (C), 138.2 (C), 128.4 (CH × 2), 127.7 (CH), 127.6 (CH × 2), 78.0 (CH), 73.2 (CH), 73.1 (CH_2), 66.2 (CH_2), 40.9 (CH), 35.1 (CH_2), 33.5 (CH_2), 32.6 (CH), 21.3 (CH_3), 16.8 (CH_3), 10.5 (CH_3); HRMS (ESI): *m/z* calcd for $C_{19}H_{26}O_5Na$ ($[M + Na]^+$): 357.1678, found: 357.1683.

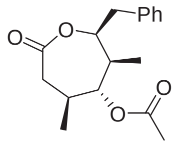

21

***rac*-(2*S*,3*R*,4*R*,5*S*)/(2*R*,3*S*,4*S*,5*R*)-2-benzyl-3,5-dimethyl-7-oxooxepan-4-yl acetate (21).** Using a procedure similar to *Method B* in the preparation of compound **11**, $BF_3 \cdot Et_2O$ (19 μL, 0.15 mmol), freshly distilled ketone **9** (0.36 mL, 3.61 mmol) and diene **20** [(*Z,E*)/(*E,E*) = 87/13, 0.870 g, 3.02 mmol (based on the (*Z,E*) isomer)] in CH_2Cl_2 (6.0 mL) were allowed to react at –78 °C for 12 h. Then, TFA (0.26 mL, 3.4 mmol) was added in one portion. After 20 min at 0 °C, the whole mixture was poured into a solution of *m*CPBA (5.40 g, 24.1 mmol) in CH_2Cl_2 (22.0 mL) and stirred at ambient temperature for 24 h. The reaction was quenched, and then, purified by flash column chromatography to give the ε-lactone **21** (0.486 g, 64%) as a colourless solid. m.p. 115–116 °C (recrystallized from *n*-hexane/$CHCl_3$); IR (thin film): ν 2974, 1732, 1637, 1455, 1266, 1239, 1181, 1162, 1022, 971, 712 cm^{-1}; ^{1}H NMR (600 MHz, $CDCl_3$): δ 7.29 (t, *J* = 7.5 Hz, 2H; Ph-H), 7.22 (t, *J* = 7.5 Hz, 1H; Ph-H), 7.19 (d, *J* = 7.6 Hz, 2H; Ph-H), 4.88 (t, *J* = 7.2 Hz, 1H; ε-methine-H), 4.80 (s, 1H, 1H; γ-methine-H), 3.18 (dd, *J*

= 14.0, 2.6 Hz, 1H; α-methylene-H), 3.13 (dd, J = 13.9, 7.1 Hz, 1H; benzylic H), 2.74 (dd, J = 13.9, 7.4 Hz, 1H; benzylic H), 2.39 (dd, J = 14.0, 5.3 Hz, 1H; α-methylene-H), 2.12 (br, 1H; β-methine-H), 2.02–1.98 (m, 1H; δ-methine-H), 1.90 (s, 3H, CO_2CH_3), 1.16 (d, J = 7.8 Hz; 3H, CH_3), 1.14 (d, J = 7.7 Hz, 3H; CH_3); ^{13}C NMR (150 MHz, $CDCl_3$): δ 173.3 (C), 169.3 (C), 137.3 (C), 129.0 (CH × 2), 128.7 (CH × 2), 126.9 (CH), 77.8 (CH), 77.4 (CH), 39.2 (CH), 39.1 (CH_2), 35.3 (CH_2), 32.6 (CH), 21.1 (CH_3), 16.9 (CH_3), 10.2 (CH_3); HRMS (ESI): *m/z* calcd for $C_{17}H_{22}O_4Na$ ($[M + Na]^+$): 313.1416, found: 313.1420.

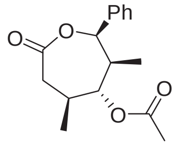

23

***rac*-(2*R*,3*S*,4*R*,5*S*)/(2*R*,3*S*,4*R*,5*S*)-3,5-dimethyl-7-oxo-2-phenyloxepan-4-yl acetate (23).** Using a procedure similar to *Method B* in the preparation of compound **11**, $BF_3 \cdot Et_2O$ (19 μL, 0.15 mmol), freshly distilled ketone **9** (0.36 mL, 3.61 mmol) and diene **22** [(*Z,E*)/(*E,E*) = 88/12, 0.830 g, 3.03 mmol (based on the (*Z,E*) isomer)] in CH_2Cl_2 (6.0 mL) were allowed to react at –78 °C for 12 h. Then, TFA (0.26 mL, 3.4 mmol) was added in one portion. After 20 min at 0 °C, the whole mixture was poured into a solution of *m*CPBA (5.40 g, 24.1 mmol) in CH_2Cl_2 (22.0 mL) and stirred at ambient temperature for 24 h. The reaction was quenched, and then, purified by flash column chromatography to give the ε-lactone **23** (0.456 g, 62%) as a colourless solid. m.p. 116–117 °C (recrystallized from *n*-hexane/$CHCl_3$); IR (thin film): ν 2975, 2937, 1735, 1453, 1382, 1369, 1266, 1237, 1159, 1024, 970, 730, 701 cm^{-1}; ^1H NMR (600 MHz, $CDCl_3$): δ 7.35–7.33 (br, 4H; Ph-H), 7.27–7.26 (m, 1H; Ph-H), 5.85 (s, 1H; ε-methine-H), 4.97 (s, 1H, 1H; γ- methine-H), 3.35 (d, J = 14.1 Hz, 1H; α-methylene-H), 2.51 (dd, J = 14.1, 4.8 Hz, 1H; α-methylene-H), 2.25 (br, 2H; β- and δ-methine-H), 2.17 (s, 3H, CO_2CH_3), 1.23 (d, J = 7.6 Hz; 3H, CH_3), 0.97 (d, J = 7.6 Hz, 3H; CH_3); ^{13}C NMR (150 MHz, $CDCl_3$): δ 172.7 (C), 169.5 (C), 138.6 (C), 128.3 (CH × 2), 127.7 (CH), 125.6 (CH × 2), 77.9 (CH), 77.2 (CH), 44.5 (CH), 35.0 (CH_2), 32.6 (CH), 21.4 (CH_3), 16.9 (CH_3), 9.7 (CH_3); HRMS (ESI): *m/z* calcd for $C_{16}H_{20}O_4Na$ ($[M + Na]^+$): 299.1259, found: 299.1260. An X-ray analysis of the crystal confirmed the structure of **23** (Supplementary Fig. 1b, CCDC 1511848).

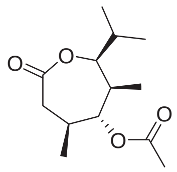

25

***rac*-(2*S*,3*R*,4*R*,5*S*)/(2*R*,3*S*,4*S*,5*R*)-2-isopropyl-3,5-dimethyl-7-oxooxepan-4-yl acetate (25).** Using a procedure similar to *Method B* in the preparation of compound **11**, $BF_3·Et_2O$ (19 μL, 0.15 mmol), freshly distilled ketone **9** (0.36 mL, 3.632 mmol) and diene **24** [(*Z,E*)/(*E,E*) = 91/9, 0.726 g, 3.03 mmol) in CH_2Cl_2 (6.0 mL) were reacted at –78 °C for 12 h. Then, TFA (0.26 mL, 3.4 mmol) was added in one portion. After 20 min at 0 °C, the whole mixture was poured into a solution of *m*CPBA (5.4 g, 24.1 mmol) in CH_2Cl_2 (22.0 mL) and stirred at ambient temperature for 24 h. The reaction was quenched and purified by flash column chromatography to give the ε-lactone **25** (0.426 g, 64%) as a colourless solid. m.p. 68–69 °C (recrystallized from *n*-hexane/$CHCl_3$); IR (thin): ν 2969, 2941, 1734, 1454, 1389, 1370, 1269, 1240, 1056, 1023, 971 cm^{-1}; ^{1}H NMR (600 MHz, $CDCl_3$): δ 4.85 (t, *J* = 3.2 Hz, 1H; ε-methine-H), 4.15 (d, *J* = 3.2 Hz, 1H; γ- methine-H), 3.20 (dd, *J* = 14.0, 3.2 Hz, 1H; α-methylene-H), 2.39 (dd, *J* = 14.0, 5.3 Hz, 1H; α-methylene-H), 2.22 (qd, *J* = 7.5, 3.3 Hz, 1H; δ-methine-H), 1.97–1.93 (m, 1H; β-methine-H), 2.06 (s, 3H, CO_2CH_3), 1.95–1.89 (m, 1H; *i*Pr-H), 1.15 (d, *J* = 7.7 Hz; 3H, CH_3), 1.04 (d, *J* = 7.1 Hz, 6H; CH_3), 0.83 (d, *J* = 6.8 Hz, 3H; CH_3); ^{13}C NMR (150 MHz, $CDCl_3$): δ 173.6 (C), 169.6 (C), 82.4 (CH), 78.1 (CH), 37.8 (CH), 35.0 (CH_2), 32.6 (CH), 29.6 (CH), 21.3 (CH_3), 20.3 (CH_3), 19.1 (CH_3), 16.9 (CH_3), 10.5 (CH_3); HRMS (APCI): *m/z* calcd for $C_{13}H_{23}O_4$ ($[M + H]^+$): 243.1596, found: 243.1603.

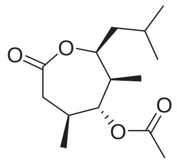

27

***rac*-(2*S*,3*R*,4*R*,5*S*)/(2*R*,3*S*,4*S*,5*R*)-2-isobutyl-3,5-dimethyl-7-oxooxepan-4-yl acetate (27).** Using a procedure similar to *Method B* in the preparation of compound **11**, $BF_3·Et_2O$ (19 μL, 0.15 mmol), freshly distilled ketone **9** (0.36 mL, 3.61 mmol) and diene **26** [(*Z,E*)/(*E,E*) = 80/20, 0.770 g, 3.03 mmol (based on the (*Z,E*) isomer)] in CH_2Cl_2 (6.0 mL) were allowed to react at –78 °C for 12 h. Then, TFA (0.26 mL, 3.4 mmol) was added in one portion. After 20 min at 0 °C, the whole mixture was poured into a solution of *m*CPBA (5.4 g, 24.1 mmol) in CH_2Cl_2 (22.0 mL) and stirred at ambient temperature for 24 h. The reaction was quenched,

and then, purified by flash column chromatography to give the ε-lactone **27** (0.373 g, 60%) as a colourless solid. m.p. 42–43 °C (recrystallized from *n*-hexane/$CHCl_3$); IR (thin film): ν 2957, 1734, 1636, 1454, 1388, 1369, 1268, 1238, 1183, 1034, 970 cm^{-1}; ^{1}H NMR (600 MHz, $CDCl_3$): δ 4.86 (t, *J* = 3.1 Hz, 1H; ε-methine-H), 4.75 (dd, *J* = 8.3, 5.2 Hz, 1H; γ- methine-H), 3.21 (dd, *J* = 14.0, 3.2 Hz, 1H; α-methylene-H), 2.40 (dd, *J* = 14.0, 5.5 Hz, 1H; α-methylene-H), 2.17–2.13 (m, 1H; δ-methine-H), 2.06 (s, 3H, CO_2CH_3), 1.97 (qd, *J* = 7.9, 3.4 Hz, 1H; β-methine-H), 1.75–1.67 (m, 2H; *i*Bu-methylene-H), 1.23–1.20 (m, 1H; *i*Bu-methine-H), 1.15 (d, *J* = 7.7 Hz; 3H, CH_3), 1.07 (d, *J* = 7.1 Hz, 3H; CH_3), 0.91 (d, *J* = 6.4 Hz, 3H; CH_3), 0.90 (d, *J* = 6.5 Hz, 3H; CH_3); ^{13}C NMR (150 MHz, $CDCl_3$): δ 173.7 (C), 169.6 (C), 78.2 (CH), 74.7 (CH), 41.8 (CH_2), 40.9 (CH), 35.2 (CH_2), 32.6 (CH), 24.7 (CH), 22.5 (CH_3), 22.1 (CH_3), 21.3 (CH_3), 16.8 (CH_3), 10.5 (CH_3); HRMS (APCI): *m/z* calcd for $C_{13}H_{23}O_4$ ($[M + H]^+$): 257.1753, found 257.1759.

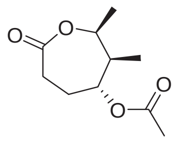

29

***rac*-(2*S*,3*R*,4*R*)/(2*R*,3*S*,4*S*)-2,3-dimethyl-7-oxooxepan-4-yl acetate (29).** Using a procedure similar to *Method B* in the preparation of compound **11**, $BF_3{\cdot}Et_2O$ (19 μL, 0.15 mmol), freshly distilled ketone **9** (0.36 mL, 3.61 mmol) and diene **28** [(*Z*)/(*E*) = 87/13, 0.600 g, 3.03 mmol (based on the (*Z*) isomer) in CH_2Cl_2 (6.0 mL) were allowed to react at –78 °C for 12 h. Then, TFA (0.26 mL, 3.4 mmol) was added in one portion. After 20 min at 0 °C, the whole mixture was poured into a solution of *m*CPBA (5.40 g, 24.1 mmol) in CH_2Cl_2 (22 mL) and stirred at ambient temperature for 24 h. The reaction was quenched, and then, purified by flash column chromatography to give the ε-lactone **29** (0.327 g, 62%) as a colourless oil. m.p. 91–92 °C (recrystallized from *n*-hexane/$CHCl_3$); IR (thin film): ν 2981, 2943, 1732, 1376, 1271, 1241, 1199, 1119, 1017, 969 cm^{-1}; ^{1}H NMR (600 MHz, $CDCl_3$): δ 4.93–4.89 (m, 2H; γ- and ε-methine-H), 2.94 (ddd, *J* = 14.6, 12.4, 3.5 Hz, 1H; α-methylene-H), 2.43 (ddd, *J* = 14.2, 5.8, 2.2 Hz, 1H; α-methylene-H), 2.09 (s, 3H; CO_2CH_3), 1.97–1.93 (m, 3H; β-methylene and δ-methine-H), 1.33 (d, *J* = 6.6 Hz, 3H, CH_3), 0.98 (d, *J* = 7.3 Hz, 3H, CH_3); ^{13}C NMR (150 MHz, $CDCl_3$): δ 174.6 (C), 169.8 (C), 73.2 (CH), 71.9 (CH), 41.0 (CH), 28.3 (CH_2), 22.9 (CH_2), 21.3 (CH_3), 19.8 (CH_3), 9.3 (CH_3); HRMS (ESI): *m/z* calcd for $C_{10}H_{16}O_4Na$ ($[M + Na]^+$): 223.0946, found 223.0945. An X-ray analysis of the crystal confirmed the structure of **29** (Supplementary Fig. 1c, CCDC 1511849).

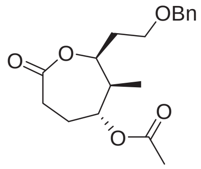

31

***rac*-(2*S*,3*S*,4*R*)/(2*R*,3*R*,4*S*)-2-(2-(benzyloxy)ethyl)-3-methyl-7-oxooxepan-4-yl acetate (31).** Using a procedure similar to *Method B* in the preparation of compound **11**, $BF_3·Et_2O$ (18 μL, 0.15 mmol), freshly distilled ketone **9** (0.35 mL, 3.63 mmol) and diene **30** [(*Z*)/(*E*) = 85/15, 0.92 g, 3.03 mmol (based on the (*Z*) isomer)] in CH_2Cl_2 (6.0 mL) were reacted at –78 °C for 12 h. Then, TFA (0.25 mL, 3.2 mmol) was added in one portion. After 20 min at 0 °C, the whole mixture was poured into a solution of *m*CPBA (5.4 g, 24.2 mmol) in CH_2Cl_2 (22 mL) and stirred at ambient temperature for 24 h. The reaction was quenched, and then, purified by flash column chromatography to give the ε-lactone **31** (0.481 g, 61%) as a colourless oil. IR (thin film): ν 2941, 2865, 1771, 1732, 1455, 1372, 1240, 1102, 1019, 976, 741, 699 cm^{-1}; ^1H NMR (600 MHz, $CDCl_3$): δ 7.34–7.27 (m, 5H; Ph-H), 4.91 (br, 2H; γ- and ε-methine-H), 4.50 (<u>A</u>Bq, *J* = 12.4 Hz, 1H; benzylic H), 4.46 (A<u>B</u>q, *J* = 12.4 Hz, 1H; benzylic H), 3.61–3.54 (m, 2H; methylene-H), 2.92 (dd, *J* = 14.6, 13.8 Hz, 1H; α-methylene-H), 2.40 (dd, *J* = 13.8, 3.9 Hz, 1H; α-methylene-H), 2.08–2.06 (m, 2H; β-methylene and δ-methine-H), 2.04 (s, 3H; CO_2CH_3), 1.96–1.94 (m, 2H; linked chain-methylene-H), 1.72–1.69 (m, 1H; β-methylene-H), 0.96 (d, *J* = 7.3 Hz, 3H; CH_3); ^{13}C NMR (150 MHz, $CDCl_3$): δ 174.7 (C), 169.8 (C), 138.2 (C), 128.4 (CH × 2), 127.7 (CH), 127.6 (CH × 2), 73.2 (CH), 73.1 (CH_2), 72.7 (CH), 66.2 (CH_2), 39.6 (CH), 33.7 (CH_2), 28.3 (CH_2), 23.0 (CH_2), 21.3 (CH_3), 9.7 (CH_3); HRMS (ESI): *m/z* calcd for $C_{18}H_{24}O_5Na$ ($[M + Na]^+$): 343.1521, found: 343.1530.

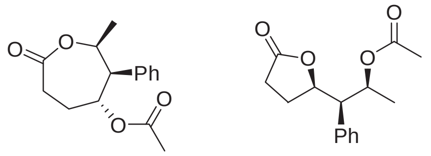

33 **34**

***rac*-(2*S*,3*R*,4*R*)/(2*R*,3*S*,4*S*)-2-methyl-7-oxo-3-phenyloxepan-4-yl acetate (33) and *rac*-(1*R*,2*S*)/(1*S*,2*R*)-1-[(*R*)/(*S*)-tetrahydro-5-oxofuran-2-yl]-1-phenylpropan-2-yl acetate (34).** Using a procedure similar to *Method B* in the preparation of compound **11**, $BF_3·Et_2O$ (19 μL, 0.15 mmol), ketone **32** (0.530 g, 3.636 mmol) and diene **28** [(*Z*)/(*E*) = 87/13, 0.600 g, 3.03 mmol (based on the (*Z*) isomer)] in CH_2Cl_2 (6.0 mL) were allowed to react at –78 °C for 12 h.

Then, TFA (0.25 mL, 3.2 mmol) was added in one portion. After 20 min at 0 °C, the whole mixture was poured into a solution of *m*CPBA (5.40 g, 24.2 mmol) in CH_2Cl_2 (22 mL) and stirred at ambient temperature for 24 h. The reaction was quenched, and then, purified by flash column chromatography to give the lactones **33** (0.313 g, 45%) and **34** (0.125 g, 18%) as colourless oils. For compound **33**; IR (thin film): ν 2984, 2936, 1774, 1733, 1455, 1373, 1242, 1186, 1078, 1042, 913, 759, 705 cm^{-1}; ^{1}H NMR (600 MHz, $CDCl_3$): δ 7.34–7.29 (m, 4H; Ph-H), 7.27–7.25 (m, 1H; Ph-H), 5.12–5.09 (m, 2H; γ- and ε-methine-H), 3.14 (dd, *J* = 14.7, 12.9 Hz, 1H; α-methylene-H), 3.03 (s, 1H; δ-methine-H), 2.59 (dd, *J* = 14.7, 6.6 Hz, 1H; α-methylene-H), 2.11 (s, 3H; CO_2CH_3), 2.06 (d, *J* = 12.9 Hz, 1H; β-methylene-H), 1.97–1.93 (m, 1H; β-methylene-H), 1.27 (d, *J* = 6.6 Hz, 3H; CH_3); ^{13}C NMR (150 MHz, $CDCl_3$): δ 174.3 (C), 169.7 (C), 135.6 (C), 129.9 (CH × 2), 128.8 (CH × 2), 127.6 (CH), 73.8 (CH), 72.6 (CH), 53.6 (CH), 28.6 (CH_2), 23.3 (CH_2), 21.3 (CH_3), 20.2 (CH_3); HRMS (ESI): *m/z* calcd for $C_{15}H_{18}O_4Na$ ($[M + Na]^+$): 285.1103, found: 285.1099. For compound **34**; m.p. 117–119 °C (recrystallized from *n*-hexane/$CHCl_3$); IR (thin film): ν 2985, 2935, 1774, 1732, 1455, 1373, 1243, 1042 cm^{-1}; ^{1}H NMR (600 MHz, $CDCl_3$): δ 7.30–7.25 (m, 5H; Ph-H), 5.50–5.46 (m, 1H; ε-methine-H), 4.98 (ddd, *J* = 12.1, 7.2, 6.9, Hz, 1H; γ-methine-H), 2.81–2.78 (m, 1H; δ-methine-H), 2.34–2.25 (m, 2H; α-methylene-H), 2.01–1.98 (m, 1H; β-methylene-H), 1.89 (s, 3H; CO_2CH_3), 1.88–1.85 (m, 1H; β-methylene-H), 1.25 (d, *J* = 6.4 Hz, 1H; CH_3); ^{13}C NMR (150 MHz, $CDCl_3$): δ 176.8 (C), 170.2 (C), 135.8 (C), 129.9 (CH × 2), 128.5 (CH × 2), 127.5 (CH), 79.9 (CH), 70.4 (CH), 55.6 (CH), 28.1 (CH_2), 26.1 (CH_3), 21.0 (CH_2), 18.6 (CH_3); HRMS (ESI): *m/z* calcd for $C_{15}H_{18}O_4Na$ ($[M + Na]^+$): 285.1103, found: 285.1111. An X-ray analysis of the crystal confirmed the structure of **34** (Supplementary Fig. 1d, CCDC 1511880).

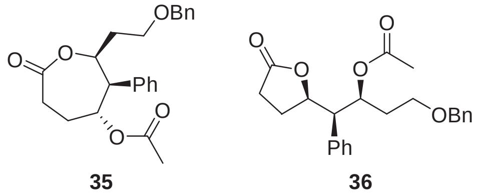

35 **36**

***rac*-(2*S*,3*S*,4*R*)/(2*R*,3*R*,4*S*)-2-[2-(benzyloxy)ethyl]-7-oxo-3-phenyloxepan-4-yl acetate (35) and rac-(1*S*,2*S*)/(1*R*,2*R*)-4-(benzyloxy)-1-[(*R*)/(*S*)-tetrahydro-5-oxofuran-2-yl]-1-phenylbutan-2-yl acetate (36).** Using a procedure similar to *Method B* in the preparation of compound **11**, $BF_3·Et_2O$ (18 µL, 0.15 mmol), ketone **32** (0.530 g, 3.63 mmol) and diene **30** [(*Z*)/(*E*) = 85/15, 0.92 g, 3.03 mmol] in CH_2Cl_2 (6.0 mL) were reacted at –78 °C for 12 h.

Then, TFA (0.25 mL, 3.2 mmol) was added in one portion. After 20 min at 0 °C, the whole mixture was poured into a solution of *m*CPBA (5.4 g, 24.2 mmol) in CH_2Cl_2 (22 mL) and stirred at ambient temperature for 24 h. The reaction was quenched, and then, purified by flash column chromatography to give the lactones **35** (0.398 g, 42%) and **36** (0.132 g, 14%) as colourless oils. For compound **35**: IR (thin film): ν 2932, 2864, 1734, 1454, 1368, 1236, 1160, 1098, 1026, 743, 701 cm^{-1}; ^1H NMR (600 MHz, $CDCl_3$): δ 7.35–7.25 (m, 10H; Ph-H), 5.12 (dd, *J* = 9.1, 3.9 Hz, 1H; ε-methine-H), 5.08 (s, 1H; γ-methine-H), 4.48 (<u>A</u>Bq, *J* = 12.0 Hz, 1H; benzylic H), 4.45 (A<u>B</u>q, *J* = 12.0 Hz, 1H; benzylic H), 3.60–3.57 (m, 1H; linked chain-methylene-H), 3.48–3.45 (m, 1H; linked chain-methylene-H), 3.13–3.10 (m, 2H; α-methylene- and δ-methine-H), 2.59 (dd, *J* = 14.5, 6.0 Hz, 1H; α-methylene-H), 2.11–2.03 (m, 1H; β-methylene-H), 2.04 (s, 3H; CO_2CH_3), 1.96–1.89 (m, 1H; β-methylene-H), 1.87–1.85 (m, 1H; linked chain-methylene-H), 1.73–1.67 (m, 1H; linked chain-methylene-H); ^{13}C NMR (150 MHz, $CDCl_3$): δ 174.3 (C), 169.8 (C), 138.2 (C), 135.9 (C), 129.9 (CH × 2), 128.8 (CH × 2), 128.4 (CH × 2), 127.8 (CH), 127.7 (CH), 127.6 (CH × 2), 73.9 (CH), 73.2 (CH_2), 73.1 (CH), 65.9 (CH_2), 52.2 (CH), 34.0 (CH_2), 28.6 (CH_2), 23.5 (CH_2), 21.3 (CH_3); HRMS (ESI): *m/z* calcd for $C_{23}H_{26}O_5Na$ ($[M + Na]^+$): 405.1678, found: 405.1679. For compound **36**; IR (thin film): ν 2927, 2863, 1772, 1737, 1372, 1237, 1185, 1099, 1027, 912, 749, 702 cm^{-1}; ^1H NMR (600 MHz, $CDCl_3$): δ 7.35–7.25 (m, 10H; Ph-H), 5.62 (ddd, *J* = 9.1, 4.1, 3.9 Hz, 1H; γ-methine-H), 4.90 (dd, *J* = 12.4, 6.2 Hz, 1H; ε-methine-H), 4.46 (<u>A</u>Bq, *J* = 11.8 Hz, 1H; benzylic H), 4.43 (A<u>B</u>q, *J* = 11.8 Hz, 1H; benzylic H), 3.49–3.42 (m, 2H; linked chain-methylene-H), 2.87 (dd, *J* = 6.2, 4.1 Hz, 1H; δ-methine-H), 2.35–2.26 (m, 2H; α- and β-methylene-H), 2.02–1.98 (m, 1H; α-methylene-H), 1.96 (s, 3H; CO_2CH_3), 1.86–1.73 (m, 3H; β- and linked chain-methylene-H); ^{13}C NMR (150 MHz, $CDCl_3$): δ 176.8 (C), 170.6 (C), 138.0 (C), 135.5 (C), 130.3 (CH × 2), 128.39 (CH × 2), 128.37 (CH × 2), 127.8 (CH × 2), 127.7 (CH), 127.6 (CH), 80.4 (CH), 73.2 (CH_2), 71.3 (CH), 66.3 (CH_2), 54.3 (CH), 32.8 (CH_2), 28.1 (CH_2), 26.4 (CH_2), 21.0 (CH_3); HRMS (ESI): *m/z* calcd for $C_{23}H_{26}O_5Na$ ($[M + Na]^+$): 405.1678, found: 405.1679.

General procedure for deacetylation and translactonization. To a solution of the acetylated lactone (0.467 mmol, 1.0 equiv.) in MeOH (1.6 mL) was added NaOMe (0.514 mmol, 1.1 equiv.) at 0 °C. The resulting mixture was allowed to warm up to room temperature, and the reaction progress was monitored by TLC. Upon consumption of the starting material, the reaction was neutralized with Dowex 50 WX2-200 IR-resin. The

mixture was filtered through sintered glass, and the filtrate was evaporated under reduced pressure. The residue was purified by flash column chromatography (*n*-hexane/ethyl acetate = 1:2) to give the alcohol compound.

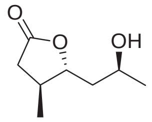

S2

***rac*-(4*S*,5*R*)/(4*R*,5*S*)-dihydro-5-[(*S*)/(*R*)-2-hydroxypropyl]-4-methylfuran-2(3*H*)-one (S2).** Using the general procedure for deacetylation and translactonization, ε-lactone **16** (93 mg, 0.468 mmol) was converted to compound **S2** (71 mg, 96%), a colourless oil. IR (thin film) ν: 2967, 2934, 1763, 1652, 1458, 1421, 1376, 1328, 1219, 1163, 1050, 982, 946 cm^{-1}; ^1H NMR (400 MHz, $CDCl_3$): δ 4.27 (ddd, *J* = 10.1, 7.9, 2.6 Hz, 1H; γ-methine-H), 4.09–4.03 (m, 1H; ε-methine-H), 2.68–2.60 (m, 1H; β-methine-H), 2.23–2.16 (m, 2H; α-methylene-H), 1.74 (ddd, *J* = 14.5, 7.9, 2.6 Hz, 1H; δ-methylene-H), 1.63 (ddd, *J* = 14.5, 10.1, 3.0 Hz, 1H; δ-methylene-H), 1.22 (d, *J* =6.3 Hz, 1H; CH_3), 1.12 (d, *J* =6.3 Hz, 1H; CH_3); ^{13}C NMR (100 MHz, $CDCl_3$): δ 176.6 (C), 84.0 (CH), 64.3 (CH), 42.8 (CH_2), 36.9 (CH), 36.4 (CH_2), 24.0 (CH_3), 16.7 (CH_3); HRMS (FAB): *m/z* calcd for $C_8H_{15}O_3$ ($[M + H]^+$): 159.1021, found 159.1024.

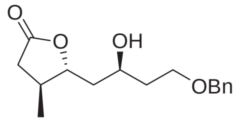

S3

***rac*-(4*S*,5*R*)/(4*R*,5*S*)-5-[(*S*)/(*R*)-4-(benzyloxy)-2-hydroxybutyl]-dihydro-4-methylfuran-2(3*H*)-one (S3).** Using the general procedure for deacetylation and translactonization, ε-lactone **18** (150 mg, 0.468 mmol) was converted to compound **S3** (123 mg, 94%), a colourless oil. IR (thin film): ν 2923, 2873, 1773, 1637, 1454, 1420, 1364, 1218, 1158, 1094, 1075, 940, 742, 699 cm^{-1}; ^1H NMR (600 MHz, $CDCl_3$): δ 7.34–7.26 (m, 1H; Ph-H), 4.51 (<u>A</u>Bq, *J* = 12.0 Hz, 1H; benzylic H), 4.49 (<u>A</u>Bq, *J* = 12.0 Hz, 1H; benzylic H), 4.29 (ddd, *J* = 9.8, 7.7, 2.0 Hz, 1H; γ-methine-H), 4.08–4.05 (m, 1H, ε-methine-H), 3.72 (dt, *J* = 9.5, 4.8 Hz, 1H; linked chain-methylene-H), 3.66 (dt, *J* = 9.5, 4.1 Hz, 1H; linked chain-methylene-H), 3.23 (s, 1H, OH), 2.65–2.60 (m, 1H; δ-methylene-H), 2.20–2.16 (m, 2H; δ-methylene- and β-methine-H), 1.81–1.73 (m, 3H, α-methylene- and linked chain-methylene-H), 1.63–1.58 (m, 1H, α-methylene-H), 1.12 (d, *J* = 6.2 Hz, 3H, Me); ^{13}C NMR (150 MHz, $CDCl_3$): δ 176.4 (C), 137.7 (C), 128.5 (CH × 2), 127.9 (CH), 127.7 (CH × 2), 83.9 (CH), 73.4

(CH_2), 69.3 (CH), 68.5 (CH_2), 41.7 (CH_2), 37.0 (CH), 36.7 (CH_2), 36.6 (CH_2), 16.8 (CH_3); HRMS (ESI): *m/z* calcd for $C_{16}H_{22}O_4Na$ ($[M + Na]^+$): 301.1416, found: 301.1411.

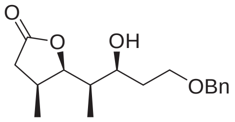

S4

***rac*-(4*S*,5*R*)/(4*R*,5*S*)-5-[(2*R*,3*S*)/(2*S*,3*R*)-5-(benzyloxy)-3-hydroxypentan-2-yl]-dihydro-4-methylfuran-2(3*H*)-one (S4).** Using the general procedure for deacetylation and translactonization, ε-lactone **19** (156 mg, 0.467 mmol) was converted to compound **S4** (128 mg, 94%), a colourless oil. m.p. 52–53 °C (recrystallized from *n*-hexanes/$CHCl_3$); IR (thin film): ν 2924, 1770, 1647, 1455, 1421, 1218, 1183, 1155, 1100, 973, 740, 699 cm^{-1}; ^{1}H NMR (600 MHz, $CDCl_3$): δ 7.34–7.32 (m, 2H; Ph-H), 7.29–7.27 (m, 3H; Ph-H), 4.52 (<u>A</u>Bq, *J* = 11.8 Hz, 1H; benzylic H), 4.46 (A<u>B</u>q, *J* = 11.8 Hz, 1H; benzylic H), 4.48 (dd, *J* = 8.9, 4.8 Hz, 1H; γ-methine-H), 3.88 (ddd, *J* = 9.7, 4.1, 2.3 Hz, 1H; ε-methine-H), 3.74 (dt, *J* = 9.7, 4.3 Hz, 1H; linked chain-methylene-H), 3.64 (td, *J* = 9.7, 3.2 Hz, 1H; linked chain-methylene-H), 2.98 (br, 1H; OH), 2.72 (dd, *J* = 16.5, 7.4 Hz, 1H; α-methylene-H), 2.68–2.66 (m, 1H; β-methine-H), 2.18 (dd, *J* = 16.5, 1.4 Hz, 1H; α-methylene-H), 1.97–1.91 (m, 1H; linked chain-methylene-H), 1.86–1.81 (m, 1H; δ-methine-H), 1.59–1.56 (m, 1H; linked chain-methylene-H), 1.05 (d, *J* = 6.8 Hz, 3H; CH_3); 0.98 (d, *J* = 6.9 Hz, 3H; CH_3); ^{13}C NMR (150 MHz, $CDCl_3$): δ 176.9 (C), 137.6 (C), 128.5 (CH × 2), 127.9 (CH), 127.7 (CH × 2), 84.8 (CH), 73.5 (CH_2), 71.4 (CH), 69.8 (CH_2), 38.8 (CH_2), 38.1 (CH), 33.9 (CH_2), 32.4 (CH), 14.1 (CH_3), 10.1 (CH_3); HRMS (ESI): *m/z* calcd for $C_{17}H_{24}O_4Na$ ($[M + Na]^+$): 315.1572, found 315.1566.

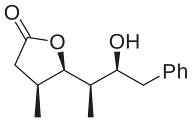

S5

***rac*-(4*S*,5*R*)/(4*R*,5*S*)-dihydro-5-[(2*R*,3*S*)/(2*S*,3*R*)-3-hydroxy-4-phenylbutan-2-yl)-4-methylfuran-2(3*H*)-one (S5).** Using the general procedure for deacetylation and translactonization, ε-lactone **21** (136 mg, 0.469 mmol) was converted to compound **S5** (112 mg, 96%), a colourless oil. m.p. 97–98 °C (recrystallized from *n*-hexanes/$CHCl_3$); IR (thin film): ν 2976, 1768, 1736, 1455, 1220, 1175, 1150, 997, 971, 934, 749, 702 cm^{-1}; ^{1}H NMR (600 MHz, $CDCl_3$): δ 7.30 (t, *J* = 7.4 Hz, 2H; Ph-H), 7.23 (t, *J* = 7.4 Hz, 1H; Ph-H), 7.18 (d, *J* = 7.5 Hz, 2H; Ph-H), 4.46 (dd, *J* = 9.3, 4.8 Hz, 1H; γ-methine-H), 3.84 (dd, *J* = 6.3, 4.8 Hz,

1H; ε-methine-H), 2.81 (dd, *J* = 13.4, 8.5 Hz, 1H; benzylic H), 2.75 (dd, *J* = 13.4, 5.4 Hz, 1H; benzylic H), 2.70 (dd *J* = 16.9, 7.5 Hz, 1H; α-methylene-H), 2.62–2.58 (m, 1H; β-methine-H), 2.15 (d, *J* = 16.9 Hz, 1H; α-methylene-H), 1.92–1.87 (m, 1H; δ-methine-H), 1.80 (br, H; OH), 1.15 (d, *J* = 6.8 Hz, 3H; CH_3), 0.84 (d, *J* = 7.1 Hz, 3H; CH_3); ^{13}C NMR (150 MHz, $CDCl_3$): δ 176.9 (C), 137.9 (C), 129.2 (CH × 2), 128.7 (CH × 2), 126.7 (CH), 85.3 (CH), 72.1 (CH), 41.5 (CH_2), 38.7 (CH_2), 36.7 (CH), 32.1 (CH), 13.7 (CH_3), 9.6 (CH_3); HRMS (ESI): *m/z* calcd for $C_{15}H_{20}O_3Na$ ($[M + Na]^+$): 271.1310, found: 271.1309.

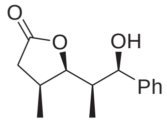

S6

***rac*-(4*S*,5*R*)/(4*R*,5*S*)-dihydro-5-[(1*R*,2*R*)/(1*S*,2*S*)-1-hydroxy-1-phenylpropan-2-yl]-4-methylfuran-2(3*H*)-one (S6).** Using the general procedure for deacetylation and translactonization, ε-lactone **23** (130 mg, 0.470 mmol) was converted to compound **S6** (105 mg, 95%), a colourless oil. IR (thin film): ν 2977, 1741, 1764, 1641, 1452, 1221, 1181, 1151, 1000, 975, 935, 755, 703 cm^{-1}; ^{1}H NMR (600 MHz, $CDCl_3$): δ 7.35 (t, *J* = 7.4 Hz, 2H; Ph-H), 7.31 (d, *J* = 7.6 Hz, 2H; Ph-H), 7.28 (d, *J* = 7.2 Hz, 1H; Ph-H), 4.84 (d, *J* = 2.8 Hz, 1H; γ-methine-H), 4.46 (dd, *J* = 7.5, 5.0 Hz, 1H; ε-methine-H), 2.75–2.71 (m, 2H; α-methylene- and β-methine-H), 2.25 (dd *J* = 19.9, 6.5 Hz, 1H; α-methylene-H), 2.10 (br, 1H; δ-methine-H), 1.92 (br, H; OH), 1.13 (d, *J* = 6.2 Hz, 3H; Me), 0.99 (d, *J* = 6.6 Hz, 3H; Me); ^{13}C NMR (150 MHz, $CDCl_3$): δ 176.8 (C), 142.6 (C), 128.5 (CH × 2), 127.7 (CH), 125.7 (CH × 2), 84.9 (CH), 73.8 (CH), 40.2 (CH_2), 38.4 (CH_2), 32.4 (CH), 13.9 (CH_3), 9.7 (CH_3); HRMS (ESI): *m/z* calcd for $C_{14}H_{18}O_3Na$ ($[M + Na]^+$): 257.1154, found: 257.1153.

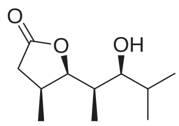

S7

***rac*-(4*S*,5*R*)/(4*R*,5*S*)-dihydro-5-[(2*R*,3*S*)/(2*S*,3*R*)-3-hydroxy-4-methylpentan-2-yl]-4-methylfuran-2(3H)-one (S7).** Using the general procedure for deacetylation and translactonization, ε-lactone **25** (95 mg, 0.393 mmol) was converted to compound **S7** (75 mg, 95%), a colourless oil. m.p. 112–113 °C (recrystallized from *n*-hexanes/$CHCl_3$); IR (thin film): ν 2977, 2944, 1757, 1736, 1459, 1379, 1345, 1229, 1193, 1164, 1085, 995, 966, 935 cm^{-1}; ^{1}H NMR (600 MHz, $CDCl_3$): δ 4.43 (dd, *J* = 9.9, 4.6 Hz, 1H; γ-methine-H), 3.16 (dd, *J*

= 9.4, 1.9 Hz, 1H; ε-methine-H), 2.74 (dd, *J* = 16.8, 7.3 Hz, 1H; α-methylene-H), 2.67–2.61 (m, 1H; β-methine-H), 2.20 (dd, *J* = 16.8, 1.2 Hz, 1H; α-methylene-H), 2.05–2.00 (m, 1H; δ-methine-H), 1.74–1.68 (m, 1H; *i*Pr-H), 1.53 (br, 1H; OH), 1.03 (d, *J* = 6.8 Hz, 3H; CH_3), 1.02 (d, *J* = 6.8 Hz, 3H; CH_3), 0.98 (d, *J* = 7.0 Hz, 3H; CH_3), 0.86 (d, *J* = 6.7 Hz, 3H; CH_3); ^{13}C NMR (150 MHz, $CDCl_3$): δ 176.8 (C), 86.1 (CH), 76.6 (CH), 39.0 (CH_2), 34.7 (CH), 32.0 (CH), 31.6 (CH), 19.7 (CH_3), 18.8 (CH_3), 13.7 (CH_3), 9.0 (CH_3); HRMS (ESI): *m/z* calcd for $C_{11}H_{21}O_3$ ($[M + H]^+$): 201.1491, found 201.1494.

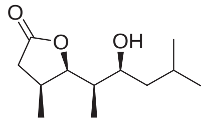

S8

***rac*-(4*S*,5*R*)/(4*R*,5*S*)-dihydro-5-[(2*R*,3*S*)/(2*S*,3*R*)-3-hydroxy-5-methylhexan-2-yl)-4-methylfuran-2(3*H*)-one (S8).** Using the general procedure for deacetylation and translactonization, ε-lactone **27** (92 mg, 0.359 mmol) was converted to compound **S8** (72 mg, 94%), a colourless oil. m.p. 72–73 °C (recrystallized from *n*-hexanes/$CHCl_3$); IR (thin film): ν 2956, 2933, 1767, 1738, 1464, 1386, 1220, 1185, 1156, 974, 934 cm^{-1}; ^{1}H NMR (600 MHz, $CDCl_3$): δ 4.44 (dd, *J* = 9.3, 4.8 Hz, 1H; γ-methine-H), 3.74 (ddd, *J* = 9.0, 3.9, 2.0 Hz, 1H; ε-methine-H), 2.73 (dd, *J* = 16.7, 7.5 Hz, 1H; α-methylene-H), 2.69–2.65 (m, 1H; β-methine-H), 2.20 (dd, *J* = 16.8, 1.3 Hz, 1H; α-methylene-H), 1.81–1.77 (m, 1H; δ-methine-H), 1.73–1.67 (m, 1H; *i*Pr-H), 1.48 (ddd, *J* = 14.4, 9.1, 5.6 Hz, 1H; *i*Bu-methylene-H), 1.32 (br, 1H; OH), 1.21 (ddd, *J* = 14.4, 8.5, 4.1 Hz, 1H; *i*Bu-methylene-H), 1.03 (d, *J* = 6.7 Hz, 3H; Me), 0.99 (d, *J* = 7.0 Hz, 3H; Me), 0.94 (d, *J* = 6.6 Hz, 3H; Me), 0.91 (d, *J* = 6.6 Hz, 3H; Me); ^{13}C NMR (150 MHz, $CDCl_3$): δ 176.8 (C), 85.6 (CH), 69.1 (CH), 44.6 (CH_2), 38.8 (CH_2), 38.0 (CH), 32.2 (CH), 24.6 (CH), 23.4 (CH_3), 22.1 (CH_3), 14.0 (CH_3), 9.4 (CH_3); HRMS (ESI): *m/z* calcd for $C_{12}H_{23}O_3$ ($[M + H]^+$): 215.1647, found 215.1653.

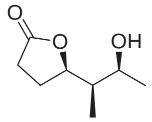

S9

***rac*-(*R*)/(*S*)-dihydro-5-[(2*R*,3*S*)/(2*S*,3*R*)-3-hydroxybutan-2-yl]furan-2(3H)-one (S9).** Using the general procedure for deacetylation and translactonization, ε-lactone **29** (94 mg, 0.470 mmol) was converted to compound **S9** (72 mg, 97%), a colourless oil. IR (thin film): ν 2974, 2925, 1764, 1460, 1351, 1225, 1191, 1148, 1013, 959, 902 cm^{-1}; ^{1}H NMR (600 MHz,

$CDCl_3$): δ 4.56 (ddd, *J* = 7.2, 6.6 Hz, 1H; γ-methine-H), 3.95 (qd, *J* = 6.4, 2.9 Hz, 1H; ε-methine-H), 2.52–2.49 (m, 2H; α-methylene-H), 2.30 (ddd, *J* = 13.1, 12.9, 6.6 Hz, 1H; β-methylene-H), 2.05–2.00 (m, 1H; β-methylene-H), 1.74 (br, 1H; OH), 1.67–1.64 (m, 1H; δ-methine-H), 1.20 (d, *J* = 6.4 Hz, 3H; CH_3) , 0.99 (d, *J* = 7.0 Hz, 3H; CH_3); ^{13}C NMR (150 MHz, $CDCl_3$): δ 177.2 (C), 83.0 (CH), 68.5 (CH), 43.8 (CH_2), 28.9 (CH_2), 26.2 (CH), 21.1 (CH_3), 8.3 (CH_3); HRMS (ESI): *m/z* calcd for $C_8H_{14}O_3Na$ ($[M + Na]^+$): 181.0841, found: 181.0836.

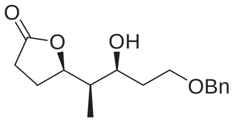

S10

***rac*-(*R*)/(*S*)-5-[(2*R*,3*S*)/(2*S*,3*R*)-5-(benzyloxy)-3-hydroxypentan-2-yl]-dihydrofuran-2(3*H*)-one (S10).** Using the general procedure for deacetylation and translactonization, ε-lactone **31** (150 mg, 0.469 mmol) was converted to compound **S10** (121 mg, 93%), a colourless oil.; IR (thin film): ν 2943, 2882, 1764, 1455, 1356, 1223, 1190, 1096, 1011, 972, 744, 699 cm^{-1}; ^{1}H NMR (600 MHz, $CDCl_3$): δ 7.33–7.26 (m, 5 H; Ph-H), 4.58 (dd, *J* = 7.2, 6.6 Hz, 1H; γ-methine-H), 4.52 (<u>A</u>Bq, *J* = 12.2 Hz, 1H; benzylic H), 4.49 (A<u>B</u>q, *J* = 12.2 Hz, 1H; benzylic H), 3.92 (d, *J* = 9.7 Hz, 1H; ε-methine-H), 3.74–3.73 (m, 1H; linked chain-methylene-H), 3.64–3.62 (m, 1H; linked chain-methylene-H), 3.13 (br, 1H; OH), 2.51–2.48 (m, 2H; α-methylene-H), 2.32–2.27 (m, 1H; β-methylene-H), 2.05–1.98 (m, 1H; β-methylene-H), 1.93–1.86 (m, 1H; linked chain-methylene-H), 1.71–1.62 (m, 1H; δ-methine-H), 1.60 (d, *J* = 14.6 Hz, 1H; linked chain-methylene-H), 1.00 (d, *J* = 6.9 Hz, 3H; CH_3); ^{13}C NMR (150 MHz, $CDCl_3$): δ 177.4 (C), 137.6 (C), 128.5 (CH × 2), 127.8 (CH), 127.7 (CH × 2), 82.4 (CH), 73.4 (CH_2), 72.1 (CH), 69.9 (CH_2), 43.0 (CH), 33.6 (CH_2), 29.0 (CH_2), 26.2 (CH_2), 9.3 (CH_3); HRMS (ESI): *m/z* calcd for $C_{16}H_{22}O_4Na$ ($[M + Na]^+$): 301.1416, found 301.1424.

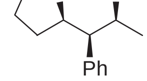

S11

***rac*-(*R*)/(*S*)-dihydro-5-[(1*R*,2*S*)/(1*S*,2*R*)-2-hydroxy-1-phenylpropyl)furan-2(3H)-one (S11).** *Method A*: Using the general procedure for deacetylation and translactonization, ε-lactone **33** (123 mg, 0.469 mmol) was converted to compound **S11** (99 mg, 95%), a

colourless oil. *Method B*: Using the general procedure for deacetylation and translactonization, γ-lactone **34** (66 mg, 0.252 mmol) was converted to compound **S11** (54 mg, 97%), a colourless oil. IR (thin film): ν 2976, 2924, 1765, 1462, 1355, 1225, 1191, 1146, 1013, 958, 902 cm^{-1}; ^{1}H NMR (600 MHz, $CDCl_3$): δ 7.35–7.27 (m, 1H; Ph-H), 4.96 (ddd, *J* = 7.2, 7.0, 4.5 Hz, 1H; γ-methine-H), 4.35–4.30 (m, 1H; ε-methine-H), 2.62 (dd, *J* = 6.9, 4.5 Hz, 1H; δ-methine-H), 2.37–2.27 (m, 2H; α-methylene-H), 1.98–1.94 (m, 1H; β-methylene-H), 1.86–1.81 (m, 1H; β-methylene-H), 1.49 (d, *J* = 3.7 Hz, 1H; OH), 1.28 (d, *J* = 6.2 Hz, 3H; Me); ^{13}C NMR (150 MHz, $CDCl_3$): δ 177.0 (C), 136.0 (C), 130.1 (CH × 2), 128.9 (CH × 2), 127.8 (CH), 80.5 (CH), 68.2 (CH), 58.1 (CH), 28.2 (CH_2), 26.0 (CH_2), 21.2 (CH_3); HRMS (ESI): *m/z* calcd for $C_{13}H_{16}O_3Na$ ($[M + Na]^+$): 243.0997, found: 243.0993.

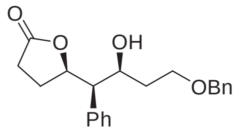

S12

***rac*-(*R*)/(*S*)-5-[(1*R*,2*S*)/(1*S*,2*R*)-4-(benzyloxy)-2-hydroxy-1-phenylbutyl]-dihydrofuran-2(3*H*)-one (S12).** *Method A*: Using the general procedure for deacetylation and translactonization, ε-lactone **35** (180 mg, 0.471 mmol) was converted to compound **S12** (152 mg, 95%), a colourless oil. *Method B*: Using the general procedure for deacetylation and translactonization, γ-lactone **36** (60 mg, 0.157 mmol) was converted to compound **S12** (51 g, 95%), a colourless oil. IR (thin film): ν 2926, 1772, 1646, 1457, 1422, 1218, 1183, 1156, 1100, 973, 740, 698 cm^{-1}; ^{1}H NMR (600 MHz, $CDCl_3$): δ 7.33–7.25 (m, 10H; Ph-H), 5.00 (dd, *J* = 6.6, 5.3 Hz, 1H; γ-methine-H), 4.48 (<u>A</u>Bq, *J* = 11.7 Hz, 1H; benzylic H), 4.46 (A<u>B</u>q, *J* = 11.7 Hz, 1H; benzylic H), 4.30 (br, 1H; ε-methine-H), 3.67–3.65 (m, 2H; linked chain-methylene-H), 2.90 (br, 1H; OH), 2.70 (dd, *J* = 6.0, 5.3 Hz, 1H; δ-methine-H), 2.43–2.33 (m, 2H; α- and β-methylene-H), 2.18–2.13 (m, 1H; β-methylene-H), 1.95–1.90 (m, 1H; α-methylene-H), 1.76–1.74 (m, 1H; linked chain-methylene-H), 1.69–1.64 (m, 1H; linked chain-methylene-H); ^{13}C NMR (150 MHz, $CDCl_3$): δ 177.0 (C), 137.7 (C), 136.8 (C), 129.9 (CH × 2), 128.5 (CH × 2), 128.4 (CH × 2), 127.8 (CH), 127.7 (CH × 2), 127.4 (CH), 81.2 (CH), 73.4 (CH_2), 71.5 (CH), 68.9 (CH_2), 56.1 (CH), 34.4 (CH_2), 28.6 (CH_2), 26.4 (CH_2); HRMS (ESI): *m/z* calcd for $C_{21}H_{24}O_4Na$ ($[M + Na]^+$): 363.1572, found 363.1571.

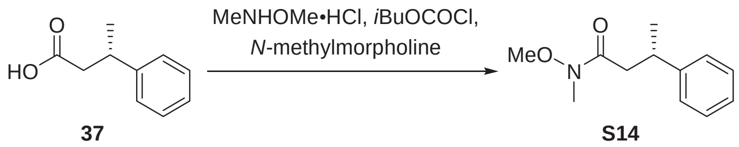

(*S*)-*N*-methoxy-*N*-methyl-3-phenylbutanamide (S14). Following a literature procedure[2], *N*-methylmorpholine (7 mL, 67.1 mmol) was added to a solution of (*S*)-3-phenylbutanoic acid (**37**, 5.0 g, 30.5 mmol) in CH_2Cl_2 (61 mL) at –4 °C (ice brine bath). While stirring at this temperature under N_2 atmosphere, isobutyl chloroformate (4.4 mL, 33.5 mmol) was added dropwise for over 30 minutes, and the reaction mixture was allowed to stir for an additional 15 minutes, in which the mixture changed from yellow to dark orange in colour. To this solution was quickly added *N*,*O*-dimethylhydroxylamine hydrochloride (3.5 g, 35.1 mmol), after which, the mixture was stirred for 8 h at 0 °C and then allowed to warm up to ambient temperature. The reaction solution was poured into vigorously stirring H_2O (75 mL), and then, transferred to a separatory funnel. The crude organic portion was extracted with CH_2Cl_2, and the combined organic layers were dried over $MgSO_4$, filtered, concentrated and purified by flash column chromatography (*n*-hexane/ethyl acetate = 4/1) to give compound **S14** (5.7 g, 90%) as a colourless oil. $[\alpha]^{22}_D = -1.83$ (*c* 10, $CHCl_3$); IR (thin film): ν 2964, 2937, 1662, 1603, 1494, 1453, 1416, 1385, 1322, 1176, 1119, 1020, 996, 763, 701 cm^{-1}; ^1H NMR (600 MHz, $CDCl_3$): δ 7.29–7.23 (m, 4H; Ph-H), 7.19–7.16 (m, 1H; Ph-H), 3.56 (s, 3H; OCH_3), 3.37–3.33 (m, 1H; β-methine-H), 3.12 (s, 3H; CH_3), 2.73–2.70 (m, 1H; α-methylene-H), 2.65–2.61 (m, 1H; α-methylene-H), 1.30 (d, J = 6.9 Hz, 3H; CH_3); ^{13}C NMR (150 MHz, $CDCl_3$): δ 173.2 (C), 146.6 (C), 128.4 (CH × 2), 126.9 (CH × 2), 126.2 (CH), 61.2 (CH), 40.3 (CH_2), 35.8 (CH), 32.1 (CH), 21.6 (CH_3); HRMS (APCI): *m/z* calcd for $C_{12}H_{18}NO_2$ ($[M+H]^+$): 208.1338, found: 208.1342.

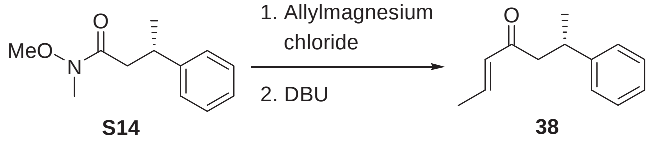

(*S,E*)-6-phenylhept-2-en-4-one (38). Allylmagnesium chloride (2.0 M solution in THF, 13.5 mL, 27.0 mmol) was added dropwise to a stirring solution of amide **S14** (5.1 g, 24.6 mmol) in THF (50 mL) at –78 °C under argon atmosphere. After 30 min at the same temperature, the reaction was quenched with satd. $NH_4Cl_{(aq)}$, extracted with ethyl acetate, dried over anhydrous $MgSO_4$, filtered and concentrated. The residue was dissolved in CH_2Cl_2 (50 mL), and DBU (1.9 mL, 12.3 mmol) was added at ambient temperature. After

stirring for 12 hours, the reaction was quenched with satd. $NH_4Cl_{(aq)}$, extracted with CH_2Cl_2, dried over anhydrous $MgSO_4$, filtered and concentrated. Purification of the crude product by flash column chromatography (*n*-hexane/ethyl acetate = 4/1) provided the title compound **38** (4.1 g, 88%) as a colourless oil. $[\alpha]^{22}_{D}$ = +19.8 (*c* 10, $CHCl_3$); IR (thin film): ν 3028, 2965, 1696, 1670, 1630, 1494, 1452, 1376, 1292, 1186, 970, 762 cm^{-1}; ^{1}H NMR (600 MHz, $CDCl_3$): δ 7.27 (t, *J* = 7.6 Hz, 2H; Ph-H), 7.20 (d, *J* = 7.0 Hz, 2H; Ph-H), 7.18–7.15 (m, 1H; Ph-H), 6.78 (dq, *J* = 16.2 6.8 Hz, 1H; β-vinylic H), 6.07 (ddd, *J* = 16.2, 3.2, 1.7 Hz, 1H; α-vinylic H), 3.36–3.30 (m, 1H; β-methine-H), 2.82 (dd, *J* = 15.8, 6.0 Hz, 1H; α-methylene-H), 2.73 (dd, *J* = 15.8, 8.3 Hz, 1H; α-methylene-H), 1.85 (dd, *J* = 6.9, 1.6 Hz, 3H; vinylic CH_3), 1.25 (d, *J* = 6.9 Hz, 3H; CH_3); ^{13}C NMR (150 MHz, $CDCl_3$): δ 199.2 (C), 146.5 (C), 142.7 (CH), 132.2 (CH), 128.5 (CH × 2), 126.8 (CH × 2), 126.2 (CH), 48.3 (CH_2), 35.6 (CH), 21.8 (CH_3), 18.2 (CH_3); HRMS (APCI): *m/z* calcd for $C_{13}H_{17}O$ ($[M + H]^+$): 189.1279, found: 189.1281.

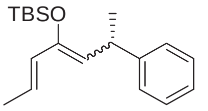

39

1-[(*S*,3*Z*,5*E*)/(*S*,3*E*,5*E*)-4-*tert*-butyldimethylsilyloxyhepta-3,5-dien-2-yl)benzene (39). Following the procedure similar to the preparation of compound **8**, LiHMDS (1.0 M solution in THF, 30 mL, 29.4 mmol) was slowly added to a solution of enone **38** (4.6 g, 24.5 mmol) in anhydrous THF (82 mL) at –78 °C under N_2 atmosphere. After 30 min of stirring, TBSOTf (98%, 6.3 mL, 26.9 mmol) was added dropwise. The reaction was stirred at the same temperature for 2 h, and then, quenched with satd. $NaHCO_{3(aq)}$, extracted with Et_2O, dried over anhydrous $MgSO_4$, filtered and concentrated under reduced pressure. Purification of the crude product by flash column chromatography (*n*-hexane/Et_3N = 95/5) provided the silyloxydiene **39** (6.06 g, 82%, (*Z*,*E*)/(*E*,*E*) = 70/30) as a colourless oil. For the (*Z*,*E*) isomer: IR (thin film): ν 3028, 2930, 2959, 2858, 1623, 1607, 1472 1360, 1254, 1018, 993, 826 cm^{-1}; ^{1}H NMR (600 MHz, C_6D_6): δ 7.30 (d, *J* = 7.5 Hz, 2H; Ph-H), 7.22–7.16 (m, 2H; Ph-H), 7.07–7.02 (m, 1H; Ph-H), 5.90–5.81 (m, 2H; vinylic H), 4.89 (d, *J* = 9.9 Hz, 1H; vinylic H), 4.08 (dq, *J* = 14.1, 7.0 Hz, 1H; methylene-H), 1.59 (d, *J* = 6.0 Hz, 3H; CH_3), 1.35 (d, *J* = 7.0 Hz, 3H; CH_3), 1.03 (s, 9H; Si-C$(CH_3)_3$), 0.13 (s, 3H, Si-CH_3), 0.08 (s, 3H, Si-CH_3); ^{13}C NMR (150 MHz, C_6D_6): δ 147.4 (C), 147.1 (C), 130.6 (CH), 128.7 (CH × 2), 127.3 (CH × 2), 126.1 (CH), 124.7 (CH), 117.8 (CH), 35.8 (CH), 26.1 (CH_3 × 3), 22.7 (CH_3), 18.6 (C), 17.6 (CH_3),

–3.4 (CH_3), –3.5 (CH_3); HRMS (ESI): *m/z* calcd for $C_{19}H_{31}O_2Si$ ($[M + H]^+$): 303.2144, found: 303.2148. For the (*E*,*E*) isomer: IR (thin film): ν 3028, 2930, 2959, 2858, 1623, 1607, 1472 1360, 1254, 1018, 993, 826 cm^{-1}; ^1H NMR (600 MHz, C_6D_6): δ 7.29 (d, *J* = 7.5 Hz, 2H; Ph-H), 7.21–7.17 (m, 2H; Ph-H), 7.05–7.02 (m, 1H; Ph-H), 6.37 (dd, *J* = 14.8, 1.1 Hz, 1H; vinylic H), 6.27–6.22 (m, 1H; vinylic H), 5.03 (d, *J* = 9.8 Hz, 1H; vinylic H), 3.75–3.70 (m, 1H; methylene-H), 1.64 (d, *J* = 6.6 Hz, 3H; CH_3), 1.31 (d, *J* = 6.9 Hz, 3H; CH_3), 1.00 (s, 9H; Si-$C(CH_3)_3$), 0.15 (s, 3H, Si-CH_3), 0.12 (s, 3H, Si-CH_3); ^{13}C NMR (150 MHz, C_6D_6): δ 147.2 (C), 147.1 (C), 128.7 (CH × 2), 127.2 (CH), 127.1 (CH × 2), 126.2 (CH), 125.0 (CH), 116.1 (CH), 36.9 (CH), 25.8 (CH_3 × 3), 23.2 (CH_3), 18.5 (C), 18.1 (CH_3), –4.20 (CH_3), –4.21 (CH_3); HRMS (ESI): *m/z* calcd for $C_{19}H_{31}O_2Si$ ($[M + H]^+$): 303.2144, found: 303.2148.

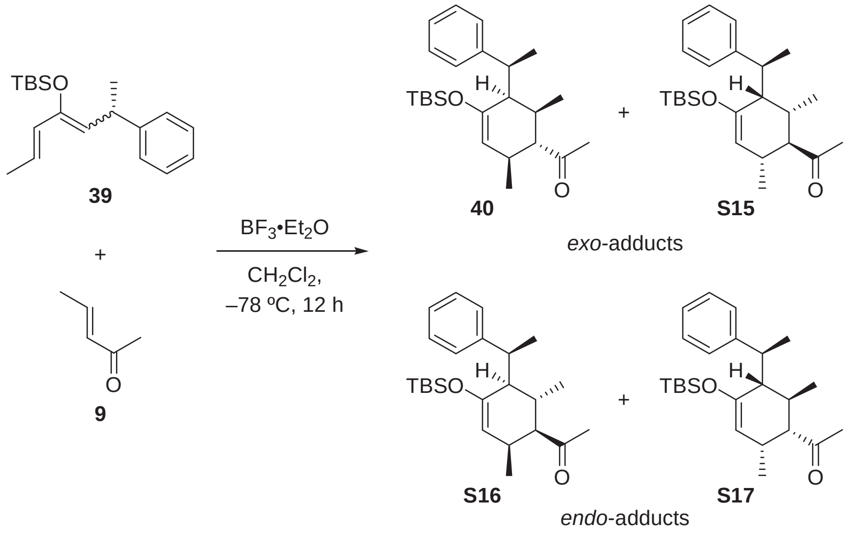

1-[4-*tert*-butyldimethylsilyloxy-2,6-dimethyl-5-(1-phenylethyl)cyclohex-3-enyl]ethanones (40, S15, S16, S17). Freshly distilled ketone **9** (2.1 mL, 21.38 mmol) was added by syringe to a solution of $BF_3 \cdot OEt_2$ (110 μL, 0.89 mmol) in CH_2Cl_2 (26 mL) under an argon atmosphere at –78 °C. After stirring at –78 °C for 10 min, diene **39** [(*Z*,*E*)/(*E*,*E*) = 70/30, 5.38 g, 17.8 mmol (based on the (Z,E) isomer)] in CH_2Cl_2 (10 mL) was added dropwise, and the resulting mixture was stirred at this temperature for 12 h. The reaction was quenched with Et_3N and satd. $NaHCO_{3(aq)}$. The crude organic portion was extracted with CH_2Cl_2, dried over anhydrous $MgSO_4$, filtered and concentrated under reduced pressure. The residue was purified by flash column chromatography (*n*-hexane/ethyl acetate = 20/1) to give the corresponding Diels-Alder cycloadducts **40**, **S15**, **S16** and **S17** (total: 4.34 g, 90%, *d.r.* = 72:12:6:10) as colourless oil. For compound **40**:$[\alpha]^{22}_D$ = –70.6 (*c* 5, $CHCl_3$); m.p. 64–65 °C (recrystallized from *n*-hexanes/$CHCl_3$); IR (thin film): ν 2958, 2930, 1711, 1664, 1457, 1359,

1252, 1189, 1176, 838, 778, 701 cm^{-1}; ^{1}H NMR (600 MHz, $CDCl_3$): δ 7.28–7.25 (m, 4H; Ar-H), 7.13 (tt, *J* = 6.8, 1.3 Hz, 1H; Ar-H), 4.68 (d, *J* = 2.8 Hz, 1H; H3), 2.89 (qd, *J* = 7.3, 4.5 Hz, 1H; H6), 2.52–2.49 (m, 1H; H2), 2.36 (dd, *J* = 4.5, 4.0 Hz, 1H; H5), 2.14–2.13 (m, 2H; H3, H4), 2.12 (s, 3H; $COCH_3$), 1.27 (d, *J* = 7.4 Hz, 3H; CH_3), 0.93 (s, 9H; Si-$C(CH_3)_3$), 0.91 (d, *J* = 6.8 Hz, 3H; CH_3), 0.76 (d, *J* = 6.8 Hz, 3H; CH_3), 0.16 (s, 3H; Si-CH_3), 0.12 (s, 3H; Si-CH_3); ^{13}C NMR (150 MHz, $CDCl_3$): δ 213.9 (C), 151.8 (C), 149.6 (C), 128.2 (CH × 2), 127.8 (CH × 2), 125.5 (CH), 108.7 (CH), 57.6 (CH), 50.1 (CH), 38.8 (CH), 37.1 (CH), 33.5 (CH), 30.7 (CH_3), 25.8 (CH_3 × 3), 21.8 (CH_3), 21.1 (CH_3), 18.1 (C), 16.9 (CH_3), –4.2 (CH_3), –4.5 (CH_3); HRMS (ESI): *m/z* calcd for $C_{24}H_{38}O_2SiNa$ ($[M+ Na]^+$): 409.2539, found: 409.2534. An X-ray analysis of the crystal of the racemic version of **40** supported the structure of the compound (Supplementary Fig. 1e, CCDC 1511850). For compound **S15**: IR (thin film): ν 2958, 1711, 1665, 1461, 1359, 1252, 1189, 1176, 838, 778 cm^{-1}; ^{1}H NMR (600 MHz, $CDCl_3$): δ 7.28–7.24 (m, 4H; Ar-H), 7.18–7.16 (m, 1H; Ar-H), 4.59 (d, *J* = 2.7 Hz, 1H; H3), 3.21 (qd, *J* = 7.3, 4.5 Hz, 1H; H6), 2.24–2.20 (m, 2H; H2, H4), 2.07–2.01 (m, 1H; H5), 1.91 (s, 3H; $COCH_3$), 1.60 (dd, *J* = 11.9, 9.7 Hz, 1H; H1), 1.46 (d, *J* = 7.5 Hz, 3H; Me3), 0.98 (d, *J* = 6.8 Hz, 3H; CH_3), 0.97 (s, 9H; Si-$C(CH_3)_3$), 0.36 (d, *J* = 6.8 Hz, 3H; CH_3), 0.19 (s, 6H; Si-$(CH_3)_2$); ^{13}C NMR (150 MHz, $CDCl_3$): δ 214.9 (C), 150.8 (C), 145.8 (C), 129.3 (CH × 2), 127.8 (CH × 2), 126.1 (CH), 111.3 (CH), 55.2 (CH), 50.4 (CH), 38.6 (CH), 36.6 (CH), 33.3 (CH), 32.5 (CH_3), 25.9 (CH_3 × 3), 24.2 (CH_3), 20.1 (CH_3), 18.1 (C), 16.8 (CH_3), –4.0 (CH_3), –4.6 (CH_3); HRMS (ESI): *m/z* calcd for $C_{24}H_{38}O_2SiNa$ ($[M+ Na]^+$): 409.2539, found: 409.2543. For compound **S16**: IR (thin film): ν 2958, 2930, 1711, 1666, 1461, 1359, 1252, 1189, 1176, 838 cm^{-1}; ^{1}H NMR (600 MHz, $CDCl_3$): δ 7.27–7.22 (m, 4H; Ar-H), 7.12 (tt, *J* = 6.8, 1.3 Hz, 1H; Ar-H), 4.77 (d, *J* = 4.2 Hz, 1H; H3), 2.90 (qd, *J* = 7.4, 4.5 Hz, 1H; H6), 2.81 (qd, *J* = 11.5, 6.4 Hz, 1H; H1), 2.65–2.60 (m, 1H; H2), 2.35 (dd, *J* = 4.6, 4.5 Hz, 1H; H4), 2.25–2.18 (m, 1H; H5), 2.13 (s, 3H; $COCH_3$), 1.28 (d, *J* = 7.3 Hz, 3H; CH_3), 0.91 (s, 9H; Si-$C(CH_3)_3$), 0.83 (d, *J* = 6.8 Hz, 3H; CH_3), 0.78 (d, *J* = 6.8 Hz, 3H; CH_3), 0.13 (s, 3H; Si-CH_3), 0.08 (s, 3H; Si-CH_3); ^{13}C NMR (150 MHz, $CDCl_3$): δ 210.6 (C), 151.9 (C), 149.9 (C), 128.2 (CH × 2), 127.7 (CH × 2), 125.4 (CH), 109.3 (CH), 52.4 (CH), 50.1 (CH), 38.3 (CH), 31.3 (CH), 30.3 (CH), 30.2 (CH_3), 25.8 (CH_3 × 3), 21.7 (CH_3), 18.2 (CH_3), 18.1 (C), 16.5 (CH_3), –4.2 (CH_3), –4.6 (CH_3); HRMS (ESI): *m/z* calcd for $C_{24}H_{38}O_2SiNa$ ($[M + Na]^+$): 409.2539, found: 409.2544. For compound **S17**: IR (thin film): ν 2958, 2930, 1711, 1666, 1471, 1462, 1359, 1252, 1189, 1176, 838, 778 cm^{-1}; ^{1}H NMR (600 MHz, $CDCl_3$): δ 7.26–7.23 (m, 4H; Ar-H), 7.17–7.14 (m, 1H; Ar-H), 4.74 (d, *J* = 3.7 Hz, 1H; H3), 2.90 (qd, *J* = 7.0, 4.2 Hz, 1H;

H6), 2.21–2.19 (m, 1H; H4), 2.14–2.11 (m, 1H; H5), 2.09–2.07 (m, 1H; H2), 1.76 (s, 3H; $COCH_3$), 1.61 (dd, J = 11.8, 9.5 Hz, 1H; H1), 1.43 (d, J = 6.5 Hz, 3H; CH_3), 0.91 (s, 9H; Si-$C(CH_3)_3$), 0.98 (d, J = 6.8 Hz, 3H; CH_3), 0.63 (d, J = 7.2 Hz, 3H; CH_3), 0.18 (s, 3H; Si-CH_3), 0.15 (s, 3H; Si-CH_3); ^{13}C NMR (150 MHz, $CDCl_3$): δ 210.9 (C), 150.9 (C), 145.9 (C), 128.9 (CH × 2), 127.7 (CH × 2), 125.9 (CH), 111.3 (CH), 51.2 (CH), 50.9 (CH), 38.6 (CH), 31.0 (CH), 30.7 (CH), 29.8 (CH_3), 25.8 (CH_3 × 3), 23.5 (CH_3), 18.1 (C), 17.9 (CH_3), 16.4 (CH_3), –4.7 (CH_3 × 2); HRMS (ESI): m/z calcd for $C_{24}H_{38}O_2SiNa$ ($[M + Na]^+$): 409.2539, found: 409.2540.

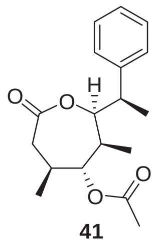

41

(2*S*,3*S*,4*R*,5*S*)-3,5-dimethyl-7-oxo-2-[(*R*)-1-phenylethyl]oxepan-4-yl acetate (41). *Alternative method*: To a solution of the *exo*-adduct **40** (0.560 g, 1.45 mmol) in CH_2Cl_2 (3 mL) was added TFA (0.13 mL, 1.74 mmol) dropwise at 0 °C. After stirring for 20 minutes, the reaction mixture was diluted with CH_2Cl_2 (14.5 mL), and *m*CPBA (77%, 2.6 g, 11.6 mmol) was added in one portion. The resultant mixture was allowed to warm to ambient temperature and stirred for 24 hours. After this time, the reaction mixture was slowly quenched with satd. $Na_2S_2O_{3(aq)}$ at 0 °C, and the crude organic portion was extracted with CH_2Cl_2. The combined organic layer was washed with satd. $NaHCO_{3(aq)}$ and brine, dried over $MgSO_4$, filtered and concentrated under reduced pressure. Purification by flash column chromatography (*n*-hexane/ethyl acetate = 4/1) provided the ε-lactone **41** (0.33 g, 75%) as a colourless solid.

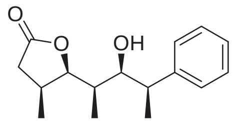

42

(4*S*,5*R*)-dihydro-5-[(2*R*,3*S*,4*R*)-3-hydroxy-4-phenylpentan-2-yl]-4-methylfuran-2(3*H*)-one (42). To a solution of ε-lactone **41** (1.4 g, 4.61 mmol) in MeOH (15 mL) was added NaOMe (0.3 g, 5.53 mmol) at 0 °C. The resulting mixture was allowed to warm to room temperature. Upon consumption of starting material as indicated by TLC analysis

(about 6 h), the reaction was neutralized with DOWEX 50 WX2-200 IR-resin. The mixture was filtered through sintered glass, and the filtrate was concentrated under reduced pressure. Purification by flash column chromatography (*n*-hexane/ethyl acetate = 1/2) gave the alcohol **42** (1.11 g, 92%) as a colourless solid. $[\alpha]^{22}_{D} = -13.0$ (*c* 0.5, $CHCl_3$); m.p. 142–143 °C (recrystallized from *n*-hexanes/$CHCl_3$); IR (thin film): ν 2962, 2927, 1763, 1735, 1646, 1454, 1221, 1183, 1157, 992, 973, 763 cm^{-1}; ^1H NMR (600 MHz, $CDCl_3$): δ 7.28 (t, *J* = 7.5 Hz, 2H; Ph-H), 7.20 (t, *J* = 7.4 Hz, 1H; Ph-H), 7.15 (d, *J* = 7.8 Hz, 2H; Ph-H), 4.37 (dd, *J* = 9.9, 4.6 Hz, 1H; γ-methine-H), 3.61 (dd, *J* = 9.9, 8.5 Hz, 1H; ε-methine-H), 2.84 (dq, *J* = 13.8, 6.9 Hz, 1H; benzylic H), 2.67 (dd *J* = 16.9, 7.3 Hz, 1H; α-methylene-H), 2.58–2.54 (m, 1H; β-methine-H), 2.10 (d, *J* = 16.9 Hz, 1H; α-methylene-H), 1.61–1.57 (m, 1H; δ-methine-H), 1.38 (d, *J* = 6.8 Hz, 3H; CH_3), 1.05 (d, *J* = 6.7 Hz, 3H; CH_3), 0.60 (d, *J* = 7.0 Hz, 3H; CH_3); ^{13}C NMR (150 MHz, $CDCl_3$): δ 176.6 (C), 143.4 (C), 128.8 (CH × 2), 127.4 (CH × 2), 126.9 (CH), 86.0 (CH), 76.1 (CH), 43.7 (CH), 38.9 (CH_2), 34.6 (CH), 31.9 (CH), 18.8 (CH_3), 13.1 (CH_3), 9.0 (CH_3); HRMS (FAB): *m/z* calcd for $C_{16}H_{23}O_3$ ($[M + H]^+$): 263.1647, found: 263.1648. An X-ray analysis of the crystal supported the structure of **42** and the relative configuration of its contiguous chiral centres (Fig. 4c, CCDC 1556034).

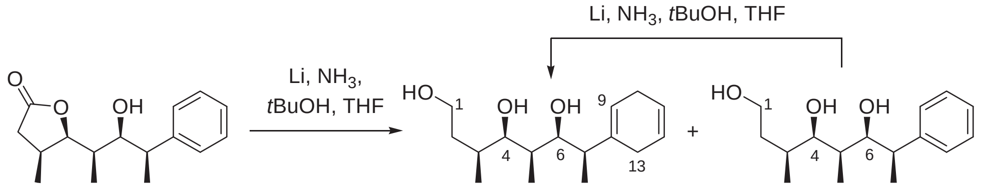

(The numberings in the carbon skeletons are guides for NMR peak assignment only.)

(3*S*,4*R*,5*S*,6*S*,7*R*)-7-(cyclohexa-1,4-dienyl)-3,5-dimethyloctan-1,4,6-triol (S18) and (3*S*,4*R*,5*S*,6*S*,7*R*)-3,5-dimethyl-7-phenyloctan-1,4,6-triol (S19). According to the known procedure[3], a solution of lactone **42** (50 mg, 0.191 mmol, 1.0 equiv) in 2 mL of THF was added to a solution of lithium (66 mg, 9.54 mmol, 50.0 equiv) in 9.5 mL of ammonia at –78 °C. The mixture was stirred at –40 °C for 45 min, cooled to –78 °C, and treated with *tert*-butanol (0.12 mL). The blue solution was stirred for 1 h and treated sequentially with *tert*-butanol (0.24 mL), isoprene (0.95 mL) and satd. $NH_4Cl_{(aq)}$ (5 mL). Ammonia was allowed to evaporate through a bubbler, and the residue was partitioned between brine (5 mL) and ethyl acetate (10 mL). After extraction with ethyl acetate, the combined organic layers was dried over anhydrous $MgSO_4$, filtered and concentrated under reduced pressure to afford a colourless oil. The crude material was purified by flash column chromatography (*n*-hexane/

ethyl acetate = 2/1 to 1/2) to give compounds **S18** (27 mg, 52%) and **S19** (22 mg, 43%) as colourless oils. Triol **S19** (110 mg, 0.413 mmol) was further subjected to the same Birch reduction to afford compound **S18** (38 mg, 34%) and recovered the starting material **S19** (68 mg, 62%). For **S18**: $[\alpha]^{27}_{D}$ = +20.5 (*c* 0.3, benzene); IR (thin film): ν 3364, 2966, 2931, 1455, 1428, 1055, 959, 702, 667 cm^{-1}; ^{1}H NMR (600 MHz, $CDCl_3$): δ 5.66 (s, 2H; H^{11} and H^{12}), 5.48 (s, 1H; H^{9}), 3.71 (dt, *J* = 10.7, 6.3 Hz, 1H; H^{1a}), 3.66–3.62 (m, 2H; H^{6} and H^{1b}), 3.48 (dd, *J* = 7.2, 3.3 Hz, 1H; H^{4}), 2.69–2.63 (m, 3H; H^{10ab} and H^{13a}), 2.42–2.39 (m, 1H; H^{13b}), 2.25 (dq, *J* = 9.8, 6.8 Hz, 1H; H^{7}), 1.84–1.77 (m, 2H; H^{5} and H^{3}), 1.64–1.58 (m, 1H; H^{2a}), 1.33 (ddt, *J* = 14.2, 8.2, 6.2 Hz, 1H; H^{2b}), 1.10 (d, *J* = 6.8 Hz; 3H, CH_3), 0.95 (d, *J* = 6.7 Hz, 3H; CH_3), 0.89 (d, *J* = 6.9 Hz, 3H; CH_3); ^{13}C NMR (150 MHz, $CDCl_3$): δ 136.9 (C), 124.4 (CH), 123.9 (CH), 120.3 (CH), 80.8 (CH), 78.8 (CH), 60.4 (CH_2), 45.0 (CH), 36.0 (CH_2), 35.9 (CH), 33.3 (CH_2), 26.6 (CH), 25.9 (CH_2), 16.3 (CH_3), 15.0 (CH_3), 5.4 (CH_3); HRMS (APCI): *m/z* calcd for $C_{16}H_{29}O_3$ ($[M + H]^+$): 269.2117, found: 269.2114. For **S19**: $[\alpha]^{28}_{D}$ = +54.5 (*c* = 1, $CHCl_3$); m.p. 97–99 °C; IR (thin film): ν 3359, 2966, 2929, 1493, 1453, 1380, 1139, 1053, 977, 761, 702 cm^{-1}; ^{1}H NMR (600 MHz, $CDCl_3$): δ 7.27 (t, *J* = 7.5 Hz, 2H; Ph-H), 7.18 (t, *J* = 7.4 Hz, 1H; Ph-H), 7.13 (d, *J* = 7.5 Hz, 2H; Ph-H), 3.83 (d, *J* = 10.0 Hz, 1H; H^{6}), 3.58–3.49 (m, 2H; H^{1a} and H^{1b}), 3.42 (dd, *J* = 16.9, 3.5 Hz, 1H; H^{4}), 2.81 (dq, *J* = 13.9, 6.9 Hz, 1H; H^{7}), 1.73–1.68 (m, 1H; H^{5}), 1.44–1.42 (m, 1H; H^{3}), 1.39–1.33 (m, 1H; H^{2a}), 1.34 (d, *J* = 6.7 Hz; 3H, CH_3), 1.19–1.13 (m, 1H; H^{2b}), 0.88 (d, *J* = 6.8 Hz; 3H, CH_3), 0.82 (d, *J* = 6.7 Hz; 3H, CH_3); ^{13}C NMR (150 MHz, $CDCl_3$): δ 144.5 (C), 128.6 (CH × 2), 127.4 (CH × 2), 126.4 (CH), 81.2 (CH), 80.5 (CH), 60.2 (CH_2), 43.8 (CH), 35.9 (CH_2), 35.6 (CH), 33.1 (CH), 19.3 (CH_3), 14.6 (CH_3), 5.4 (CH_3); HRMS (APCI): *m/z* calcd for $C_{16}H_{27}O_3$ ($[M + H]^+$): 267.1960, found: 267.1961.

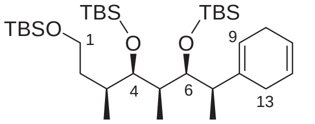

43

(The numbering in the carbon skeleton is a guide for NMR peak assignment only.)

1-[(2*R*,3*S*,4*S*,5*R*,6*S*)-3,5,8-tri-*tert*-butyldimethylsilyloxy-4,6-dimethyloctan-2-yl]cyclohexa-1,4-diene (43). To a solution of triol **S18** (10 mg, 0.037 mmol) and 2,6-lutidine (35 μL, 0.299 mmol) in CH_2Cl_2 (0.5 mL) was added TBSOTf (98%, 35 μL, 0.149 mmol) at 0 °C under Ar atmosphere. The mixture was allowed to warm to room temperature and was stirred for 8 h. The reaction was quenched with satd. $NaHCO_{3(aq)}$ followed by extraction with

CH_2Cl_2. The combined organic layers was dried over anhydrous $MgSO_4$, filtered and concentrated under reduced pressure. The crude material was purified by flash column chromatography (*n*-hexane) to give the compound **43** (21 mg, 94%) as a colourless oil. $[\alpha]^{27}_D$ = +3.5 (*c* 0.3, benzene); IR (thin film): ν 2956, 2930, 2857, 1427, 1462, 1386, 1360, 1256, 1096, 1028, 1004, 834, 771, 667 cm^{-1}; ^1H NMR (600 MHz, C_6D_6): δ 5.69 (d, J = 10.3 Hz, 1H; H^{11}), 5.62 (d, J = 10.3 Hz, 1H; H^{12}), 5.44 (br, 1H; H^9), 3.77 (d, J = 8.1 Hz, 1H; H^6), 3.69–3.66 (m, 2H; H^4 and H^{1a}), 3.62 (ddd, J = 9.8, 9.7, 4.6 Hz, 1H; H^{1b}), 2.77–2.71 (m, 1H; H^{13a}), 2.57–2.149 (m, 3H; H^{10a}, H^{10b} and H^{13b}), 2.36–2.31 (m, 1H; H^7), 2.14–2.09 (m, 1H; H^3 and H^5), 1.82–1.77 (m, 1H; H^{2a}), 1.61–1.54 (m, 1H; H^{2b}), 1.17 (d, J = 6.9 Hz; 3H, CH_3), 1.14 (d, J = 6.9 Hz, 3H; CH_3), 1.05 (s, 9H; Si-$C(CH_3)_3$), 1.03 (s, 9H; Si-$C(CH_3)_3$), 1.00 (s, 9H; Si-$C(CH_3)_3$), 0.987 (d, J = 6.6 Hz, 3H; CH_3), 0.22 (s, 3H; $SiCH_3$), 0.21 (s, 3H; $SiCH_3$), 0.18 (s, 3H; $SiCH_3$), 0.16 (s, 3H; $SiCH_3$) , 0.09 (s, 6H; $SiCH_3$ × 2); ^{13}C NMR (150 MHz, C_6D_6): δ 137.9 (C), 124.6 (CH), 124.3 (CH), 120.5 (CH), 78.2 (CH), 75.4 (CH), 61.1 (CH_2), 46.1 (CH), 40.1 (CH), 39.3 (CH_2), 32.1 (CH), 27.7 (CH_2), 27.0 (CH_2), 26.6 (CH_3 × 6), 26.2 (CH_3 × 3), 18.9 (C), 18.8 (C), 18.5 (C), 17.2 (CH_3), 11.8 (CH_3), 11.5 (CH_3), –2.5 (CH_3), –2.6 (CH_3), –2.9 (CH_3), –3.1 (CH_3), –5.2 (CH_3), –5.3 (CH_3); HRMS (APCI): *m/z* calcd for $C_{34}H_{71}O_3Si_3$ ($[M + H]^+$): 611.4711, found: 611.4713.

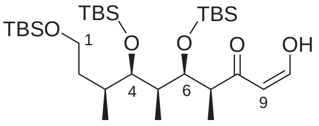

44

(The numbering in the carbon skeleton is a guide for NMR peak assignment only.)

***(Z*,4*S*,5*R*,6*S*,7*R*,8*S*)-5,7,10-tri-*tert*-butyldimethylsilyloxy-1-hydroxy-4,6,8-trimethyldec-1-en-3-one (44).** According to the known procedure[3], a solution of diene **43** (90 mg, 0.148 mmol) in CH_2Cl_2 (1.2 mL) and MeOH (0.3 mL) was added pyridine (7 μL). The mixture was treated with a dilute stream of ozone in oxygen at –78 °C until it turned light blue, at which point dimethyl sulfide (0.21 mL) was added. The mixture was stirred at room temperature for 12 h. The reaction was partitioned between brine and ethyl acetate followed by extraction with ethyl acetate. The combined organic layers was dried over anhydrous $MgSO_4$, filtered and concentrated under reduced pressure. Purification by flash column chromatography (*n*-hexane/ethyl acetate = 1/0 to 10/1) gave compound **44** (78 mg, 88%) as a light yellow oil. $[\alpha]^{28}_D$ = +14.3 (*c* 2.0, $CHCl_3$); IR (thin film): ν 2930, 2857, 1598, 1462, 1387, 1255, 1094, 1030, 1004, 834, 772 cm^{-1}; ^1H NMR (600 MHz, $CDCl_3$): δ 8.04 (d,

J = 4.1 Hz, 1H; β-vinyl-H), 5.55 (d, J = 4.1 Hz, 1H; α-vinyl-H), 3.90 (dd, J = 5.7, 4.1 Hz, 1H; H^6), 3.67 (ddd, J = 10.3, 7.1, 4.6 Hz, 1H; H^{1a}), 3.59 (ddd, J = 10.1, 8.4, 6.1 Hz, 1H; H^{1b}), 3.44 (dd, J = 7.0, 2.3 Hz, 1H; H^4), 2.57–2.54 (m, 1H; H^7), 1.83–1.79 (m, 1H; H^5), 1.73–1.70 (m, 1H; H^3), 1.64–1.59 (m, 1H; H^{2a}), 1.39–1.34 (m, 1H; H^{2b}), 1.10 (d, J = 7.0 Hz; 3H, CH_3), 0.88 (s, 9H; Si-$C(CH_3)_3$), 0.87 (s, 18H; Si-$C(CH_3)_3$ × 2), 0.86 (d, J = 7.0 Hz, 3H; CH_3), 0.75 (d, J = 7.0 Hz, 3H; CH_3), 0.05 (s, 3H; $SiCH_3$), 0.04 (s, 3H; $SiCH_3$), 0.03 (s, 3H; $SiCH_3$), 0.02 (s, 6H; $SiCH_3$ × 2), –0.02 (s, 3H; $SiCH_3$); ^{13}C NMR (150 MHz, $CDCl_3$): δ 200.5 (C), 177.6 (CH), 101.8 (CH), 77.2 (CH), 74.2 (CH), 61.4 (CH_2), 47.7 (CH), 40.7 (CH), 38.0 (CH_2), 33.1 (CH), 26.3 (CH_3 × 3), 26.2 (CH_3 × 3), 26.0 (CH_3 × 3), 18.6 (C), 18.5 (C), 18.3 (C), 13.1 (CH_3), 12.7 (CH_3), 11.9 (CH_3), –3.24 (CH_3), –3.32 (CH_3), –3.38 (CH_3), –3.93 (CH_3), –5.23 (CH_3), –5.33 (CH_3); HRMS (APCI): *m/z* calcd for $C_{31}H_{67}O_5Si_3$ ($[M + H]^+$): 603.4296, found: 603.4290.

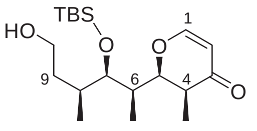

45

(The numbering in the carbon skeleton is a guide for NMR peak assignment and matches the carbon numbering of the etnangien fragment as specified in the main text.)

(2*R*,3*S*)-2-[(2*S*,3*R*,4*S*)-3-*tert*-butylsilyloxy-6-hydroxy-4-methylhexan-2-yl]-2,3-dihydro-3-methylpyran-4-one (45). HF·pyridine (70%, 16 μL, 0.598 mmol) was added to a solution of enone **44** (60 mg, 0.099 mmol) in pyridine (2.0 mL) at 0 °C. The mixture was allowed to warm to room temperature and stirred for 36 h. The reaction was slowly quenched with silica gel, and the crude material was poured onto a silica gel column (*n*-hexane/ethyl acetate = 4/1 to 1/1) to give compound **45** (33 mg, 92%) as a colourless oil. $[\alpha]^{27}_D$ = +10.1 (*c* 1.0, $CHCl_3$); IR (thin film): ν 2931, 2885, 2857, 1668, 1595, 1462, 1407, 1255, 1226, 1255, 1226, 1098, 1034, 835, 773 cm^{-1}; ^{1}H NMR (600 MHz, $CDCl_3$): δ 7.37 (d, J = 5.8 Hz, 1H; H^1), 5.33 (dd J = 5.8, 0.9 Hz, 1H; H^2), 4.17 (dd, J = 9.4, 2.6 Hz, 1H; H^5), 3.72–3.69 (m, 1H; H^{10a}), 3.62–3.59 (m, 1H; H^{10b}), 3.46 (dd, J = 5.4, 1.7 Hz, 1H; H^7), 2.46–2.42 (m, 1H; H^4), 2.13–2.09 (m, 1H; H^6), 1.83–1.76 (m, 2H; H^8 and H^{9a}), 1.35–1.30 (m, 1H; H^{9b}), 1.16 (d, J = 6.7 Hz; 3H, CH_3), 1.05 (d, J = 7.3 Hz, 3H; CH_3), 0.92 (d, J = 6.9 Hz, 3H; CH_3), 0.87 (s, 9H; Si-$C(CH_3)_3$), 0.046 (s, 3H; $SiCH_3$), –0.004 (s, 3H; $SiCH_3$); ^{13}C NMR (150 MHz, $CDCl_3$): δ 197.5 (C), 163.4 (CH), 105.5 (CH), 83.8 (CH), 74.9 (CH), 61.4 (CH_2), 42.4 (CH), 36.5 (CH), 36.2 (CH_2), 35.1 (CH), 26.0 (CH_3 × 3), 18.4 (C), 16.4 (CH_3), 10.4 (CH_3),

9.8 (CH_3), –3.69 (CH_3), –3.89 (CH_3); HRMS (APCI): *m/z* calcd for $C_{19}H_{37}O_4Si$ ($[M + H]^+$): 357.2461, found: 357.2461.

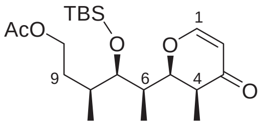

46

(The numbering in the carbon skeleton is a guide for NMR peak assignment and matches the carbon numbering of the etnangien fragment as specified in the main text.)

(3*S*,4*R*,5*S*)-4-*tert*-butyldimethylsilyloxy-5-[(2*R*,3*S*)-3,4-dihydro-3-methyl-4-oxo-2H-pyran-2-yl]-3-methylhexyl acetate (46). Acetic anhydride (16 µL, 0.169 mmol) was added to a mixture of pyranone **45** (20 mg, 0.056 mmol), trimethylamine (50 µL, 0.337 mmol) and DMAP (1.0 mg, 0.008 mmol) in CH_2Cl_2 (0.7 mL) at 0 °C. The mixture was allowed to warm to room temperature and stirred for 8 h. The reaction was quenched with satd. $NaHCO_{3(aq)}$ followed by extraction with CH_2Cl_2. The combined organic layers was dried over anhydrous $MgSO_4$, filtered and concentrated under reduced pressure. The crude material was purified by flash column chromatography (*n*-hexane/ethyl acetate = 6/1 to 2/1) to give compound **46** (22 mg, 98%) as a colourless oil. $[\alpha]^{27}_D$ = +13.6 (*c* 0.8, $CHCl_3$); IR (thin film): ν 2955, 2857, 1741, 1677, 1597, 1461, 1364, 1251, 1226, 1098, 1039, 836, 774 cm^{-1}; ^1H NMR (600 MHz, $CDCl_3$): δ 7.37 (d, J = 5.9 Hz, 1H; H^1), 5.33 (d J = 5.8 Hz, 1H; H^2), 4.16 (dd, J = 9.5, 2.4 Hz, 1H; H^5), 4.13–4.10 (m, 1H; H^{10a}), 4.04–4.00 (m, 1H; H^{10b}), 3.46 (dd, J = 5.5, 1.4 Hz, 1H; H^7), 2.42 (dd, J = 7.3, 1.9 Hz, 1H; H^4), 2.11–2.06 (m, 1H; H^6), 2.02 (s, 3H; CH_3), 1.93–1.88 (m, 1H; H^{9a}), 1.77–1.73 (m, 1H; H^8), 1.38–1.32 (m, 1H; H^{9b}), 1.05 (d, J = 6.8 Hz; 6H, $CH_3 \times 2$), 0.92 (d, J = 6.9 Hz, 3H; CH_3), 0.86 (s, 9H; Si-C(CH_3)$_3$), 0.04 (s, 3H; $SiCH_3$), –0.01 (s, 3H; $SiCH_3$); ^{13}C NMR (150 MHz, $CDCl_3$): δ 197.4 (C), 171.1 (C), 163.4 (CH), 105.5 (CH), 83.7 (CH), 74.6 (CH), 63.0 (CH_2), 42.3 (CH), 36.3 (CH), 35.4 (CH), 31.7 (CH_2), 26.0 ($CH_3 \times 3$), 21.0 (CH_3), 18.4 (C), 16.1 (CH_3), 10.4 (CH_3), 9.8 (CH_3), –3.8 (CH_3), –4.0 (CH_3); HRMS (APCI): *m/z* calcd for $C_{21}H_{39}O_5Si$ ($[M + H]^+$): 399.2567, found: 399.2562.

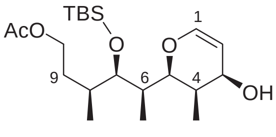

47

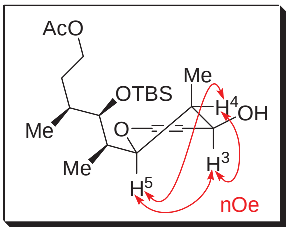

(The numbering in the carbon skeleton is a guide for NMR peak assignment and matches the carbon numbering of the etnangien fragment as specified in the main text.)

(3*S*,4*R*,5*S*)-4-*tert*-butyldimethylsilyloxy-5-[(2*S*,3*R*,4*R*)-3,4-dihydro-4-hydroxy-3-methyl-2*H*-pyran-2-yl]-3-methylhexyl acetate (47). According to the known procedure[4], $CeCl_3 \cdot 7H_2O$ (14 mg, 0.038 mmol) was added to a solution of pyranone **46** (10 mg, 0.025 mmol) in CH_2Cl_2 (0.6 mL) and MeOH (0.6 mL) and stirred at room temperature for 10 min under Ar atmosphere. The mixture was cooled to –20 °C, and then $NaBH_4$ (1.2 mg, 0.033 mmol) was slowly added. After stirring at –20 °C for 10 min, the reaction was quenched with satd. $NH_4Cl_{(aq)}$ followed by extraction with CH_2Cl_2. The combined organic layers was dried over anhydrous $MgSO_4$, filtered and concentrated under reduced pressure. The crude material was purified by flash column chromatography (*n*-hexane/ethyl acetate = 6/1 to 2/1) to give the compound **47** (single diastereomer, 9.5 mg, 95%) as a colorless oil. $[\alpha]^{27}_D$ = +18.8 (*c* 1.4, benzene); IR (thin film): ν 2930, 2857, 1742, 1654, 1462, 1366, 1251, 1227, 1103, 1046, 836, 773 cm^{-1}; ^1H NMR (600 MHz, C_6D_6): δ 6.23 (dd, J = 6.1, 1.5 Hz, 1H; H^1), 4.53 (d, J = 6.3 Hz, 1H; H^3), 4.43 (d, J = 6.1 Hz, 1H; H^2), 4.07 (ddd, J = 11.1, 7.3, 5.3 Hz, 1H; H^{10a}), 3.98 (ddd, J = 11.1, 7.9, 6.7 Hz, 1H; H^{10b}), 3.81 (d, J = 9.8 Hz, 1H; H^5), 3.46 (dd, J = 5.3, 0.8 Hz, 1H; H^7), 2.13–2.09 (m, 1H; H^4), 1.99–1.94 (m, 1H; H^6), 1.91–1.86 (m, 1H; H^{9a}), 1.69 (s, 3H; CH_3), 1.70–1.63 (m, 1H; H^8), 1.20–1.15 (m, 1H; H^{9b}), 1.18 (d, J = 6.7 Hz; 3H, CH_3), 0.95 (d, J = 6.8 Hz, 3H; CH_3), 0.93 (s, 9H; Si-$C(CH_3)_3$), 0.77 (d, J = 6.9 Hz, 3H; CH_3), –0.01 (s, 3H; $SiCH_3$), –0.06 (s, 3H; $SiCH_3$); ^{13}C NMR (150 MHz, C_6D_6): δ 170.1 (C), 144.9 (CH), 104.4 (CH), 80.4 (CH), 75.0 (CH), 66.9 (CH), 62.9 (CH_2), 37.1 (CH), 35.9 (CH), 33.6 (CH), 32.1 (CH_2), 26.2 ($CH_3 \times 3$), 20.5 (CH_3), 18.5 (C), 16.3 (CH_3), 11.2 (CH_3), 5.1 (CH_3), –3.7 (CH_3), –4.2 (CH_3); HRMS (ESI): *m/z* calcd for $C_{21}H_{40}O_5NaSi$ ($[M + Na]^+$): 423.2543, found: 423.2540.

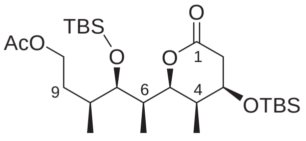

48

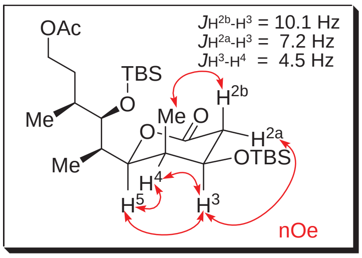

(The numbering in the carbon skeleton is a guide for NMR peak assignment and matches the carbon numbering of the etnangien fragment as specified in the main text.)

(3*S*,4*R*,5*S*)-4-*tert*-butyldimethylsilyloxy-5-[(2*S*,3*R*,4*R*)-4-*tert*-butyldimethylsilyloxy-tetrahydro-3-methyl-6-oxo-2*H*-pyran-2-yl]-3-methylhexyl acetate (48). TBSOTf (98%, 8 μL, 0.033 mmol) was added to a solution of compound **47** (10 mg, 0.025 mmol) and 2,6-lutidine (8 μL, 0.065 mmol) in CH_2Cl_2 (0.5 mL) at 0 °C under Ar atmosphere. The mixture was allowed to warm to room temperature and stirred for 8 h. The reaction was quenched with satd. $NaHCO_{3(aq)}$ followed by extraction with CH_2Cl_2. The combined organic layers was dried over anhydrous $MgSO_4$, filtered and concentrated under reduced pressure. The crude material was purified by flash column chromatography (*n*-hexane/ethyl acetate = 20/1 to 10/1) to give the di-O-silylated enol ether (12 mg) as a colourless oil, which was used in the next step according to the known procedure[5]. Pyridinium chlorochromate (17 mg, 0.093 mmol) was added to a solution of the enol ether (10 mg, 0.023 mmol) in 1,2-dichloroethane (0.5 mL). The mixture was stirred at room temperature for 6 h and the solvent was removed under reduced pressure. Purification by flash column chromatography (*n*-hexane/ethyl acetate = 7/1) provided the lactone **48** (12 mg, 92% in 2 steps) as a colourless oil. $[\alpha]^{28}_{D} = +17.4$ (*c* 1.4, benzene); IR (thin film): ν 2955, 2929, 2857, 1742, 1462, 1362, 1232, 1098, 1034, 836, 774 cm^{-1}; ^{1}H NMR (600 MHz, C_6D_6): δ 4.06 (ddd, *J* = 11.0, 7.3, 5.3 Hz, 1H; H^{10a}), 3.99–3.96 (m, 1H; H^{10b}), 3.94 (dd, *J* = 9.2, 1.9 Hz, 1H; H^5), 3.81 (ddd, *J* = 10.1, 7.2, 4.5 Hz, 1H; H^3), 3.45 (dd, *J* = 5.3, 1.6 Hz, 1H; H^7), 2.57 (dd, *J* = 18.3, 7.2 Hz, 1H; H^{2a}), 2.37 (dd, *J* = 18.3, 10.1 Hz, 1H; H^{2b}), 2.11–2.06 (m, 1H; H^4), 1.88–1.80 (m, 1H; H^6 and H^{9a}), 1.71 (s, 3H; CH_3), 1.67–1.63 (m, 1H; H^8), 1.19–1.14 (m, 1H; H^{9b}), 1.17 (d, *J* = 6.6 Hz; 6H, CH_3), 0.94 (s, 9H; Si-$C(CH_3)_3$), 0.89 (s, 9H; Si-$C(CH_3)_3$), 0.78 (d, *J* = 7.0 Hz, 3H; CH_3), 0.76 (d, *J* = 6.8 Hz, 3H; CH_3), –0.01 (s, 3H; $SiCH_3$), –0.03 (s, 3H; $SiCH_3$), –0.05 (s, 3H; $SiCH_3$), –0.07 (s, 3H; $SiCH_3$); ^{13}C NMR (150 MHz, C_6D_6): δ 170.1 (C), 168.6 (C), 81.1 (CH), 75.3 (CH), 68.2 (CH), 62.8 (CH_2), 38.2 (CH), 36.8 (CH_2), 36.3 (CH_2), 35.3 (CH), 32.3 (CH_2), 26.2 ($CH_3 \times 3$), 25.8 ($CH_3 \times 3$), 20.5 (CH_3), 18.5 (C), 17.9 (C), 16.2 (CH_3), 11.3 (CH_3), 5.1 (CH_3), –3.7 (CH_3), –3.8 (CH_3) , –4.6 (CH_3), –4.8 (CH_3); HRMS (ESI): *m/z* calcd for $C_{27}H_{54}O_6NaSi_2$ ($[M + Na]^+$): 553.3357, found: 553.3356.

Supplementary References

1. LeFort, F. M., Mishra, V., Dexter, G. D., Morgan, T. D. R. & Burnell, D. J. Nazarov reactions intercepted by (4 + 3) cycloadditions with oxygen-substituted dienes. *J. Org. Chem.* **80**, 5877–5886 (2015).

2. Lee, C. E., Kick, E. K. & Ellman, J. A. General solid-phase synthesis approach to prepare mechanism-based aspartyl protease inhibitor libraries. Identification of potent cathepsin D inhibitors. *J. Am. Chem. Soc.* **120**, 9735–9747 (1998).

3. Evans, D. A., Gauchet-Prunet, J. A., Carreira, E. M. & Charette, A. B. Synthesis of 1,3-diol synthons from epoxy aromatic precursors: an approach to the construction of polyacetate-derived natural products. *J. Org. Chem.* **56**, 741–750 (1991).

4. Chaładaj, W., Kowalczyk, R. & Jurczak, J. Enantioselective construction of *cis*-2,6-disubstituted dihydropyrans: total synthesis of (−)-centrolobine. *J. Org. Chem.* **75**, 1740–1743 (2010).

5. Baba, T., Huang, G. & Isobe, M. Synthesis of the JKLM-ring fragment of ciguatoxin. *Tetrahedron* **59**, 6851–6872 (2003).
