# Supplementary material for: Stereoselective one-pot synthesis of polypropionates
Source: Nat Commun. 2017 Sep 25;8:679. doi: 10.1038/s41467-017-00787-y (PMC5612996; doi:10.1038/s41467-017-00787-y)
Supplement: Supplementary file 3 — Description of Additional Supplementary Files [file 41467_2017_787_MOESM3_ESM.pdf]

Description of Additional Supplementary Files

File Name: Supplementary Data 1:
Description: Crystallographic information file for compound 11

File Name: Supplementary Data 2
Description: Crystallographic information file for compound 16

File Name: Supplementary Data 3
Description: Crystallographic information file for compound 23

File Name: Supplementary Data 4
Description: Crystallographic information file for compound 29

File Name: Supplementary Data 5
Description: Crystallographic information file for compound 34

File Name: Supplementary Data 6
Description: Crystallographic information file for compound 40

File Name: Supplementary Data 7
Description: Crystallographic information file for compound 41

File Name: Supplementary Data 8
Description: Crystallographic information file for compound 42
